# Enhanced Thermal Resilience of Olive Oils: Fatty Acid Dynamics with Polyphenols Supplementation

**DOI:** 10.3390/foods14122085

**Published:** 2025-06-13

**Authors:** Taha Mehany, José M. González-Sáiz, Consuelo Pizarro

**Affiliations:** Department of Chemistry, University of La Rioja, 26006 Logroño, Spain; taha.abdellatif@unirioja.es (T.M.); josemaria.gonzalez@unirioja.es (J.M.G.-S.)

**Keywords:** chemometrics, deep-frying, extra virgin olive oil, fatty acid profile, gas chromatography, hydroxytyrosol supplementation, olive oil quality, thermal stability, vegetable oils, principal component analysis

## Abstract

This study investigates the impact of hydroxytyrosol (HTyr) supplementation on the fatty acid profiles and oxidative stability of various extra virgin olive oil (EVOO) cultivars and other edible oils during prolonged deep-frying. EVOO cultivars including Picual, Cornicabra, Empeltre, Arbequina, Hojiblanca, Manzanilla, Royuela, Koroneiki, and Arbosana were analyzed alongside two sunflower oils and three refined olive oils under thermal stress at 170–210 °C for 3–6 h. HTyr consistently preserved monounsaturated fatty acids (MUFAs), particularly oleic acid (C18:1), while significantly reducing the degradation of polyunsaturated (PUFAs) and saturated fatty acids (SFAs) (*p* < 0.05) in many oil samples; for example, in olive oil °1, TMUFAs in Exp 1 revealed 7.28%, while in Exp 5 (with HTyr), TMUFAs increased to 7.47%. In olive oil °0.4, TMUFAs increased from 8.52% in Exp 1 to 9.17% in Exp 5. Additionally, In EVOO cv. Picual, total SFAs increased slightly, from 16.58% in Exp 1 to 16.96%, in Exp 5. Notably, total MUFA content (TMUFAs) was best preserved in Manzanilla (81.92%), followed by Hojiblanca (78.52%), Empeltre (78.09%), olive oil 1° (78.20%), Koroneiki (77.60%), and Arbosana (77.01%) (*p* < 0.05), indicating strong oxidative resistance. In Arbequina and Royuela oils, oleic acid retention also exceeded 76% after deep-frying. HTyr helped maintain fatty acid profiles within EU regulatory limits across most cultivars, despite minor exceedances in specific SFAs, such as lignoceric acid (C24:0), likely due to varietal traits or harvest timing. Principal component analysis (PCA) revealed distinct clustering patterns: sunflower oils grouped around linoleic acid (C18:2), reflecting high PUFA content, while olive oils clustered near oleic and palmitic acids. Cultivars such as Picual, Empeltre, Manzanilla, and Royuela showed unique associations with lignoceric acid, supporting the use of fatty acid profiles as cultivar-specific markers. HTyr supplementation enhanced oxidative stability and quality retention across oil types in terms of fatty acids profile, corroborating previous findings on the resilience of polyphenol-rich EVOOs under thermal stress. Furthermore, fatty acid composition varied significantly according to cultivar, HTyr, and deep-frying (*p* < 0.05), highlighting the complexity of oil quality determinants. This study supports the application of HTyr as a natural antioxidant to improve thermal stability and nutritional quality, not only in olive oils but also in other edible oils. These findings promote sustainable practices aligned with circular economy principles and advance the understanding of fatty acid dynamics during deep-frying. HTyr-enriched oils present promising potential in both culinary and industrial contexts.

## 1. Introduction

Olive oil (OO), especially extra virgin olive oil (EVOO), is a cornerstone of the Mediterranean diet (MD), valued both for its culinary versatility and significant health benefits. Its high content of monounsaturated fatty acids (MUFAs), primarily oleic acid, enhances its oxidative stability and nutritional profile. Serving as a functional food, EVOO delivers numerous bioactive compounds that contribute to improved serum lipid profiles and reduce oxidative damage to low-density lipoproteins (LDL) and free radicals [1,2]. Cardiovascular diseases (CVDs) remain a leading cause of mortality worldwide, with diet being a major modifiable risk factor. Among various dietary strategies, the MD has gained considerable attention for its protective effects against CVDs. A distinguishing feature of the MD is its primary fat source—OO, particularly EVOO. Due to its fatty acid profile—mainly oleic acid—and its rich content of bioactive compounds such as polyphenols, olive oil contributes significantly to the MD’s cardioprotective effects. These fatty acids help regulate blood lipids, reduce inflammation, and improve vascular function, making OO a central element in the prevention and management of CVDs [3,4,5].

EVOO is highly valued for its exceptional nutritional quality, largely attributed to its complex composition of over 200 compounds. Its main component, oleic acid—a monounsaturated fatty acid—constitutes 70–80% of the oil. Minor compounds, such as polyphenols, squalene, tocopherols, chlorophyll, and carotenoids, although present in smaller quantities (1–2%), also contribute to EVOO’s health benefits. However, the primary pharmacological effects are mainly ascribed to oleic acid and polyphenols due to their potent antioxidant and anti-inflammatory properties [5].

Fatty acids (FAs) are essential lipid components, playing key roles in cell membrane structure and function, as well as serving as primary energy sources across all trophic levels. They are also critically involved in various biochemical and physiological processes, including neural function [6,7].

In the context of olive oil, FAs are particularly important due to their direct influence on the oil’s quality, nutritional value, and stability. Furthermore, the FA profile of olive oil serves as a key indicator of varietal characteristics, geographic origin, and environmental conditions. Because FAs reflect both metabolic activity and environmental influences, they are increasingly recognized as effective bio-indicators—not only in ecological studies but also in agricultural systems concerned with product authenticity and ecosystem health [8].

In the human body, FAs serve as vital energy sources, structural components of cell membranes, and regulators of metabolism, gene expression, and inflammation. They influence the risk and progression of various diseases, including cardiovascular disease, diabetes, and cancer. While traditionally classified by type (e.g., saturated, omega-3), it is now clear that individual fatty acids exert distinct biological effects, underscoring the need for more specific analytical approaches [7]. Additionally, FA composition is a key indicator of oil degradation, oxidative stability, and nutritional quality [9]. Lipid oxidation is a primary cause of oil and fat deterioration during processing and storage, leading to significant changes in key quality attributes, such as color, flavor, aroma, and nutritional value. This degradation primarily stems from the breakdown of essential fatty acids and the formation of potentially toxic compounds. Many of these oxidative byproducts are highly reactive and have been linked to adverse health outcomes, including cancer, atherosclerosis, cardiovascular diseases, and allergic reactions [9,10].

Thus, FAs play crucial roles in physiological and ecological processes, including energy storage, membrane structure, and cellular signaling pathways. They also serve as important indicators of metabolic activity and environmental adaptation.

Phenolic compounds within EVOO are crucial for preventing lipid oxidation, prolonging shelf life, and imparting its distinctive bitterness, astringency, and sensory appeal. Beyond these roles, they offer protective effects against diseases such as coronary heart disease, cancer, and other stress-related conditions, with their effectiveness dependent on bioavailability and bioaccessibility. Additionally, these phenolics help regulate oxidative stress, preserve telomere length, and promote healthy aging through their impact on redox balance and epigenetic mechanisms [11,12,13].

Frying, one of the oldest cooking methods, is widely used in both domestic and industrial settings due to the desirable sensory qualities it imparts to food [14]. This process involves immersing food in edible oil heated between 150 °C and 200 °C or applying a thin layer of oil between a heated surface and the food until it achieves the desired color, texture, and flavor. While valued for its culinary benefits, frying is fundamentally governed by the principles of heat and mass transfer [15]. However, during high-temperature cooking methods such as deep-frying, EVOO undergoes chemical transformations that can degrade its quality and health-promoting properties [16]. Additionally, thermal oxidation during frying produces hydroperoxides as primary oxidation products, which degrade into volatile secondary compounds like aldehydes and ketones, which are responsible for rancid flavors. Some of these secondary products are toxic to human health. Increased total polar compounds, linked to oxidized triglyceride monomers, serve as indicators of oil degradation and potential safety risks [15,17].

Hydroxytyrosol (HTyr), a potent polyphenol derived from olives, exhibits a wide range of health benefits supported by both preclinical and clinical studies. It plays a significant role in cardiovascular health by reducing arterial inflammation and atherosclerotic plaque microcalcification, particularly in the elderly, and may improve lipid profiles and glucose metabolism. HTyr also demonstrates strong antioxidant and anti-inflammatory properties, helping to combat oxidative stress and regulate apoptosis, which are crucial in managing chronic diseases such as cardiovascular conditions, osteoarthritis, and neurodegenerative disorders. Additionally, it supports bone, joint, and cognitive health, and shows promise in cancer prevention and therapy, particularly in neuroblastoma, by inducing apoptosis, enhancing antioxidant defenses, and potentially reducing the side effects of chemotherapy. HTyr’s antimicrobial, antiviral, skin-protective, and wound-healing effects further underscore its therapeutic versatility. While current findings are encouraging, further research is necessary to establish optimal dosing, long-term efficacy, and broader clinical applications [18,19,20,21,22].

Recent studies have explored the supplementation of EVOO with natural antioxidants to enhance its stability under thermal stress [23,24]. HTyr, a potent phenolic compound derived from olive fruit, has garnered attention for its ability to inhibit lipid oxidation and preserve the oil’s integrity during frying processes [25]. For instance, Mehany et al. [26] investigated the effects of enriching EVOO with olive fruit extract (OFE) containing HTyr during prolonged deep-frying at temperatures of 170 °C and 210 °C. Their findings indicated that HTyr supplementation effectively maintained the oil’s quality parameters, such as peroxide value and acidity, within acceptable limits over extended frying durations. Furthermore, the addition of HTyr has been shown to preserve the sensory attributes of fried olive oils. A study assessing the sensorial markers in deep-fried EVOO supplemented with natural exogenous antioxidants from olive fruit extract enriched in HTyr demonstrated a reduction in rancidity and an enhancement in positive sensory characteristics [27]. These findings underscore the potential of HTyr to not only improve the oxidative stability of EVOO but also to enhance the overall quality of fried foods.

Given the critical functional roles of FAs, understanding their thermal dynamics offers valuable insight into the oxidative stability and degradation behavior of oils under high-temperature conditions. This knowledge is especially important for evaluating the suitability of edible oils for domestic and industrial frying applications. In particular, the fatty acid profile—especially the high oleic acid content in olive oil—directly influences both its nutritional value and thermal stability, making it a key parameter in assessing oil performance and health implications.

Despite existing research on lipid oxidation, important questions remain—such as whether HTyr, a potent natural antioxidant, and its derivatives can enhance the thermal stability of fatty acids during deep-frying. Furthermore, there is a lack of comparative studies investigating how different edible oils respond to high-temperature frying when supplemented or not with exogenous polyphenols.

This study aims to address these gaps in the following ways:

1. The changes in fatty acid composition are monitored across various olive oil categories—including EVOOs, blends of EVOO, virgin olive oil with refined olive oil, and olive pomace olive oil—supplemented with hydroxytyrosol.
2. These results are compared with those from two types of sunflower oil under identical deep-frying conditions.
3. The protective effects of hydroxytyrosol on key fatty acids during thermal exposure are evaluated.
4. Fatty acid profiles in oils are evaluated without frying to establish baseline stability and the impact of supplementation independent of thermal stress.

The novelty of this research lies in its comprehensive and comparative analysis of fatty acid degradation patterns under realistic frying conditions alongside baseline evaluations, coupled with the exploration of exogenous HTyr as a natural stabilizer. This approach provides new insights into strategies for improving the oxidative resilience and nutritional quality of edible oils.

## 2. Materials and Methods

### 2.1. Materials and Samples

The following chemicals and reagents were used in the present study: n-Heptane (≥99%), for gas chromatography was brought from VWR (VWR International, Rosny-sous-Bois, France). LC-MS grade–methanol with ≥99.9% purity was supplied by Fisher Scientific Ltd. (Loughborough, UK). Moreover, 4-methyl 2-pentanol with ≥99% purity, potassium hydroxide (≥85%), and analytical standards for palmitic acid (methyl palmitate, ≥98.5%), stearic acid (methyl stearate, ≥99%), oleic acid (methyl oleate, 99%), and linoleic acid (methyl linoleate, ≥98%) were purchased from Sigma-Aldrich (Saint Louis, MO, USA). Ultrapure water was obtained from a MilliQ system (Millipore, Bedford, MA, USA).

Olive fruit dry extract (20% hydroxytyrosol) was sourced from Natac BioTech (Madrid, Spain). This study examined various vegetable oils obtained from local Spanish suppliers, including nine EVOOs from different cultivars (Picual, Cornicabra, Empeltre, Arbequina, Hojiblanca, Manzanilla Cacereña, Royuela/Arróniz, Koroneiki, and Arbosana), one EVOO blended with refined olive oil (ROO), labeled “olive oil 1°”, one pomace olive oil (a mix of refined pomace oil and EVOO, known in Spain as Orujo oil), and one virgin olive oil (VOO) blended with ROO, labeled “olive oil 0.4°”, as well as refined sunflower oil and refined high-oleic sunflower oil.

For each olive oil category, 11 samples were analyzed: the original non-fried oil (Control 1), non-fried oil supplemented with olive fruit extract (OFE), a 1:1 mixture of original and supplemented oils (Control 2), and eight samples (Table S1) subjected to deep-frying (D-F), as shown in the full factorial experimental design, under varying conditions (differences in time, temperature, and the addition of polyphenol). Each sunflower oil category included five samples (Table S2). In total, 142 samples were evaluated, comprising both control and deep-fried oils.

### 2.2. Exogenous Polyphenol Supplementation of Olive Oil

To enhance their antioxidant and health-related properties, each olive oil category was fortified with a hydroxytyrosol-rich OFE. The process involved supplementing the original oils (EVOO, VOO, or refined olive oils, referred to as Control 1) to produce a polyphenol-enriched oil. This enriched oil was then blended with the original oil in equal parts to create Control 2.

It is well established that HTyr is a highly polar, water-soluble compound with limited solubility in lipid matrices. To overcome this limitation, we adopted a supplementation approach using an aqueous olive fruit extract, which facilitated the incorporation of HTyr into the oil phase. This method, previously described and validated in our recent works [25,27], effectively enriched olive oil with HTyr and its derivatives without the use of synthetic additives or adulteration. As shown in these studies, the supplementation process allows for the natural enrichment of olive oil with bioactive phenolics.

Following the method described by Mehany et al. [27], 40 g of OFE was added to 400 g of H_2_O (10% *w*/*v*). Then, the solution was stirred (magnetic stirrer, IKA-WERKE, Staufen, Germany) at ambient temperature for 30 min. Subsequently, 200 g of this aqueous solution was mixed with 500 g of olive oil (OFE aqueous solution and olive oil ratio of 2:5 *w*/*w,* respectively), and the mixture was stirred mechanically for 60 min. The prepared solution was then centrifuged at 9961× *g* for 20 min using a Sorvall RC-6 Plus centrifuge (Dreieich, Germany). The resulting supplemented oil was stored in amber containers at 7 ± 2 °C until further analysis. Table S3 details the polyphenol concentrations of the original, supplemented, and blended oils, with levels reaching up to 650 mg/kg.

### 2.3. Deep-Frying Process

Different categories of olive and sunflower oils were subjected to deep-frying (D-F) using a 0.5 L flask equipped with a Soxhlet heating system (SELECTA, Barcelona, Spain). For each experiment, 0.4 L of oil was heated continuously at two temperatures—170 ± 10 °C and 210 ± 10 °C—for durations of 3 and 6 h. After each D-F cycle, 400 mL of oil was collected in amber glass vials to assess degradation and oxidative changes under varying conditions, including oil type, frying time, temperature, and the presence of natural antioxidants. Samples were stored at 5 °C in the dark to prevent further oxidation prior to analysis [1].

### 2.4. Fatty Acid Quantification by Gas Chromatography

Fatty acid composition (FAC) was determined using a conventional methylation method, as described by Wen et al. [28]. Briefly, 0.2 g of oil was dissolved in 4 mL of heptane in a 10 mL centrifuge tube. The tube was sealed and vigorously shaken for 30 s at room temperature (RT) using a Heidolph shaker (Schwabach, Germany). For methyl esterification, 400 µL of 2N methanolic KOH was added, followed by another 30 s shake. The mixture was then left to stand at RT for 30 min, resulting in two distinct layers. The upper phase, containing fatty acid methyl esters (FAMEs), was collected using a Pasteur pipette and transferred to chromatography vials.

Gas chromatography (GC) analysis was performed using an Agilent 6890 N Network GC System (USA) equipped with an autosampler (7683 Series), split injector, and flame ionization detector (FID). An HP-WAX capillary column (Crosslinked Polyethylene Glycol; 30 m length, 0.25 mm diameter, 0.25 μm film thickness; HP Part No. 199091X-133, U.S. Patent No. 4.293.415) was used.

GC conditions were as follows: injector and detector temperatures were both set at 250 °C, and the column oven temperature at 260 °C. A 0.5 µL sample volume was injected. The oven temperature program began at 120 °C (held for 4 min), before increasing to 180 °C at 25 °C/min (held for 6.4 min), and then to 280 °C at 7 °C/min (held for 24.4 min). Helium was used as the carrier gas at a flow rate of 43.3 mL/min.

The identification of FAMEs was achieved by comparing retention times with those of external standards: methyl palmitate (C16:0), methyl stearate (C18:0), methyl oleate (C18:1), and methyl linoleate (C18:2). Standard solutions (0.5 µL each) were prepared by dissolving 0.205 g of each ester in 25 mL of heptane to obtain a 1.2% (*w*/*w*) solution, which was stable for up to three months when stored at 4 °C.

The elution sequence was confirmed as follows: methyl palmitate, methyl stearate, methyl oleate, and methyl linoleate. The same injection volume (0.5 µL) was used for the esterified oil samples.

For quantification, the internal standard method was applied using 4-methyl-2-pentanol. A known amount (1.5 µg) of the internal standard was added to each sample and to the standard solution. Relative response factors (RRFs) were calculated, and quantitative analysis was performed using the equation provided below.

(1) $$Fatty\;acids\;\left(\frac{mg}{kg}\right)=\frac{Areas\;of\;each\;component\;peak\times Weight\;of\;inetrnal\;standard}{RRF\times Area\;of\;inetrnal\;standard}\;$$

where the relative response factor (RRF) is the multiplication coefficient of the response factor (RF) for the internal standard (4-methyl-2-pentanol) and the RF for the external standard (oleic acid). The RF for 4-methyl-2-pentanol is calculated as the ratio of the area of 4-methyl-2-pentanol to the concentration of the injected 4-methyl-2-pentanol. Similarly, the RF for the external standard is calculated as the ratio of the area of the external standard to the concentration of the injected external standard. The RFF for 4-methyl-2-pentanol and the external standard is determined as the ratio of the RF for 4-methyl-2-pentanol to the RF for the external standard.

### 2.5. Preparation of External Calibration Standards and Method Validation

For validation purposes, the results were expressed in terms of oleic acid. A standard oleic acid stock solution (1 mg/mL) was prepared by dissolving 10 mg of methyl oleate in 10 mL of heptane. This stock solution was then used to prepare a series of standards with concentrations ranging from 0.008 to 0.558 mg/mL.

**Figure 1:**
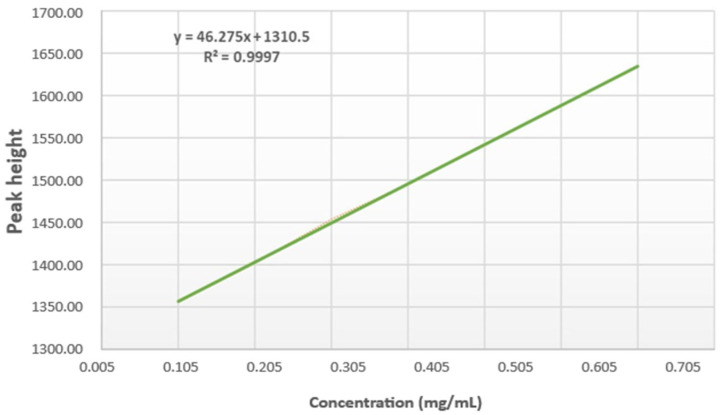
Methyl oleate calibration curve used for the validation of the GC method.

The calibration curve for the GC analysis was constructed by plotting the ratio of the tyrosol peak area against its concentration levels (Figure 1). The peak area was directly proportional to the methyl oleate concentration, and the calibration curve was described by the following regression equation:

(2) y=46.275x+1310.5

where *x* represents the concentration of methyl oleate (mg/mL), and *y* represents the peak height. The peak height exhibited a linear relationship with the methyl oleate concentration within the range of 0.008–0.558 mg/mL.

The calculated limits of detection (LOD) and quantification (LOQ) were 0.3764 mg/kg and 1.1407 mg/kg, respectively, with a coefficient of determination (R^2^) value of 0.9997. These results demonstrate the method’s high sensitivity and strong linearity across the specified range. Regarding accuracy (trueness and precision), each oil sample was analyzed in triplicate. The standard deviation was calculated to evaluate the agreement among multiple measurements from homogeneous samples. This assessment confirmed the method’s precision, reproducibility, and repeatability, ensuring the reliability of the results.

### 2.6. Data Analyses

All analyses were performed in triplicate, and results are expressed as mean ± standard deviation (SD). Statistical significance was evaluated using one-way analysis of variance (ANOVA) in SPSS (version 28; IBM SPSS Statistics, Chicago, IL, USA), followed by Tukey’s post hoc test for multiple comparisons. Differences were considered statistically significant at *p* < 0.05. Additionally, principal component analysis (PCA), including scores and loadings (biplot), was conducted using OriginLab 2025 software (OriginLab Corporation, Northampton, MA, USA).

## 3. Results and Discussion

### 3.1. Fatty Acids of EVOOs Picual, Cornicabra, Empeltre, and Arbequina

Under deep-frying conditions, the fatty acid profiles of EVOO cultivars Picual (Table 1), Cornicabra (Table S4), Empeltre (Table S5), and Arbequina (Table S6) show that HTyr supplementation offers a consistent protective effect by preserving monounsaturated fatty acids (MUFAs), particularly oleic acid (C18:1), while limiting the degradation of polyunsaturated (PUFAs) and saturated fatty acids (SFAs). In Picual, HTyr helped maintain high MUFA levels (up to ~71–73%) and slightly reduced SFAs, although certain fatty acids like C14:0 and C24:0 exceeded the EU limits—likely due to cultivar-specific traits. Similarly, in Empeltre, HTyr led to a marked drop in SFAs such as C14:0, C17:0, and C18:0, with C14:0 even becoming undetectable, while boosting oleic acid content from 69.98% in Control 2 to 78.09% in Exp. 8, and thus reducing total SFAs from 20.11% to 10.79%. Cornicabra showed comparable trends with MUFAs rising from 67.10% to 68.02% in Control 1 and Exp. 4, respectively, and SFAs declining from 21.56% in Control 2 to 18.62% in Exp. 8, although the changes were less pronounced than in Empeltre. Across all three cultivars, HTyr supplementation maintained fatty acid profiles within EU regulatory limits, highlighting its role in enhancing oxidative stability and preserving oil quality during high-temperature frying, with the strongest protective effects observed in Empeltre. Furthermore, in Arbequina, a cultivar already rich in MUFAs and low in SFAs, HTyr helped stabilize oleic acid (up to 72.65%) and reduce stearic acid (C18:0), although regulatory exceedances in minor SFAs (C14:0, C24:0) persisted. Hojiblanca, with its inherently stable fatty acid profile, showed minimal response to HTyr—TMUFAs levels remained high (76.25–78.52% in Exp. 4 and Exp. 8, respectively) with only modest SFA reductions—highlighting its natural thermal resilience.

**Figure 2:**
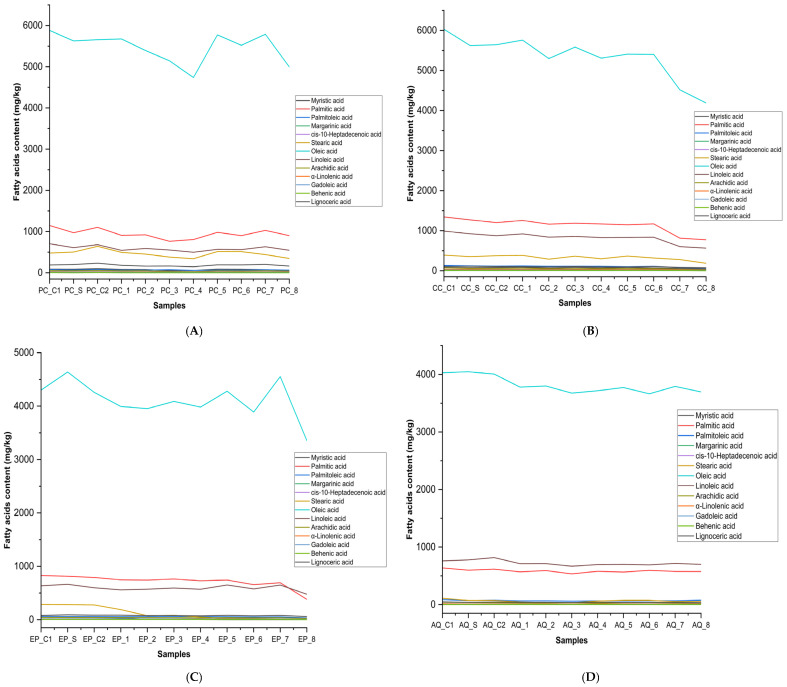
Fatty acid dynamics (mg/kg) under deep-frying conditions with HTyr supplementation in four extra virgin olive oil varieties: (**A**) Picual, (**B**) Cornicabra, (**C**) Empeltre, and (**D**) Arbequina. PC_C1: Picual_Control 1; PC_S: Picual_Supplemented; PC_C2: Picual_Control 2; PC_1: Picual_Exp 1; PC_2: Picual_Exp 2; PC_3: Picual_Exp 3; PC_4: Picual_Exp 4; PC_5: Picual_Exp 5; PC_6: Picual_Exp 6; PC_7: Picual_Exp 7; PC_8: Picual_Exp 8; CC_C1: Cornicabra_Control 1; CC_S: Cornicabra_Supplemented; CC_C2: Cornicabra_Control 2; CC_1: Cornicabra_Exp 1; CC_2: Cornicabra_Exp 2; CC_3: Cornicabra_Exp 3; CC_4: Cornicabra_Exp 4; CC_5: Cornicabra_Exp 5; CC_6: Cornicabra_Exp 6; CC_7: Cornicabra_Exp 7; CC_8: Cornicabra_Exp 8; EP_C1: Empeltre_Control 1; EP_S: Empeltre_Supplemented; EP_C2: Empeltre_Control 2; EP_1: Empeltre_Exp 1; EP_2: Empeltre_Exp 2; EP_3: Empeltre_Exp 3; EP_4: Empeltre_Exp 4; EP_5: Empeltre_Exp 5; EP_6: Empeltre_Exp 6; EP_7: Empeltre_Exp 7; EP_8: Empeltre_Exp 8; AQ_C1: Arbequina_Control 1; AQ_S: Arbequina_Supplemented; AQ_C2: Arbequina_Control 2; AQ_1: Arbequina_Exp 1; AQ_2: Arbequina_Exp 2; AQ_3: Arbequina_Exp 3; AQ_4: Arbequina_Exp 4; AQ_5: Arbequina_Exp 5; AQ_6: Arbequina_Exp 6; AQ_7: Arbequina_Exp 7; AQ_8: Arbequina_Exp 8.

Recent studies focused on enhancing the biostability of EVOO supplemented with olive fruit extract enriched with HTyr during prolonged deep-frying. The research demonstrated that HTyr supplementation improved the sensorial attributes, polyphenolic content, and thermal oxidative stability of the oil [26,27]. These studies concluded that HTyr-enriched EVOOs exhibited exceptional thermal stability, showing low hydrolysis, low oxidation, enhanced antioxidant activity, and elevated levels of chlorophyll and carotenoids during deep-frying at 170 °C for 3 h. Moreover, the difference in fatty acid profiles among these cultivars (Figure 2) in the present study is probably due to varying agronomic practices. A comparative analysis of 68 monovarietal EVOOs from Greece, harvested in 2018–2019, revealed significant variations in fatty acid composition and antioxidant content based on both cultivar and geographic origin. The two cultivars examined—Koroneiki (from Peloponnese and Crete) and Kolovi (from Lesvos)—demonstrated distinct chemical profiles. Kolovi samples contained higher concentrations of γ-tocopherol, linoleic acid (C18:2), linolenic acid (C18:3), and gadoleic acid (C20:1), whereas Koroneiki oils were richer in squalene, palmitic acid (C16:0), palmitoleic acid (C16:1), and arachidic acid (C20:0). Furthermore, tocopherol content showed geographic dependency, with α-tocopherol levels peaking in oils from Peloponnese and γ-tocopherol being most abundant in samples from Lesvos. Multivariate analysis identified γ-tocopherol and specific fatty acids—linoleic, linolenic, gadoleic, palmitic, palmitoleic, and arachidic acids—as key discriminant markers, effectively differentiating EVOOs by both cultivar and region [29].

**Table 1:** Fatty acids content (%) of supplemented and non-supplemented EVOO cv. Picual with HTyr under deep-frying conditions compared to the standard limits.

Fatty Acid	Con 1	SOO	Con 2	Exp 1	Exp 2	Exp 3	Exp 4	Exp 5	Exp 6	Exp 7	Exp 8	Standard Limits *
C14:0	1.03 ± 0.04 ^b^	1.07 ± 0.03 ^a^	1.19 ± 0.06 ^a^	1.03 ± 0.04 ^b^	1.03 ± 0.03 ^b^	0.74 ± 0.03 ^d^	0.85 ± 0.04 ^c^	1.09 ± 0.01 ^a^	1.09 ± 0.02 ^a^	0.92 ± 0.03 ^c^	0.85 ± 0.02 ^d^	≤0.03
C16:0	13.20 ± 0.11 ^a^	11.87 ± 0.09 ^a^	12.77 ± 0.03 ^b^	11.38 ± 0.11 ^a^	11.85 ± 0.03 ^a^	10.61 ± 0.05 ^c^	12.02 ± 0.08 ^a^	12.01 ± 0.05 ^a^	11.36 ± 0.08 ^b^	12.31 ± 0.12 ^a^	12.56 ± 0.04 ^a^	7.00–20.00
C16:1	0.91 ± 0.05 ^b^	0.85 ± 0.03 ^b^	0.92 ± 0.01 ^b^	0.83 ± 0.05 ^b^	0.84 ± 0.03 ^b^	1.05 ± 0.04 ^a^	0.89 ± 0.03 ^b^	0.81 ± 0.03 ^b^	0.81 ± 0.01 ^b^	0.88 ± 0.03 ^b^	0.91 ± 0.03 ^b^	0.30–3.50
C17:0	0.08 ± 0.00 ^a^	0.08 ± 0.01 ^a^	0.09 ± 0.02 ^a^	0.10 ± 0.00 ^a^	0.10 ± 0.03 ^a^	0.04 ± 0.01 ^b^	0.06 ± 0.02 ^ab^	0.10 ± 0.01 ^a^	0.07 ± 0.00 ^b^	0.09 ± 0.01 ^ab^	0.09 ± 0.01 ^a^	≤0.40
C17:1	0.17 ± 0.01 ^a^	0.16 ± 0.02 ^a^	0.19 ± 0.03 ^a^	0.17 ± 0.01 ^a^	0.17 ± 0.01 ^a^	0.11 ± 0.03 ^ab^	0.13 ± 0.02 ^ab^	0.17 ± 0.04 ^a^	0.15 ± 0.00 ^a^	0.16 ± 0.01 ^a^	0.18 ± 0.02 ^a^	≤0.60
C18:0	5.50 ± 0.04 ^bc^	6.16 ± 0.11 ^a^	7.44 ± 0.08 ^a^	6.21 ± 0.04 ^bc^	5.83 ± 0.06 ^ab^	5.30 ± 0.04 ^b^	5.09 ± 0.03 ^b^	6.30 ± 0.03 ^a^	6.46 ± 0.03 ^a^	5.28 ± 0.03 ^b^	4.86 ± 0.03 ^c^	0.50–5.00
C18:1	67.66 ± 0.35 ^ab^	68.77 ± 0.10 ^ab^	65.50 ± 0.28 ^b^	71.13 ± 0.45 ^a^	69.34 ± 0.27 ^a^	71.41 ± 0.12 ^a^	70.50 ± 0.09 ^a^	70.61 ± 0.05 ^a^	69.56 ± 0.03 ^b^	69.26 ± 0.09 ^a^	69.54 ± 0.14 ^a^	55.00–85.00
C18:2	8.10 ± 0.09 ^a^	7.46 ± 0.03 ^a^	7.91 ± 0.01 ^a^	6.82 ± 0.09 ^a^	7.60 ± 0.04 ^a^	7.66 ± 0.03 ^a^	7.41 ± 0.05 ^a^	6.97 ± 0.09 ^ab^	7.09 ± 0.13 ^a^	7.56 ± 0.03 ^a^	7.60 ± 0.02 ^a^	2.50–21.00
C20:0	0.60 ± 0.03 ^a^	0.62 ± 0.03 ^a^	0.68 ± 0.03 ^a^	0.61 ± 0.03 ^a^	0.65 ± 0.03 ^a^	0.44 ± 0.03 ^ab^	0.45 ± 0.06 ^ab^	0.60 ± 0.03 ^a^	0.56 ± 0.04 ^a^	0.61 ± 0.01 ^a^	0.60 ± 0.03 ^a^	≤0.60
C18:3	0.31 ± 0.02 ^a^	0.29 ± 0.05 ^a^	0.32 ± 0.01 ^a^	0.31 ± 0.02 ^a^	0.31 ± 0.06 ^a^	0.19 ± 0.00 ^b^	0.23 ± 0.03 ^ab^	0.30 ± 0.01 ^a^	0.26 ± 0.01 ^a^	0.30 ± 0.03 ^a^	0.31 ± 0.04 ^a^	≤1.00
C20:1	0.23 ± 0.03 ^a^	0.21 ± 0.03 ^a^	0.23 ± 0.03 ^a^	0.22 ± 0.03 ^a^	0.22 ± 0.01 ^a^	0.19 ± 0.03 ^a^	0.19 ± 0.03 ^a^	0.22 ± 0.03 ^a^	0.19 ± 0.03 ^a^	0.22 ± 0.03 ^a^	0.23 ± 0.03 ^a^	≤0.50
C22:0	0.05 ± 0.00 ^a^	ND	0.07	ND	ND	ND	ND	ND	ND	ND	ND	≤0.20
C24:0	2.17 ± 0.01 ^c^	2.41 ± 0.00 ^a^	2.69 ± 0.05 ^a^	2.27 ± 0.01 ^c^	2.06 ± 0.03 ^b^	2.27 ± 0.02 ^ab^	2.20 ± 0.03 ^b^	2.36 ± 0.07 ^a^	2.39 ± 0.05 ^a^	2.42 ± 0.05 ^a^	2.26 ± 0.03 ^b^	≤0.20
TSFAs	22.63 ± 0.17 ^ab^	22.25 ± 0.04 ^ab^	24.94 ± 0.03 ^a^	21.61 ± 0.17 ^b^	21.51 ± 0.13 ^b^	19.39 ± 0.09 ^b^	20.66 ± 0.23 ^b^	22.46 ± 0.08 ^ab^	21.94 ± 0.11 ^ab^	21.61 ± 0.09 ^ab^	21.22 ± 0.62 ^ab^	
TMFAs	68.79 ± 0.81 ^a^	70.00 ± 0.65 ^b^	66.84 ± 0.47 ^b^	72.36 ± 0.81 ^a^	70.57 ± 0.22 ^ab^	72.75 ± 0.13 ^a^	71.70 ± 0.08 ^a^	71.81 ± 0.21 ^a^	70.71 ± 0.09 ^b^	70.52 ± 0.12 ^b^	70.86 ± 0.06 ^a^	
TPUFAs	8.41 ± 0.08 ^a^	7.75 ± 0.03 ^a^	8.23 ± 0.03 ^a^	7.12 ± 0.08 ^a^	7.92 ± 0.03 ^a^	7.86 ± 0.03 ^a^	7.64 ± 0.03 ^a^	7.27 ± 0.03 ^a^	7.35 ± 0.03 ^a^	7.86 ± 0.03 ^a^	7.92 ± 0.03 ^a^	

^a,b,c,d^ Data in the same row followed by different superscript letters differ significantly (*p* < 0.05). ND: not detected. Con 1 (used as the control for Experiments 1–4) refers to original, non-deep-fried olive oil. SOO (supplemented olive oil) refers to non-deep-fried olive oil that has been enriched with olive fruit extract, which is also used in the preparation of Con 2. Con 2 (used as the control for Experiments 5–8) is a mixture of Con 1 and the supplemented oil, resulting in a total polyphenol content of up to 650 mg/kg. Exp.1: olive oil deep-fried at 170 °C for 3 h without polyphenol supplementation; Exp.2: olive oil deep-fried at 170 °C for 6 h without polyphenol supplementation; Exp.3: olive oil deep-fried at 210 °C for 3 h without polyphenol supplementation; Exp.4: olive oil deep-fried at 210 °C for 6 h without polyphenol supplementation; Exp.5: olive oil deep-fried at 170 °C for 3 h with polyphenol supplementation; Exp.6: olive oil deep-fried at 170 °C for 6 h with polyphenol supplementation; Exp.7: olive oil deep-fried at 210 °C for 3 h with polyphenol supplementation; Exp.8: olive oil deep-fried at 210 °C for 6 h with polyphenol supplementation. C14:0 (myristic acid); C16:0 (palmitic acid); C16:1 (n−7) (palmitoleic acid); C17:0 (margarinic acid); C17:1 (n−7) (cis-10-heptadecenoic acid); C18:0 (stearic acid); C18:1 (n−9) (oleic acid); C18:2 (n−6) (linoleic acid); C20:0 (arachidic acid); C18:3 (n−3) (α-linolenic acid); C20:1 (n−9) (gadoleic acid); C22:0 (behenic acid); C24:0 (lignoceric acid). TSFAa: total saturated fatty acids; TMUFAs: total monounsaturated fatty acids; TPUFAs: total polyunsaturated fatty acids; TSFAs (C14:0 + C16:0 + C17:0 + C18:0 + C20:0 + C22:0 + C24:0); TMUFAs (C16:1 + C18:1 + C20:1); TPUFAs (C18:2 + C18:3). * Standard limits (% m/m methyl esters): legally established ranges (European Union Commission). EU Regulation 2019/1604 Amending Commission Regulation (EEC) No 2568/91 on the Characteristics of Olive Oil and Olive-Residue Oil and on the Relevant Methods Analysis; European Commission: Brussels, Belgium, 2019 [30].

### 3.2. Fatty Acids of EVOO Hojiblanca, EVOO Manzanilla, EVOO Royuella, and Pomace Olive Oil (Orujo)

When evaluating the effects of HTyr supplementation on EVOO across multiple cultivars—Picual, Cornicabra, Empeltre, Arbequina, Hojiblanca (Table S7), Manzanilla (Table 2), and Royuella (Table 3)—under deep-frying conditions, a consistent protective trend emerges, especially in the preservation of MUFAs like oleic acid (C18:1) and mitigation of the degradation of saturated (SFAs) and PUFAs.

In addition, the Manzanilla cultivar demonstrated strong monounsaturated fatty acid (MUFA) preservation (up to 81.92% in Experiment 6) and improved saturated fatty acid (SFA) stability when supplemented with hydroxytyrosol (HTyr). However, lignoceric acid (C24:0) consistently exceeded acceptable limits, suggesting this may be an inherent varietal trait. Lignoceric acid (tetracosanoic acid) is a saturated fatty acid commonly found in plant-derived lipids and has been previously identified in *Populus tremuloides* [31]. It can be readily reduced to lignoceryl alcohol, although its biological activities remain largely unexplored. Interestingly, lignoceric acid may serve as a potential chromatographic fingerprint for polyphenol-enriched olive oils.

In Royuela, oleic acid (C18:1) remained stable and high (72.47–75.95%), with TPUFAs also remaining steady (~9.49–10.35%). Moreover, TSFAs showed a slight increase. Additionally, in Orujo oil, a non-EVOO base oil, the fatty acid composition remained within regulatory boundaries (Table S8), with C18:1 and linoleic acid (C18:2) levels showing minimal change. However, C14:0 slightly exceeded the EU limit (up to 0.07% in Exp 7), and occasional overages in minor SFAs like C20:0 and C24:0 were observed, indicating that HTyr’s protective effect was less consistent than in EVOO due to Orujo’s distinct composition.

**Figure 3:**
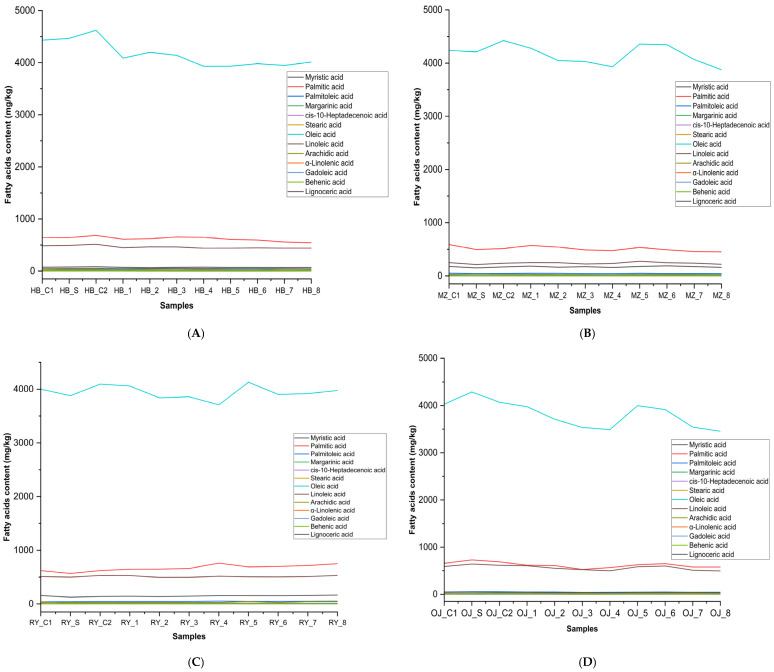
Fatty acid dynamics (mg/kg) under deep-frying conditions with HTyr supplementation in four extra virgin olive oil varieties: (**A**) Hojiblanca, (**B**) Manzanilla, and (**C**) Royuella, as well as (**D**) Pomace olive oil (Orujo). HB_C1: Hojiblanca_Control 1; Hojiblanca_Supplemented; Hojiblanca_Control 2; HB_1: Hojiblanca_Exp 1; HB_2: Hojiblanca_Exp 2; HB_3: Hojiblanca_Exp 3; HB_4: Hojiblanca_Exp 4; HB_5: Hojiblanca_Exp 5; HB_6: Hojiblanca_Exp 6; HB_7: Hojiblanca_Exp 7; HB_8: Hojiblanca_Exp 8; MZ_C1: Manzanilla_Control 1; MZ_S: Manzanilla_Supplemented; MZ_C2: Manzanilla_Control 2; MZ_1: Manzanilla_Exp 1; MZ_2: Manzanilla_Exp 2; MZ_3: Manzanilla_Exp 3; MZ_4: Manzanilla_Exp 4; MZ_5: Manzanilla_Exp 5; MZ_6: Manzanilla_Exp 6; MZ_7: Manzanilla_Exp 7; MZ_8: Manzanilla_Exp 8; RY_C1: Royuela_Control 1; RY_S: Royuela_Supplemented; RY_C2: Royuela_Control 2; RY_1: Royuela_Exp 1; RY_2: Royuela_Exp 2; RY_3: Royuela_Exp 3; RY_4: Royuela_Exp 4; RY_5: Royuela_Exp 5; RY_6: Royuela_Exp 6; RY_7: Royuela_Exp 7; RY_8: Royuela_Exp 8; OJ_C1: Pomace_Control 1; OJ_S: Pomace_Supplemented; OJ_C2: Pomace_Control 2; OJ_1: Pomace_Exp 1; OJ_2: Pomace_Exp 2; OJ_3: Pomace_Exp 3; OJ_4: Pomace_Exp 4; OJ_5: Pomace_Exp 5; OJ_6: Pomace_Exp 6; OJ_7: Pomace_Exp 7; OJ_8: Pomace_Exp 8.

The Manzanilla cultivar exhibited higher fatty acid stability, likely due to its naturally elevated polyphenol content, as shown in (Table 2). Additionally, the difference in fatty acid profiles among these varieties (Figure 3) is possibly due to varying agronomic practices. This highlights the importance of cultivar selection in maintaining oil stability and quality. A recent study by Mehany et al. [27] also reported that certain EVOOs possess distinctive sensory attributes compared to others. The results indicated that Arbequina, Picual, Royuella, Hojiblanca, Arbosana, and Manzanilla oils exhibited low rancidity scores—0.0, 1.7, 1.8, 2.3, 3.1, and 3.7, respectively—and maintained stable or enhanced positive sensorial attributes, such as fruity, bitter, and pungent notes. In contrast, olive oil 1° and olive oil 0.4° demonstrated higher rancidity and reduced positive sensory qualities. Furthermore, a particularly notable finding in this study is the elevated presence of lignoceric acid among the EVOO cultivars, which may be associated with early harvesting or the green ripening stage of the olives. This fatty acid appears to serve as a differentiating marker between cultivars, supporting our previous findings that Manzanilla and Royuella exhibit distinctive sensory profiles—characterized by pronounced fruity and pungent notes—potentially due to their unique fatty acid and polyphenol compositions.

HTyr has been shown to significantly enhance the oxidative stability of various oils. In Echium oil, HTyr increased the oxidative stability index (OSI) by up to threefold, demonstrating strong protective effects against oxidation comparable to rosemary extract, despite the oil’s high susceptibility to oxidative degradation [32]. Similarly, enzymatic modification of common vegetable oils like pomace olive and sunflower oil with HTyr—using immobilized lipase to acylate the antioxidant—increased the lipophilicity and bioactivity of HTyr, resulting in notably improved thermooxidative stability at 60 °C over 28 days. This modification was confirmed through multiple analytical and spectroscopic techniques, highlighting its ability to delay oxidation and spoilage. Moreover, this biocatalytic process leverages agro-industrial by-products, aligning with green chemistry and circular economy principles, and offers practical applications in food products by extending shelf life, despite HTyr’s inherent solubility and cost challenges [33].

### 3.3. Fatty Acids of EVOO Koroneiki, EVOO Arbosana, Olive Oil 1°, and Olive Oil 0.4°

For Koroneiki (Table S9), deep-frying led to slight reductions in TSFAs and maintained high TMUFA levels (75.11–77.60% in Exp. 7 and Exp. 3, respectively), reflecting good oxidative stability. Still, minor SFAs such as C14:0 and C24:0 exceeded EU thresholds—with C14:0 reaching 0.86% in control 2 and C24:0 exceeding 2%—likely due to varietal traits, cultivation period, and/or frying-induced changes. Moreover, while HTyr effectively supports fatty acid preservation during frying across these cultivars, especially in MUFA-rich oils like Royuela and Koroneiki, consistent exceedances of specific minor SFAs highlight the need for cultivar-specific evaluation when aiming to meet strict regulatory standards.

In addition, the application of HTyr supplementation across various olive oil types—including EVOO cv. Arbosana, 1° olive oil, and olive oil 0.4°—under deep-frying conditions reveals a consistent trend of improved fatty acid stability and regulatory compliance. In Arbosana (Table S10), oleic acid remained high (74.25–75.39% in Con 2 and Exp. 8, respectively), with SFAs such as palmitic and stearic within accepted limits, though the latter trended low (0.40–0.71%). Minor SFAs like myristic acid occasionally exceeded the ≤0.03% limit but declined with HTyr, suggesting a modest protective effect. PUFAs, including linoleic and linolenic acid, remained stable.

Similarly, 1° olive oil (Table S11) benefited from HTyr, with oleic acid increasing from 72.79% to 76.43% in Con 1 and Exp. 4, respectively, stearic acid dropping below 0.50%, and TSFAs decreasing from 18.82% to 14.36%, while total MUFAs rose, all indicating enhanced compositional quality. Myristic acid slightly exceeded limits in controls but improved with supplementation due to the fatty acid changes and dynamics during deep-frying and Hyty supplantation. In addition, olive oil 0.4° (Table S12) also showed improved profiles: HTyr supplementation reduced C14:0 and C18:0 levels, increased oleic acid content, and maintained PUFA stability, resulting in lowered TSFAs and elevated MUFAs.

**Figure 4:**
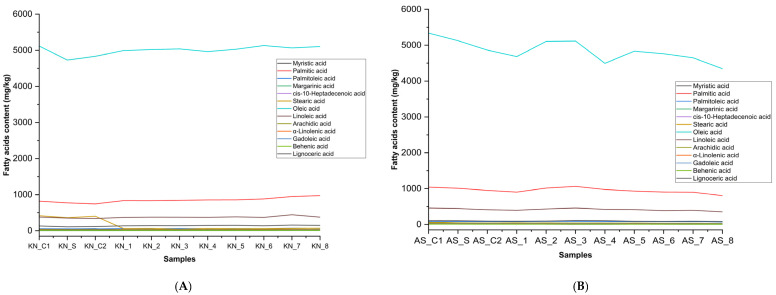

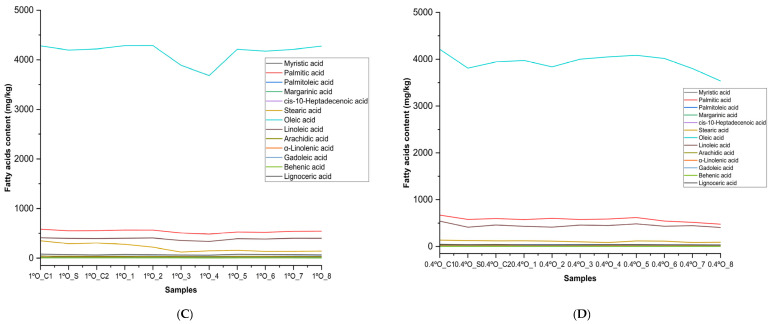
Fatty acid dynamics (mg/kg) during deep-frying with HTyr supplementation in various olive oils: (**A**) EVOO Koroneiki, (**B**) EVOO Arbosana, (**C**) olive oil 1°, and (**D**) olive oil 0.4°. KN_C1: Koroneiki_Control 1; KN_S: Koroneiki_Supplemented; KN_C2: Koroneiki_Control 2; KN_1: Koroneiki_Exp 1; KN_2: Koroneiki_Exp 2; KN_3: Koroneiki_Exp 3; KN_4: Koroneiki_Exp 4; KN_5: Koroneiki_Exp 5; KN_6: Koroneiki_Exp 6; KN_7: Koroneiki_Exp 7; KN_8: Koroneiki_Exp 8; AS_C1: Arbosana_Control 1; AS_S: Arbosana_Supplemented; AS_C2: Arbosana_Control 2; AS_1: Arbosana_Exp 1; AS_2: Arbosana_Exp 2; AS_3: Arbosana_Exp 3; AS_4: Arbosana_Exp 4; AS_5: Arbosana_Exp 5; AS_6: Arbosana_Exp 6; AS_7: Arbosana_Exp 7; AS_8: Arbosana_Exp 8; 1°O_C1: Olive 1°_Control 1; 1°O_S: Olive 1°_Supplemented; 1°O_C2: Olive 1°_Control 2; 1°O_1: Olive 1°_Exp 1; 1°O_2: Olive 1°_Exp 2; 1°O_3: Olive 1°_Exp 3; 1°O_4: Olive 1°_Exp 4; 1°O_5: Olive 1°_Exp 5; 1°O_6: Olive 1°_Exp 6; 1°O_7: Olive 1°_Exp 7; 1°O_8: Olive 1°_Exp 8; 0.4°O_C1: Olive 0.4°_Control 1; 0.4°O_S: Olive 0.4°_Supplemented; 0.4°O_C2: Olive 0.4°_Control 2; 0.4°O_1: Olive 0.4°_Exp 1; 0.4°O_2: Olive 0.4°_Exp 2; 0.4°O_3: Olive 0.4°_Exp 3; 0.4°O_4: Olive 0.4°_Exp 4; 0.4°O_5: Olive 0.4°_Exp 5; 0.4°O_6: Olive 0.4°_Exp 6; 0.4°O_7: Olive 0.4°_Exp 7; 0.4°O_8: Olive 0.4°_Exp 8.

Figure 4 shows the fatty acid dynamics (mg/kg) during deep-frying with HTyr supplementation in various olive oils: (A) EVOO Koroneiki, (B) EVOO Arbosana, (C) Olive oil 1°, and (D) Olive oil 0.4°. Collectively, these results confirm that HTyr supports oxidative protection across refined, virgin, and extra virgin olive oil grades, preserving fatty acid composition and enhancing thermal stability under frying stress, while ensuring broad compliance with European regulatory standards.

Overall, as reported, HTyr supplementation contributes to thermal oxidative stability, along with its well-known nutritional and health benefits. Moreover, the fatty acid profiles—particularly oleic acid—of most oil samples supplemented with HTyr during deep-frying remained within the specified limits. Indeed, polyphenols are natural secondary metabolites widely distributed in plants, known for their diverse biological activities, including antioxidant, anti-inflammatory, antimicrobial, cardioprotective, and anticancer effects. Due to these properties, polyphenols are increasingly utilized as active ingredients in nutraceuticals, food, pharmaceutical, and cosmetic formulations. Aligning with green chemistry principles and circular economy strategies, these valuable compounds can be sustainably recovered from agro-industrial waste, promoting resource efficiency and reducing environmental impact. The recent literature focusing on Mediterranean plants such as olive (*Olea europaea* L.) and pomegranate (*Punica granatum* L.) highlights the structural characteristics, extraction methods, biological properties, and applications of polyphenolic extracts, suggesting promising avenues for future research and industrial application [22].

Additionally, our study aligned with those reporting the positive role of natural extracts in improving the oxidative stability of edible oil. One study demonstrated the use of supercritical CO2 and ultrasound-assisted ethanol extraction (UAE) to obtain apolar and polar antioxidant fractions from *Citrus aurantium* flowers. High-resolution mass spectrometry (HPLC-HRMS) revealed distinct phytochemical profiles, with the polar extract exhibiting higher phenolic content and stronger antioxidant activity. When these extracts were co-encapsulated with linseed oil using the Particles from Gas-Saturated Solution (PGSS) technique, they significantly enhanced the oxidative stability of the oil. Key kinetic parameters such as induction time and oxidation rates confirmed that the polar extract had superior antioxidant efficiency, even surpassing the synthetic antioxidant BHT in performance. This finding underscores the potential of *Citrus aurantium* flower extracts as natural, sustainable antioxidants that can improve the shelf life and quality of oils in food applications [34]. Another relevant application of natural antioxidants is seen in the context of industrial transformer insulating fluids, where oxidative degradation is a major challenge. A recent investigation focused on developing sustainable insulating fluids by blending transesterified used cooking oil with corn oil, enhanced by the incorporation of tocopherol—a natural antioxidant known to improve oxidative stability. The study found that tocopherol significantly improved the electrical and physicochemical properties of these oil blends, effectively mitigating oxidative degradation and extending the lifespan of the insulating fluids. This research highlights the critical role of natural antioxidants in improving the performance and sustainability of industrial oils, aligning with demands for environmentally friendly alternatives [35]. Together, these studies illustrate the expanding role of plant-derived polyphenols and antioxidants in enhancing oxidative stability across various oil-based systems, from edible oils to industrial fluids. They emphasize sustainable extraction methods and practical applications that not only improve product quality and durability but also contribute to circular economy goals by valorizing agroindustrial by-products.

### 3.4. Fatty Acids of Sunflower Oil and High-Oleic-Acid Sunflower Oil

**Figure 5:**
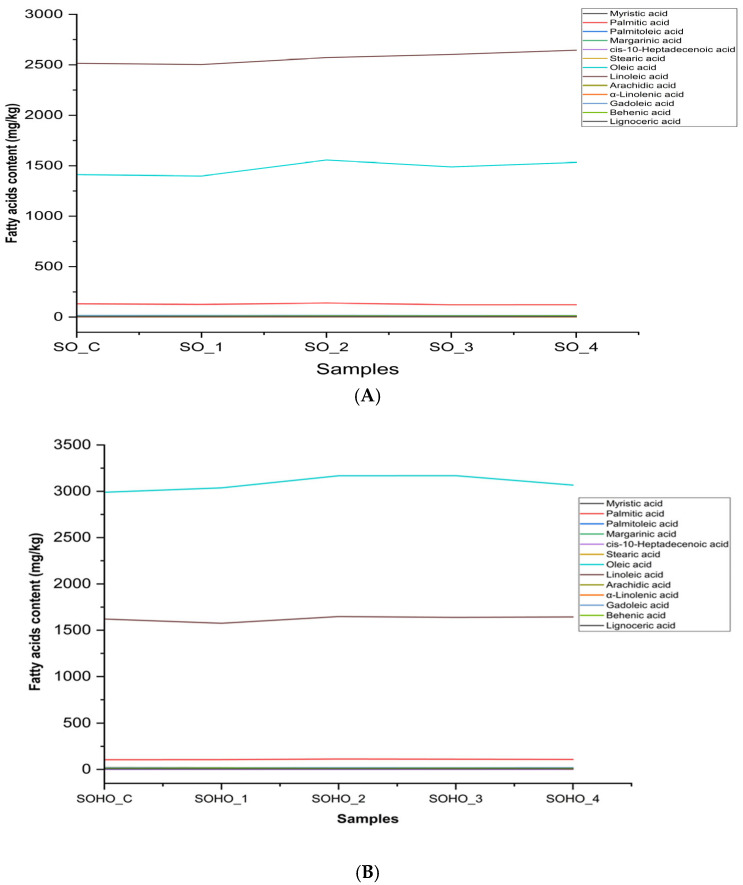
Fatty acid dynamics (mg/kg) during deep-frying at 170–210 °C for 3–6 h in various experiments using sunflower oils (**A**) and high-oleic-acid sunflower oils (**B**). Results are compared to the non-fried control samples (SO_C) and (SOHO_C). The figure illustrates the degradation or retention trends of major fatty acids under thermal conditions. SO_C: sunflower oil control; SO_1: sunflower oil deep-fried at 170 °C for 3 h; SO_2: sunflower oil deep-fried at 170 °C for 6 h; SO_3: sunflower oil deep-fried at 210 °C for 3 h; SO_4: sunflower oil deep-fried at 210 °C for 6 h; SOHO_C: high-oleic acid sunflower oil control; SOHO_1: high-oleic acid sunflower oil deep-fried at 170 °C for 3 h; SOHO_2: high-oleic acid sunflower oil deep-fried at 170°C for 6h; SOHO_3: high-oleic acid sunflower oil deep-fried at 210 °C for 3 h; SOHO_4: high-oleic acid sunflower oil deep-fried at 210 °C for 6 h.

The results illustrated in Table 4 and Table 5 present the fatty acid profiles of sunflower oil and high-oleic-acid sunflower oil (SOHO), respectively, under deep-frying conditions compared to their controls before thermal treatments. Also, Figure 5 presents the fatty acid dynamics (mg/kg) during deep-frying in sunflower oil (A) and high-oleic-acid sunflower oil (B). In regular sunflower oil (Table 4), the predominant fatty acids are linoleic acid (C18:2) at around 60–61% and oleic acid (C18:1) at around 34–36%, with saturated fatty acids (TSFAs) consistently low (~3.5%). The deep-frying treatments (varying times at 170 °C and 210 °C) cause minor fluctuations but the fatty acid contents remain largely stable. For high-oleic sunflower oil (Table 5), oleic acid dominates (~62–64%) while linoleic acid is lower (~33%), and total saturated fats remain below 3.2%. Deep-frying results in slight variations but the fatty acid composition remains stable, indicating good oxidative stability. Overall, both oils maintain their characteristic fatty acid profiles after frying, with high-oleic sunflower oil showing greater oleic acid content and marginally lower saturated fats, suggesting enhanced suitability for high-temperature cooking, as indicated previously [26]. High-oleic sunflower oil demonstrates significantly better thermal oxidative stability across all markers compared to regular sunflower oil under deep-frying conditions. It shows lower acidity, oxidation values (K_232_, K_270_, ∆K), and total oxidation (TOTOX), making it a more suitable option for high-temperature cooking. Moreover, a recent study evaluated the behavior of SOHO during the deep-frying of purple potatoes, simulating fast-food cooking processes. The results demonstrated that SOHO exhibited significantly better thermal stability than regular sunflower oil, with lower levels of oxidation markers such as peroxide value (PV), anisidine value (AnV), and TOTOX values. This indicates that SOHO maintains its quality and nutritional properties more effectively under high-temperature conditions [36]. Another study investigated the quality changes in SOHO during the frying of South American white shrimp (*Litopenaeus vannamei*). The findings revealed that SOHO had a higher resistance to oxidative degradation compared to regular sunflower oil, as evidenced by the lower increases in PV and AnV over time. This suggests that SOHO is more suitable for repeated frying applications, maintaining better oil quality and extending the usable life of the oil [37]. Finally, Figures S1–S142 show the total ion chromatograms (TICs) from a gas chromatography analysis of the fatty acids in each oil sample investigated in the study.

### 3.5. Principal Component Analysis of Changes in Fatty Acids in Edible Oils During Deep-Frying

**Figure 6:**
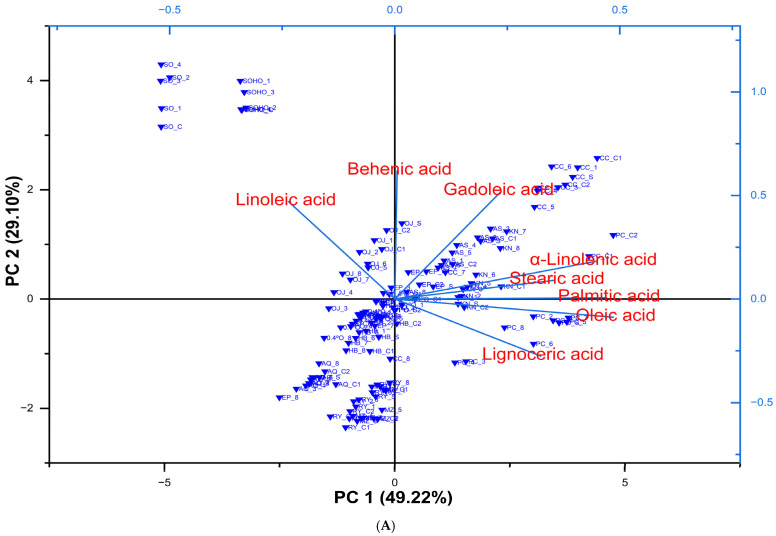

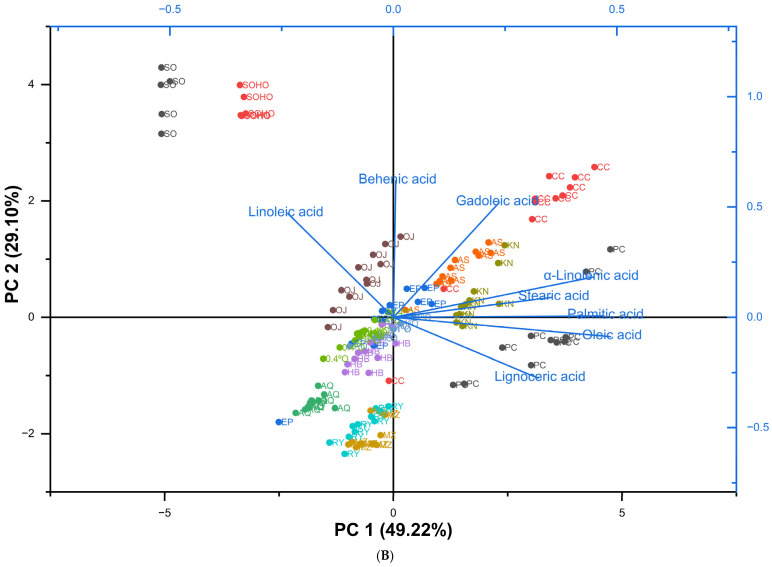

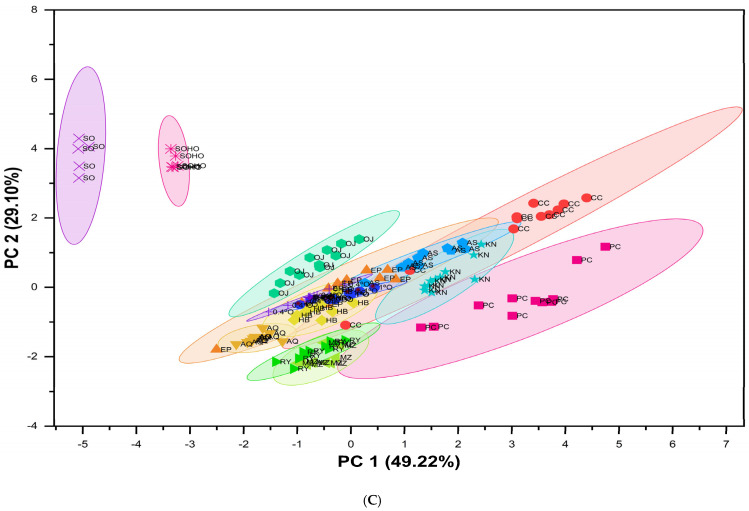
(**A**) Score and loading plots of various extra virgin olive oils (EVOOs), refined olive oils blended with EVOO or virgin olive oil (VOO) under HTyr supplementation, compared to sunflower oil and high-oleic sunflower oil. The plots illustrate the potential clustering of oil samples based on fatty acid changes (mg/kg) before and during deep-frying, as revealed by principal component analysis (PCA). (**B**) PCA clustering analysis based on different oil categories: the PCA clustering analysis categorizes different oils based on their fatty acids content (mg/kg), including myristic acid, palmitic acid, palmitoleic acid, margarinic acid, cis-10-heptadecenoic acid, stearic acid, oleic acid, linoleic acid, α-linolenic acid, arachidic acid, gadoleic acid, behenic acid, and lignoceric acid. (**C**) Cluster plot with confidence ellipse and PCA biplot showing the variables influencing the principal components of various categories of edible oils based on their fatty acid content. Refer to the captions of Table 6 for the abbreviations and meanings of the olive oil and sunflower oil samples.

The mean values of fatty acids (%) observed in the present study were within the limits established by the EU for the purity criteria of olive oils. Moreover, Table 6 shows the evolution and changes in fatty acid concentrations (mg/kg) in different cultivars and categories of olive oils as affected by hydroxytyrosol supplementation and deep-frying, compared to fired and non-fried sunflower oil and high-oleic sunflower oil. Data are presented as the mean of three replicates. The mean ± SD for each oil type is provided in the Supplementary Data (Tables S13–S26). Our findings confirm that chemometric analysis, e.g., PCA applied to fatty acid composition, is effective for distinguishing EVOOs by cultivar, as also demonstrated in previous research. Figure 6 demonstrates how HTyr supplementation influences the clustering of different edible oils based on their fatty acid profiles before and during deep-frying, highlighting key fatty acids that differentiate oil categories and their stability under thermal stress.

The clustering patterns shown in Figure 6 illustrate the distinct fatty acid compositions (mg/kg) characteristic of different oil types. Sunflower oils cluster prominently around linoleic acid, reflecting their high content of polyunsaturated fatty acids. In contrast, cultivars such as Picual, Empeltre, Manzanilla, Hojiblanca, and Royuella group closely with lignoceric acid, indicating a unique fatty acid profile likely influenced by their varietal traits and harvesting stages. As anticipated, most olive oils cluster near oleic acid and palmitic acid, the predominant fatty acids that contribute to their oxidative stability and nutritional benefits. These clear clustering trends demonstrate the effectiveness of fatty acid profiling combined with PCA in distinguishing oil types and evaluating their quality and stability during deep-frying.

Youssef et al. [38] found significant variation in palmitic, linoleic, and oleic acid content in Questati olive oils from seven Tunisian regions, attributing these differences to interactions between cultivar and environmental conditions. Similarly, Stefanoudaki et al. [39] studied the Koroneiki and Mastoides cultivars from Crete and suggested that altitude and rainfall may also contribute to compositional differences. The classification of Turkish olive oils based on cultivar, region, and harvest year was achieved through fatty acid profiling by Diraman et al. [40], highlighting the role of environmental factors. The use of non-parametric discriminant analysis for classifying virgin olive oils by origin based on fatty acid composition was further supported by Tsimidou and Karakostas [41]. Finally, variations in fatty acid profiles were shown to be primarily influenced by cultivar, followed by climatic and geographical factors, as reported by Kosma et al. [42]. Together with these studies, our results emphasize the strong discriminative power of fatty acid profiling in identifying olive oil origin and cultivar and underline the influence of agro-environmental practices. Moreover, under deep-frying conditions, the supplementation of hydroxytyrosol plays a significant role in preserving the fatty acid composition by protecting monounsaturated and polyunsaturated fatty acids from thermal degradation. This antioxidant effect enhances the oxidative stability of oils from different cultivars, helping maintain their unique fatty acid profiles despite the stresses of high-temperature frying. Thus, hydroxytyrosol supplementation not only supports oil quality but also complements the natural compositional differences shaped by cultivar and environmental factors.

## 4. Conclusions

Hydroxytyrosol (HTyr) supplementation significantly improves the thermal oxidative stability of olive oils, particularly extra virgin olive oils (EVOOs) from various cultivars and categories during prolonged deep-frying. HTyr helps preserve monounsaturated fatty acids, such as oleic acid, reduces the degradation of polyunsaturated and saturated fats, and maintains fatty acid profiles within regulatory limits. The protective effect varies by cultivar, with Empeltre showing the strongest response. Overall, fatty acid stability was observed in various olive oils during deep-frying, especially in Arbequina, Royuela, and Manzanilla. Principal component analysis (PCA) highlights that fatty acid profiling, combined with HTyr supplementation, can effectively differentiate oil types and monitor quality under heat stress. Minor increases in certain saturated fatty acids are linked to varietal characteristics and harvesting stages, emphasizing the importance of cultivar-specific assessments. Future research should focus on elucidating HTyr’s antioxidant mechanisms during thermal processing, optimizing supplementation levels for industrial applications, and evaluating sensory and nutritional impacts under real cooking conditions. Investigating other natural polyphenols and sustainable extraction methods will support the development of eco-friendly antioxidant solutions. Additionally, exploring HTyr’s potential applications beyond the food industry could further expand its industrial relevance.

## Figures and Tables

**Table 2:** Fatty acids content (%) of supplemented and non-supplemented EVOO cv. Manzanilla with HTyr under deep-frying conditions compared to the standard limits.

Fatty Acid	Con 1	SOO	Con 2	Exp 1	Exp 2	Exp 3	Exp 4	Exp 5	Exp 6	Exp 7	Exp 8	Standard Limits
C14:0	ND	ND	ND	ND	ND	ND	ND	ND	ND	ND	ND	≤0.03
C16:0	10.95 ± 0.17 ^a^	9.60 ± 0.12 ^ab^	9.43 ± 0.21 ^ab^	10.58 ± 0.37 ^a^	10.58 ± 0.25	9.74 ± 0.16 ^ab^	9.72 ± 0.32 ^ab^	9.80 ± 0.09 ^ab^	9.14 ± 0.11 ^ab^	9.06 ± 0.08 ^bc^	9.41 ± 0.14 ^a^	7.00–20.00
C16:1	0.96 ± 0.09 ^a^	0.85 ± 0.10 ^a^	0.84 ± 0.07 ^a^	0.93 ± 0.08 ^a^	0.94 ± 0.11 ^a^	0.85 ± 0.07 ^a^	0.87 ± 0.06 ^a^	0.91 ± 0.05 ^a^	0.81 ± 0.07 ^a^	0.88 ± 0.02 ^a^	0.91 ± 0.03 ^a^	0.30–3.50
C17:0	ND	ND	ND	ND	ND	ND	ND	ND	ND	ND	ND	≤0.40
C17:1	0.04 ± 0.01 ^a^	0.03 ± 0.00 ^a^	0.03 ± 0.00 ^a^	0.05 ± 0.01 ^a^	0.04 ± 0.00 ^a^	0.03 ± 0.00 ^a^	0.03 ± 0.00 ^a^	0.04 ± 0.00 ^a^	0.03 ± 0.00 ^a^	0.04 ± 0.01 ^a^	0.05 ± 0.01 ^a^	≤0.60
C18:0	0.23 ± 0.05 ^a^	0.14 ± 0.07 ^bc^	0.14 ± 0.04 ^bc^	0.18 ± 0.07 ^b^	0.14 ± 0.08 ^bc^	0.14 ± 0.03 ^bc^	0.13 ± 0.07 ^bc^	0.13 ± 0.01 ^bc^	0.12 ± 0.05 ^c^	0.11 ± 0.03 ^c^	0.13 ± 0.02 ^bc^	0.50–5.00
C18:1	78.89 ± 0.18 ^ab^	81.36 ± 0.24 ^a^	81.19 ± 0.31 ^a^	79.25 ± 0.13 ^ab^	79.25 ± 0.19 ^ab^	80.43 ± 0.27 ^a^	80.41 ± 0.09 ^a^	79.88 ± 0.11 ^a^	80.89 ± 0.13 ^a^	80.73 ± 0.22 ^a^	80.70 ± 0.08 ^a^	55.00–85.00
C18:2	4.64 ± 0.14 ^a^	4.14 ± 0.19 ^ab^	4.37 ± 0.17 ^ab^	4.62 ± 0.06 ^a^	4.86 ± 0.12 ^a^	4.46 ± 0.08 ^a^	4.76 ± 0.09 ^a^	5.04 ± 0.13 ^a^	4.59 ± 0.06 ^a^	4.76 ± 0.10 ^a^	4.56 ± 0.04 ^a^	2.50–21.00
C20:0	0.52 ± 0.07 ^a^	0.61 ± 0.05 ^a^	0.53 ± 0.02 ^a^	0.53 ± 0.03 ^a^	0.51 ± 0.07 ^a^	0.48 ± 0.05 ^a^	0.45 ± 0.01 ^ab^	0.53 ± 0.01 ^a^	0.50 ± 0.05 ^a^	0.54 ± 0.07 ^a^	0.50 ± 0.03 ^a^	≤0.60
C18:3	0.21 ± 0.02 ^a^	0.18 ± 0.04 ^ab^	0.19 ± 0.03 ^ab^	0.21 ± 0.02 ^a^	0.24 ± 0.05 ^a^	0.20 ± 0.02 ^a^	0.20 ± 0.04 ^a^	0.21 ± 0.03 ^a^	0.20 ± 0.05 ^a^	0.20 ± 0.06 ^a^	0.20 ± 0.01 ^a^	≤1.00
C20:1	0.20 ± 0.04 ^a^	0.18 ± 0.02 ^a^	0.18 ± 0.03 ^a^	0.20 ± 0.05 ^a^	0.21 ± 0.03 ^a^	0.20 ± 0.07 ^a^	0.20 ± 0.08 ^a^	0.20 ± 0.06 ^a^	0.20 ± 0.03 ^a^	0.20 ± 0.04 ^a^	0.20 ± 0.07 ^a^	≤0.50
C22:0	0.04 ± 0.00 ^a^	ND	ND	ND	0.04 ± 0.01 ^a^	ND	ND	ND	ND	ND	ND	≤0.20
C24:0	3.31 ± 0.12 ^a^	2.92 ± 0.09 ^b^	3.10 ± 0.15 ^ab^	3.40 ± 0.06 ^a^	3.19 ± 0.11 ^ab^	3.46 ± 0.21 ^a^	3.24 ± 0.17 ^a^	3.26 ± 0.13 ^a^	3.53 ± 0.05 ^a^	3.48 ± 0.07 ^a^	3.34 ± 0.09 ^a^	≤0.20
TSFAs	15.06 ± 0.19 ^a^	13.26 ± 0.08 ^b^	13.19 ± 0.10 ^b^	14.73 ± 0.33 ^a^	14.47 ± 0.26 ^ab^	13.83 ± 0.18 ^b^	13.54 ± 0.23 ^b^	13.72 ± 0.12 ^b^	13.29 ± 0.09 ^b^	13.20 ± 0.15 ^b^	13.38 ± 0.26 ^b^	
TMFAs	80.09 ± 0.26 ^b^	82.42 ± 0.33 ^a^	82.25 ± 0.22 ^a^	80.44 ± 0.15 ^b^	80.44 ± 0.24 ^b^	81.51 ± 0.24 ^ab^	81.50 ± 0.07 ^ab^	81.03 ± 0.19 ^ab^	81.92 ± 0.08 ^ab^	81.84 ± 0.23 ^ab^	81.85 ± 0.08 ^ab^	
TPUFAs	4.85 ± 0.08 ^a^	4.32 ± 0.18 ^b^	4.56 ± 0.05 ^ab^	4.83 ± 0.16 ^a^	5.09 ± 0.06 ^a^	4.66 ± 0.13 ^ab^	4.96 ± 0.09 ^a^	5.25 ± 0.04 ^a^	4.79 ± 0.11 ^a^	4.96 ± 0.02 ^a^	4.77 ± 0.15 ^a^	

^a,b,c^ Data in the same row followed by different superscript letters differ significantly (*p* < 0.05). ND: not detected. Refer to the caption of Table 1 for the meanings of the abbreviations used for the oil samples and fatty acids.

**Table 3:** Fatty acids content (%) of supplemented and non-supplemented EVOO cv. Royuela with HTyr under deep-frying conditions compared to the standard limits.

Fatty Acid	Con 1	SOO	Con 2	Exp 1	Exp 2	Exp 3	Exp 4	Exp 5	Exp 6	Exp 7	Exp 8	Standard Limits
C14:0	0.06 ± 0.01 ^a^	ND	ND	ND	ND	ND	ND	ND	ND	ND	ND	≤0.03
C16:0	11.80 ± 0.14 ^d^	11.25 ± 0.08 ^d^	11.62 ± 0.09 ^d^	12.10 ± 0.10 ^c^	12.73 ± 0.05 ^b^	12.88 ± 0.04 ^b^	14.85 ± 0.02 ^a^	12.60 ± 0.13 ^a^	13.37 ± 0.07 ^ab^	13.52 ± 0.04 ^ab^	13.86 ± 0.09 ^a^	7.00–20.00
C16:1	0.78 ± 0.12 ^bc^	0.83 ± 0.04 ^b^	0.84 ± 0.11 ^b^	0.87 ± 0.03 ^b^	0.93 ± 0.06 ^a^	0.91 ± 0.08 ^a^	1.05 ± 0.15 ^a^	0.89 ± 0.08 ^ab^	0.94 ± 0.07 ^a^	0.95 ± 0.05 ^a^	0.97 ± 0.03 ^a^	0.30–3.50
C17:0	0.04 ± 0.00 ^b^	0.04 ± 0.01 ^b^	0.05 ± 0.01 ^b^	0.05 ± 0.00 ^b^	0.06 ± 0.01 ^ab^	0.06 ± 0.00 ^ab^	0.07 ± 0.01 ^a^	0.06 ± 0.01 ^ab^	0.07 ± 0.00 ^a^	0.08 ± 0.02 ^a^	0.08 ± 0.01 ^a^	≤0.40
C17:1	0.09 ± 0.01 ^c^	0.09 ± 0.02 ^c^	0.09 ± 0.00 ^c^	0.10 ± 0.01 ^c^	0.12 ± 0.02 ^b^	0.12 ± 0.02 ^b^	0.15 ± 0.03 ^a^	0.14 ± 0.02 ^ab^	0.16 ± 0.04 ^a^	0.17 ± 0.04 ^a^	0.18 ± 0.03 ^a^	≤0.60
C18:0	0.74 ± 0.08 ^a^	0.29 ± 0.04 ^b^	0.24 ± 0.05 ^c^	0.23 ± 0.02 ^c^	0.26 ± 0.06 ^bc^	0.25 ± 0.05 ^c^	0.28 ± 0.04 ^b^	0.33 ± 0.03 ^b^	0.28 ± 0.01 ^b^	0.28 ± 0.02 ^b^	0.25 ± 0.04 ^c^	0.50–5.00
C18:1	76.03 ± 0.24 ^a^	76.78 ± 0.19 ^a^	76.48 ± 0.22 ^a^	75.95 ± 0.17 ^a^	75.28 ± 0.26 ^a^	75.20 ± 0.19 ^a^	72.47 ± 0.25 ^c^	75.49 ± 0.11 ^a^	74.58 ± 0.16 ^ab^	74.08 ± 0.08 ^ab^	73.59 ± 0.21 ^b^	55.00–85.00
C18:2	9.72 ± 0.13 ^a^	9.89 ± 0.08 ^a^	9.89 ± 0.07 ^a^	9.87 ± 0.15 ^a^	9.70 ± 0.23 ^a^	9.68 ± 0.08 ^a^	10.09 ± 0.18 ^a^	9.28 ± 0.07 ^a^	9.64 ± 0.29 ^a^	9.65 ± 0.12 ^a^	9.80 ± 0.08 ^a^	2.50–21.00
C20:0	0.49 ± 0.05 ^b^	0.55 ± 0.04 ^b^	0.49 ± 0.08 ^b^	0.49 ± 0.02 ^b^	0.54 ± 0.11 ^b^	0.51 ± 0.07 ^b^	0.56 ± 0.09 ^b^	0.82 ± 0.04 ^a^	0.53 ± 0.02 ^b^	0.81 ± 0.01 ^a^	0.80 ± 0.04 ^a^	≤0.60
C18:3	0.13 ± 0.04 ^a^	0.15 ± 0.04 ^a^	0.17 ± 0.04 ^a^	0.18 ± 0.04 ^a^	0.21 ± 0.04 ^a^	0.21 ± 0.04 ^a^	0.26 ± 0.04 ^a^	0.21 ± 0.04 ^a^	0.23 ± 0.04 ^a^	0.25 ± 0.04 ^a^	0.26 ± 0.09 ^a^	≤1.00
C20:1	0.12 ± 0.02 ^c^	0.13 ± 0.03 ^bc^	0.14 ± 0.00 ^b^	0.15 ± 0.01 ^b^	0.17 ± 0.03 ^ab^	0.17 ± 0.02 ^ab^	0.21 ± 0.05 ^a^	0.18 ± 0.02 ^a^	0.19 ± 0.02 ^a^	0.20 ± 0.04 ^a^	0.21 ± 0.05 ^a^	≤0.50
C22:0	ND	ND	ND	ND	ND	ND	ND	ND	ND	ND	ND	≤0.20
C24:0	2.98 ± 0.19 ^a^	2.50 ± 0.09 ^c^	2.62 ± 0.31 ^b^	2.69 ± 0.21 ^b^	2.72 ± 0.18 ^b^	2.82 ± 0.09 ^ab^	3.02 ± 0.14 ^a^	2.82 ± 0.17 ^ab^	2.92 ± 0.08 ^a^	2.99 ± 0.05 ^a^	3.06 ± 0.04 ^a^	≤0.20
TSFAs	16.11 ± 0.25 ^ab^	14.64 ± 0.13 ^bc^	15.01 ± 0.07 ^b^	15.56 ± 0.31 ^b^	16.31 ± 0.09 ^ab^	16.52 ± 0.15 ^ab^	18.79 ± 0.08 ^a^	16.64 ± 0.03 ^ab^	17.17 ± 0.04 ^a^	17.68 ± 0.06 ^a^	18.05 ± 0.11 ^a^	
TMFAs	77.02 ± 0.18 ^a^	77.82 ± 0.08 ^a^	77.55 ± 0.10 ^a^	77.07 ± 0.22 ^a^	76.49 ± 0.17 ^a^	76.41 ± 0.22 ^a^	73.88 ± 0.09 ^bc^	76.69 ± 0.31 ^a^	75.88 ± 0.19 ^ab^	75.40 ± 0.29 ^ab^	74.95 ± 0.04 ^b^	
TPUFAs	9.85 ± 0.19 ^a^	10.04 ± 0.11 ^a^	10.06 ± 0.10 ^a^	10.06 ± 0.09 ^a^	9.91 ± 0.04 ^a^	9.89 ± 0.15 ^a^	10.35 ± 0.28 ^a^	9.49 ± 0.14 ^a^	9.88 ± 0.15 ^a^	9.90 ± 0.04 ^a^	10.06 ± 0.09 ^a^	

^a,b,c,d^ Data in the same row followed by different superscript letters differ significantly (*p* < 0.05). ND: not detected. Refer to the caption of Table 1 for the meanings of the abbreviations used for the oil samples and fatty acids.

**Table 4:** Fatty acids content (%) of sunflower oil under deep-frying conditions compared to control.

Fatty Acid	Control	Exp 1 *	Exp 2	Exp 3	Exp 4
C14:0	ND	ND	ND	ND	ND
C16:0	3.20	3.08	3.21	2.87	2.82
C16:1	0.09	0.09	0.10	0.09	0.10
C17:0	ND	ND	ND	ND	ND
C17:1	ND	ND	ND	ND	ND
C18:0	0.06	0.04	0.07	0.08	0.05
C18:1	34.41	34.33	36.04	34.91	35.22
C18:2	61.31	61.48	59.53	61.06	60.78
C20:0	0.14	0.14	0.16	0.15	0.15
C18:3	0.10	0.11	0.12	0.11	0.11
C20:1	0.40	0.40	0.40	0.37	0.36
C22:0	0.16	0.20	0.24	0.26	0.28
C24:0	0.13	0.13	0.13	0.12	0.12
TSFAs	3.69	3.59	3.80	3.47	3.43
TMFAs	34.90	34.82	36.54	35.37	35.68
TPUFAs	61.41	61.59	59.65	61.17	60.89

ND: not detected. Control refers to original, non-deep-fried oil. * Exp.1: oil deep-fried at 170 °C for 3 h; Exp.2: oil deep-fried at 170 °C for 6 h; Exp.3: oil deep-fried at 210 °C for 3 h; Exp.4: oil deep-fried at 210 °C for 6 h. Refer to the caption of Table 1 for the meanings of the abbreviations used for the fatty acids.

**Table 5:** Fatty acids content (%) of high-oleic-acid sunflower oil under deep-frying conditions compared to control.

Fatty Acid	Control	Exp 1	Exp 2	Exp 3	Exp 4
C14:0	ND	ND	ND	ND	ND
C16:0	2.19	2.21	2.26	2.23	2.21
C16:1	0.10	0.10	0.10	0.10	0.10
C17:0	ND	ND	ND	ND	ND
C17:1	ND	ND	ND	ND	ND
C18:0	0.45	0.42	0.30	0.38	0.08
C18:1	62.37	63.31	63.34	63.42	62.85
C18:2	33.81	32.85	32.96	32.80	33.70
C20:0	0.15	0.15	0.15	0.16	0.15
C18:3	0.13	0.13	0.13	0.13	0.13
C20:1	0.43	0.40	0.41	0.39	0.41
C22:0	0.23	0.31	0.22	0.27	0.23
C24:0	0.14	0.13	0.13	0.13	0.14
TSFAs	3.16	3.22	3.06	3.16	2.81
TMFAs	62.89	63.81	63.85	63.91	63.36
TPUFAs	33.94	32.97	33.09	32.93	33.83

ND: not detected. Refer to the caption of Table 1 for the meanings of the symbols used for fatty acids. Refer to the caption of Table 4 for the meanings of the abbreviations used for the sunflower oil experiments.

**Table 6:** Evolution and changes in fatty acid concentrations (mg/kg) in different cultivars and categories of olive oils as affected by hydroxytyrosol supplementation and deep-frying, compared to fried and non-fried sunflower oil and high-oleic sunflower oil. Data are presented as the mean of three replicates. The mean ± SD for each oil type is provided in the Supplementary Data.

Oil	Myristic Acid	Palmitic Acid	Palmitoleic Acid	Margarinic Acid	Cis-10-Heptadecenoic Acid	Stearic Acid	Oleic Acid	Linoleic Acid	Arachidic Acid	α-Linolenic Acid	Gadoleic Acid	Behenic Acid	Lignoceric Acid
PC_C1	89.8	1147.45	79.1	6.94	14.58	477.76	5880.25	703.54	52.14	26.98	19.73	4	188.49
PC_S	87.78	971.05	69.49	6.73	13.49	503.81	5626.34	610.76	51.52	23.66	17.54	0	199.63
PC_C2	102.64	1102.74	79.16	7.75	16.14	642.61	5655.41	682.61	58.45	27.8	20.07	6.42	232.53
PC_1	82.4	908.13	66.59	8.11	13.53	495.16	5674.13	543.83	49.04	24.49	17.59	0	181.32
PC_2	80.06	921.26	65.21	7.4	13.59	453.42	5392.51	591.31	50.41	24.4	16.92	0	160.19
PC_3	53.34	764.23	75.59	2.97	7.74	381.54	5143.98	552.07	31.59	13.8	13.33	0	163.25
PC_4	56.94	807.53	59.89	4.29	8.5	341.92	4737.56	497.72	30.29	15.71	12.45	0	147.62
PC_5	89.39	982.17	66.59	8.11	13.53	515.4	5774.13	570.21	49.04	24.49	17.59	0	192.65
PC_6	86.76	901.46	64.1	5.92	11.96	512.39	5519.65	562.48	44.6	20.41	15.08	0	189.84
PC_7	76.76	1028.53	73.53	7.12	13.78	441.19	5789.22	632.03	50.79	25.28	18.24	0	201.95
PC_8	61.4	902.09	65.53	6.21	12.89	349.19	4994.83	546.07	43.02	22.5	16.38	0	162.62
CC_C1	82.43	1342.97	129.62	5.34	9.11	391.64	6028.68	990.46	72.07	35.66	20.08	8.69	104.41
CC_S	77.3	1269.68	122.35	5.81	9.35	351.12	5621.53	922.29	58.62	34.29	18.78	8.49	114.66
CC_C2	81.65	1202	115.81	5.24	7.65	377.07	5643.82	874.63	59	33.34	18.37	8.12	106.36
CC_1	88.97	1254.18	121.57	5.14	9.18	384.2	5758.62	919.74	64.66	34.91	18.92	8.91	103.09
CC_2	67.29	1164.07	111.89	5.95	12.48	289.18	5297.91	839.02	52.48	32.79	17.61	8.41	95.2
CC_3	83.45	1186.87	113.63	4.87	8.88	363.74	5583.8	856.74	52.42	33.05	17.59	8.54	105.64
CC_4	69.81	1168.01	112.49	4.91	10.9	299.4	5307.09	828.15	54.25	32.42	17.34	8.38	95.58
CC_5	81.58	1148.43	102.03	5.85	11.65	365.49	5408.68	832.14	55.82	29.91	16.12	7.79	94.41
CC_6	69.57	1169.71	111.61	4.62	8.48	317.8	5401.94	837.24	52.96	33.79	18.32	10.16	105.71
CC_7	60.6	813.13	78.11	5.28	8.55	278.48	4516.58	600.95	37.48	23.43	12.31	5.68	82.97
CC_8	44.84	772.57	68.61	4.49	0	186.65	4189.05	563.99	25.34	15.01	10.01	0	74.85
EP_C1	42.66	828.26	61.23	8.35	15.91	286.86	4301.14	635.67	44.01	16.53	14.67	4.71	81.24
EP_S	39.39	813.83	62.18	4.43	11.19	283.71	4638.78	663.73	41.98	16.19	14.03	4.06	92.39
EP_C2	38.87	790.69	60.05	9.94	9.11	276.87	4258.22	598.5	40.88	15.93	14	4.32	86.11
EP_1	33.91	747.06	57.02	8.23	12.9	189.6	3994.85	558.7	38.03	16.33	16.62	4	85.39
EP_2	9.19	742.37	57.23	4.77	10.96	74.21	3953.86	571.81	38.12	16	13.85	3.99	77.93
EP_3	9.67	762.19	59.26	4.51	11.04	82.66	4088.25	593.23	37.58	16.35	14.24	4.13	77.9
EP_4	4.49	728.16	56.33	3.94	9.36	50.36	3983	570.17	33.51	15.33	13.32	3.66	72.64
EP_5	0	742.16	56.94	3.38	8.08	21.52	4280.36	651.07	39.02	15.36	13.65	3.32	82.86
EP_6	0	657.38	51.28	2.84	7.12	18.85	3889.28	576.28	35.21	13.21	11.67	2.83	72.54
EP_7	0	692.38	54.03	2.57	6.74	14.82	4554.13	648.73	36.14	13.6	11.72	2.78	82.15
EP_8	0	381.61	32.98	1.24	3.51	5.07	3354.12	476.55	22.85	7.06	6.46	0	58.81
AQ_C1	5.66	637.35	87.9	0	2.47	112.23	4029.32	760.63	41.35	14.33	4.07	0	47.23
AQ_S	5.72	598.27	69.71	0	2.26	71.16	4046.16	777.02	36.4	10.89	5.54	0	38.82
AQ_C2	6.5	616.45	74.78	0	2.55	70.33	4006.26	817.56	32.42	11.87	6.05	0	45.11
AQ_1	2.51	570.12	64.1	0	2.08	46.45	3780.39	711.95	28.61	10.05	5.05	0	41.17
AQ_2	2.15	591.1	65.87	0	2.21	33.94	3799.85	712.66	28.68	10.59	5.29	0	40.3
AQ_3	1.87	535.08	60.58	0	1.96	32.29	3675.57	667.93	32.93	9.59	4.85	0	36.51
AQ_4	3.88	579.41	64.58	0	2.12	62.05	3717.03	696.11	25.68	10.59	5.33	0	42.84
AQ_5	4.05	566.27	63.81	0	2.05	73.29	3773.33	699.14	31.45	10.77	5.55	0	36.54
AQ_6	2.84	595.34	65.76	0	2.19	73.21	3664.23	691.07	34.37	11.31	5.75	0	37.25
AQ_7	3.31	577.36	65.48	0	2.12	52.45	3792.7	717.09	28.66	11.28	5.81	0	38.31
AQ_8	4.11	576.29	74.9	0	2.29	57.47	3696.28	699.19	28.05	13.1	7.3	0	34.64
HB_C1	2.19	645.86	48.98	1.65	2.77	41.02	4432.1	485.59	35.52	15.08	9.1	2.46	75.18
HB_S	2.05	643.8	50.94	1.64	2.86	36.77	4466.92	494.37	31.99	16.53	10.47	2.77	78.49
HB_C2	2.43	686.14	54.63	1.86	3.41	39.93	4622.36	515.24	34.91	18.77	11.29	3.34	84.15
HB_1	1.69	611.59	48.55	1.68	3.12	19.86	4087.17	450.26	30.19	17.29	10.53	3.15	72.4
HB_2	0	621.2	51.94	1.71	3.25	18.04	4198.85	466.55	36.42	18.31	10.84	3.41	65.97
HB_3	0	655.29	52.66	2.89	3.58	14.56	4140.09	464.82	39.2	19.16	11.44	3.71	71.87
HB_4	0	649.62	50.79	4.49	4.12	11.87	3930.51	440.21	37.15	21.89	11.9	4.12	75.91
HB_5	0	608.48	50.42	2.86	3.14	13.51	3932.9	440.52	30.95	17.26	10.06	3.27	69.75
HB_6	0	597.09	49.02	2.25	2.87	8.61	3981.61	446.35	28.98	16.38	9.92	2.92	69.26
HB_7	0	558.86	46.9	1.86	2.95	9.85	3946.59	440.6	37.36	15.64	9.57	2.78	69
HB_8	0	543.9	44.22	0	4.02	7.57	4013.23	441.37	32.6	15.06	9.08	2.55	70.35
MZ_C1	0	588.24	51.71	0	2.1	12.59	4237.47	249.5	28.12	11.14	10.86	2.16	177.57
MZ_S	0	496.6	44.06	0	1.72	7.42	4210.87	214.23	31.34	9.21	9.07	0	150.87
MZ_C2	0	513.52	45.74	0	1.83	7.36	4422.9	238.29	29.01	10.17	9.93	0	168.71
MZ_1	0	571.49	50.39	0	2.65	9.56	4279.86	249.39	28.85	11.37	11.07	2.04	183.71
MZ_2	0	540.74	47.97	0	2	7.35	4048.79	248.13	25.83	12.05	10.51	2.07	163.15
MZ_3	0	488.24	42.68	0	1.67	7.12	4030.36	223.36	24.13	10.12	10.04	0	173.4
MZ_4	0	475.05	42.3	0	1.63	6.52	3931.22	232.62	21.8	9.93	9.58	0	158.38
MZ_5	0	534.45	49.65	0	1.96	7.1	4357.74	275.06	28.97	11.43	10.92	0	177.98
MZ_6	0	490.81	43.31	0	1.72	6.55	4344.56	246.55	27.08	10.55	10.48	0	189.44
MZ_7	0	456.76	44.32	0	1.78	5.57	4068.03	239.67	27.19	10.17	9.99	0	175.48
MZ_8	0	451.77	43.71	0	2.2	6.23	3875.19	219.2	24.09	9.76	9.4	0	160.38
RY_C1	3.04	621.12	41.28	2.09	4.49	39.14	4001.53	511.49	25.67	6.93	6.14	0	156.81
RY_S	0	568.16	41.72	2.03	4.42	14.85	3878.65	499.61	28.04	7.77	6.41	0	126.38
RY_C2	0	622.06	45.15	2.51	5.04	12.73	4094.66	529.34	26	9.06	7.39	0	140.07
RY_1	0	646.74	46.68	2.46	5.54	12.27	4059.09	527.69	26.45	9.74	8.04	0	143.79
RY_2	0	648.95	47.33	3.22	6.33	13.21	3838.34	494.81	27.64	10.71	8.47	0	138.5
RY_3	0	660.92	46.85	3.05	6.36	12.99	3859.74	496.91	26.12	10.94	8.92	0	144.9
RY_4	0	759.8	53.63	3.83	7.86	14.36	3707.65	516.31	28.62	13.18	10.68	0	154.6
RY_5	0	689.46	48.47	3.47	7.57	17.97	4129.94	507.43	45.08	11.72	9.66	0	154.22
RY_6	0	699.5	49.32	3.72	8.43	14.47	3903.22	504.56	27.99	12.29	9.95	0	153.07
RY_7	0	715.05	50.29	4.16	9.18	15.06	3917.34	510.35	42.91	13.2	10.74	0	157.97
RY_8	0	749.2	52.5	4.36	9.53	13.4	3976.99	529.72	43.23	13.94	11.33	0	165.11
OJ_C1	2	658.43	50.87	3.34	6.44	17.21	4027.62	590.97	45.79	21.6	14.94	5.98	43.67
OJ_S	1.91	729.42	56.32	4.51	7.28	16.22	4288.76	643.2	44.35	22.36	16.81	7.1	47.63
OJ_C2	1.62	688.58	55.71	3.72	6.69	13.51	4069.96	616.48	39.98	21.39	16.12	6.91	41.59
OJ_1	1.89	617.58	49.22	3.59	6.8	13.27	3975.11	607.49	39.45	20.47	15.71	6.33	41.63
OJ_2	1.46	611.45	48.44	3.16	6.09	13.7	3709.25	552	35.57	19.03	14.62	6.14	36.44
OJ_3	0	525.7	41.45	2.08	4.28	11.76	3534.52	522.2	29.11	15.76	10.92	3.75	39.11
OJ_4	1.43	566.09	43.02	3.03	6.3	13.83	3490.57	497.63	30.17	15.83	12.02	4.55	34.79
OJ_5	2.28	628.13	46.72	3.11	5.53	21.51	3998.37	584.67	36.04	19.17	13.78	5.26	41.68
OJ_6	1.92	651.34	49.46	3.53	6.01	20.31	3913.28	601.69	37.59	19.09	13.99	5.34	41.29
OJ_7	3.26	578.18	44.08	3.11	5.3	22.85	3543.45	510.46	30.65	18.92	12.7	5.04	42.79
OJ_8	1.68	578.37	44.31	3.42	5.86	13.13	3454.47	495.47	31.02	17.38	13.19	5.42	36.86
KN_C1	55.86	819.6	55.82	2.48	3.65	416.88	5118.27	381.04	39.09	22.05	15.65	4.67	133.82
KN_S	51.16	773.06	52.56	2.34	4.01	362.28	4728.14	350.97	33.84	18.22	13.73	4.28	109.09
KN_C2	57.12	744.21	51.28	2.29	5.81	402.28	4832.42	339.35	33.01	18	13.56	4.32	117.57
KN_1	8.18	834.44	58.79	3.17	4.61	61.59	4991.08	369.86	34.87	20.94	15.58	5.07	137.72
KN_2	8.61	833.24	58.78	3.13	4.48	65.41	5020.98	376.57	36.46	21.09	15.68	5.22	143.57
KN_3	6.96	842.79	59.14	3.14	4.51	40.94	5037.81	375.43	39.47	21.75	16.32	5.39	141.07
KN_4	7.48	854.86	60.95	4.03	5.27	63.57	4960.98	372.14	35.31	21.95	16.34	5.71	149.08
KN_5	9.12	855.51	60.41	3.79	5.69	56.91	5029.19	385.57	42.42	22.54	16.56	5.88	151.59
KN_6	10.23	881.04	59.76	3.32	4.8	59.01	5130.7	371.37	40.33	22.85	17.12	6.08	139.36
KN_7	10.28	948.29	72.15	4.58	6.69	67.53	5065.38	444.74	43.32	27.56	20.23	7.57	157.85
KN_8	10	974.05	68.48	4.35	7.21	72.2	5103.3	376.98	40.66	25.28	19.1	7.22	151.19
AS_C1	10.03	1041.66	87.29	7.82	15.84	58.75	5339.56	458.66	42.79	24.66	18.27	7.34	107.26
AS_S	7.55	1010.43	84.81	8.1	14.46	45.47	5125.76	443.79	41.62	24.33	17.91	7.33	104.79
AS_C2	6.54	950.96	79	6.69	14.56	39.93	4863.99	412.28	38.39	21.57	16.08	6.47	94.53
AS_1	6.44	904.01	75.66	7.16	13.36	44.58	4682.25	396.6	36.41	21.74	16.08	6.66	90.49
AS_2	6.13	1015.62	86.17	7.61	14.97	37.52	5106.21	432.35	43.42	24.33	17.97	7.47	95.58
AS_3	2.74	1062.73	90.07	8.31	14.98	29.37	5116.66	459.37	40.25	25.54	18.97	7.73	109.68
AS_4	2.68	976.7	83.44	6.94	15.73	27.37	4493.7	423.24	39.86	23.5	17.62	7.11	106.53
AS_5	5.82	931.48	79.17	6.64	13.96	39.92	4834.56	415.57	36.77	22.23	16.6	7	89.71
AS_6	5.33	905.52	76.23	6.7	13.15	31.51	4761.25	389.12	34.52	21.2	15.85	6.44	85.36
AS_7	3.57	899.64	76.28	6.57	12.84	27.13	4648.61	396.3	34	20.96	15.84	6.29	90.62
AS_8	2.87	804.42	67.5	5.3	12.05	23.08	4342.46	353.79	28.99	18.26	13.88	5.47	82.2
1°O_C1	43.13	584.83	39.92	3.07	7.37	350.38	4279.46	407.82	38.81	17.92	12.4	4.4	79.89
1°O_S	23.12	551.56	37.63	2.83	9	290.31	4196.02	398.78	37.16	16.8	11.6	3.94	68.99
1°O_C2	24.93	553.77	38.4	2.98	4.43	303.52	4217.66	394.68	36.44	16.67	11.38	3.92	62.58
1°O_1	20.48	566.65	39.14	2.9	4.6	277.65	4284.13	400.34	35.86	17.49	12.19	4.15	73.57
1°O_2	14.31	565.35	39.49	2.64	4.1	219.35	4286	405.52	35.46	17.43	11.98	4.02	67.17
1°O_3	18.33	505.66	35.23	2.56	4.05	122.76	3887.86	356.74	31.72	16.1	11.36	3.86	61.6
1°O_4	9.06	483.89	34.1	2.38	3.79	147.75	3680.83	337.18	29.39	15.19	10.48	3.57	58.35
1°O_5	8.17	525.52	37.08	2.74	4.24	157.08	4212.27	392.14	35.33	16.76	11.73	3.72	76.56
1°O_6	5.51	520.3	36.63	2.65	4.11	136.51	4175.14	385.85	34.91	16.67	11.7	3.69	72.45
1°O_7	6.93	541.04	38.8	2.56	3.99	137.91	4209.47	400.37	33.97	17.02	11.83	3.64	70.31
1°O_8	7.19	543.94	38.76	2.47	3.99	142.32	4274.88	399.18	31.83	16.76	11.72	3.6	64.12
0.4°O_C1	8.77	671.02	47.84	3.49	4.96	136.67	4212.31	542.6	33.53	19.63	12.25	3.71	46.32
0.4°O_S	7.14	579.33	38.59	3.94	6.16	126.48	3809.32	413.01	29.44	16.61	11.09	3.24	39.77
0.4°O_C2	6.51	596.89	41.23	3.56	5.38	120.05	3941.82	458.81	30.64	17.24	11.32	3.29	43.61
0.4°O_1	6.19	575.77	39.07	3.7	5.88	121.52	3973.73	431.48	30.2	16.25	11.05	3.09	36.4
0.4°O_2	6.69	600.36	40.1	4.17	6.18	114.24	3837.32	414.36	28.05	16.36	11.06	3.29	35.63
0.4°O_3	2.62	577.64	40.53	3.22	5.05	100.17	4000.67	457.54	27.53	16.73	11.21	3.1	36.49
0.4°O_4	4.09	587.26	41.18	3.46	5.37	84.76	4049.42	447.63	25.9	17.21	11.46	3.18	38.72
0.4°O_5	6.25	618.94	42.86	3.67	5.91	118.63	4084.75	483.28	31.28	18.27	12.45	3.21	41.11
0.4°O_6	5.21	544	37.86	3.27	5.45	115.45	4015.2	432.43	28.58	15.87	10.92	2.86	36.64
0.4°O_7	3.88	516.36	37.44	2.8	4.39	84.64	3799.66	444.45	26.27	15.34	10.25	2.63	35.52
0.4°O_8	4.29	476.36	34.52	2.72	4.27	91.48	3535.01	407.06	22.27	13.86	9.23	2.49	33.3
SO_C	0	131.45	3.73	0	0	2.66	1411.84	2515.28	5.7	4.28	16.23	6.48	5.21
SO_1	0	125.41	3.7	0	0	1.44	1397.48	2502.72	5.85	4.42	16.41	8.25	5.32
SO_2	0	138.71	4.52	0	0	2.9	1556.4	2570.98	6.75	5.05	17.23	10.37	5.58
SO_3	0	122.25	3.68	0	0	3.21	1488.16	2602.61	6.22	4.71	15.7	10.93	5.2
SO_4	0	122.88	4.28	0	0	2.06	1532.73	2644.89	6.39	4.84	15.69	12.34	5.38
SOHO_C	0	104.94	4.67	0	0	21.74	2989.55	1620.68	7.35	6.28	20.37	10.9	6.61
SOHO_1	0	106.06	4.98	0	0	20.07	3037.42	1575.9	7.11	6.05	19.06	15.03	6.29
SOHO_2	0	112.86	5.08	0	0	15.1	3167.68	1648.14	7.54	6.37	20.3	11.13	6.56
SOHO_3	0	111.18	5.12	0	0	18.85	3168.68	1638.88	8.21	6.26	19.5	13.3	6.6
SOHO_4	0	108.05	5.06	0	0	3.8	3066.9	1644.34	7.29	6.37	19.96	11.22	6.8

PC_C1: Picual_Control 1; PC_S: Picual_Supplemented; PC_C2: Picual_Control 2; PC_1: Picual_Exp 1; PC_2: Picual_Exp 2; PC_3: Picual_Exp 3; PC_4: Picual_Exp 4; PC_5: Picual_Exp 5; PC_6: Picual_Exp 6; PC_7: Picual_Exp 7; PC_8: Picual_Exp 8; CC_C1: Cornicabra_Control 1; CC_S: Cornicabra_Supplemented; CC_C2: Cornicabra_Control 2; CC_1: Cornicabra_Exp 1; CC_2: Cornicabra_Exp 2; CC_3: Cornicabra_Exp 3; CC_4: Cornicabra_Exp 4; CC_5: Cornicabra_Exp 5; CC_6: Cornicabra_Exp 6; CC_7: Cornicabra_Exp 7; CC_8: Cornicabra_Exp 8; EP_C1: Empeltre_Control 1; EP_S: Empeltre_Supplemented; EP_C2: Empeltre_Control 2; EP_1: Empeltre_Exp 1; EP_2: Empeltre_Exp 2; EP_3: Empeltre_Exp 3; EP_4: Empeltre_Exp 4; EP_5: Empeltre_Exp 5; EP_6: Empeltre_Exp 6; EP_7: Empeltre_Exp 7; EP_8: Empeltre_Exp 8; AQ_C1: Arbequina_Control 1; AQ_S: Arbequina_Supplemented; AQ_C2: Arbequina_Control 2; AQ_1: Arbequina_Exp 1; AQ_2: Arbequina_Exp 2; AQ_3: Arbequina_Exp 3; AQ_4: Arbequina_Exp 4; AQ_5: Arbequina_Exp 5; AQ_6: Arbequina_Exp 6; AQ_7: Arbequina_Exp 7; AQ_8: Arbequina_Exp 8; HB_C1: Hojiblanca_Control 1; Hojiblanca_Supplemented; Hojiblanca_Control 2; HB_1: Hojiblanca_Exp 1; HB_2: Hojiblanca_Exp 2; HB_3: Hojiblanca_Exp 3; HB_4: Hojiblanca_Exp 4; HB_5: Hojiblanca_Exp 5; HB_6: Hojiblanca_Exp 6; HB_7: Hojiblanca_Exp 7; HB_8: Hojiblanca_Exp 8; MZ_C1: Manzanilla_Control 1; MZ_S: Manzanilla_Supplemented; MZ_C2: Manzanilla_Control 2; MZ_1: Manzanilla_Exp 1; MZ_2: Manzanilla_Exp 2; MZ_3: Manzanilla_Exp 3; MZ_4: Manzanilla_Exp 4; MZ_5: Manzanilla_Exp 5; MZ_6: Manzanilla_Exp 6; MZ_7: Manzanilla_Exp 7; MZ_8: Manzanilla_Exp 8; RY_C1: Royuela_Control 1; RY_S: Royuela_Supplemented; RY_C2: Royuela_Control 2; RY_1: Royuela_Exp 1; RY_2: Royuela_Exp 2; RY_3: Royuela_Exp 3; RY_4: Royuela_Exp 4; RY_5: Royuela_Exp 5; RY_6: Royuela_Exp 6; RY_7: Royuela_Exp 7; RY_8: Royuela_Exp 8; OJ_C1: Pomace_Control 1; OJ_S: Pomace_Supplemented; OJ_C2: Pomace_Control 2; OJ_1: Pomace_Exp 1; OJ_2: Pomace_Exp 2; OJ_3: Pomace_Exp 3; OJ_4: Pomace_Exp 4; OJ_5: Pomace_Exp 5; OJ_6: Pomace_Exp 6; OJ_7: Pomace_Exp 7; OJ_8: Pomace_Exp 8; KN_C1: Koroneiki_Control 1; KN_S: Koroneiki_Supplemented; KN_C2: Koroneiki_Control 2; KN_1: Koroneiki_Exp 1; KN_2: Koroneiki_Exp 2; KN_3: Koroneiki_Exp 3; KN_4: Koroneiki_Exp 4; KN_5: Koroneiki_Exp 5; KN_6: Koroneiki_Exp 6; KN_7: Koroneiki_Exp 7; KN_8: Koroneiki_Exp 8; AS_C1: Arbosana_Control 1; AS_S: Arbosana_Supplemented; AS_C2: Arbosana_Control 2; AS_1: Arbosana_Exp 1; AS_2: Arbosana_Exp 2; AS_3: Arbosana_Exp 3; AS_4: Arbosana_Exp 4; AS_5: Arbosana_Exp 5; AS_6: Arbosana_Exp 6; AS_7: Arbosana_Exp 7; AS_8: Arbosana_Exp 8; 1°O_C1: Olive 1°_Control 1; 1°O_S: Olive 1°_Supplemented; 1°O_C2: Olive 1°_Control 2; 1°O_1: Olive 1°_Exp 1; 1°O_2: Olive 1°_Exp 2; 1°O_3: Olive 1°_Exp 3; 1°O_4: Olive 1°_Exp 4; 1°O_5: Olive 1°_Exp 5; 1°O_6: Olive 1°_Exp 6; 1°O_7: Olive 1°_Exp 7; 1°O_8: Olive 1°_Exp 8; 0.4°O_C1: Olive 0.4°_Control 1; 0.4°O_S: Olive 0.4°_Supplemented; 0.4°O_C2: Olive 0.4°_Control 2; 0.4°O_1: Olive 0.4°_Exp 1; 0.4°O_2: Olive 0.4°_Exp 2; 0.4°O_3: Olive 0.4°_Exp 3; 0.4°O_4: Olive 0.4°_Exp 4; 0.4°O_5: Olive 0.4°_Exp 5; 0.4°O_6: Olive 0.4°_Exp 6; 0.4°O_7: Olive 0.4°_Exp 7; 0.4°O_8: Olive 0.4°_Exp 8. SO_C: sunflower oil control; SO_1: sunflower oil deep-fried at 170°C for 3h; SO_2: sunflower oil deep-fried at 170 °C for 6 h; SO_3: sunflower oil deep-fried at 210 °C for 3 h; SO_4: sunflower oil deep-fried at 210 °C for 6 h; SOHO_C: high-oleic acid sunflower oil control; SOHO_1: high-oleic acid sunflower oil deep-fried at 170 °C for 3 h; SOHO_2: high-oleic acid sunflower oil deep-fried at 170 °C for 6 h; SOHO_3: high-oleic acid sunflower oil deep-fried at 210 °C for 3 h; SOHO_4: high-oleic acid sunflower oil deep-fried at 210 °C for 6 h. Control 1 (used as the control for Experiments 1–4) refers to original, non-deep-fried olive oil. Supplemented oil refers to non-deep-fried olive oil enriched with olive fruit extract, which was also used in the preparation of Control 2. Control 2 (used as the control for Experiments 5–8) was a mixture of Control 1 and the supplemented oil, resulting in a total polyphenol content of up to 650 mg/kg. Exp.1 was olive oil deep-fried at 170 °C for 3 h without polyphenol supplementation; Exp.2 was olive oil deep-fried at 170 °C for 6 h without polyphenol supplementation; Exp.3 was olive oil deep-fried at 210 °C for 3 h without polyphenol supplementation; Exp.4 was olive oil deep-fried at 210 °C for 6 h without polyphenol supplementation; Exp.5 was olive oil deep-fried at 170 °C for 3 h with polyphenol supplementation; Exp.6 was olive oil deep-fried at 170 °C for 6 h with polyphenol supplementation; Exp.7 was olive oil deep-fried at 210 °C for 3 h with polyphenol supplementation; and Exp.8 was olive oil deep-fried at 210 °C for 6 h with polyphenol supplementation.

## Data Availability

Data will be available upon reasonable request.

## Supplementary Materials

The following supporting information can be downloaded at https://www.mdpi.com/article/10.3390/foods14122085/s1. Table S1. Experimental design (2^3^) methodology of olive oils supplemented with hydroxytyrosol under deep-frying stress. The experiment was conducted separately for each olive oil category, comprising nine EVOOs and three lower-quality olive oils (i.e., Orujo oil, olive oil 1°, and olive oil 0.4°). Each olive oil category includes three non-deep-fried samples: Control 1 (Con 1), supplemented olive oil (SOO), and Control 2 (Con 2). Table S2. Experimental design (2^2^) methodology for sunflower oil and high-oleic-acid sunflower oil under deep-frying stress*. Each sunflower oil category includes one non-deep-fried sample: the Control. Table S3. The total phenolic content (TPC) of various olive oils before thermal processing, serving as an independent variable in the experimental design (Table S1) for the deep-frying process. Tables S4–S12 show the fatty acids content (%) of different edible oils across HTyr supplementation and numerous deep-frying conditions. Tables S13–S26 show the fatty acids content (mg/kg) of different edible oils across HTyr supplementation and numerous deep-frying conditions. Figures S1–S142 show the total ion chromatograms (TICs) from the gas chromatography analysis of fatty acids in each oil sample investigated in the study.
