# Supplementary material for: Enhanced Thermal Resilience of Olive Oils: Fatty Acid Dynamics with Polyphenols Supplementation
Source: Foods. 2025 Jun 13;14(12):2085. doi: 10.3390/foods14122085 (PMC12192125; doi:10.3390/foods14122085)
Supplement: Supplementary file 1 [file foods-14-02085-s001.zip › foods-3703389-supplementary.pdf]

Supplementary materials for the article:

Enhanced Thermal Resilience of Olive Oils: Fatty Acid Dynamics with Polyphenols Supplementation

Taha Mehany, José M. González-Sáiz and Consuelo Pizarro *

Department of Chemistry, University of La Rioja, 26006 Logroño, Spain;
taha.abdellatif@unirioja.es (T.M.); josemaria.gonzalez@unirioja.es (J.M.G.-S.)
* Correspondence: consuelo.pizarro@unirioja.es; Tel.: +34-941299626

Table S1. Experimental design (2^3) methodology of olive oils supplemented with hydroxytyrosol under deep-frying stress. The experiment was conducted separately for each olive oil category, comprising nine EVOOs and three lower-quality olive oils (i.e., Orujo oil, Olive oil 1°, and Olive oil 0.4°). Each olive oil category includes three non-deep-fried samples: Control 1 (Con 1), Supplemented olive oil (SOO), and Control 2 (Con 2).*

Experiment*	Design Matrix			Independent Variables			Response
	X_1	X_2	X_3	Time (h)	Temp. (°C)	Polyphenols (mg/kg)	
1	-1	-1	-1	3	170	-	Fatty acids
2	+1	-1	-1	6	170	-	
3	-1	+1	-1	3	210	-	
4	+1	+1	-1	6	210	-	
5	-1	-1	+1	3	170	650	
6	+1	-1	+1	6	170	650	
7	-1	+1	+1	3	210	650	
8	+1	+1	+1	6	210	650	

Level	Time (h)	Temp. (°C)	Polyphenols (mg/kg)
-1	3	170	Original concentration (0 addition)
+1	6	210	650

*For the olive oil categories: **Con 1** (used as the control for Experiments 1–4) refers to original, non-deep-fried olive oil. **SOO** (Supplemented olive oil) refers to non-deep-fried olive oil that has been enriched with olive fruit extract, which is also used in the preparation of Con 2. **Con 2** (used as the control for Experiments 5–8) is a mixture of Con 1 and the supplemented oil, resulting in a total polyphenol content of up to 650 mg/kg. **Exp.1**: olive oil deep fried at 170 °C for 3 h without polyphenol supplementation, **Exp.2**: olive oil deep fried at 170 °C for 6 h without polyphenol supplementation, **Exp.3**: olive oil deep fried at 210 °C for 3 h without polyphenol supplementation, **Exp.4**: olive oil deep fried at 210 °C for 6 h without polyphenol supplementation, **Exp.5**: olive oil deep fried at 170 °C for 3 h with polyphenol supplementation, **Exp.6**: olive oil deep fried at 170 °C for 6 h with polyphenol supplementation, **Exp.7**: olive oil deep fried at 210 °C for 3 h with polyphenol supplementation, **Exp.8**: olive oil deep fried at 210 °C for 6 h with polyphenol supplementation.

Table S2. Experimental design (2^2) Methodology for sunflower oil and sunflower oil-high oleic acid under deep-frying stress*. Each sunflower oil category includes one non-deep-fried samples: Control.*

Experiment*	X_1	X_2	Independent Variables		Response
			Time (h)	Temp.(℃)	
1	-1	-1	3	170	Fatty acids
2	+1	-1	6	170	
3	-1	+1	3	210	
4	+1	+1	6	210	
Level	Time (h)	Temp. (℃)			
-1	3	170			
+1	6	210			

* For sunflower oil categories: **Control** refers to original, non-deep-fried oil. ****Exp.1**: oil deep fried at 170 °C for 3 h, **Exp.2**: oil deep fried at 170 °C for 6 h, **Exp.3**: oil deep fried at 210 °C for 3 h, **Exp.4**: oil deep fried at 210 °C for 6 h.

Table S3. The total phenolic content (TPC) of various olive oils before thermal processing, serving as an independent variable in the experimental design (Table S1) for the deep-frying process.

Olive oil type	Original olive oil	Supplemented olive oil	Supplemented olive oil and original olive oil mix
Picual	307.89±8.76	1524.33±2.43	658.60±4.46
Cornicabra	275.67±9.37	1683.03±32.09	658.15±17.44
Empeltre	337.92±11.58	1468.88 ± 47.30	647.23±5.26
Arbequina	227.71±6.32	1384.96±31.46	663.96±6.53
Hojiblanca	209.51±9.19	1208.67±41.67	655.73±13.09
Manzanilla	309.05±16.69	1255.28±28.17	661.28±7.78
Royuela	400.63±10.17	1211.16±30.83	662.38±10.84
Orujo	3.89±0.29	1259.06±20.40	652.25±5.55
Koroneiki	327.77±12.99	1230.89±18.22	663.93±3.15
Arbosana	393.00±6.59	1434.58±27.86	666.95±16.55
Olive oil 1°	181.88±7.21	1152.24±45.18	653.11±17.59
Olive oil 0.4°	26.50±2.40	1113.40±14.77	650.87±13.12

Table S4. Fatty acids content (%) of supplemented and non-supplemented EVOO cv. Cornicabra with HTyr under deep-frying conditions compared to the standard limits.

Fatty acid	Con 1	SOO	Con 2	Exp 1	Exp 2	Exp 3	Exp 4	Exp 5	Exp 6	Exp 7	Exp 8	Standard limits
C14:0	0.89±0.05^{a}	0.92±0.01^{a}	0.96±0.04^{a}	1.01±0.09^{a}	0.84±0.01^{ab}	0.99±0.02^{a}	0.87±0.05^{ab}	1.00±0.03^{a}	0.85±0.01^{ab}	0.93±0.04^{a}	0.75±0.06^{b}	≤0.03
C16:0	14.56±0.24^{a}	15.05±0.19^{a}	14.09±0.14^{a}	14.30±0.08^{a}	14.56±0.18^{a}	14.10±0.08^{a}	14.58±0.01^{a}	14.07±0.07^{a}	14.37±0.06^{a}	12.46±0.31^{b}	12.97±0.09^{b}	7.00-20.00
C16:1	1.41±0.05^{a}	1.45±0.07^{a}	1.36±0.01^{a}	1.39±0.14^{a}	1.40±0.06^{a}	1.35±0.03^{a}	1.40±0.21^{a}	1.25±0.03^{b}	1.37±0.01^{a}	1.20±0.02^{b}	1.15±0.04^{b}	0.30-3.50
C17:0	0.06±0.01^{a}	0.07±0.00^{a}	0.06±0.01^{a}	0.06±0.02^{a}	0.07±0.01^{a}	0.06±0.00^{a}	0.06±0.01^{a}	0.07±0.01^{a}	0.06±0.01^{a}	0.08±0.02^{a}	0.08±0.01^{a}	≤0.40
C17:1	0.10±0.02^{b}	0.11±0.01^{b}	0.09±0.00^{b}	0.10±0.01^{b}	0.16±0.03^{a}	0.11±0.01^{b}	0.14±0.04^{ab}	0.14±0.01^{a}	0.10±0.02^{b}	0.13±0.01^{a}	ND	≤0.60
C18:0	4.25±0.33^{a}	4.16±0.18^{a}	4.42±0.05^{a}	4.38±0.12^{a}	3.62±0.10^{b}	4.32±0.08^{a}	3.74±0.11^{b}	4.48±0.24^{a}	3.90±0.04^{ab}	4.27±0.07^{a}	3.13±0.03^{b}	0.50-5.00
C18:1	65.38±0.18^{b}	66.62±0.41^{b}	66.14±0.25^{b}	65.65±0.13^{b}	66.27±0.09^{b}	66.32±0.08^{b}	66.27±0.29^{b}	66.28±0.42^{b}	66.35±0.19^{b}	69.23±0.06^{a}	70.34±0.35^{a}	55.00-85.00
C18:2	10.74±0.04^{a}	10.93±0.05^{a}	10.25±0.01^{a}	10.48±0.10^{a}	10.50±0.01^{a}	10.18±0.06^{ab}	10.34±0.03^{a}	10.20±0.02^{a}	10.28±0.12^{a}	9.21±0.08^{b}	9.47±0.07^{b}	2.50-21.00
C20:0	0.78±0.07^{a}	0.69±0.03^{a}	0.69±0.01^{a}	0.74±0.02^{a}	0.66±0.05^{a}	0.62±0.01^{ab}	0.68±0.06^{a}	0.68±0.01^{a}	0.65±0.07^{a}	0.57±0.03^{b}	0.43±0.04^{c}	≤0.60
C18:3	0.39±0.01^{a}	0.41±0.02^{a}	0.39±0.02^{a}	0.40±0.01^{a}	0.41±0.03^{a}	0.39±0.02^{a}	0.40±0.04^{a}	0.37±0.03^{ab}	0.42±0.00^{a}	0.36±0.01^{ab}	0.25±0.02^{c}	≤1.00
C20:1	0.22±0.03^{a}	0.22±0.01^{a}	0.22±0.03^{a}	0.22±0.01^{a}	0.22±0.00^{a}	0.21±0.01^{a}	0.22±0.03^{a}	0.20±0.02^{a}	0.23±0.01^{a}	0.19±0.01^{a}	0.17±0.02^{ab}	≤0.50
C22:0	0.09±0.01^{a}	0.10±0.00^{a}	0.10±0.02^{a}	0.10±0.01^{a}	0.11±0.02^{a}	0.10±0.00^{a}	0.10±0.01^{a}	0.10±0.02^{a}	0.12±0.01^{a}	0.09±0.02^{a}	ND	≤0.20
C24:0	1.13±0.06^{b}	1.36±0.04^{a}	1.25±0.01^{a}	1.18±0.03^{b}	1.19±0.01^{b}	1.25±0.06^{a}	1.19±0.04^{b}	1.16±0.01^{b}	1.30±0.04^{a}	1.27±0.02^{a}	1.26±0.03^{a}	≤0.20
TSFAs	21.77±0.35^{a}	22.35±0.28^{a}	21.56±0.19^{a}	21.76±0.48^{a}	21.05±0.26^{a}	21.45±0.13^{a}	21.23±0.09^{a}	21.56±0.14^{a}	21.25±0.06^{a}	19.68±0.09^{b}	18.62±0.16^{b}	
TMFAs	67.10±0.78^{b}	68.41±0.52^{a}	67.80±0.85^{b}	67.35±0.22^{b}	68.05±0.14^{b}	67.99±0.09^{ab}	68.02±0.31^{b}	67.87±0.08^{b}	68.05±0.07^{b}	70.75±0.03^{a}	71.66±0.41^{a}	
TPUFAs	11.13±0.15^{a}	11.34±0.31^{a}	10.64±0.12^{a}	10.88±0.08^{a}	10.91±0.17^{a}	10.57±0.11^{a}	10.75±0.46^{a}	10.56±0.55^{a}	10.70±0.29^{a}	9.57±0.13^{ab}	9.72±0.17^{ab}	

^{a,b,} and ^{c} Data in the same row followed by different superscript letters differ significantly (*P*<0.05). ND: Not detected. Refer to the caption of Table 1 for the meanings of the abbreviations used for the oil samples and fatty acids.

Table S5. Fatty acids content (%) of supplemented and non-supplemented EVOO cv. Empeltre with HTyr under deep-frying conditions compared to the standard limits.

Fatty acid	Con 1	SOO	Con 2	Exp 1	Exp 2	Exp 3	Exp 4	Exp 5	Exp 6	Exp 7	Exp 8	Standard limits
C14:0	0.64±0.01^{a}	0.59±0.01^{a}	0.63±0.01^{a}	0.59±0.01^{a}	0.16±0.01^{b}	0.17±0.01^{b}	0.08±0.01^{c}	ND	ND	ND	ND	≤0.03
C16:0	12.34±0.01^{a}	12.17±0.01^{a}	12.75±0.01^{a}	13.00±0.01^{a}	13.32±0.01^{a}	13.23±0.01^{a}	13.13±0.01^{a}	12.54±0.01^{a}	12.31±0.01^{a}	11.31±0.01^{b}	8.77±0.01^{c}	7.00-20.00
C16:1	0.91±0.01^{ab}	0.93±0.01^{ab}	0.97±0.01^{a}	0.99±0.01^{a}	1.03±0.01^{a}	1.03±0.01^{a}	1.02±0.01^{a}	0.96±0.01^{a}	0.96±0.01^{a}	0.88±0.01^{ab}	0.76±0.01^{b}	0.30-3.50
C17:0	0.12±0.01^{a}	0.07±0.01^{b}	0.16±0.01^{a}	0.14±0.01^{a}	0.09±0.01^{b}	0.08±0.01^{b}	0.07±0.01^{b}	0.06±0.01^{bc}	0.05±0.01^{bc}	0.04±0.01^{c}	0.03±0.01^{c}	≤0.40
C17:1	0.24±0.01^{a}	0.17±0.01^{b}	0.15±0.01^{c}	0.22±0.01^{a}	0.20±0.01^{ab}	0.19±0.01^{b}	0.17±0.01^{b}	0.14±0.01^{c}	0.13±0.01^{c}	0.11±0.01^{d}	0.08±0.01^{d}	≤0.60
C18:0	4.27±0.01^{a}	4.24±0.01^{a}	4.46±0.01^{a}	3.30±0.01^{b}	1.33±0.01^{c}	1.43±0.01^{c}	0.91±0.01^{d}	0.36±0.01^{d}	0.35±0.01^{d}	0.24±0.01^{ef}	0.12±0.01^{f}	0.50-5.00
C18:1	64.06±0.01^{e}	69.38±0.01^{d}	68.64±0.01^{d}	69.49±0.01^{d}	70.93±0.01^{cd}	70.96±0.01^{cd}	71.84±0.01^{c}	72.33±0.01^{c}	72.85±0.01^{c}	74.42±0.01^{b}	77.10±0.01^{a}	55.00-85.00
C18:2	9.47±0.01^{b}	9.93±0.01^{a}	9.65±0.01^{ab}	9.72±0.01^{ab}	10.26±0.01^{a}	10.30±0.01^{a}	10.28±0.01^{a}	11.00±0.01^{a}	10.79±0.01^{a}	10.60±0.01^{a}	10.95±0.01^{a}	2.50-21.00
C20:0	0.66±0.01^{a}	0.63±0.01^{a}	0.66±0.01^{a}	0.66±0.01^{a}	0.68±0.01^{a}	0.65±0.01^{a}	0.60±0.01^{ab}	0.66±0.01^{a}	0.66±0.01^{a}	0.59±0.01^{a}	0.53±0.01^{b}	≤0.60
C18:3	0.25±0.01^{ab}	0.24±0.01^{ab}	0.26±0.01^{a}	0.28±0.01^{a}	0.29±0.01^{a}	0.28±0.01^{a}	0.28±0.01^{a}	0.26±0.01^{a}	0.25±0.01^{a}	0.22±0.01^{b}	0.16±0.01^{c}	≤1.00
C20:1	0.22±0.01^{bc}	0.21±0.01^{bc}	0.23±0.01^{b}	0.29±0.01^{a}	0.25±0.01^{b}	0.25±0.01^{b}	0.24±0.01^{b}	0.23±0.01^{b}	0.22±0.01^{bc}	0.19±0.01^{c}	0.15±0.01^{d}	≤0.50
C22:0	0.07±0.01^{a}	0.06±0.01^{a}	0.07±0.01^{a}	0.07±0.01^{a}	0.07±0.01^{a}	0.07±0.01^{a}	0.07±0.01^{a}	0.06±0.01^{a}	0.05±0.01^{a}	0.05±0.01^{a}	ND	≤0.20
C24:0	1.21±0.01^{c}	1.38±0.01^{a}	1.39±0.01^{a}	1.49±0.01^{a}	1.40±0.01^{a}	1.35±0.01^{ab}	1.31±0.01^{b}	1.40±0.01^{a}	1.36±0.01^{ab}	1.34±0.01^{b}	1.35±0.01^{ab}	≤0.20
TSFAs	19.30±0.01^{a}	19.14±0.01^{a}	20.11±0.01^{a}	19.24±0.01^{a}	17.05±0.01^{b}	16.99±0.01^{b}	16.17±0.01^{b}	15.08±0.01^{c}	14.79±0.01^{d}	13.58±0.01^{c}	10.79±0.01^{e}	
TMFAs	70.99±0.01^{c}	70.69±0.01^{b}	69.98±0.01^{b}	71.00±0.01^{b}	72.40±0.01^{ab}	72.43±0.01^{ab}	73.26±0.01^{ab}	73.66±0.01^{a}	74.17±0.01^{a}	75.60±0.01^{a}	78.09±0.01^{a}	
TPUFAs	9.71±0.01^{a}	10.17±0.01^{a}	9.90±0.01^{a}	10.00±0.01^{a}	10.54±0.01^{a}	10.58±0.01^{a}	10.56±0.01^{a}	11.26±0.01^{a}	11.04±0.01^{a}	10.82±0.01^{a}	11.12±0.01^{a}	

^{a,b,c,d,e,} and ^{f} Data in the same row followed by different superscript letters differ significantly (*P*<0.05). ND: Not detected. Refer to the caption of Table 1 for the meanings of the abbreviations used for the oil samples and fatty acids.

Table S6. Fatty acids content (%) of supplemented and non-supplemented EVOO cv. Arbequina with HTyr under deep-frying conditions compared to the standard limits.

Fatty acid	Con 1	SOO	Con 2	Exp 1	Exp 2	Exp 3	Exp 4	Exp 5	Exp 6	Exp 7	Exp 8	Standard limits
C14:0	0.10±0.01^{a}	0.10±0.00^{a}	0.11±0.02^{a}	0.05±0.01^{c}	0.04±0.00^{c}	0.04±0.00^{c}	0.07±0.01^{ab}	0.08±0.01^{a}	0.05±0.00^{c}	0.06±0.01^{b}	0.08±0.02^{a}	≤0.03
C16:0	11.10±0.08^{a}	10.57±0.23^{a}	10.83±0.17^{a}	10.83±0.06^{a}	11.17±0.11^{a}	10.58±0.08^{a}	11.12±0.07^{a}	10.76±0.12^{a}	11.49±0.04^{a}	10.90±0.10^{a}	11.10±0.03^{a}	7.00-20.00
C16:1	1.53±0.05^{a}	1.23±0.11^{bc}	1.31±0.06^{ab}	1.22±0.02^{c}	1.24±0.09^{bc}	1.20±0.14^{c}	1.24±0.03^{bc}	1.21±0.02^{c}	1.27±0.04^{bc}	1.24±0.08^{bc}	1.44±0.02^{a}	0.30-3.50
C17:0	ND	ND	ND	ND	ND	ND	ND	ND	ND	ND	ND	≤0.40
C17:1	0.04±0.00^{a}	0.04±0.01^{a}	0.04±0.00^{a}	0.04±0.00^{a}	0.04±0.01^{a}	0.04±0.00^{a}	0.04±0.00^{a}	0.04±0.00^{a}	0.04±0.01^{a}	0.04±0.01^{a}	0.04±0.01^{a}	≤0.60
C18:0	1.95±0.06^{a}	1.26±0.03^{c}	1.24±0.04^{c}	0.88±0.16^{d}	0.64±0.08^{e}	0.64±0.06^{e}	1.19±0.05^{c}	1.39±0.11^{b}	1.41±0.03^{b}	0.99±0.03^{cd}	1.11±0.05^{a}	0.50-5.00
C18:1	70.17±0.15^{b}	71.46±0.19^{a}	70.41±0.11^{a}	71.84±0.09^{a}	71.79±0.07^{a}	72.65±0.08^{a}	71.35±0.06^{a}	71.71±0.13^{a}	70.70±0.13^{a}	71.63±0.05^{a}	71.17±0.07^{a}	55.00-85.00
C18:2	13.25±0.08^{b}	13.72±0.06^{ab}	14.37±0.18^{a}	13.53±0.10^{b}	13.47±0.03^{b}	13.20±0.31^{b}	13.36±0.11^{b}	13.29±0.09^{b}	13.33±0.06^{b}	13.54±0.14^{b}	13.46±0.05^{b}	2.50-21.00
C20:0	0.72±0.15^{a}	0.64±0.09^{a}	0.57±0.08^{b}	0.54±0.04^{b}	0.54±0.02^{b}	0.65±0.01^{a}	0.49±0.01^{b}	0.60±0.05^{ab}	0.66±0.02^{a}	0.54±0.03^{b}	0.54±0.06^{b}	≤0.60
C18:3	0.25±0.05^{a}	0.19±0.04^{a}	0.21±0.01^{a}	0.19±0.02^{a}	0.20±0.04^{a}	0.19±0.03^{a}	0.20±0.01^{a}	0.20±0.03^{a}	0.22±0.07^{a}	0.21±0.03^{a}	0.25±0.01^{a}	≤1.00
C20:1	0.07±0.01^{b}	0.10±0.00^{a}	0.11±0.01^{a}	0.10±0.02^{a}	0.10±0.01^{a}	0.10±0.02^{a}	0.10±0.01^{a}	0.11±0.00^{a}	0.11±0.01^{ab}	0.11±0.03^{a}	0.14±0.01^{a}	≤0.50
C22:0	ND	ND	ND	ND	ND	ND	ND	ND	ND	ND	ND	≤0.20
C24:0	0.82±0.13^{a}	0.69±0.09^{b}	0.79±0.06^{a}	0.78±0.05^{a}	0.76±0.04^{a}	0.72±0.05^{ab}	0.82±0.03^{a}	0.69±0.01^{b}	0.72±0.02^{ab}	0.72±0.01^{ab}	0.67±0.03^{b}	≤0.20
TSFAs	14.69±0.44^{a}	13.25±0.36^{b}	13.55±0.19^{ab}	13.09±0.11^{b}	13.15±0.21^{b}	12.62±0.01^{bc}	13.70±0.09^{ab}	13.52±0.15^{b}	14.34±0.06^{a}	13.22±0.31^{b}	13.49±0.05^{ab}	
TMFAs	71.81±0.85^{ab}	72.83±0.41^{a}	71.88±0.93^{ab}	73.19±0.48^{a}	73.18±0.56^{a}	73.98±0.27^{a}	72.73±0.11^{a}	73.06±0.23^{a}	72.12±0.19^{a}	73.02±0.12^{a}	72.80±0.21^{a}	
**TPUFAs**	13.49±0.32^{a}	13.92±0.26^{a}	14.58±0.15^{a}	13.72±0.71^{a}	13.67±0.19^{a}	13.39±0.13^{a}	13.57±0.08^{a}	13.49±0.41^{a}	13.55±0.63^{a}	13.76±0.49^{a}	13.71±0.33^{a}	

^{a,b,c,} and ^{d} Data in the same row followed by different superscript letters differ significantly (*P*<0.05). ND: Not detected. Refer to the caption of Table 1 for the meanings of the abbreviations used for the oil samples and fatty acids.

Table S7. Fatty acids content (%) of supplemented and non-supplemented EVOO cv. Hojiblanca with HTyr under deep-frying conditions compared to the standard limits.

Fatty acid	Con 1	SOO	Con 2	Exp 1	Exp 2	Exp 3	Exp 4	Exp 5	Exp 6	Exp 7	Exp 8	Standard limits
C14:0	0.04±0.01^{a}	0.04±0.00^{a}	0.04±0.01^{a}	0.03±0.00^{a}	ND	ND	ND	ND	ND	ND	ND	≤0.03
C16:0	11.14±0.06^{a}	11.02±0.13^{a}	11.29±0.18^{a}	11.35±0.06^{a}	11.30±0.13^{a}	11.96±0.09^{a}	12.39±0.15^{a}	11.74±0.22^{a}	11.45±0.03^{a}	10.87±0.12^{a}	10.49±0.13^{ab}	7.00-20.00
C16:1	0.84±0.07^{ab}	0.87±0.03^{a}	0.90±0.11^{a}	0.90±0.09^{a}	0.94±0.17^{a}	0.96±0.08^{a}	0.97±0.11^{a}	0.97±0.07^{a}	0.94±0.04^{a}	0.91±0.22^{a}	0.85±0.16^{ab}	0.30-3.50
C17:0	0.03±0.00^{b}	0.03±0.01^{b}	0.03±0.00^{b}	0.03±0.00^{b}	0.03±0.00^{b}	0.05±0.01^{ab}	0.09±0.02^{a}	0.06±0.03^{ab}	0.04±0.01^{b}	0.04±0.00^{b}	ND	≤0.40
C17:1	0.05±0.01^{a}	0.05±0.00^{a}	0.06±0.01^{a}	0.06±0.01^{a}	0.06±0.00^{a}	0.07±0.02^{a}	0.08±0.01^{a}	0.06±0.02^{a}	0.06±0.00^{a}	0.06±0.03^{a}	0.08±0.03^{a}	≤0.60
C18:0	0.71±0.08^{a}	0.63±0.03^{a}	0.66±0.06^{a}	0.30±0.03^{b}	0.33±0.01^{b}	0.27±0.02^{bc}	0.23±0.03^{c}	0.26±0.00^{bc}	0.17±0.04^{d}	0.19±0.03^{d}	0.15±0.02^{d}	0.50-5.00
C18:1	76.45±0.85^{a}	76.49±0.34^{a}	76.04±0.41^{a}	76.48±0.29^{a}	76.39±0.43^{a}	75.56±0.52^{ab}	74.97±0.69^{ab}	75.88±0.53^{ab}	76.35±0.33^{a}	76.75±0.19^{a}	77.42±0.47^{a}	55.00-85.00
C18:2	8.38±0.37^{a}	8.47±0.41^{a}	8.48±0.22^{a}	8.38±0.18^{a}	8.49±0.36^{a}	8.48±0.18^{a}	8.40±0.58^{a}	8.50±0.27^{a}	8.56±0.41^{a}	8.57±0.67^{a}	8.51±0.52^{a}	2.50-21.00
C20:0	0.61±0.17^{ab}	0.55±0.15^{b}	0.57±0.09^{b}	0.56±0.03^{b}	0.66±0.18^{ab}	0.72±0.12^{a}	0.71±0.08^{a}	0.60±0.04^{b}	0.56±0.19^{b}	0.73±0.09^{a}	0.63±0.18^{ab}	≤0.60
C18:3	0.26±0.02^{bc}	0.28±0.05^{bc}	0.31±0.08^{b}	0.32±0.02^{b}	0.33±0.01^{ab}	0.35±0.06^{ab}	0.42±0.11^{a}	0.33±0.07^{ab}	0.31±0.05^{ab}	0.30±0.04^{ab}	0.29±0.03^{b}	≤1.00
C20:1	0.16±0.13^{a}	0.18±0.13^{a}	0.19±0.13^{a}	0.20±0.13^{a}	0.20±0.13^{a}	0.21±0.13^{a}	0.23±0.13^{a}	0.19±0.13^{a}	0.19±0.13^{a}	0.19±0.13^{a}	0.18±0.13^{a}	≤0.50
C22:0	0.04±0.00^{ab}	0.05±0.01^{ab}	0.06±0.01^{a}	0.06±0.00^{a}	0.06±0.01^{a}	0.07±0.02^{a}	0.08±0.01^{a}	0.06±0.00^{a}	0.06±0.00^{a}	0.05±0.01^{ab}	0.05±0.00^{ab}	≤0.20
C24:0	1.30±0.05^{b}	1.34±0.15^{ab}	1.38±0.09^{a}	1.33±0.14^{ab}	1.20±0.06^{c}	1.31±0.08^{ab}	1.45±0.14^{a}	1.35±0.03^{a}	1.33±0.07^{ab}	1.34±0.10^{a}	1.36±0.16^{a}	≤0.20
TSFAs	13.87±0.26^{a}	13.66±0.29^{a}	14.03±0.17^{a}	13.66±0.35^{a}	13.59±0.18^{a}	14.37±0.23^{a}	14.94±0.42^{a}	14.06±0.35^{a}	13.60±0.23^{a}	13.22±0.09^{ab}	12.67±0.19^{b}	
TMFAs	77.50±0.61^{a}	77.59±0.54^{a}	77.19±0.48^{a}	77.63±0.43^{a}	77.59±0.31^{a}	76.79±0.36^{ab}	76.25±0.18^{ab}	77.11±0.26^{a}	77.53±0.13^{a}	77.91±0.22^{a}	78.52±0.14^{a}	
TPUFAs	8.64±0.12^{ab}	8.75±0.09^{a}	8.79±0.13^{a}	8.70±0.20^{ab}	8.82±0.16^{a}	8.83±0.19^{a}	8.81±0.06^{a}	8.83±0.08^{a}	8.87±0.23^{a}	8.87±0.35^{a}	8.80±0.15^{a}	

^{a,b,} and ^{c} Data in the same row followed by different superscript letters differ significantly (*P*<0.05). ND: Not detected. Refer to the caption of Table 1 for the meanings of the abbreviations used for the oil samples and fatty acids.

Table S8. Fatty acids content (%) of supplemented and non-supplemented Orujo oil with HTyr under deep-frying conditions compared to the standard limits.

Fatty acid	Con 1	SOO	Con 2	Exp 1	Exp 2	Exp 3	Exp 4	Exp 5	Exp 6	Exp 7	Exp 8	Standard limits
C14:0	0.04±0.01^{ab}	0.03±0.00^{b}	0.03±0.00^{b}	0.04±0.00^{ab}	0.03±0.00^{b}	ND	0.03±0.00^{b}	0.04±0.01^{ab}	0.04±0.00^{ab}	0.07±0.01^{a}	0.04±0.01^{b}	≤0.03
C16:0	12.00±0.15^{a}	12.39±0.11^{a}	12.34±0.09^{a}	11.44±0.21^{a}	12.09±0.08^{a}	11.09±0.05^{ab}	12.00±0.06^{a}	11.62±0.12^{a}	12.14±0.14^{a}	11.99±0.08^{a}	12.30±0.05^{a}	7.00-20.00
C16:1	0.93±0.10^{a}	0.96±0.08^{a}	1.00±0.05^{a}	0.91±0.07^{a}	0.96±0.04^{a}	0.87±0.04^{a}	0.91±0.05^{a}	0.86±0.02^{a}	0.92±0.09^{a}	0.91±0.12^{a}	0.94±0.05^{a}	0.30-3.50
C17:0	0.06±0.01^{a}	0.08±0.02^{a}	0.07±0.01^{a}	0.07±0.00^{a}	0.06±0.01^{a}	0.04±0.00^{ab}	0.06±0.00^{a}	0.06±0.01^{a}	0.07±0.01^{a}	0.06±0.01^{a}	0.07±0.05^{a}	≤0.40
C17:1	0.12±0.06^{a}	0.12±0.05^{a}	0.12±0.04^{a}	0.13±0.01^{a}	0.12±0.02^{a}	0.09±0.03^{ab}	0.13±0.02^{a}	0.10±0.01^{a}	0.11±0.01^{a}	0.11±0.03^{a}	0.12±0.05^{a}	≤0.60
C18:0	0.31±0.07^{b}	0.28±0.06^{b}	0.24±0.06^{c}	0.25±0.05^{bc}	0.27±0.03^{bc}	0.25±0.05^{bc}	0.29±0.01^{b}	0.40±0.06^{a}	0.38±0.05^{a}	0.47±0.02^{a}	0.28±0.05^{b}	0.50-5.00
C18:1	73.38±0.22^{a}	72.87±0.31^{ab}	72.91±0.12^{ab}	73.63±0.29^{a}	73.34±0.31^{a}	74.56±0.12^{a}	73.96±0.17^{a}	73.99±0.12^{a}	72.95±0.08^{a}	73.50±0.19^{a}	73.49±0.22^{a}	55.00-85.00
C18:2	10.77±0.17^{a}	10.93±0.10^{a}	11.04±0.09^{a}	11.25±0.18^{a}	10.91±0.215^{a}	11.02±0.06^{a}	10.54±0.05^{a}	10.82±0.13^{a}	11.22±0.09^{a}	10.59±0.05^{a}	10.54±0.05^{a}	2.50-21.00
C20:0	0.83±0.08^{a}	0.75±0.11^{a}	0.72±0.20^{ab}	0.73±0.06^{ab}	0.70±0.01^{b}	0.61±0.11^{c}	0.64±0.08^{bc}	0.67±0.04^{bc}	0.70±0.05^{b}	0.64±0.09^{bc}	0.66±0.02^{b}	≤0.60
C18:3	0.39±0.02^{a}	0.38±0.08^{a}	0.38±0.04^{a}	0.38±0.01^{a}	0.38±0.04^{a}	0.33±0.05^{a}	0.34±0.09^{a}	0.35±0.01^{a}	0.36±0.08^{a}	0.39±0.06^{a}	0.37±0.09^{a}	≤1.00
C20:1	0.27±0.07^{b}	0.29±0.05^{a}	0.29±0.00^{a}	0.29±0.03^{a}	0.29±0.05^{a}	0.23±0.06^{b}	0.25±0.05^{ab}	0.26±0.08^{ab}	0.26±0.02^{ab}	0.26±0.04^{ab}	0.28±0.05^{a}	≤0.50
C22:0	0.11±0.02^{a}	0.12±0.03^{a}	0.12±0.01^{a}	0.12±0.04^{a}	0.12±0.01^{a}	0.08±0.00^{a}	0.10±0.01^{a}	0.10±0.02^{a}	0.10±0.01^{a}	0.10±0.00^{a}	0.12±0.01^{a}	≤0.20
C24:0	0.80±0.04^{ab}	0.81±0.05^{ab}	0.75±0.02^{b}	0.77±0.03^{b}	0.72±0.01^{bc}	0.82±0.05^{b}	0.74±0.00^{b}	0.77±0.05^{b}	0.77±0.03^{b}	0.89±0.03^{a}	0.78±0.05^{b}	≤0.20
TSFAs	14.15±0.05^{a}	14.46±0.05^{a}	14.26±0.05^{a}	13.41±0.05^{a}	14.00±0.05^{a}	12.90±0.05^{b}	13.86±0.05^{a}	13.66±0.05^{a}	14.19±0.05^{a}	14.23±0.05^{a}	14.25±0.10^{a}	
TMFAs	74.69±0.18^{a}	74.23±0.23^{a}	74.32±0.36^{a}	74.96±0.19^{a}	74.71±0.18^{a}	75.75±0.20^{a}	75.26±0.24^{a}	75.21±0.19^{a}	74.24±0.12^{a}	74.79±0.29^{a}	74.84±0.32^{a}	
TPUFAs	11.16±0.11^{a}	11.31±0.09^{a}	11.43±0.21^{a}	11.63±0.12^{a}	11.29±0.06^{a}	11.35±0.11^{a}	10.88±0.08^{a}	11.17±0.05^{a}	11.57±0.02^{a}	10.98±0.06^{a}	10.91±0.12^{a}	

^{a,b,} and ^{c} Data in the same row followed by different superscript letters differ significantly (*P*<0.05). ND: Not detected. Refer to the caption of Table 1 for the meanings of the abbreviations used for the oil samples and fatty acids.

Table S9. Fatty acids content (%) of supplemented and non-supplemented EVOO cv. Koroneiki with HTyr under deep-frying conditions compared to the standard limits.

Fatty acid	Con 1	SOO	Con 2	Exp 1	Exp 2	Exp 3	Exp 4	Exp 5	Exp 6	Exp 7	Exp 8	Standard limits
C14:0	0.79±0.04^{a}	0.79±0.02^{a}	0.86±0.03^{a}	0.12±0.01^{c}	0.13±0.02^{bc}	0.11±0.03^{c}	0.11±0.02^{c}	0.14±0.00^{b}	0.15±0.02^{b}	0.15±0.03^{b}	0.15±0.01^{b}	≤0.03
C16:0	11.59±0.22^{c}	11.89±0.13^{c}	11.24±0.09^{c}	12.75±0.17^{b}	12.64±0.08^{b}	12.78±0.24^{b}	13.04±0.36^{ab}	12.89±0.07^{ab}	13.06±0.06^{a}	13.79±0.14^{a}	14.20±0.09^{a}	7.00-20.00
C16:1	0.79±0.04^{bc}	0.81±0.05^{b}	0.77±0.02^{c}	0.90±0.04^{b}	0.89±0.01^{b}	0.90±0.03^{b}	0.93±0.04^{ab}	0.91±0.02^{b}	0.89±0.05^{b}	1.05±0.02^{a}	1.00±0.04^{a}	0.30-3.50
C17:0	0.04±0.00^{ab}	0.04±0.01^{ab}	0.03±0.00^{b}	0.05±0.00^{a}	0.05±0.01^{a}	0.05±0.00^{a}	0.06±0.01^{a}	0.06±0.00^{a}	0.05±0.00^{a}	0.07±0.01^{a}	0.06±0.00^{a}	≤0.40
C17:1	0.05±0.01^{bc}	0.06±0.00^{b}	0.09±0.01^{a}	0.07±0.02^{ab}	0.07±0.00^{b}	0.07±0.02^{ab}	0.08±0.01^{ab}	0.09±0.01^{a}	0.07±0.00^{ab}	0.10±0.02^{a}	0.11±0.01^{a}	≤0.60
C18:0	5.90±0.16^{a}	5.57±0.05^{ab}	6.08±0.12^{a}	0.94±0.17^{c}	0.99±0.09^{c}	0.62±0.16^{e}	0.97±0.21^{c}	0.86±0.08^{d}	0.87±0.15^{cd}	0.98±0.01^{c}	1.05±0.05^{c}	0.50-5.00
C18:1	72.41±0.29^{c}	72.70±0.31^{c}	72.98±0.13^{c}	76.25±0.36^{a}	76.15±0.11^{a}	76.39±0.09^{a}	75.65±0.14^{a}	75.79±0.23^{a}	76.06±0.17^{a}	73.67±0.26^{bc}	74.39±0.14^{b}	55.00-85.00
C18:2	5.39±0.15^{b}	5.40±0.06^{b}	5.13±0.19^{bc}	5.65±0.26^{b}	5.71±0.18^{b}	5.69±0.31^{b}	5.67±0.21^{b}	5.81±0.07^{ab}	5.51±0.09^{b}	6.47±0.14^{a}	5.50±0.08^{b}	2.50-21.00
C20:0	0.55±0.08^{a}	0.52±0.01^{ab}	0.50±0.06^{ab}	0.53±0.04^{a}	0.55±0.11^{a}	0.60±0.09^{a}	0.54±0.06^{a}	0.64±0.10^{a}	0.60±0.01^{a}	0.63±0.03^{a}	0.59±0.06^{a}	≤0.60
C18:3	0.31±0.04^{ab}	0.28±0.03^{bc}	0.27±0.00^{c}	0.32±0.03^{b}	0.32±0.01^{b}	0.33±0.02^{ab}	0.33±0.03^{ab}	0.34±0.01^{ab}	0.34±0.03^{ab}	0.40±0.02^{a}	0.37±0.01^{a}	≤1.00
C20:1	0.22±0.02^{a}	0.21±0.02^{a}	0.20±0.02^{a}	0.24±0.02^{a}	0.24±0.02^{a}	0.25±0.02^{a}	0.25±0.02^{a}	0.25±0.02^{a}	0.25±0.02^{a}	0.29±0.02^{a}	0.28±0.02^{a}	≤0.50
C22:0	0.07±0.03^{ab}	0.07±0.02^{ab}	0.07±0.01^{ab}	0.08±0.01^{a}	0.08±0.02^{a}	0.08±0.00^{a}	0.09±0.02^{a}	0.09±0.03^{a}	0.09±0.00^{a}	0.11±0.01^{a}	0.11±0.02^{a}	≤0.20
C24:0	1.89±0.14^{c}	1.68±0.16^{cd}	1.78±0.09^{c}	2.10±0.17^{b}	2.18±0.09^{b}	2.14±0.01^{b}	2.27±0.06^{a}	2.28±0.02^{a}	2.07±0.03^{a}	2.30±0.06^{a}	2.20±0.11^{ab}	≤0.20
TSFAs	20.83±0.25^{a}	20.54±0.42^{a}	20.55±0.13^{a}	16.58±0.21^{c}	16.62±0.31^{c}	16.37±0.09^{c}	17.08±0.18^{c}	16.96±0.13^{c}	16.89±0.06^{c}	18.02±0.23^{b}	18.36±0.30^{b}	
TMFAs	73.47±0.45^{c}	73.78±0.31^{bc}	74.05±0.19^{bc}	77.45±0.26^{a}	77.35±0.19^{a}	77.60±0.33^{a}	76.91±0.14^{a}	77.03±0.15^{a}	77.27±0.22^{a}	75.11±0.06^{b}	75.77±0.17^{b}	
TPUFAs	5.70±0.10^{b}	5.68±0.15^{b}	5.40±0.08^{b}	5.97±0.14^{ab}	6.03±0.17^{a}	6.02±0.08^{a}	6.01±0.06^{a}	6.15±0.22^{a}	5.84±0.09^{ab}	6.87±0.13^{a}	5.86±0.09^{ab}	

$^{a,b,\ c,\ d,}$ and ^{e} Data in the same row followed by different superscript letters differ significantly (*P*<0.05). ND: Not detected. Refer to the caption of Table 1 for the meanings of the abbreviations used for the oil samples and fatty acids.

Table S10. Fatty acids content (%) of supplemented and non-supplemented EVOO cv. Arbosana with HTyr under deep-frying conditions compared to the standard limits.

Fatty acid	Con 1	SOO	Con 2	Exp 1	Exp 2	Exp 3	Exp 4	Exp 5	Exp 6	Exp 7	Exp 8	Standard limits
C14:0	0.14±0.02^{a}	0.11±0.00^{b}	0.10±0.01^{b}	0.10±0.01^{b}	0.09±0.00^{b}	0.04±0.00^{c}	0.04±0.00^{c}	0.09±0.01^{b}	0.08±0.00^{b}	0.06±0.01^{bc}	0.05±0.16^{c}	≤0.03
C16:0	14.43±0.16^{a}	14.57±0.21^{a}	14.52±0.18^{a}	14.35±0.06^{a}	14.73±0.31^{a}	15.21±0.25^{a}	15.69±0.09^{a}	14.34±0.19^{a}	14.26±0.34^{a}	14.42±0.23^{a}	13.97±0.16^{ab}	7.00-20.00
C16:1	1.21±0.08^{a}	1.22±0.12^{a}	1.21±0.05^{a}	1.20±0.03^{a}	1.25±0.14^{a}	1.29±0.21^{a}	1.34±0.06^{a}	1.22±0.08^{a}	1.20±0.14^{a}	1.22±0.16^{a}	1.17±0.16^{a}	0.30-3.50
C17:0	0.11±0.02^{a}	0.12±0.02^{a}	0.10±0.00^{a}	0.11±0.01^{a}	0.11±0.01^{a}	0.12±0.00^{a}	0.11±0.01^{a}	0.10±0.01^{a}	0.11±0.00^{a}	0.11±0.01^{a}	0.09±0.16^{a}	≤0.40
C17:1	0.22±0.06^{a}	0.21±0.04^{a}	0.22±0.02^{a}	0.21±0.06^{a}	0.22±0.00^{a}	0.21±0.01^{a}	0.25±0.03^{a}	0.22±0.02^{a}	0.21±0.03^{a}	0.21±0.02^{a}	0.21±0.16^{a}	≤0.60
C18:0	0.81±0.05^{a}	0.66±0.02^{b}	0.61±0.06^{b}	0.71±0.02^{b}	0.54±0.02^{c}	0.42±0.03^{d}	0.44±0.01^{d}	0.61±0.16^{b}	0.50±0.04^{c}	0.43±0.04^{cd}	0.40±0.16^{d}	0.50-5.00
C18:1	73.96±0.29^{ab}	73.90±0.19^{ab}	74.25±0.21^{a}	74.30±0.16^{a}	74.05±0.11^{a}	73.24±0.07^{b}	72.19±0.18^{b}	74.45±0.26^{a}	74.98±0.09^{a}	74.51±0.14^{a}	75.39±0.16^{a}	55.00-85.00
C18:2	6.35±0.21^{a}	6.40±0.17^{a}	6.29±0.09^{a}	6.29±0.17^{a}	6.27±0.14^{a}	6.58±0.06^{a}	6.80±0.15^{a}	6.40±0.02^{a}	6.13±0.12^{a}	6.35±0.05^{a}	6.14±0.16^{a}	2.50-21.00
C20:0	0.59±0.02^{a}	0.60±0.01^{a}	0.59±0.02^{a}	0.58±0.03^{a}	0.63±0.02^{a}	0.58±0.01^{a}	0.64±0.04^{a}	0.57±0.01^{a}	0.54±0.03^{a}	0.54±0.01^{a}	0.50±0.16^{a}	≤0.60
C18:3	0.34±0.01^{a}	0.35±0.02^{a}	0.33±0.02^{a}	0.35±0.04^{a}	0.35±0.01^{a}	0.37±0.02^{a}	0.38±0.01^{a}	0.34±0.01^{a}	0.33±0.02^{a}	0.34±0.01^{a}	0.32±0.16^{a}	≤1.00
C20:1	0.25±0.03^{a}	0.26±0.05^{a}	0.25±0.01^{a}	0.26±0.02^{a}	0.26±0.01^{a}	0.27±0.03^{a}	0.28±0.06^{a}	0.26±0.02^{a}	0.25±0.04^{a}	0.25±0.02^{a}	0.24±0.16^{a}	≤0.50
C22:0	0.10±0.01^{a}	0.11±0.02^{a}	0.10±0.00^{a}	0.11±0.01^{a}	0.11±0.01^{a}	0.11±0.02^{a}	0.11±0.00^{a}	0.11±0.03^{a}	0.10±0.01^{a}	0.10±0.00^{a}	0.09±0.16^{a}	≤0.20
C24:0	1.49±0.19^{a}	1.51±0.11^{a}	1.44±0.06^{a}	1.44±0.15^{a}	1.39±0.09^{a}	1.57±0.04^{a}	1.71±0.10^{a}	1.38±0.18^{ab}	1.34±0.08^{ab}	1.45±0.03^{a}	1.43±0.16^{a}	≤0.20
TSFAs	17.67±0.36^{a}	17.66±0.45^{a}	17.46±0.33^{a}	17.39±0.25^{a}	17.60±0.19^{a}	18.05±0.23^{a}	18.75±0.09^{a}	17.21±0.24^{a}	16.93±0.31^{ab}	17.12±0.18^{a}	16.53±0.16^{ab}	
TMFAs	75.64±0.41^{b}	75.59±0.17^{b}	75.92±0.21^{b}	75.97±0.17^{b}	75.78±0.04^{b}	75.01±0.19^{b}	74.07±0.28^{b}	76.14±0.09^{a}	76.64±0.17^{a}	76.20±0.34^{a}	77.01±0.16^{a}	
TPUFAs	6.69±0.07^{a}	6.75±0.10^{a}	6.62±0.11^{a}	6.64±0.06^{a}	6.62±0.17^{a}	6.94±0.18^{a}	7.18±0.09^{a}	6.74±0.15^{a}	6.46±0.12^{ab}	6.69±0.07^{a}	6.46±0.16^{a}	

^{a,b,} and ^{c} Data in the same row followed by different superscript letters differ significantly (*P*<0.05). ND: Not detected. Refer to the caption of Table 1 for the meanings of the abbreviations used for the oil samples and fatty acids.

Table S11. Fatty acids content (%) of supplemented and non-supplemented olive oil 1° with HTyr under deep-frying conditions compared to the standard limits.

Fatty acid	Con 1	SOO	Con 2	Exp 1	Exp 2	Exp 3	Exp 4	Exp 5	Exp 6	Exp 7	Exp 8	Standard limits
C14:0	0.73±0.09^{a}	0.41±0.03^{b}	0.44±0.05^{b}	0.36±0.07^{c}	0.25±0.04^{d}	0.36±0.06^{c}	0.19±0.02^{e}	0.15±0.02^{f}	0.10±0.00^{g}	0.13±0.01^{fg}	0.13±0.02^{a}	≤0.03
C16:0	9.95±0.15^{a}	9.77±0.17^{a}	9.76±0.08^{a}	9.87±0.10^{a}	9.97±0.06^{a}	10.00±0.15^{a}	10.05±0.18^{a}	9.60±0.07^{a}	9.62±0.03^{a}	9.88±0.14^{a}	9.82±0.08^{a}	7.00-20.00
C16:1	0.68±0.02^{a}	0.67±0.08^{a}	0.68±0.04^{a}	0.68±0.07^{a}	0.70±0.08^{a}	0.70±0.05^{a}	0.71±0.01^{a}	0.68±0.06^{a}	0.68±0.07^{a}	0.71±0.08^{a}	0.70±0.02^{a}	0.30-3.50
C17:0	0.05±0.01^{a}	0.05±0.00^{a}	0.05±0.00^{a}	0.05±0.00^{a}	0.05±0.01^{a}	0.05±0.00^{a}	0.05±0.00^{a}	0.05±0.01^{a}	0.05±0.00^{a}	0.05±0.00^{a}	0.04±0.00^{a}	≤0.40
C17:1	0.13±0.02^{ab}	0.16±0.03^{a}	0.08±0.01^{c}	0.08±0.02^{c}	0.07±0.01^{c}	0.08±0.00^{c}	0.08±0.01^{c}	0.08±0.00^{c}	0.08±0.01^{c}	0.07±0.01^{c}	0.07±0.00^{c}	≤0.60
C18:0	5.96±0.17^{a}	5.14±0.11^{ab}	5.35±0.07^{a}	4.84±0.09^{b}	3.87±0.21^{c}	2.43±0.14^{e}	3.07±0.08^{cd}	2.87±0.02^{d}	2.53±0.07^{e}	2.52±0.05^{e}	2.57±0.03^{e}	0.50-5.00
C18:1	72.79±0.39^{c}	74.30±0.27^{b}	74.37±0.28^{b}	74.65±0.33^{b}	75.55±0.41^{b}	76.87±0.27^{a}	76.43±0.18^{a}	76.93±0.26^{a}	77.23±0.32^{a}	76.85±0.14^{a}	77.15±0.36^{a}	55.00-85.00
C18:2	6.94±0.12^{ab}	7.06±0.08^{a}	6.96±0.05^{ab}	6.98±0.04^{ab}	7.15±0.10^{a}	7.05±0.12^{a}	7.00±0.08^{a}	7.16±0.14^{a}	7.14±0.06^{a}	7.31±0.21^{a}	7.20±0.05^{a}	2.50-21.00
C20:0	0.66±0.01^{a}	0.66±0.01^{a}	0.64±0.07^{a}	0.62±0.08^{a}	0.63±0.03^{a}	0.63±0.06^{a}	0.61±0.07^{a}	0.65±0.01^{a}	0.65±0.04^{a}	0.62±0.07^{a}	0.57±0.02^{ab}	≤0.60
C18:3	0.30±0.00^{a}	0.30±0.03^{a}	0.29±0.02^{a}	0.30±0.01^{a}	0.31±0.00^{a}	0.32±0.02^{a}	0.32±0.03^{a}	0.31±0.04^{a}	0.31±0.01^{a}	0.31±0.03^{a}	0.30±0.00^{a}	≤1.00
C20:1	0.21±0.02^{a}	0.21±0.01^{a}	0.20±0.00^{a}	0.21±0.02^{a}	0.21±0.03^{a}	0.22±0.02^{a}	0.22±0.00^{a}	0.21±0.01^{a}	0.22±0.02^{a}	0.22±0.00^{a}	0.21±0.03^{a}	≤0.50
C22:0	0.07±0.00^{a}	0.07±0.01^{a}	0.07±0.01^{a}	0.07±0.00^{a}	0.07±0.00^{a}	0.08±0.01^{a}	0.07±0.00^{a}	0.07±0.01^{a}	0.07±0.00^{a}	0.07±0.00^{a}	0.06±0.01^{a}	≤0.20
C24:0	1.36±0.11^{a}	1.22±0.08^{a}	1.10±0.05^{a}	1.28±0.12^{a}	1.18±0.09^{a}	1.22±0.06^{a}	1.21±0.12^{a}	1.40±0.03^{a}	1.34±0.04^{a}	1.28±0.08^{a}	1.16±0.07^{a}	≤0.20

TSFAs	18.82±0.19^{a}	17.31±0.27^{a}	17.42±0.26^{a}	17.10±0.13^{a}	16.01±0.28^{a}	14.76±0.08^{b}	15.25±0.10^{b}	14.78±0.05^{b}	14.35±0.07^{b}	14.54±0.08^{b}	14.36±0.15^{b}
TMFAs	73.93±0.37^{bc}	75.33±0.29^{b}	75.32±0.41^{b}	75.62±0.57^{b}	76.53±0.08^{b}	77.87±0.16^{a}	77.43±0.09^{a}	77.90±0.41^{a}	78.20±0.33^{a}	77.84±0.14^{a}	78.14±0.15^{a}
TPUFAs	7.25±0.09^{a}	7.36±0.05^{a}	7.25±0.11^{a}	7.28±0.05^{a}	7.46±0.06^{a}	7.37±0.19^{a}	7.32±0.08^{a}	7.47±0.07^{a}	7.45±0.01^{a}	7.62±0.18^{a}	7.51±0.21^{a}

^{a,b,c,d,e,f,} and ^{g} Data in the same row followed by different superscript letters differ significantly (*P*<0.05). ND: Not detected. Refer to the caption of Table 1 for the meanings of the abbreviations used for the oil samples and fatty acids.

Table S12. Fatty acids content (%) of supplemented and non-supplemented olive oil 0.4° with HTyr under deep-frying conditions compared to the standard limits.

Fatty acid	Con 1	SOO	Con 2	Exp 1	Exp 2	Exp 3	Exp 4	Exp 5	Exp 6	Exp 7	Exp 8	Standard limits
C14:0	0.15±0.01^{a}	0.14±0.02^{a}	0.12±0.00^{b}	0.12±0.01^{b}	0.13±0.02^{ab}	0.05±0.01^{d}	0.08±0.01^{c}	0.11±0.00^{bc}	0.10±0.00^{bc}	0.08±0.01^{c}	0.09±0.02^{bc}	≤0.03
C16:0	11.68±0.13^{a}	11.39±0.07^{a}	11.30±0.06^{a}	10.96±0.19^{a}	11.73±0.21^{a}	10.93±0.18^{a}	11.04±0.08^{a}	11.31±0.11^{a}	10.35±0.07^{ab}	10.36±0.10^{ab}	10.27±0.06^{ab}	7.00-20.00
C16:1	0.83±0.03^{a}	0.76±0.02^{a}	0.78±0.01^{a}	0.74±0.01^{a}	0.78±0.00^{a}	0.77±0.02^{a}	0.77±0.01^{a}	0.78±0.03^{a}	0.72±0.02^{a}	0.75±0.08^{a}	0.74±0.03^{a}	0.30-3.50
C17:0	0.06±0.01^{a}	0.08±0.01^{a}	0.07±0.00^{a}	0.07±0.01^{a}	0.08±0.00^{a}	0.06±0.00^{a}	0.06±0.00^{a}	0.07±0.01^{a}	0.06±0.00^{a}	0.06±0.00^{a}	0.06±0.01^{a}	≤0.40
C17:1	0.09±0.00^{ab}	0.12±0.02^{a}	0.10±0.01^{a}	0.11±0.00^{a}	0.12±0.02^{a}	0.10±0.00^{a}	0.10±0.00^{a}	0.11±0.00^{a}	0.10±0.01^{a}	0.09±0.00^{a}	0.09±0.00^{a}	≤0.60
C18:0	2.38±0.11^{a}	2.49±0.09^{ab}	2.27±0.08^{ab}	2.31±0.07^{ab}	2.23±0.04^{b}	1.90±0.10^{c}	1.59±0.05^{d}	2.17±0.03^{b}	2.20±0.08^{b}	1.70±0.04^{d}	1.97±0.01^{c}	0.50-5.00
C18:1	73.35±0.33^{bc}	74.93±0.36^{ab}	74.65±0.21^{ab}	75.63±0.19^{a}	74.98±0.32^{ab}	75.73±0.09^{a}	76.12±0.12^{a}	74.67±0.13^{ab}	76.43±0.22^{a}	76.24±0.15^{a}	76.24±0.17^{a}	55.00-85.00
C18:2	9.45±0.06^{a}	8.12±0.04^{a}	8.69±0.12^{a}	8.21±0.13^{a}	8.10±0.15^{a}	8.66±0.09^{a}	8.41±0.06^{a}	8.83±0.03^{a}	8.23±0.12^{a}	8.92±0.01^{a}	8.78±0.11^{a}	2.50-21.00
C20:0	0.58±0.02^{a}	0.58±0.01^{a}	0.58±0.00^{a}	0.57±0.02^{a}	0.55±0.03^{a}	0.52±0.02^{a}	0.49±0.01^{ab}	0.57±0.01^{a}	0.54±0.03^{a}	0.53±0.02^{a}	0.48±0.02^{ab}	≤0.60
C18:3	0.34±0.01^{a}	0.33±0.02^{a}	0.33±0.02^{a}	0.31±0.03^{a}	0.32±0.00^{a}	0.32±0.01^{a}	0.32±0.02^{a}	0.33±0.00^{a}	0.30±0.03^{a}	0.31±0.03^{a}	0.30±0.02^{a}	≤1.00
C20:1	0.21±0.01^{a}	0.22±0.01^{a}	0.21±0.00^{a}	0.21±0.02^{a}	0.22±0.03^{a}	0.21±0.02^{a}	0.22±0.00^{a}	0.23±0.01^{a}	0.21±0.02^{a}	0.21±0.00^{a}	0.20±0.03^{a}	≤0.50
C22:0	0.06±0.00^{a}	0.06±0.01^{a}	0.06±0.01^{a}	0.06±0.01^{a}	0.06±0.01^{a}	0.06±0.00^{a}	0.06±0.00^{a}	0.06±0.00^{a}	0.05±0.01^{a}	0.05±0.01^{a}	0.05±0.00^{a}	≤0.20
C24:0	0.81±0.08^{a}	0.78±0.02^{a}	0.83±0.05^{a}	0.69±0.05^{a}	0.70±0.04^{a}	0.69±0.05^{ab}	0.73±0.02^{a}	0.75±0.05^{a}	0.70±0.04^{a}	0.71±0.06^{a}	0.72±0.04^{a}	≤0.20
TSFAs	15.73±0.10^{a}	15.53±0.21^{a}	15.24±0.09^{a}	14.79±0.23^{a}	15.48±0.07^{a}	14.21±0.17^{b}	14.05±0.11^{b}	15.05±0.09^{b}	14.01±0.17^{b}	13.49±0.10^{ab}	13.65±0.21^{ab}	
TMFAs	74.48±0.41^{b}	76.02±0.33^{a}	75.75±0.11^{ab}	76.69±0.09^{b}	76.10±0.38^{b}	76.81±0.10^{a}	77.21±0.18^{a}	75.79±0.16^{ab}	77.46±0.30^{a}	77.29±0.18^{a}	77.27±0.19^{a}	
TPUFAs	9.79±0.10^{a}	8.45±0.08^{a}	9.02±0.05^{a}	8.52±0.12^{a}	8.42±0.09^{a}	8.98±0.13^{a}	8.74±0.07^{a}	9.17±0.11^{a}	8.53±0.06^{a}	9.23±0.07^{a}	9.08±0.16^{a}	

^{a,b,c,d,e,f,} and ^{g} Data in the same row followed by different superscript letters differ significantly (*P*<0.05). ND: Not detected. Refer to the caption of Table 1 for the meanings of the abbreviations used for the oil samples and fatty acids.

Table S13. Fatty acids content (mg/kg) of supplemented and non-supplemented EVOO Picual with HTyr under deep-frying conditions.

Fatty acid	Control 1	SOO	Control 2	Exp 1	Exp 2	Exp 3	Exp 4	Exp 5	Exp 6	Exp 7	Exp 8
C14:0	89.80±1.22	87.78±10.31	102.64±0.74	82.40±5.01	80.06±1.03	53.34±7.32	56.94±6.67	89.39±4.87	86.76±0.15	76.76±0.14	61.40±15.74
C16:0	1147.45±13.17	971.05±79.79	1102.74±5.04	908.13±2.64	921.26±11.90	764.23±33.48	807.53±68.37	982.17±26.02	901.46±0.66	1028.53±1.89	902.09±76.94
C16:1	79.10±1.28	69.49±5.61	79.16±0.72	66.59±2.85	65.21±0.84	75.59±6.87	59.89±4.82	66.59±2.85	64.10±0.64	73.53±0.14	65.53±6.73
C17:0	6.94±0.11	6.73±0.56	7.75±0.08	8.11±0.67	7.40±0.10	2.97±0.62	4.29±0.15	8.11±0.67	5.92±0.07	7.12±0.01	6.21±0.62
C17:1	14.58±1.46	13.49±1.47	16.14±0.18	13.53±0.14	13.59±0.18	7.74±0.55	8.50±0.42	13.53±0.14	11.96±0.23	13.78±0.03	12.89±0.72
C18:0	477.76±8.16	503.81±44.42	642.61±2.42	495.16±1.23	453.42±5.86	381.54±39.35	341.92±29.98	515.40±29.85	512.39±3.89	441.19±0.81	349.19±96.16
C18:1	5880.25±19.05	5626.34±155.86	5655.41±23.14	5674.13±145.11	5392.51±70.94	5143.98±98.54	4737.56±310.89	5774.13±145.11	5519.65±15.11	5789.22±11.40	4994.83±407.61
C18:2	703.54±13.26	610.76±44.59	682.61±2.88	543.83±18.14	591.31±7.64	552.07±7.38	497.72±39.62	570.21±19.17	562.48±2.15	632.03±1.16	546.07±47.19
C20:0	52.14±0.13	51.52±5.01	58.45±0.46	49.04±2.73	50.41±0.65	31.59±1.07	30.29±2.49	49.04±2.73	44.60±0.62	50.79±0.09	43.02±1.84
C18:3	26.98±1.97	23.66±2.16	27.80±0.20	24.49±1.33	24.40±0.32	13.80±0.39	15.71±1.02	24.49±1.33	20.41±0.42	25.28±0.05	22.50±1.52
C20:1	19.73±0.62	17.54±1.80	20.07±0.40	17.59±1.12	16.92±0.22	13.33±0.01	12.45±0.79	17.59±1.12	15.08±0.18	18.24±0.03	16.38±1.43
C22:0	4.00±0.03	ND	6.42±0.00	ND	ND	ND	ND	ND	ND	ND	ND
C24:0	188.49±2.95	199.63±20.93	232.53±1.48	181.32±9.68	160.19±2.07	163.25±12.82	147.62±5.77	192.65±8.92	189.84±0.28	201.95±0.37	162.62±14.76

ND: not detected.

For the olive oil categories: Control 1 (used as the control for Experiments 1–4) refers to original, non-deep-fried olive oil. SOO (Supplemented olive oil) refers to non-deep-fried olive oil that has been enriched with olive fruit extract, which is also used in the preparation of Control 2. Control 2 (used as the control for Experiments 5–8) is a mixture of Con 1 and the supplemented oil, resulting in a total polyphenol content of up to 650 mg/kg. Exp.1: olive oil deep fried at 170 °C for 3 h without polyphenol supplementation, Exp.2: olive oil deep fried at 170 °C for 6 h without polyphenol supplementation, Exp.3: olive oil deep fried at 210 °C for 3 h without polyphenol supplementation, Exp.4: olive oil deep fried at 210 °C for 6 h without polyphenol supplementation, Exp.5: olive oil deep fried at 170 °C for 3 h with polyphenol supplementation, Exp.6: olive oil deep fried at 170 °C for 6 h with polyphenol supplementation, Exp.7: olive oil deep fried at 210 °C for 3 h with polyphenol supplementation, Exp.8: olive oil deep fried at 210 °C for 6 h with polyphenol supplementation.

C14:0 (Myristic acid), C16:0 (Palmitic acid), C16:1 (n−7) (Palmitoleic acid), C17:0 (Margarinic acid), C17:1 (n−7) (cis-10-Heptadecenoic acid), C18:0 (Stearic acid), C18:1 (n−9) (Oleic acid), C18:2 (n−6) (Linoleic acid), C20:0 (Arachidic acid), C18:3 (n−3) (α-Linolenic acid), C20:1 (n−9) (Gadoleic acid), C22:0 (Behenic acid), C24:0 (Lignoceric acid).

Table S14. Fatty acids content (mg/kg) of supplemented and non-supplemented EVOO Cornicabra with HTyr under deep-frying conditions.

Fatty acid	Control 1	SOO	Control 2	Exp 1	Exp 2	Exp 3	Exp 4	Exp 5	Exp 6	Exp 7	Exp 8
**C14:0**	82.43±3.18	77.30±6.02	81.65±2.48	88.97±0.74	67.29±6.12	83.45±11.62	69.81±0.51	81.58±2.45	69.57±2.85	60.60±0.66	44.84±2.95
**C16:0**	1342.97±53.00	1269.68±119.99	1202.00±92.30	1254.18±9.84	1164.07±41.03	1186.87±83.80	1168.01±9.98	1148.43±29.32	1169.71±42.78	813.13±9.04	772.57±63.69
**C16:1**	129.62±4.31	122.35±11.26	115.81±8.92	121.57±0.87	111.89±3.91	113.63±7.31	112.49±0.94	102.03±2.67	111.61±4.00	78.11±0.84	68.61±6.92
**C17:0**	5.34±0.38	5.81±1.13	5.24±0.77	5.14±0.06	5.95±1.92	4.87±0.48	4.91±0.23	5.85±0.14	4.62±0.10	5.28±0.06	4.49±1.03
**C17:1**	9.11±0.47	9.35±0.80	7.65±0.36	9.18±0.17	12.48±7.05	8.88±0.60	10.90±0.31	11.65±0.30	8.48±0.29	8.55±0.16	ND
**C18:0**	391.64±9.20	351.12±22.23	377.07±18.85	384.20±3.75	289.18±27.41	363.74±55.73	299.40±2.72	365.49±7.63	317.80±12.31	278.48±5.06	186.65±12.77
**C18:1**	6028.68±122.26	5621.53±465.81	5643.82±73.98	5758.62±47.55	5297.91±186.21	5583.80±197.43	5307.09±47.62	5408.68±101.47	5401.94±197.55	4516.58±57.15	4189.05±30.37
**C18:2**	990.46±40.79	922.29±84.18	874.63±69.20	919.74±7.76	839.02±29.02	856.74±41.51	828.15±7.31	832.14±9.73	837.24±30.77	600.95±7.44	563.99±31.09
**C20:0**	72.07±3.24	58.62±5.00	59.00±0.26	64.66±0.88	52.48±1.85	52.42±2.37	54.25±0.37	55.82±2.19	52.96±2.05	37.48±0.52	25.34±2.54
**C18:3**	35.66±1.71	34.29±3.60	33.34±2.63	34.91±0.37	32.79±1.23	33.05±2.26	32.42±0.24	29.91±0.63	33.79±1.33	23.43±1.27	15.01±0.86
**C20:1**	20.08±0.98	18.78±2.07	18.37±1.41	18.92±0.22	17.61±0.71	17.59±1.23	17.34±0.12	16.12±0.46	18.32±0.72	12.31±0.37	10.01±1.23
**C22:0**	8.69±0.42	8.49±1.04	8.12±0.36	8.91±0.20	8.41±0.32	8.54±0.54	8.38±0.05	7.79±0.10	10.16±0.36	5.68±0.09	ND
**C24:0**	104.41±4.56	114.66±7.83	106.36±13.54	103.09±0.36	95.20±3.81	105.64±6.85	95.58±0.85	94.41±1.59	105.71±3.96	82.97±0.97	74.85±2.91

ND: not detected. Refer to Table S2 caption for abbreviations meaning of the oil samples. C14:0 (Myristic acid), C16:0 (Palmitic acid), C16:1 (n−7) (Palmitoleic acid), C17:0 (Margarinic acid), C17:1 (n−7) (cis-10-Heptadecenoic acid), C18:0 (Stearic acid), C18:1 (n−9) (Oleic acid), C18:2 (n−6) (Linoleic acid), C20:0 (Arachidic acid), C18:3 (n−3) (α-Linolenic acid), C20:1 (n−9) (Gadoleic acid), C22:0 (Behenic acid), C24:0 (Lignoceric acid).

Table S15. Fatty acids content (mg/kg) of supplemented and non-supplemented EVOO Empeltre with HTyr under deep-frying conditions.

Fatty acid	Control 1	SOO	Control 2	Exp 1	Exp 2	Exp 3	Exp 4	Exp 5	Exp 6	Exp 7	Exp 8
**C14:0**	42.66±0.20	39.39±4.92	38.87±1.98	33.91±2.97	9.19±1.06	9.67±0.76	4.49±0.48	ND	ND	ND	ND
**C16:0**	828.26±8.12	813.83±71.71	790.69±5.95	747.06±20.14	742.37±7.55	762.19±1.64	728.16±12.83	742.16±9.12	657.38±93.03	692.38±15.77	381.61±16.59
**C16:1**	61.23±0.25	62.18±5.75	60.05±1.19	57.02±1.54	57.23±0.59	59.26±0.15	56.33±1.20	56.94±0.61	51.28±7.49	54.03±0.76	32.98±2.48
**C17:0**	8.35±0.07	4.43±0.37	9.94±1.25	8.23±0.65	4.77±0.27	4.51±0.05	3.94±0.11	3.38±0.04	2.84±0.59	2.57±0.14	1.24±0.16
**C17:1**	15.91±0.79	11.19±0.48	9.11±0.41	12.90±1.98	10.96±0.15	11.04±0.14	9.36±0.31	8.08±0.11	7.12±1.31	6.74±0.16	3.51±0.27
**C18:0**	286.86±0.05	283.71±6.70	276.87±16.14	189.60±5.48	74.21±2.33	82.66±0.80	50.36±4.74	21.52±1.02	18.85±3.34	14.82±0.58	5.07±0.79
**C18:1**	4301.14±106.32	4638.78±43.56	4258.22±1.95	3994.85±109.08	3953.86±12.15	4088.25±16.20	3983.00±63.49	4280.36±95.23	3889.28±320.85	4554.13±19.18	3354.12±105.44
**C18:2**	635.67±3.14	663.73±4.86	598.50±16.31	558.70±13.51	571.81±5.15	593.23±2.11	570.17±10.16	651.07±13.75	576.28±70.52	648.73±0.23	476.55±30.24
**C20:0**	44.01±0.05	41.98±3.78	40.88±0.57	38.03±2.39	38.12±0.15	37.58±0.18	33.51±0.93	39.02±0.50	35.21±3.50	36.14±0.38	22.85±1.09
**C18:3**	16.53±0.49	16.19±1.48	15.93±0.23	16.33±0.67	16.00±0.35	16.35±0.15	15.33±0.41	15.36±0.03	13.21±1.89	13.60±0.31	7.06±0.19
**C20:1**	14.67±0.67	14.03±1.31	14.00±0.06	16.62±1.12	13.85±0.53	14.24±0.27	13.32±0.28	13.65±0.02	11.67±1.79	11.72±0.23	6.46±0.63
**C22:0**	4.71±0.16	4.06±0.47	4.32±0.31	4.00±0.37	3.99±0.09	4.13±0.15	3.66±0.16	3.32±0.10	2.83±0.45	2.78±0.08	ND
**C24:0**	81.24±0.77	92.39±0.91	86.11±0.30	85.39±6.37	77.93±0	77.90±0.23	72.64±0.67	82.86±0.11	72.54±8.15	82.15±0.65	58.81±3.23

ND: not detected. Refer to Table S2 caption for abbreviations meaning of the oil samples. C14:0 (Myristic acid), C16:0 (Palmitic acid), C16:1 (n−7) (Palmitoleic acid), C17:0 (Margarinic acid), C17:1 (n−7) (cis-10-Heptadecenoic acid), C18:0 (Stearic acid), C18:1 (n−9) (Oleic acid), C18:2 (n−6) (Linoleic acid), C20:0 (Arachidic acid), C18:3 (n−3) (α-Linolenic acid), C20:1 (n−9) (Gadoleic acid), C22:0 (Behenic acid), C24:0 (Lignoceric acid).

Table S16. Fatty acids content (mg/kg) of supplemented and non-supplemented EVOO Arbequina with HTyr under deep-frying conditions.

Fatty acid	Control 1	SOO	Control 2	Exp 1	Exp 2	Exp 3	Exp 4	Exp 5	Exp 6	Exp 7	Exp 8
**C14:0**	5.66±0.27	5.72±0.06	6.50±3.40	2.51±0.63	2.15±0.62	1.87±0.85	3.88±0.28	4.05±0.18	2.84±1.43	3.31±0.12	4.11±0.55
**C16:0**	637.35±20.38	598.27±9.61	616.45±4.70	570.12±18.33	591.10±56.22	535.08±46.53	579.41±25.74	566.27±25.12	595.34±24.70	577.36±24.15	576.29±18.31
**C16:1**	87.90±4.96	69.71±0.48	74.78±9.40	64.10±2.36	65.87±4.69	60.58±4.80	64.58±2.58	63.81±2.71	65.76±2.62	65.48±2.11	74.90±0.17
**C17:0**	ND	ND	ND	ND	ND	ND	ND	ND	ND	ND	ND
**C17:1**	2.47±0.07	2.26±0.05	2.55±0.48	2.08±0.18	2.21±0.26	1.96±0.18	2.12±0.12	2.05±0.08	2.19±0.14	2.12±0.07	2.29±0.21
**C18:0**	112.23±3.29	71.16±2.42	70.33±5.27	46.45±1.45	33.94±2.12	32.29±3.83	62.05±6.44	73.29±1.53	73.21±6.89	52.45±2.24	57.47±1.25
**C18:1**	4029.32±141.05	4046.16±4.20	4006.26±83.74	3780.39±117.23	3799.85±197.36	3675.57±176.86	3717.03±159.29	3773.33±54.96	3664.23±155.73	3792.70±81.61	3696.28±121.11
**C18:2**	760.63±67.79	777.02±0.52	817.56±87.50	711.95±17.06	712.66±29.47	667.93±56.36	696.11±29.50	699.14±28.20	691.07±31.89	717.09±15.20	699.19±20.89
**C20:0**	41.35±3.07	36.40±0.23	32.42±3.85	28.61±2.06	28.68±3.30	32.93±1.15	25.68±1.41	31.45±1.33	34.37±0.72	28.66±0.57	28.05±3.04
**C18:3**	14.33±1.81	10.89±0.28	11.87±1.01	10.05±0.58	10.59±0.81	9.59±0.85	10.59±0.75	10.77±0.45	11.31±0.51	11.28±0.31	13.10±0.23
**C20:1**	4.07±0.04	5.54±0.08	6.05±0.49	5.05±0.31	5.29±0.36	4.85±0.45	5.33±0.33	5.55±0.22	5.75±0.24	5.81±0.16	7.30±0.43
**C22:0**	ND	ND	ND	ND	ND	ND	ND	ND	ND	ND	ND
**C24:0**	47.23±5.12	38.82±0.23	45.11±4.01	41.17±3.03	40.30±0.62	36.51±3.23	42.84±2.30	36.54±1.46	37.25±0.69	38.31±0.76	34.64±1.13

ND: not detected. Refer to Table S2 caption for abbreviations meaning of the oil samples. C14:0 (Myristic acid), C16:0 (Palmitic acid), C16:1 (n−7) (Palmitoleic acid), C17:0 (Margarinic acid), C17:1 (n−7) (cis-10-Heptadecenoic acid), C18:0 (Stearic acid), C18:1 (n−9) (Oleic acid), C18:2 (n−6) (Linoleic acid), C20:0 (Arachidic acid), C18:3 (n−3) (α-Linolenic acid), C20:1 (n−9) (Gadoleic acid), C22:0 (Behenic acid), C24:0 (Lignoceric acid).

Table S17. Fatty acids content (mg/kg) of supplemented and non-supplemented EVOO Hojiblanca with HTyr under deep-frying conditions.

Fatty acid	Control 1	SOO	Control 2	Exp 1	Exp 2	Exp 3	Exp 4	Exp 5	Exp 6	Exp 7	Exp 8
**C14:0**	2.19±0.08	2.05±0.16	2.43±0.19	1.69±0.30	ND	ND	ND	ND	ND	ND	ND
**C16:0**	645.86±28.14	643.80±9.35	686.14±5.19	611.59±12.72	621.20±37.88	655.29±10.12	649.62±39.39	608.48±20.97	597.09±15.49	558.86±23.67	543.90±24.04
**C16:1**	48.98±0.63	50.94±0.68	54.63±0.54	48.55±0.79	51.94±1.35	52.66±0.59	50.79±4.69	50.42±1.11	49.02±1.15	46.90±1.19	44.22±1.95
**C17:0**	1.65±0.13	1.64±0.05	1.86±0.06	1.68±0.08	1.71±0.07	2.89±1.35	4.49±0.44	2.86±0.09	2.25±0.15	1.86±0.19	ND
**C17:1**	2.77±0.06	2.86±0.13	3.41±0.17	3.12±0.11	3.25±0.10	3.58±0.20	4.12±0.19	3.14±0.16	2.87±0.23	2.95±0.78	4.02±0.19
**C18:0**	41.02±0.76	36.77±1.52	39.93±0.84	19.86±0.99	18.04±0.75	14.56±1.87	11.87±0.51	13.51±0.64	8.61±0.61	9.85±0.27	7.57±0.37
**C18:1**	4432.10±81.38	4466.92±26.15	4622.36±30.81	4087.17±38.18	4198.85±179.90	4140.09±64.92	3930.51±53.35	3932.90±44.75	3981.61±91.19	3946.59±98.33	4013.23±187.27
**C18:2**	485.59±9.84	494.37±1.50	515.24±2.66	450.26±4.51	466.55±18.98	464.82±6.49	440.21±8.24	440.52±15.38	446.35±9.78	440.60±12.99	441.37±20.62
**C20:0**	35.52±0.81	31.99±0.62	34.91±0.31	30.19±0.38	36.42±5.78	39.20±2.92	37.15±3.39	30.95±0.18	28.98±0.74	37.36±0.64	32.60±1.51
**C18:3**	15.08±0.69	16.53±0.61	18.77±0.50	17.29±0.42	18.31±0.61	19.16±0.34	21.89±1.29	17.26±0.89	16.38±0.42	15.64±0.51	15.06±0.74

**C20:1**	9.10±0.45	10.47±0.37	11.29±0.29	10.53±0.25	10.84±0.37	11.44±0.16	11.90±1.03	10.06±0.98	9.92±0.22	9.57±0.20	9.08±0.41
**C22:0**	2.46±0.22	2.77±0.18	3.34±0.18	3.15±0.14	3.41±0.07	3.71±0.08	4.12±0.39	3.27±0.26	2.92±0.09	2.78±0.08	2.55±0.11
**C24:0**	75.18±2.60	78.49±0.21	84.15±0.08	72.40±0.86	65.97±2.77	71.87±2.50	75.91±4.85	69.75±1.74	69.26±1.13	69.00±1.27	70.35±3.42

ND: not detected. Refer to Table S2 caption for abbreviations meaning of the oil samples. C14:0 (Myristic acid), C16:0 (Palmitic acid), C16:1 (n−7) (Palmitoleic acid), C17:0 (Margarinic acid), C17:1 (n−7) (cis-10-Heptadecenoic acid), C18:0 (Stearic acid), C18:1 (n−9) (Oleic acid), C18:2 (n−6) (Linoleic acid), C20:0 (Arachidic acid), C18:3 (n−3) (α-Linolenic acid), C20:1 (n−9) (Gadoleic acid), C22:0 (Behenic acid), C24:0 (Lignoceric acid).

Table S18. Fatty acids content (mg/kg) of supplemented and non-supplemented EVOO Manzanilla with HTyr under deep-frying conditions.

**Fatty acid**	**Control 1**	**SOO**	**Control 2**	**Exp 1**	**Exp 2**	**Exp 3**	**Exp 4**	**Exp 5**	**Exp 6**	**Exp 7**	**Exp 8**
**C14:0**	ND	ND	ND	ND	ND	ND	ND	ND	ND	ND	ND
**C16:0**	588.24±28.43	496.60±18.14	513.52±8.81	571.49±2.26	540.74±34.64	488.24±9.42	475.05±10.65	534.45±12.99	490.81±48.00	456.76±2.07	451.77±15.84
**C16:1**	51.71±2.78	44.06±3.09	45.74±0.61	50.39±0.19	47.97±4.24	42.68±1.06	42.30±1.44	49.65±2.21	43.31±3.28	44.32±0.73	43.71±1.46
**C17:0**	ND	ND	ND	ND	ND	ND	ND	ND	ND	ND	ND
**C17:1**	2.10±0.09	1.72±0.11	1.83±0.13	2.65±0.12	2.00±0.26	1.67±0.03	1.63±0.01	1.96±0.15	1.72±0.12	1.78±0.08	2.20±0.12
**C18:0**	12.59±1.98	7.42±1.91	7.36±0.16	9.56±0.74	7.35±0.80	7.12±0.21	6.52±0.52	7.10±0.22	6.55±0.11	5.57±0.23	6.23±0.41
**C18:1**	4237.47±194.04	4210.87±98.20	4422.90±38.76	4279.86±63.48	4048.79±69.53	4030.36±	3931.22±43.32	4357.74±56.37	4344.56±175.19	4068.03±73.67	3875.19±94.72
**C18:2**	249.50±10.33	214.23±9.61	238.29±13.72	249.39±3.16	248.13±18.40	223.36±2.27	232.62±14.81	275.06±14.34	246.55±7.21	239.67±2.94	219.20±7.59
**C20:0**	28.12±0.36	31.34±0.28	29.01±4.21	28.85±0.02	25.83±1.15	24.13±0.56	21.80±0.21	28.97±0.95	27.08±1.39	27.19±1.14	24.09±0.92
**C18:3**	11.14±0.45	9.21±0.64	10.17±0.25	11.37±0.21	12.05±1.49	10.12±0.25	9.93±0.26	11.43±0.48	10.55±0.61	10.17±0.17	9.76±0.35
**C20:1**	10.86±0.34	9.07±0.60	9.93±0.29	11.07±0.15	10.51±0.27	10.04±0.27	9.58±0.19	10.92±0.35	10.48±0.56	9.99±0.26	9.40±0.28
**C22:0**	2.16±0.13	ND	ND	2.04±0.03	2.07±0.33	ND	ND	ND	ND	ND	ND
**C24:0**	177.57±7.48	150.87±1.24	168.71±10.96	183.71±2.51	163.15±8.95	173.40±2.80	158.38±4.62	177.98±7.71	189.44±0.64	175.48±1.75	160.38±0.85

ND: not detected. Refer to Table S2 caption for abbreviations meaning of the oil samples. C14:0 (Myristic acid), C16:0 (Palmitic acid), C16:1 (n−7) (Palmitoleic acid), C17:0 (Margarinic acid), C17:1 (n−7) (cis-10-Heptadecenoic acid), C18:0 (Stearic acid), C18:1 (n−9) (Oleic acid), C18:2 (n−6) (Linoleic acid), C20:0 (Arachidic acid), C18:3 (n−3) (α-Linolenic acid), C20:1 (n−9) (Gadoleic acid), C22:0 (Behenic acid), C24:0 (Lignoceric acid).

Table S19. Fatty acids content (mg/kg) of supplemented and non-supplemented EVOO Royuela with HTyr under deep-frying conditions.

Fatty acid	Control 1	SOO	Control 2	Exp 1	Exp 2	Exp 3	Exp 4	Exp 5	Exp 6	Exp 7	Exp 8
**C14:0**	3.04±0.02	ND	ND	ND	ND	ND	ND	ND	ND	ND	ND
**C16:0**	621.12±6.08	568.16±2.00	622.06±3.39	646.74±18.70	648.95±21.72	660.92±5.15	759.80±8.39	689.46±18.32	699.50±6.73	715.05±3.36	749.20±31.50
**C16:1**	41.28±3.10	41.72±1.01	45.15±0.54	46.68±1.21	47.33±2.10	46.85±0.49	53.63±0.65	48.47±1.29	49.32±0.40	50.29±0.86	52.50±2.17
**C17:0**	2.09±0.54	2.03±0.02	2.51±0.07	2.46±0.15	3.22±0.26	3.05±0.02	3.83±0.06	3.47±0.09	3.72±0.18	4.16±0.37	4.36±0.20
**C17:1**	4.49±1.44	4.42±0.10	5.04±0.10	5.54±0.35	6.33±0.57	6.36±0.01	7.86±0.14	7.57±0.20	8.43±1.36	9.18±0.35	9.53±0.33
**C18:0**	39.14±14.68	14.85±0.53	12.73±0.90	12.27±0.40	13.21±0.95	12.99±0.37	14.36±0.27	17.97±0.48	14.47±0.19	15.06±1.19	13.40±0.77
**C18:1**	4001.53±17.40	3878.65±93.84	4094.66±36.04	4059.09±38.87	3838.34±111.17	3859.74±44.21	3707.65±34.92	4129.94±56.33	3903.22±0.55	3917.34±17.13	3976.99±182.37
**C18:2**	511.49±2.39	499.61±13.16	529.34±4.47	527.69±5.73	494.81±15.15	496.91±5.41	516.31±4.86	507.43±13.48	504.56±0.21	510.35±1.93	529.72±22.45
**C20:0**	25.67±0.66	28.04±3.90	26.00±0.08	26.45±0.66	27.64±1.31	26.12±0.15	28.62±0.20	45.08±1.20	27.99±0.17	42.91±11.50	43.23±3.94
**C18:3**	6.93±0.83	7.77±0.24	9.06±0.04	9.74±0.47	10.71±0.71	10.94±0.08	13.18±0.08	11.72±0.31	12.29±0.33	13.20±0.18	13.94±0.58
**C20:1**	6.14±0.23	6.41±0.13	7.39±0.03	8.04±0.35	8.47±0.54	8.92±0.04	10.68±0.03	9.66±0.26	9.95±0.23	10.74±0.30	11.33±0.48
**C22:0**	ND	ND	ND	ND	ND	ND	ND	ND	ND	ND	ND
**C24:0**	156.81±1.76	126.38±6.05	140.07±0.05	143.79±2.06	138.50±5.54	144.90±1.60	154.60±0.81	154.22±4.10	153.07±0.39	157.97±0.79	165.11±7.35

ND: not detected. Refer to Table S2 caption for abbreviations meaning of the oil samples. C14:0 (Myristic acid), C16:0 (Palmitic acid), C16:1 (n−7) (Palmitoleic acid), C17:0 (Margarinic acid), C17:1 (n−7) (cis-10-Heptadecenoic acid), C18:0 (Stearic acid), C18:1 (n−9) (Oleic acid), C18:2 (n−6) (Linoleic acid), C20:0 (Arachidic acid), C18:3 (n−3) (α-Linolenic acid), C20:1 (n−9) (Gadoleic acid), C22:0 (Behenic acid), C24:0 (Lignoceric acid).

Table S20. Fatty acids content (mg/kg) of supplemented and non-supplemented Orujo oil (pomace olive oil) with HTyr under deep-frying conditions.

Fatty acid	Control 1	SOO	Control 2	Exp 1	Exp 2	Exp 3	Exp 4	Exp 5	Exp 6	Exp 7	Exp 8
**C14:0**	2.00±0.34	..1.91±0.45	1.62±0.03	1.89±0.30	1.46±0.11	ND	1.43±0.03	2.28±0.17	1.92±0.67	3.26±0.12	1.68±0.02
**C16:0**	658.43±7.95	729.42±18.28	688.58±11.69	617.58±13.52	611.45±37.87	525.70±16.94	566.09±0.85	628.13±21.43	651.34±30.73	578.18±5.58	578.37±22.51
**C16:1**	50.87±1.03	56.32±1.22	55.71±1.06	49.22±2.61	48.44±2.19	41.45±1.09	43.02±0.07	46.72±1.54	49.46±2.47	44.08±0.36	44.31±1.80
**C17:0**	3.34±0.01	4.51±0.33	3.72±0.07	3.59±0.18	3.16±0.09	2.08±0.25	3.03±0.03	3.11±0.34	3.53±0.47	3.11±0.00	3.42±0.07
**C17:1**	6.44±0.07	7.28±0.17	6.69±0.20	6.80±0.17	6.09±0.09	4.28±0.38	6.30±0.05	5.53±0.53	6.01±0.52	5.30±0.02	5.86±0.12
**C18:0**	17.21±2.28	16.22±0.38	13.51±0.25	13.27±0.41	13.70±0.42	11.76±1.53	13.83±0.44	21.51±0.88	20.31±4.30	22.85±0.35	13.13±0.52
**C18:1**	4027.62±55.34	4288.76±48.61	4069.96±65.93	3975.11±124.63	3709.25±178.20	3534.52±66.52	3490.57±9.66	3998.37±62.23	3913.28±154.75	3543.45±59.94	3454.47±151.96
**C18:2**	590.97±5.73	643.20±10.46	616.48±10.06	607.49±13.64	552.00±39.83	522.20±11.46	497.63±1.15	584.67±10.32	601.69±12.05	510.46±8.26	495.47±21.50
**C20:0**	45.79±5.70	44.35±1.37	39.98±0.90	39.45±2.05	35.57±1.45	29.11±1.10	30.17±0.53	36.04±1.82	37.59±1.68	30.65±0.42	31.02±0.22

**C18:3**	21.60±0.37	22.36±0.74	21.39±0.46	20.47±0.67	19.03±1.24	15.76±1.08	15.83±0.14	19.17±0.16	19.09±1.01	18.92±0.11	17.38±0.49
**C20:1**	14.94±0.24	16.81±0.45	16.12±0.32	15.71±0.51	14.62±0.97	10.92±0.72	12.02±0.11	13.78±1.10	13.99±1.12	12.70±0.03	13.19±0.35
**C22:0**	5.98±0.25	7.10±0.24	6.91±0.22	6.33±0.40	6.14±0.35	3.75±0.44	4.55±0.06	5.26±0.88	5.34±0.70	5.04±0.12	5.42±0.17
**C24:0**	43.67±2.25	47.63±2.35	41.59±0.84	41.63±1.77	36.44±1.04	39.11±4.07	34.79±0.23	41.68±0.40	41.29±2.09	42.79±0.33	36.86±1.33

ND: not detected. Refer to Table S2 caption for abbreviations meaning of the oil samples. C14:0 (Myristic acid), C16:0 (Palmitic acid), C16:1 (n−7) (Palmitoleic acid), C17:0 (Margarinic acid), C17:1 (n−7) (cis-10-Heptadecenoic acid), C18:0 (Stearic acid), C18:1 (n−9) (Oleic acid), C18:2 (n−6) (Linoleic acid), C20:0 (Arachidic acid), C18:3 (n−3) (α-Linolenic acid), C20:1 (n−9) (Gadoleic acid), C22:0 (Behenic acid), C24:0 (Lignoceric acid).

Table S21. Fatty acids content (mg/kg) of supplemented and non-supplemented EVOO Koroneiki with HTyr under deep-frying conditions.

**Fatty acid**	**Control 1**	**SOO**	**Control 2**	**Exp 1**	**Exp 2**	**Exp 3**	**Exp 4**	**Exp 5**	**Exp 6**	**Exp 7**	**Exp 8**
**C14:0**	55.86±1.09	51.16±2.00	57.12±2.18	8.18±0.49	8.61±0.22	6.96±0.08	7.48±0.71	9.12±0.88	10.23±0.28	10.28±0.59	10.00±0.73
**C16:0**	819.60±23.02	773.06±3.50	744.21±18.47	834.44±9.76	833.24±39.40	842.79±22.05	854.86±3.84	855.51±26.74	881.04±2.23	948.29±9.48	974.05±6.93
**C16:1**	55.82±1.98	52.56±0.31	51.28±1.19	58.79±0.61	58.78±2.89	59.14±1.99	60.95±0.49	60.41±2.47	59.76±0.94	72.15±2.78	68.48±1.39
**C17:0**	2.48±0.17	2.34±0.04	2.29±0.01	3.17±0.01	3.13±0.42	3.14±0.08	4.03±0.67	3.79±0.38	3.32±0.02	4.58±1.01	4.35±0.31
**C17:1**	3.65±0.23	4.01±0.62	5.81±0.16	4.61±0.07	4.48±0.28	4.51±0.25	5.27±0.72	5.69±0.87	4.80±0.13	6.69±0.56	7.21±0.23
**C18:0**	416.88±2.12	362.28±2.40	402.28±13.54	61.59±2.64	65.41±1.38	40.94±0.27	63.57±1.45	56.91±0.95	59.01±1.63	67.53±4.23	72.20±1.89
**C18:1**	5118.27±55.40	4728.14±5.22	4832.42±27.60	4991.08±43.73	5020.98±126.07	5037.81±99.38	4960.98±32.83	5029.19±71.48	5130.70±76.48	5065.38±84.34	5103.30±22.69
**C18:2**	381.04±4.82	350.97±0.61	339.35±4.40	369.86±3.73	376.57±7.44	375.43±11.58	372.14±1.98	385.57±2.12	371.37±5.65	444.74±15.67	376.98±5.88
**C20:0**	39.09±1.65	33.84±0.19	33.01±0.28	34.87±0.62	36.46±1.15	39.47±0.99	35.31±0.22	42.42±1.33	40.33±0.78	43.32±1.77	40.66±1.80
**C18:3**	22.05±0.96	18.22±0.16	18.00±0.39	20.94±0.49	21.09±0.78	21.75±0.92	21.95±0.21	22.54±0.84	22.85±0.44	27.56±1.49	25.28±2.21
**C20:1**	15.65±1.73	13.73±0.09	13.56±0.20	15.58±0.38	15.68±0.61	16.32±0.64	16.34±0.15	16.56±0.55	17.12±0.23	20.23±0.90	19.10±0.63
**C22:0**	4.67±0.30	4.28±0.07	4.32±0.11	5.07±0.27	5.22±0.12	5.39±0.29	5.71±0.10	5.88±0.38	6.08±0.07	7.57±0.43	7.22±0.10
**C24:0**	133.82±0.79	109.09±0.59	117.57±1.36	137.72±0.82	143.57±3.26	141.07±2.54	149.08±2.82	151.59±3.74	139.36±1.06	157.85±7.65	151.19±1.59

Refer to Table S2 caption for abbreviations meaning of the oil samples. C14:0 (Myristic acid), C16:0 (Palmitic acid), C16:1 (n−7) (Palmitoleic acid), C17:0 (Margarinic acid), C17:1 (n−7) (cis-10-Heptadecenoic acid), C18:0 (Stearic acid), C18:1 (n−9) (Oleic acid), C18:2 (n−6) (Linoleic acid), C20:0 (Arachidic acid), C18:3 (n−3) (α-Linolenic acid), C20:1 (n−9) (Gadoleic acid), C22:0 (Behenic acid), C24:0 (Lignoceric acid).

Table S22. Fatty acids content (mg/kg) of supplemented and non-supplemented EVOO Arbosana with HTyr under deep-frying conditions.

Fatty acid	Control 1	SOO	Control 2	Exp 1	Exp 2	Exp 3	Exp 4	Exp 5	Exp 6	Exp 7	Exp 8
**C14:0**	10.03±1.02	7.55±0.21	6.54±0.35	6.44±0.89	6.13±0.92	2.74±0.39	2.68±0.37	5.82±0.89	5.33±0.85	3.57±0.29	2.87±0.15
**C16:0**	1041.66±26.94	1010.43±10.42	950.96±5.54	904.01±37.43	1015.62±6.03	1062.73±46.60	976.70±5.05	931.48±33.49	905.52±27.39	899.64±13.81	804.42±44.85
**C16:1**	87.29±0.41	84.81±0.83	79.00±0.58	75.66±2.97	86.17±2.72	90.07±3.63	83.44±0.29	79.17±3.61	76.23±2.25	76.28±1.09	67.50±3.64
**C17:0**	7.82±0.73	8.10±0.04	6.69±0.23	7.16±0.55	7.61±0.70	8.31±0.66	6.94±0.28	6.64±0.35	6.70±0.57	6.57±0.54	5.30±0.27
**C17:1**	15.84±0.55	14.46±00	14.56±0.36	13.36±1.10	14.97±0.71	14.98±0.85	15.73±0.67	13.96±0.94	13.15±0.89	12.84±0.65	12.05±0.62
**C18:0**	58.75±4.24	45.47±0.28	39.93±1.33	44.58±2.57	37.52±6.18	29.37±1.19	27.37±1.33	39.92±1.98	31.51±3.94	27.13±1.57	23.08±1.07
**C18:1**	5339.56±42.32	5125.76±67.89	4863.99±44.32	4682.25±35.16	5106.21±0.03	5116.66±93.20	4493.70±157.40	4834.56±26.71	4761.25±57.71	4648.61±55.32	4342.46±21.45
**C18:2**	458.66±14.88	443.79±5.78	412.28±18.22	396.60±15.52	432.35±9.39	459.37±12.05	423.24±10.02	415.57±5.45	389.12±12.80	396.30±4.83	353.79±19.47
**C20:0**	42.79±1.67	41.62±0.26	38.39±0.96	36.41±1.70	43.42±5.59	40.25±2.23	39.86±5.84	36.77±1.39	34.52±1.04	34.00±1.47	28.99±0.22
**C18:3**	24.66±0.93	24.33±0.15	21.57±2.05	21.74±1.06	24.33±0.51	25.54±1.17	23.50±0.60	22.23±0.41	21.20±0.76	20.96±0.43	18.26±1.02
**C20:1**	18.27±0.71	17.91±0.12	16.08±1.57	16.08±0.81	17.97±0.29	18.97±0.79	17.62±0.38	16.60±0.34	15.85±0.58	15.84±0.26	13.88±0.76
**C22:0**	7.34±0.40	7.33±0.06	6.47±0.64	6.66±0.40	7.47±0.37	7.73±0.40	7.11±0.16	7.00±0.07	6.44±0.19	6.29±0.19	5.47±0.28
**C24:0**	107.26±0.14	104.79±1.36	94.53±6.26	90.49±3.75	95.58±4.48	109.68±3.90	106.53±6.00	89.71±5.19	85.36±2.60	90.62±0.40	82.20±4.29

Refer to Table S2 caption for abbreviations meaning of the oil samples. C14:0 (Myristic acid), C16:0 (Palmitic acid), C16:1 (n−7) (Palmitoleic acid), C17:0 (Margarinic acid), C17:1 (n−7) (cis-10-Heptadecenoic acid), C18:0 (Stearic acid), C18:1 (n−9) (Oleic acid), C18:2 (n−6) (Linoleic acid), C20:0 (Arachidic acid), C18:3 (n−3) (α-Linolenic acid), C20:1 (n−9) (Gadoleic acid), C22:0 (Behenic acid), C24:0 (Lignoceric acid).

Table S23. Fatty acids content (mg/kg) of supplemented and non-supplemented olive oil 1° with HTyr under deep-frying conditions.

Fatty acid	Control 1	SOO	Control 2	Exp 1	Exp 2	Exp 3	Exp 4	Exp 5	Exp 6	Exp 7	Exp 8
**C14:0**	43.13±2.48	23.12±0.48	24.93±1.03	20.48±0.97	14.31±0.03	18.33±0.08	9.06±0.82	8.17±0.63	5.51±0.15	6.93±0.70	7.19±0.21
**C16:0**	584.83±19.24	551.56±3.38	553.77±16.49	566.65±4.43	565.35±1.18	505.66±0.41	483.89±2.49	525.52±14.47	520.30±6.61	541.04±16.74	543.94±3.83
**C16:1**	39.92±1.37	37.63±0.17	38.40±1.14	39.14±0.89	39.49±0.15	35.23±0.02	34.10±0.00	37.08±0.99	36.63±0.16	38.80±1.08	38.76±0.35
**C17:0**	3.07±0.28	2.83±0.03	2.98±0.32	2.90±0.03	2.64±0.03	2.56±0.07	2.38±0.04	2.74±0.17	2.65±0.10	2.56±0.08	2.47±0.04
**C17:1**	7.37±0.51	9.00±0.19	4.43±0.11	4.60±0.07	4.10±0.06	4.05±0.03	3.79±0.04	4.24±0.16	4.11±0.17	3.99±0.13	3.99±0.07
**C18:0**	350.38±11.65	290.31±3.07	303.52±0.96	277.65±17.71	219.35±1.20	122.76±0.39	147.75±5.94	157.08±6.26	136.51±33.80	137.91±12.79	142.32±2.45
**C18:1**	4279.46±57.24	4196.02±28.88	4217.66±69.70	4284.13±62.03	4286.00±1.24	3887.86±4.21	3680.83±14.88	4212.27±87.60	4175.14±25.23	4209.47±80.91	4274.88±9.12
**C18:2**	407.82±6.46	398.78±2.81	394.68±14.41	400.34±3.55	405.52±0.31	356.74±0.24	337.18±1.21	392.14±7.07	385.85±3.07	400.37±12.31	399.18±4.13
**C20:0**	38.81±0.78	37.16±0.40	36.44±1.11	35.86±0.20	35.46±1.17	31.72±0.02	29.39±0.07	35.33±0.60	34.91±0.19	33.97±1.12	31.83±0.36

**C18:3**	17.92±0.93	16.80±0.10	16.67±0.62	17.49±0.32	17.43±0.12	16.10±0.09	15.19±0.09	16.76±0.38	16.67±0.04	17.02±0.43	16.76±0.33
**C20:1**	12.40±0.62	11.60±0.07	11.38±0.44	12.19±0.27	11.98±0.11	11.36±0.08	10.48±0.02	11.73±0.26	11.70±0.08	11.83±0.25	11.72±0.21
**C22:0**	4.40±0.46	3.94±0.01	3.92±0.15	4.15±0.01	4.02±0.09	3.86±0.06	3.57±0.01	3.72±0.09	3.69±0.12	3.64±0.10	3.60±0.07
**C24:0**	79.89±1.89	68.99±0.38	62.58±0.72	73.57±0.24	67.17±0.09	61.60±1.12	58.35±0.81	76.56±6.54	72.45±0.65	70.31±2.71	64.12±0.68

Refer to Table S2 caption for abbreviations meaning of the oil samples. C14:0 (Myristic acid), C16:0 (Palmitic acid), C16:1 (n−7) (Palmitoleic acid), C17:0 (Margarinic acid), C17:1 (n−7) (cis-10-Heptadecenoic acid), C18:0 (Stearic acid), C18:1 (n−9) (Oleic acid), C18:2 (n−6) (Linoleic acid), C20:0 (Arachidic acid), C18:3 (n−3) (α-Linolenic acid), C20:1 (n−9) (Gadoleic acid), C22:0 (Behenic acid), C24:0 (Lignoceric acid).

Table S24. Fatty acids content (mg/kg) of supplemented and non-supplemented olive oil 0.4° with HTyr under deep-frying conditions.

Fatty acid	Control 1	SOO	Control 2	Exp 1	Exp 2	Exp 3	Exp 4	Exp 5	Exp 6	Exp 7	Exp 8
**C14:0**	8.77±0.58	7.14±0.79	6.51±0.23	6.19±0.28	6.69±1.00	2.62±0.22	4.09±0.16	6.25±0.21	5.21±0.00	3.88±0.13	4.29±0.08
**C16:0**	671.02±29.59	579.33±32.33	596.89±5.63	575.77±15.72	600.36±32.49	577.64±18.68	587.26±4.36	618.94±22.01	544.00±1.53	516.36±12.29	476.36±5.38
**C16:1**	47.84±2.47	38.59±1.95	41.23±0.30	39.07±1.04	40.10±2.05	40.53±0.90	41.18±0.15	42.86±1.29	37.86±0.03	37.44±0.79	34.52±0.59
**C17:0**	3.49±0.12	3.94±0.31	3.56±0.08	3.70±0.08	4.17±0.63	3.22±0.17	3.46±0.07	3.67±0.15	3.27±0.05	2.80±0.12	2.72±0.02
**C17:1**	4.96±0.22	6.16±0.39	5.38±0.10	5.88±0.08	6.18±0.45	5.05±0.08	5.37±0.07	5.91±0.23	5.45±0.01	4.39±0.10	4.27±0.04
**C18:0**	136.67±3.44	126.48±11.85	120.05±1.22	121.52±0.51	114.24±11.49	100.17±2.50	84.76±2.05	118.63±1.46	115.45±2.10	84.64±1.75	91.48±3.58
**C18:1**	4212.31±244.60	3809.32±184.03	3941.82±18.11	3973.73±100.84	3837.32±37.50	4000.67±50.30	4049.42±10.39	4084.75±96.57	4015.20±21.49	3799.66±72.99	3535.01±71.52
**C18:2**	542.60±31.91	413.01±19.96	458.81±1.79	431.48±11.10	414.36±3.18	457.54±5.90	447.63±1.22	483.28±10.81	432.43±2.21	444.45±9.48	407.06±10.32
**C20:0**	33.53±1.69	29.44±1.80	30.64±0.27	30.20±0.78	28.05±0.48	27.53±0.45	25.90±0.23	31.28±0.89	28.58±0.20	26.27±0.70	22.27±0.50
**C18:3**	19.63±0.93	16.61±1.01	17.24±0.22	16.25±0.36	16.36±0.69	16.73±0.46	17.21±0.12	18.27±0.54	15.87±0.22	15.34±0.18	13.86±0.12
**C20:1**	12.25±0.61	11.09±0.64	11.32±0.14	11.05±0.28	11.06±0.44	11.21±0.29	11.46±0.05	12.45±0.40	10.92±0.13	10.25±0.11	9.23±0.12
**C22:0**	3.71±0.16	3.24±0.21	3.29±0.09	3.09±0.05	3.29±0.46	3.10±0.18	3.18±0.04	3.21±0.11	2.86±0.01	2.63±0.09	2.49±0.08
**C24:0**	46.32±2.67	39.77±1.39	43.61±0.24	36.40±0.89	35.63±0.84	36.49±0.49	38.72±0.14	41.11±0.85	36.64±0.14	35.52±0.48	33.30±0.98

Refer to Table S2 caption for abbreviations meaning of the oil samples. C14:0 (Myristic acid), C16:0 (Palmitic acid), C16:1 (n−7) (Palmitoleic acid), C17:0 (Margarinic acid), C17:1 (n−7) (cis-10-Heptadecenoic acid), C18:0 (Stearic acid), C18:1 (n−9) (Oleic acid), C18:2 (n−6) (Linoleic acid), C20:0 (Arachidic acid), C18:3 (n−3) (α-Linolenic acid), C20:1 (n−9) (Gadoleic acid), C22:0 (Behenic acid), C24:0 (Lignoceric acid).

Table S25. Fatty acids content (mg/kg) of sunflower oil under deep-frying conditions.

Fatty acid	Control 1	Exp 1	Exp 2	Exp 3	Exp 4
**C14:0**	ND	ND	ND	ND	ND
**C16:0**	131.45±5.62	125.41±3.50	138.71±0.75	122.25±3.39	122.88±3.38
**C16:1**	3.73±0.16	3.70±0.14	4.52±0.13	3.68±0.03	4.28±0.25
**C17:0**	ND	ND	ND	ND	ND
**C17:1**	ND	ND	ND	ND	ND
**C18:0**	2.66±0.04	1.44±0.04	2.90±0.13	3.21±0.08	2.06±0.13
**C18:1**	1411.84±48.76	1397.48±28.66	1556.40±44.98	1488.16±14.15	1532.73±39.72
**C18:2**	2515.28±89.14	2502.72±57.82	2570.98±81.35	2602.61±10.57	2644.89±63.17
**C20:0**	5.70±0007	5.85±0.16	6.75±0.20	6.22±0.12	6.39±0.17
**C18:3**	4.28±0.05	4.42±0.16	5.05±0.12	4.71±0.08	4.84±0.15
**C20:1**	16.23±0.24	16.41±0.38	17.23±0.38	15.70±0.44	15.69±0.51
**C22:0**	6.48±0.14	8.25±0.28	10.37±0.28	10.93±0.02	12.34±0.30
**C24:0**	5.21±0.14	5.32±0.16	5.58±0.15	5.20±0.09	5.38±0.14

ND: Not detected. Refer to Table 2 caption for abbreviations meaning of the oil samples.

For sunflower oil categories: Control refers to original, non-deep-fried oil. Exp.1: oil deep fried at 170 °C for 3 h, Exp.2: oil deep fried at 170 °C for 6 h, Exp.3: oil deep fried at 210 °C for 3 h, Exp.4: oil deep fried at 210 °C for 6 h.

Table S26. Fatty acids content (mg/kg) of sunflower oil-high oleic acid under deep-frying conditions.

Fatty acid	Control 1	Exp 1	Exp 2	Exp 3	Exp 4
**C14:0**	ND	ND	ND	ND	ND
**C16:0**	104.94±0.31	106.06±3.47	112.86±2.36	111.18±2.37	108.05±3.40
**C16:1**	4.67±0.26	4.98±0.12	5.08±0.30	5.12±0.27	5.06±0.22
**C17:0**	ND	ND	ND	ND	ND
**C17:1**	ND	ND	ND	ND	ND
**C18:0**	21.74±0.37	20.07±0.41	15.10±0.63	18.85±0.10	3.80±0.16
**C18:1**	2989.55±41.13	3037.42±68.40	3167.68±38.42	3168.68±99.25	3066.90±31.43
**C18:2**	1620.68±29.23	1575.90±38.65	1648.14±11.11	1638.88±58.18	1644.34±27.81
**C20:0**	7.35±0.03	7.11±0.20	7.54±0.23	8.21±0.13	7.29±0.11
**C18:3**	6.28±0.03	6.05±0.16	6.37±0.12	6.26±0.07	6.37±0.13
**C20:1**	20.37±0.15	19.06±0.60	20.30±0.60	19.50±0.28	19.96±0.44
**C22:0**	10.90±0.33	15.03±0.40	11.13±0.05	13.30±0.64	11.22±0.22
**C24:0**	6.61±0.03	6.29±0.19	6.56±0.16	6.60±0.23	6.80±0.05

ND: not detected. Refer to Table S14 caption for abbreviations meaning of the oil sample.

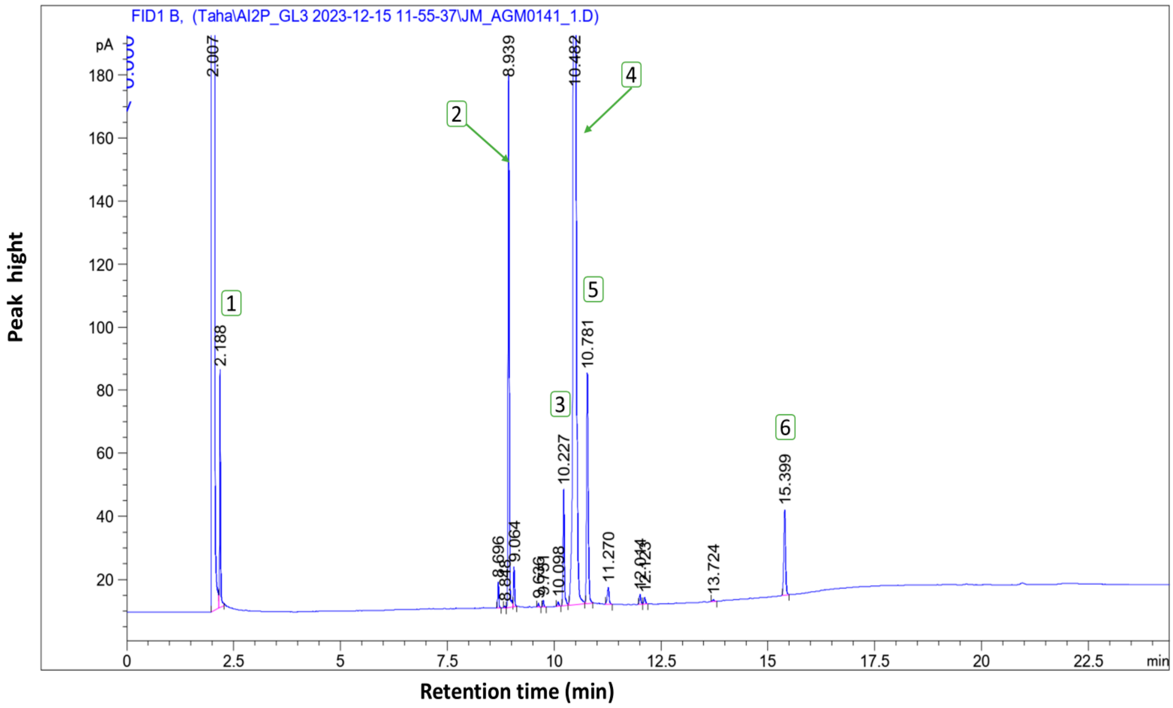

Figure S1. Total ion chromatograms (TICs) from the gas chromatography analysis of the fatty acids in PC_C1: Picual_Control 1. (1: Internal standard (4-Methyl-2-pentanol); 2: Palmitic acid; 3: Stearic acid; 4: Oleic acid; 5: Linoleic acid; 6: Lignoceric acid.

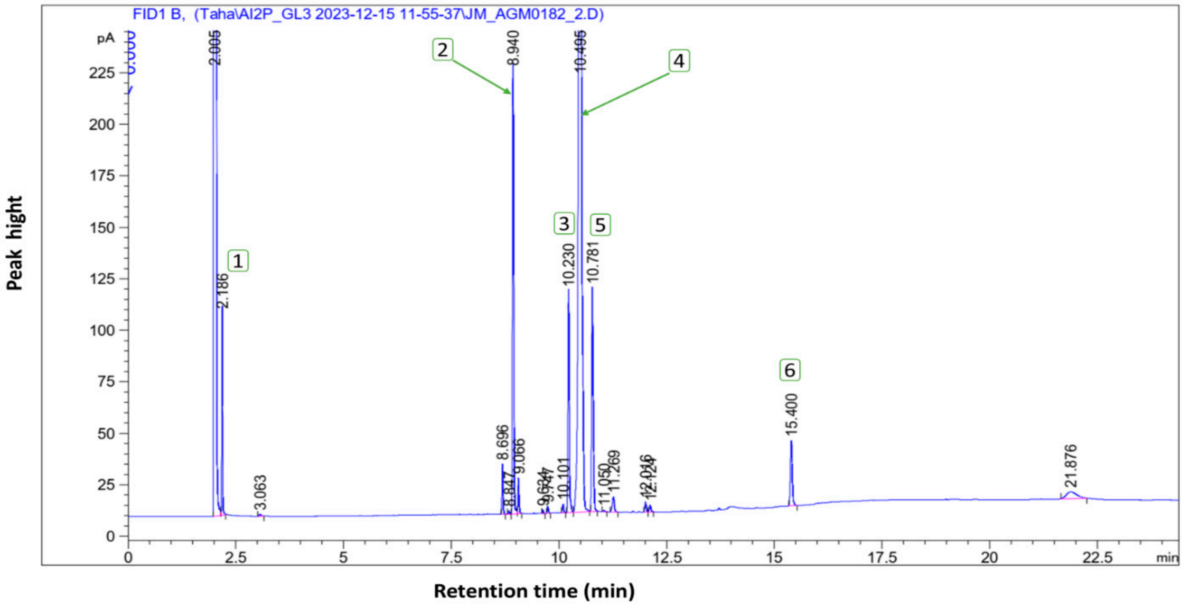

Figure S2. Total ion chromatograms (TICs) from the gas chromatography analysis of the fatty acids in PC_S: Picual_Supplemented. (1: Internal standard (4-Methyl-2-pentanol); 2: Palmitic acid; 3: Stearic acid; 4: Oleic acid; 5: Linoleic acid; 6: Lignoceric acid.

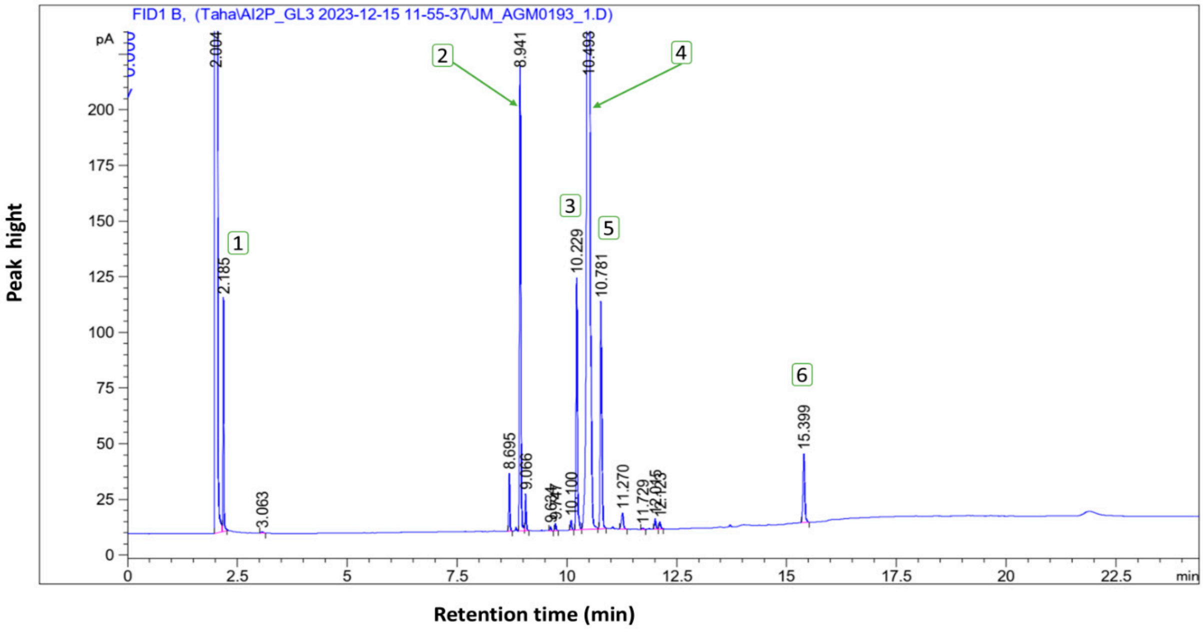

Figure S3. Total ion chromatograms (TICs) from the gas chromatography analysis of the fatty acids in PC_C2: Picual_Control 2. (1: Internal standard (4-Methyl-2-pentanol); 2: Palmitic acid; 3: Stearic acid; 4: Oleic acid; 5: Linoleic acid; 6: Lignoceric acid.

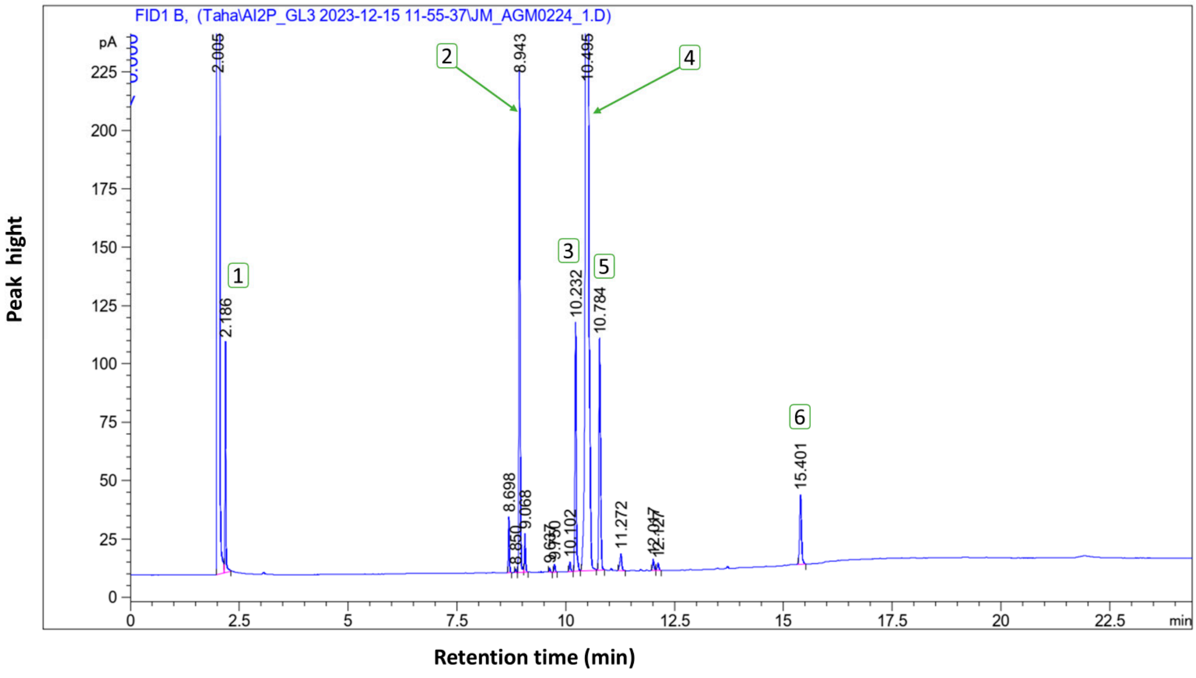

Figure S4. Total ion chromatograms (TICs) from the gas chromatography analysis of the fatty acids in PC_1: Picual_Exp 1. (1: Internal standard (4-Methyl-2-pentanol); 2: Palmitic acid; 3: Stearic acid; 4: Oleic acid; 5: Linoleic acid; 6: Lignoceric acid.

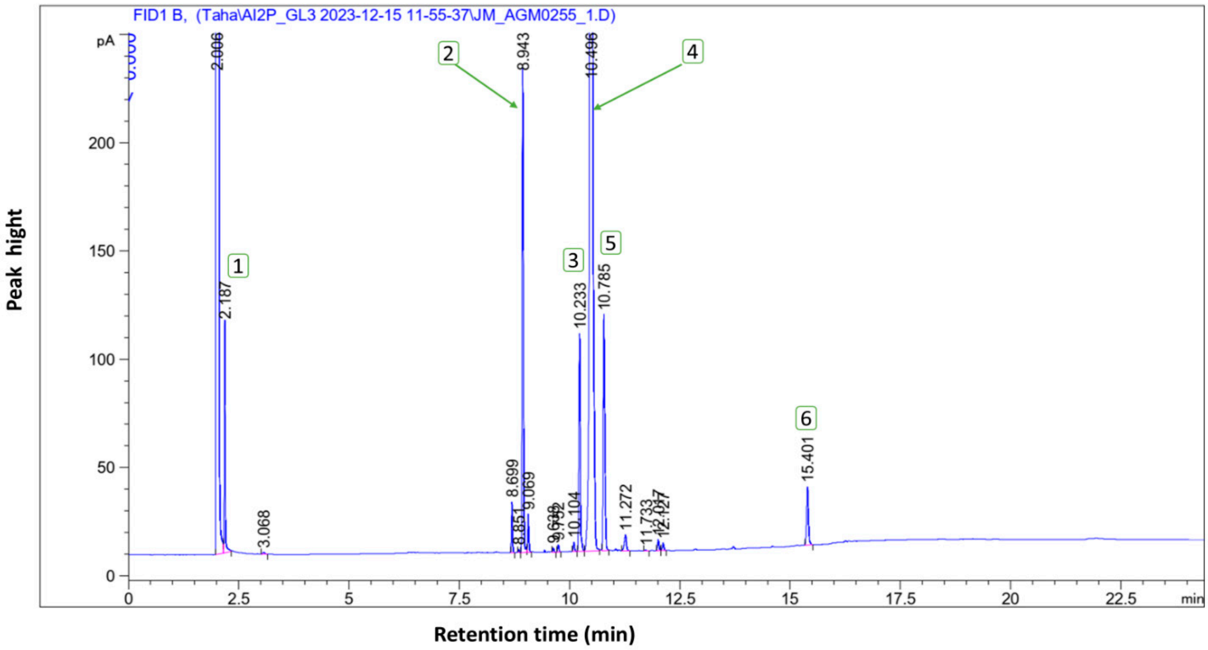

Figure S5. Total ion chromatograms (TICs) from the gas chromatography analysis of the fatty acids in PC_2: Picual_Exp 2. (1: Internal standard (4-Methyl-2-pentanol); 2: Palmitic acid; 3: Stearic acid; 4: Oleic acid; 5: Linoleic acid; 6: Lignoceric acid.

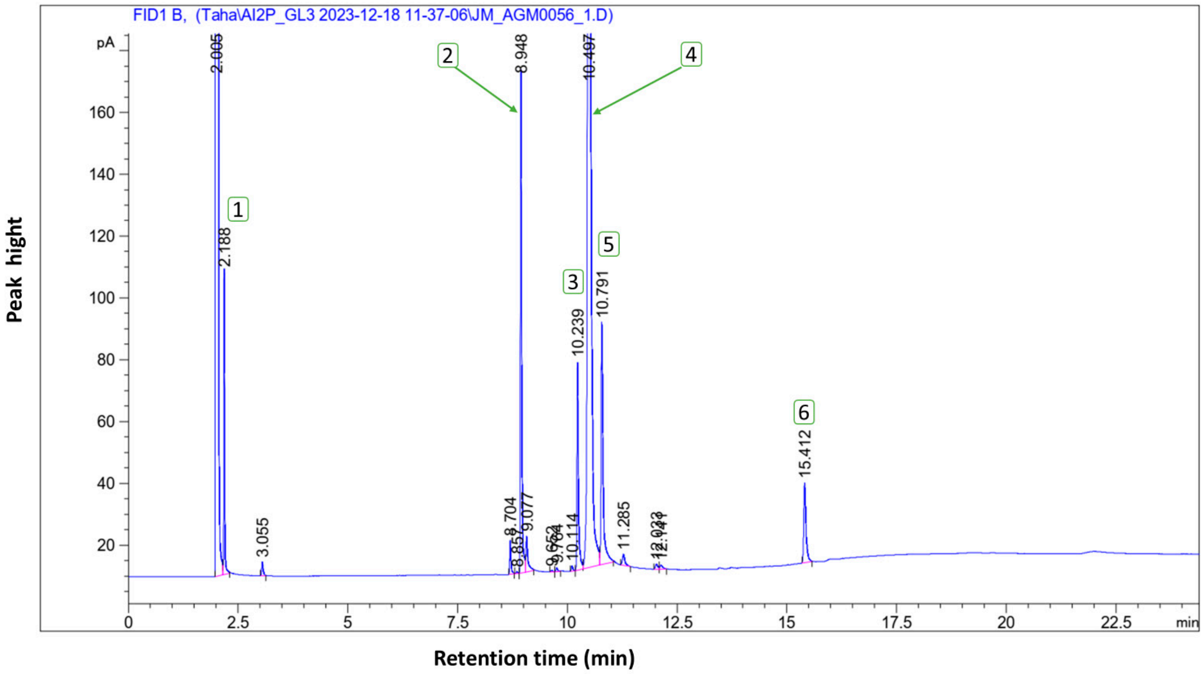

Figure S6. Total ion chromatograms (TICs) from the gas chromatography analysis of the fatty acids in PC_3: Picual_Exp 3. (1: Internal standard (4-Methyl-2-pentanol); 2: Palmitic acid; 3: Stearic acid; 4: Oleic acid; 5: Linoleic acid; 6: Lignoceric acid.

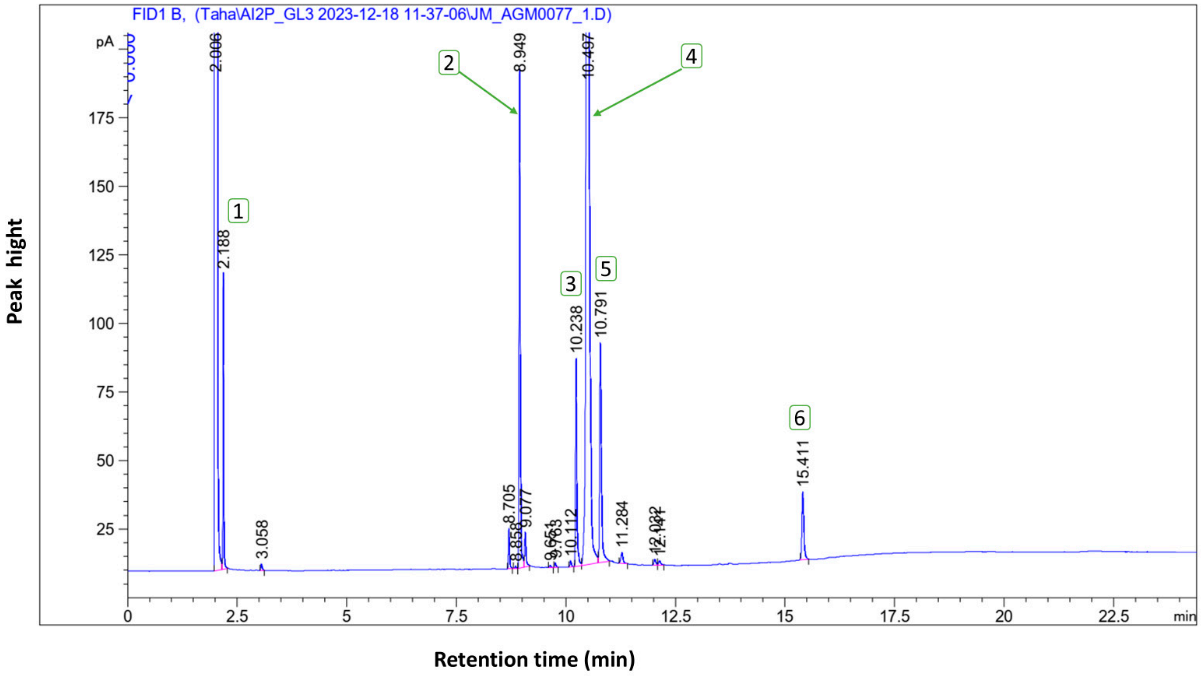

Figure S7. Total ion chromatograms (TICs) from the gas chromatography analysis of the fatty acids in PC_4: Picual_Exp 4. (1: Internal standard (4-Methyl-2-pentanol); 2: Palmitic acid; 3: Stearic acid; 4: Oleic acid; 5: Linoleic acid; 6: Lignoceric acid.

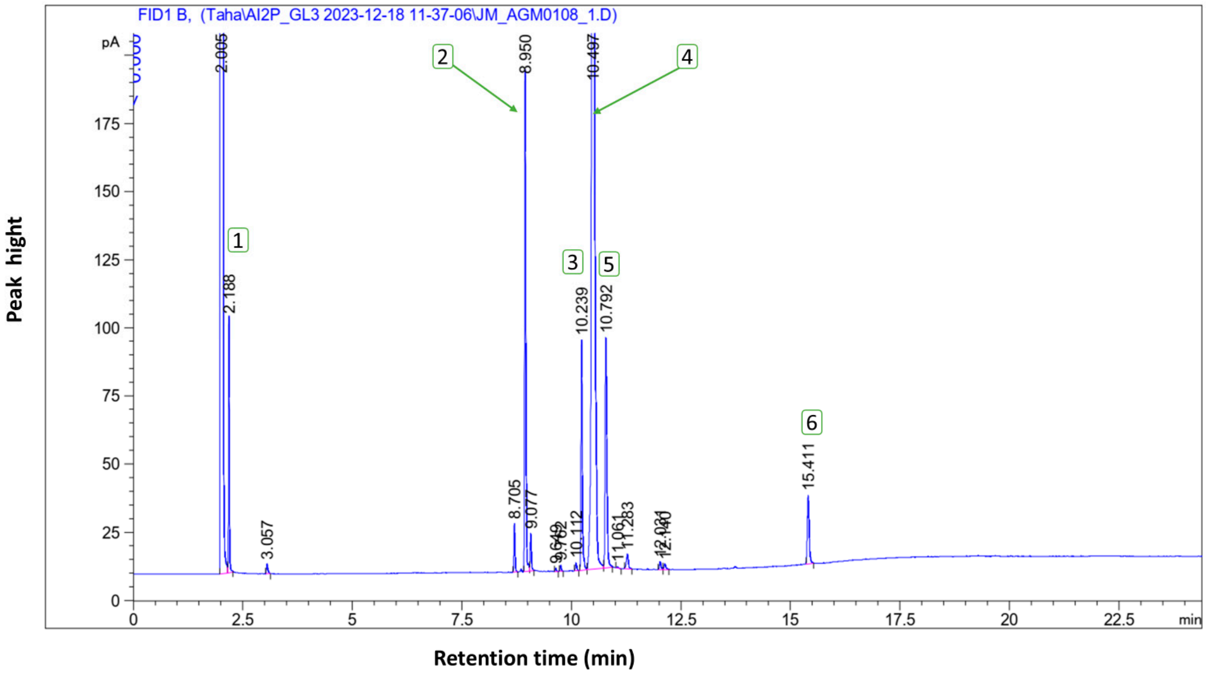

Figure S8. Total ion chromatograms (TICs) from the gas chromatography analysis of the fatty acids in PC_5: Picual_Exp 5. (1: Internal standard (4-Methyl-2-pentanol); 2: Palmitic acid; 3: Stearic acid; 4: Oleic acid; 5: Linoleic acid; 6: Lignoceric acid.

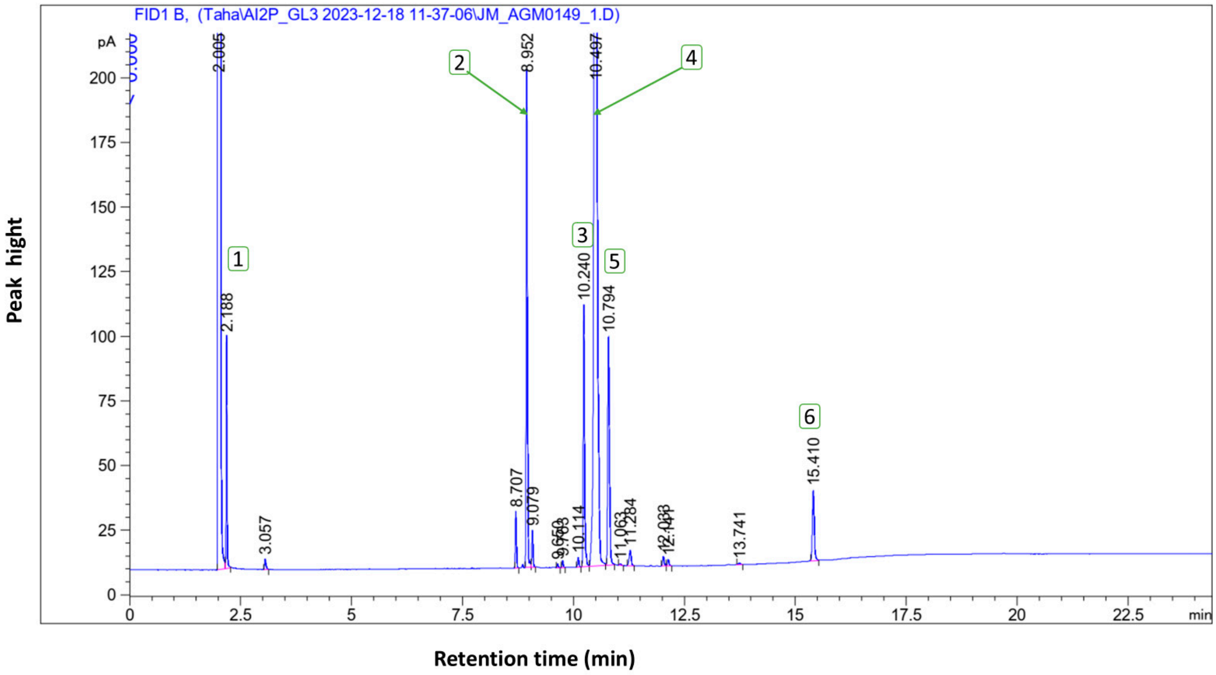

Figure S9. Total ion chromatograms (TICs) from the gas chromatography analysis of the fatty acids in PC_6: Picual_Exp 6. (1: Internal standard (4-Methyl-2-pentanol); 2: Palmitic acid; 3: Stearic acid; 4: Oleic acid; 5: Linoleic acid; 6: Lignoceric acid.

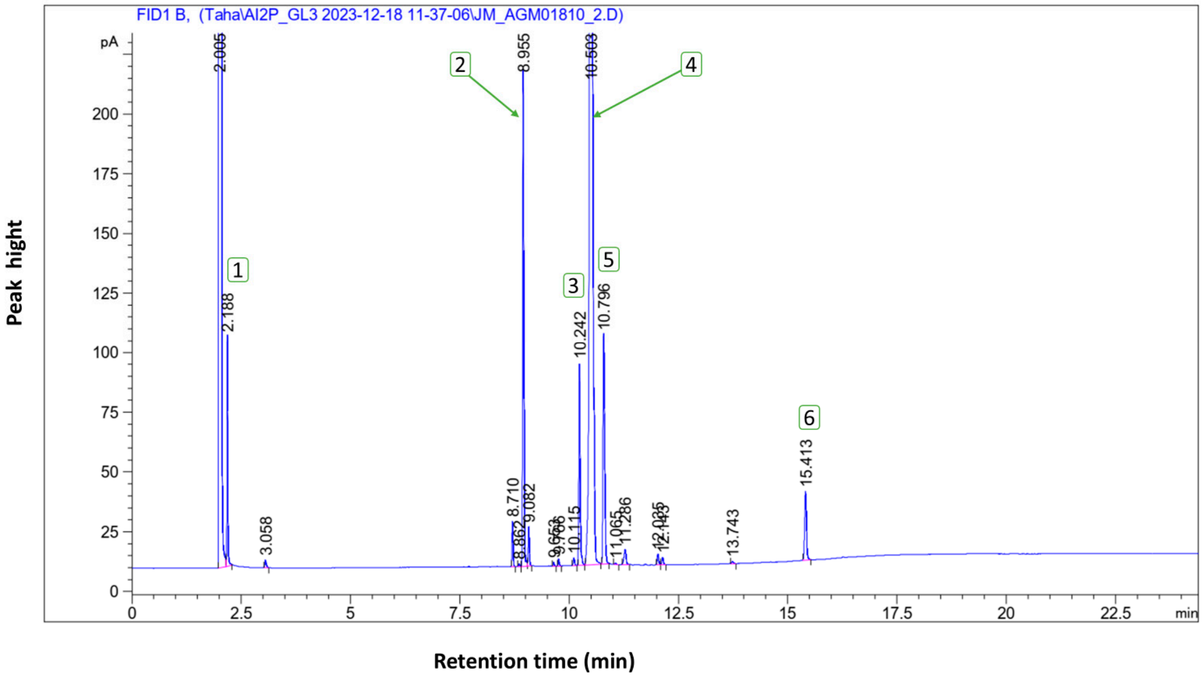

Figure S10. Total ion chromatograms (TICs) from the gas chromatography analysis of the fatty acids in PC_7: Picual_Exp 7. (1: Internal standard (4-Methyl-2-pentanol); 2: Palmitic acid; 3: Stearic acid; 4: Oleic acid; 5: Linoleic acid; 6: Lignoceric acid.

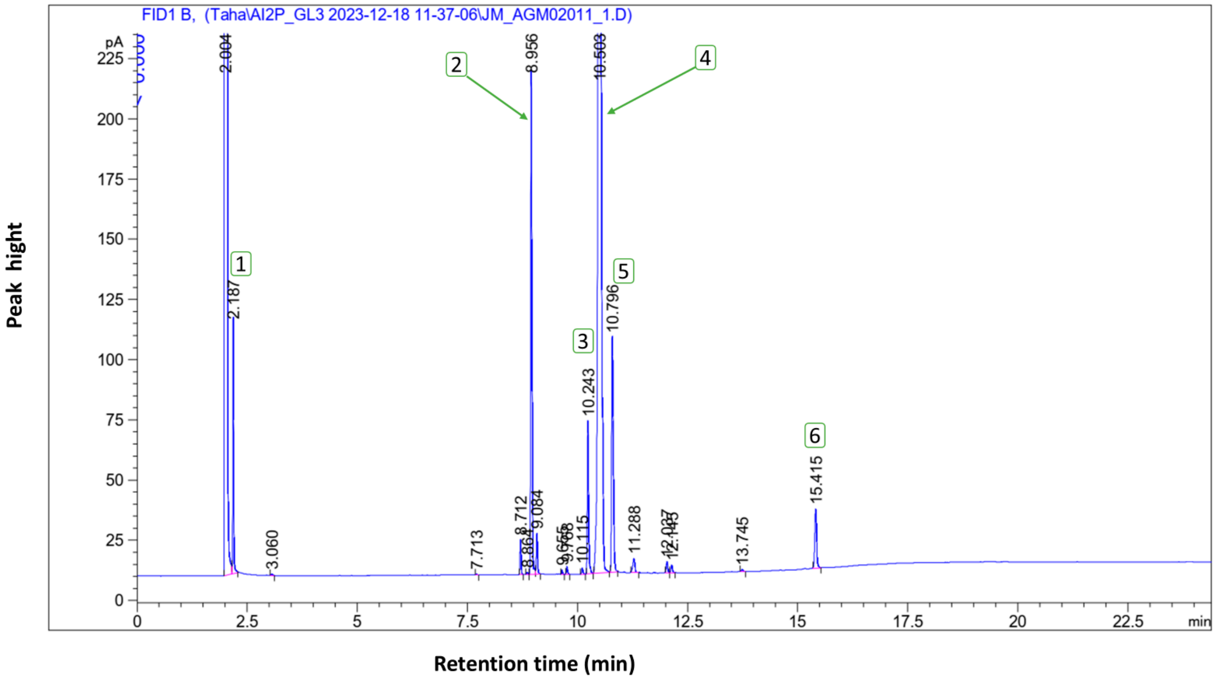

Figure S11. Total ion chromatograms (TICs) from the gas chromatography analysis of the fatty acids in PC_8: Picual_Exp 8. (1: Internal standard (4-Methyl-2-pentanol); 2: Palmitic acid; 3: Stearic acid; 4: Oleic acid; 5: Linoleic acid; 6: Lignoceric acid.

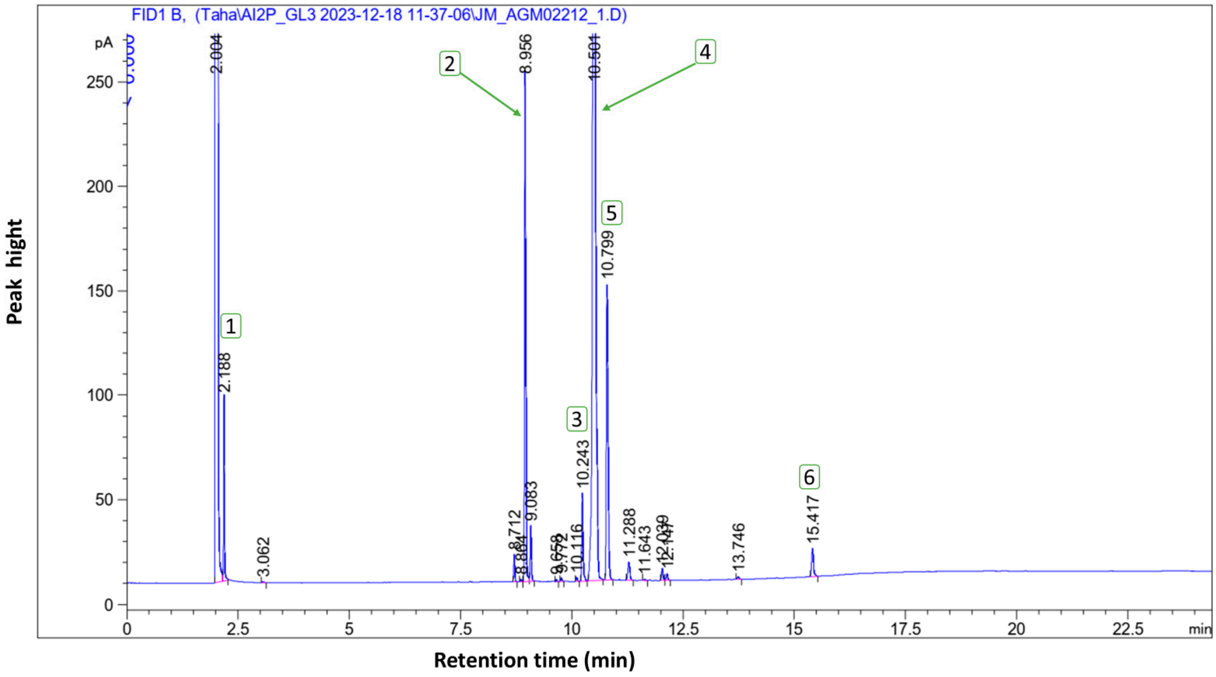

Figure S12. Total ion chromatograms (TICs) from the gas chromatography analysis of the fatty acids in CC_C1: Cornicabra_Control 1. (1: Internal standard (4-Methyl-2-pentanol); 2: Palmitic acid; 3: Stearic acid; 4: Oleic acid; 5: Linoleic acid; 6: Lignoceric acid.

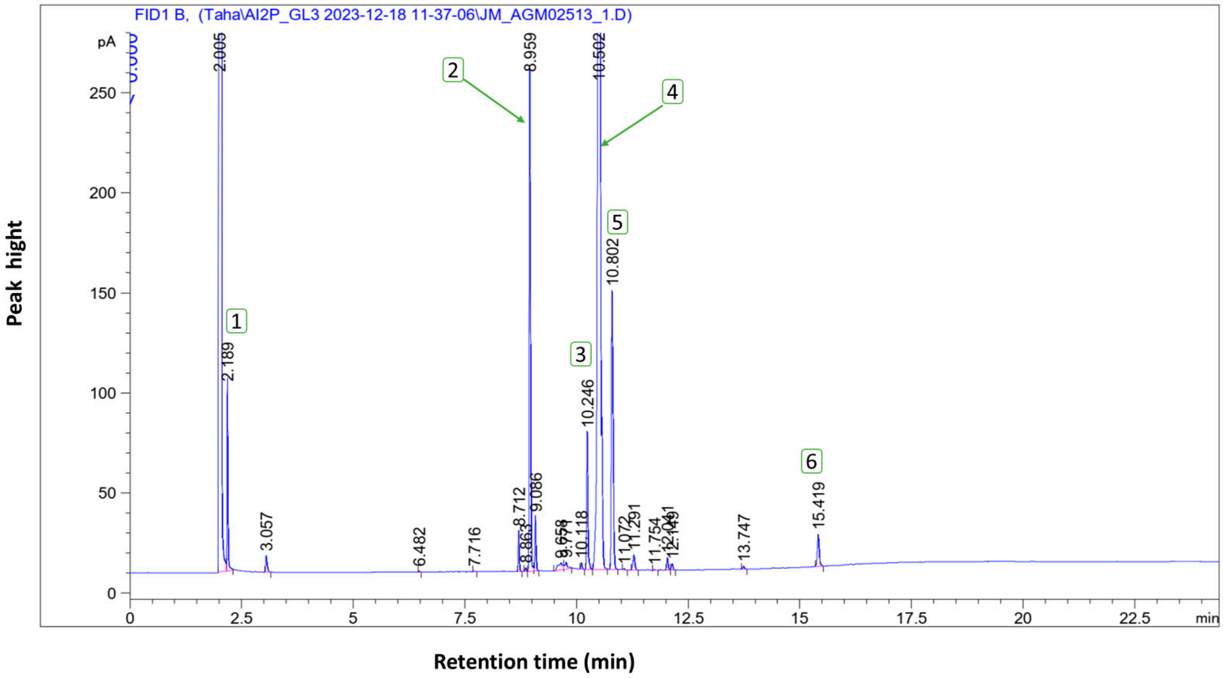

Figure S13. Total ion chromatograms (TICs) from the gas chromatography analysis of the fatty acids in CC_S: Cornicabra_Supplemented. (1: Internal standard (4-Methyl-2-pentanol); 2: Palmitic acid; 3: Stearic acid; 4: Oleic acid; 5: Linoleic acid; 6: Lignoceric acid.

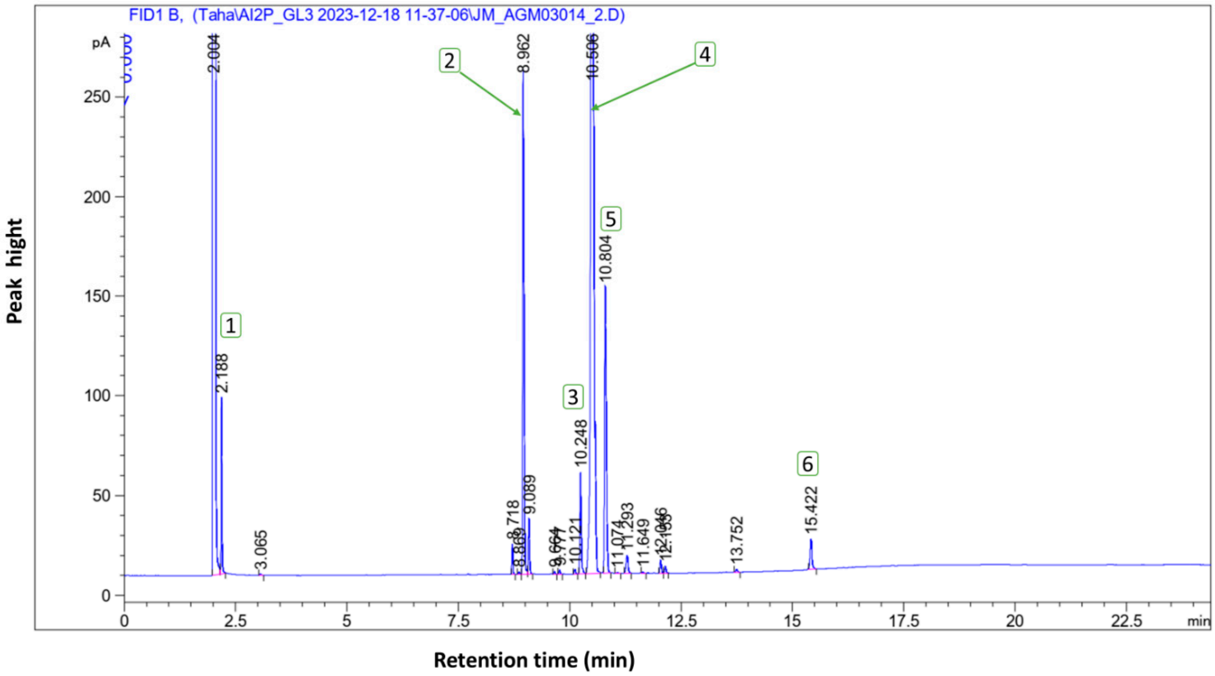

Figure S14. Total ion chromatograms (TICs) from the gas chromatography analysis of the fatty acids in CC_C2: Cornicabra_Control 2. (1: Internal standard (4-Methyl-2-pentanol); 2: Palmitic acid; 3: Stearic acid; 4: Oleic acid; 5: Linoleic acid; 6: Lignoceric acid.

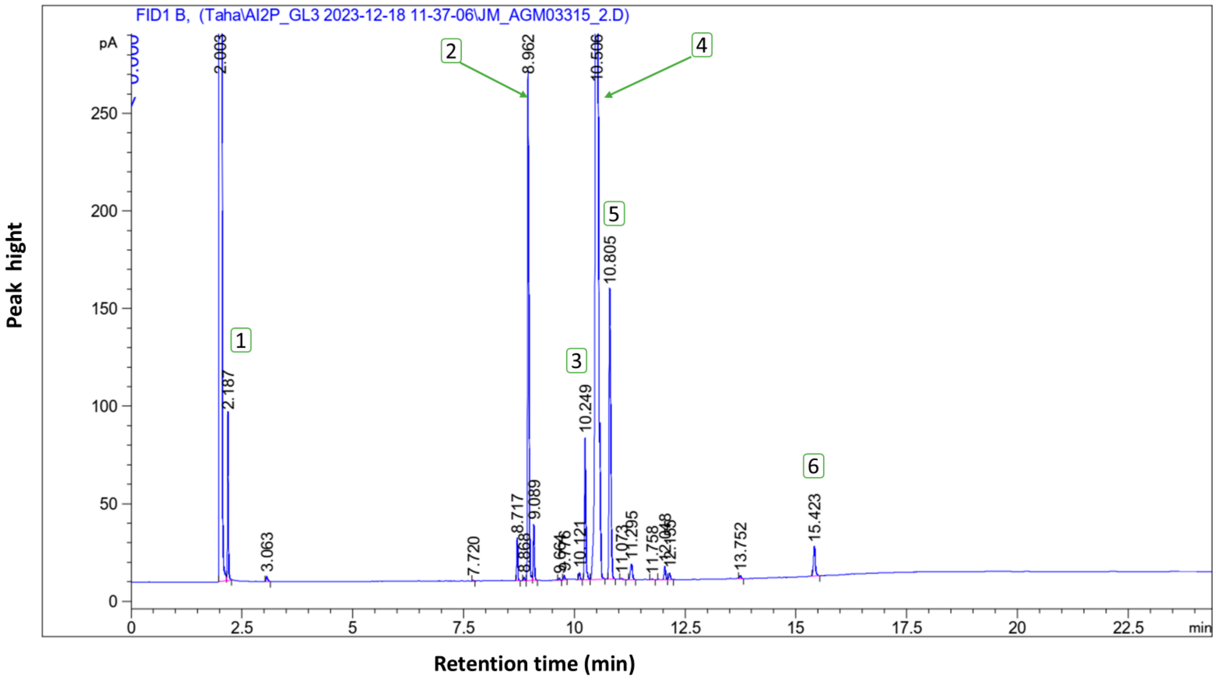

Figure S15. Total ion chromatograms (TICs) from the gas chromatography analysis of the fatty acids in CC_1: Cornicabra_Exp 1. (1: Internal standard (4-Methyl-2-pentanol); 2: Palmitic acid; 3: Stearic acid; 4: Oleic acid; 5: Linoleic acid; 6: Lignoceric acid.

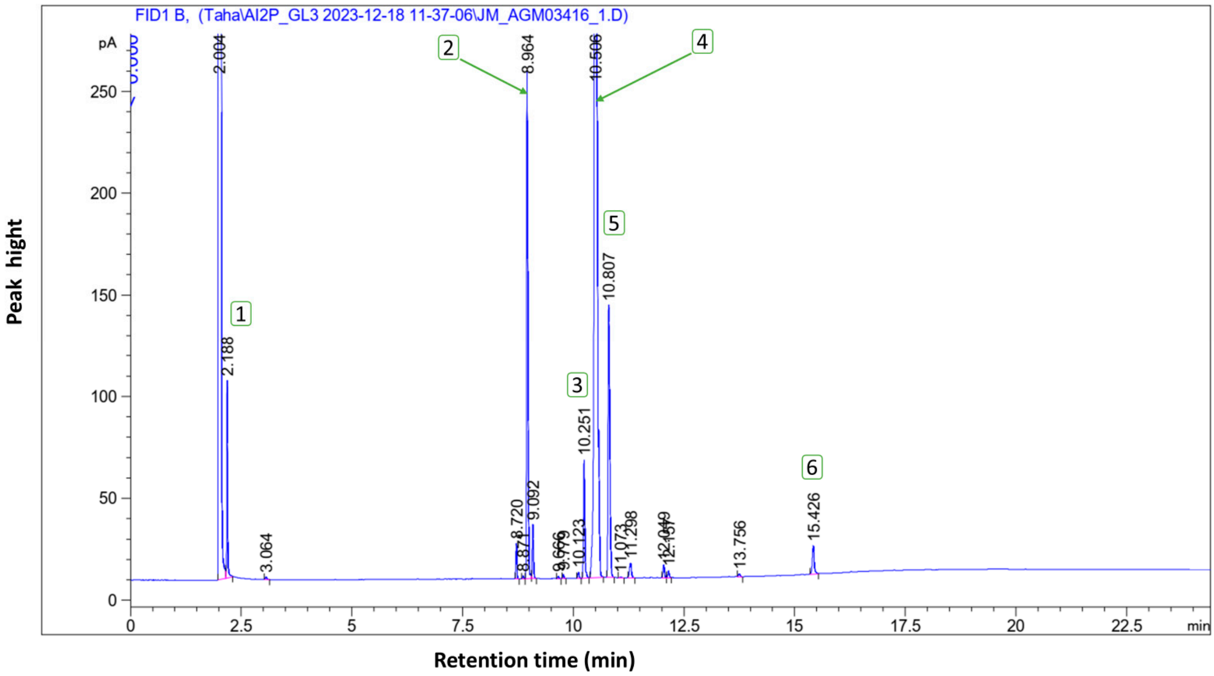

Figure S16. Total ion chromatograms (TICs) from the gas chromatography analysis of the fatty acids in CC_2: Cornicabra_Exp 2. (1: Internal standard (4-Methyl-2-pentanol); 2: Palmitic acid; 3: Stearic acid; 4: Oleic acid; 5: Linoleic acid; 6: Lignoceric acid.

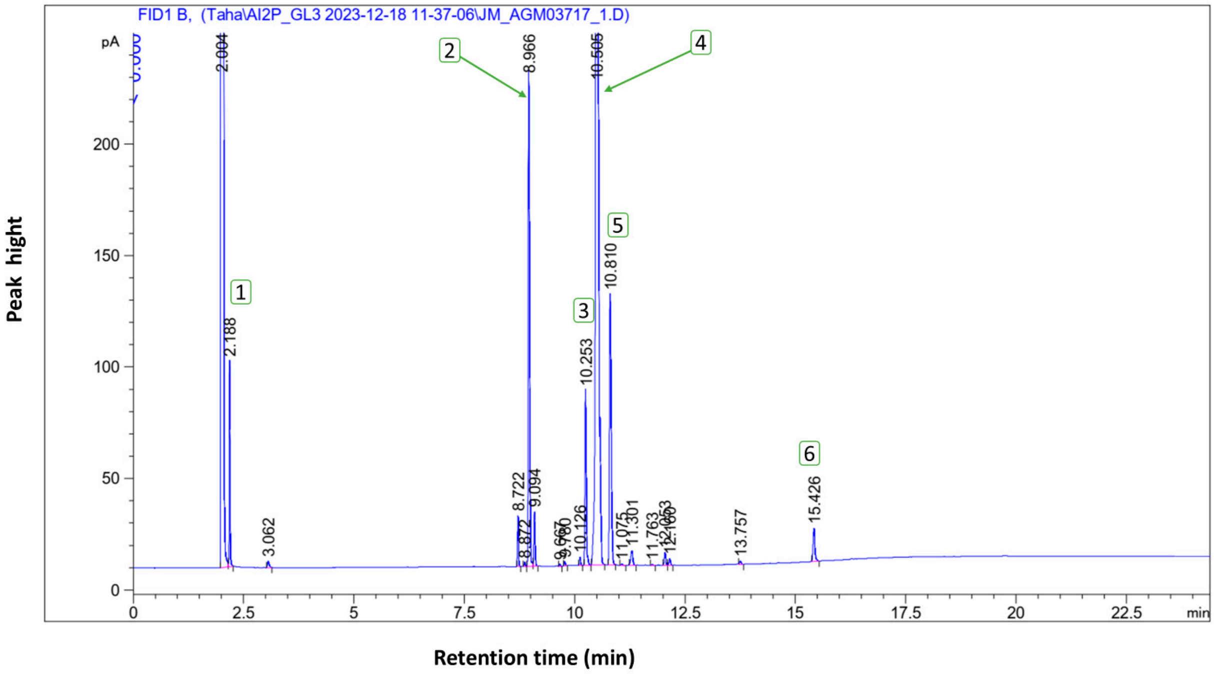

Figure S17. Total ion chromatograms (TICs) from the gas chromatography analysis of the fatty acids in CC_3: Cornicabra_Exp 3. (1: Internal standard (4-Methyl-2-pentanol); 2: Palmitic acid; 3: Stearic acid; 4: Oleic acid; 5: Linoleic acid; 6: Lignoceric acid.

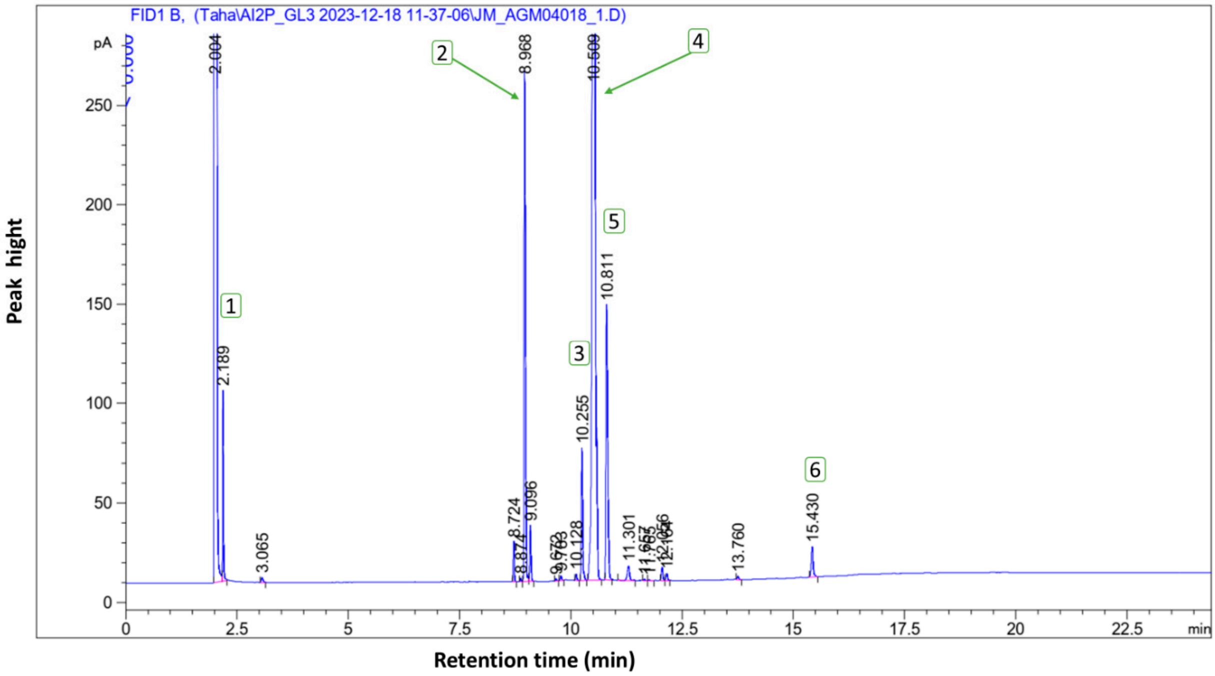

Figure S18. Total ion chromatograms (TICs) from the gas chromatography analysis of the fatty acids in CC_4: Cornicabra_Exp 4. (1: Internal standard (4-Methyl-2-pentanol); 2: Palmitic acid; 3: Stearic acid; 4: Oleic acid; 5: Linoleic acid; 6: Lignoceric acid.

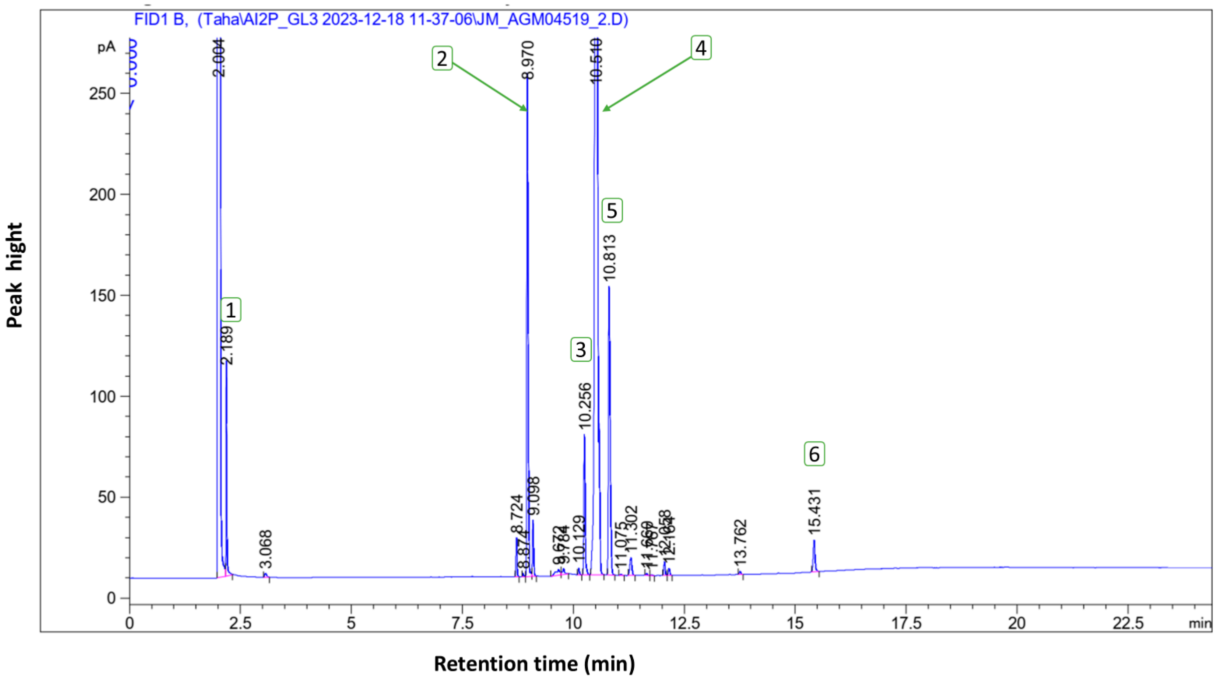

Figure S19. Total ion chromatograms (TICs) from the gas chromatography analysis of the fatty acids in CC_5: Cornicabra_Exp 5. (1: Internal standard (4-Methyl-2-pentanol); 2: Palmitic acid; 3: Stearic acid; 4: Oleic acid; 5: Linoleic acid; 6: Lignoceric acid.

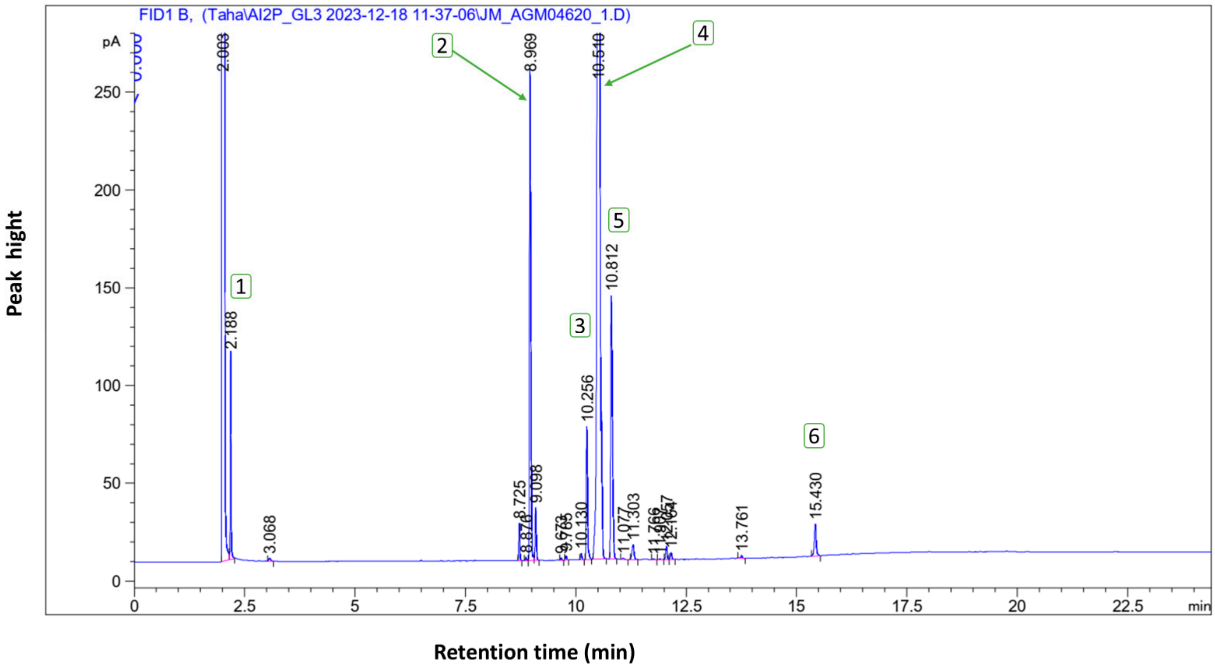

Figure S20. Total ion chromatograms (TICs) from the gas chromatography analysis of the fatty acids in CC_6: Cornicabra_Exp 6. (1: Internal standard (4-Methyl-2-pentanol); 2: Palmitic acid; 3: Stearic acid; 4: Oleic acid; 5: Linoleic acid; 6: Lignoceric acid.

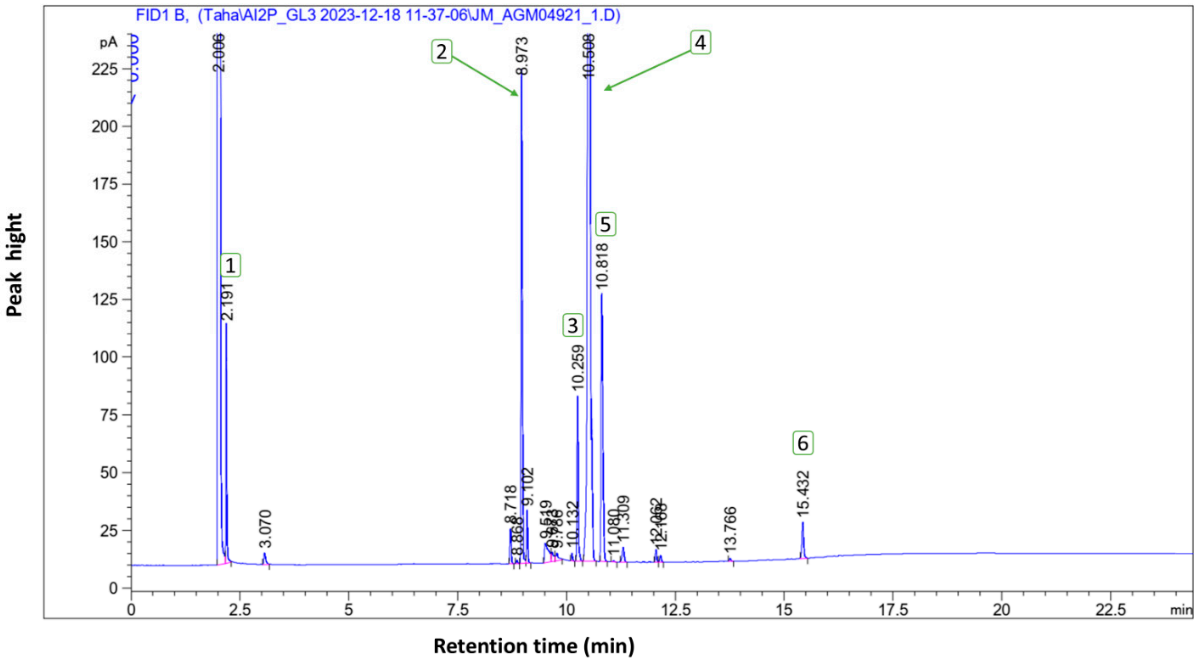

Figure S21. Total ion chromatograms (TICs) from the gas chromatography analysis of the fatty acids in CC_7: Cornicabra_Exp 7. (1: Internal standard (4-Methyl-2-pentanol); 2: Palmitic acid; 3: Stearic acid; 4: Oleic acid; 5: Linoleic acid; 6: Lignoceric acid.

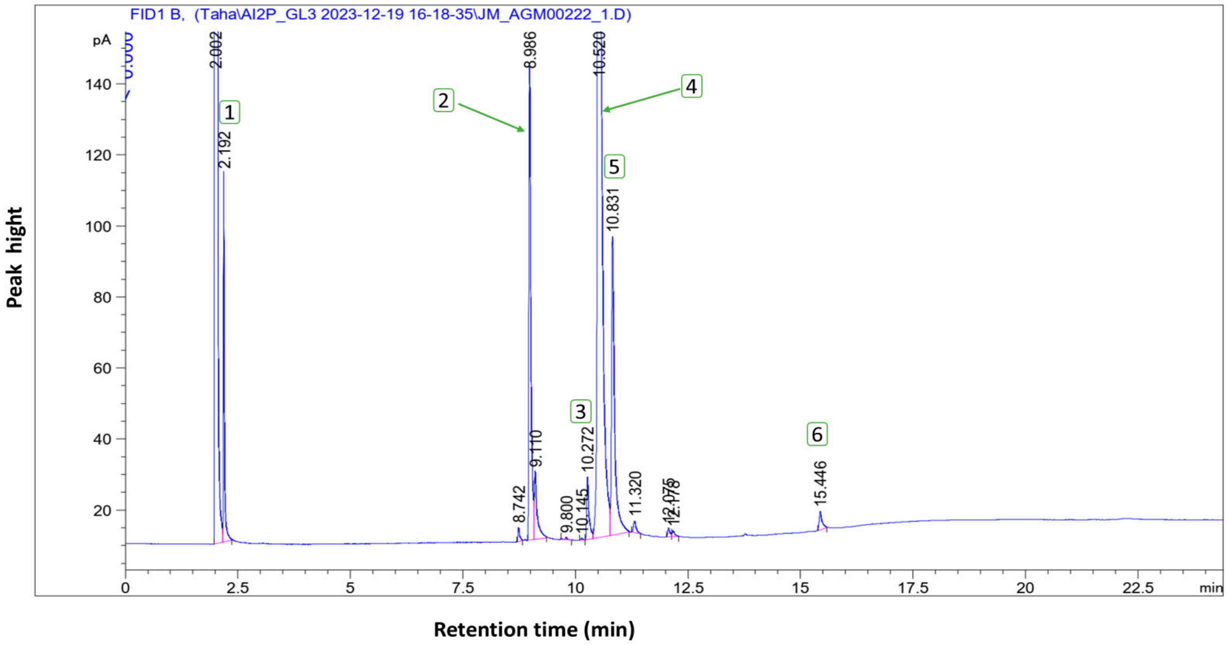

Figure S22. Total ion chromatograms (TICs) from the gas chromatography analysis of the fatty acids in CC_8: Cornicabra_Exp 8. (1: Internal standard (4-Methyl-2-pentanol); 2: Palmitic acid; 3: Stearic acid; 4: Oleic acid; 5: Linoleic acid; 6: Lignoceric acid.

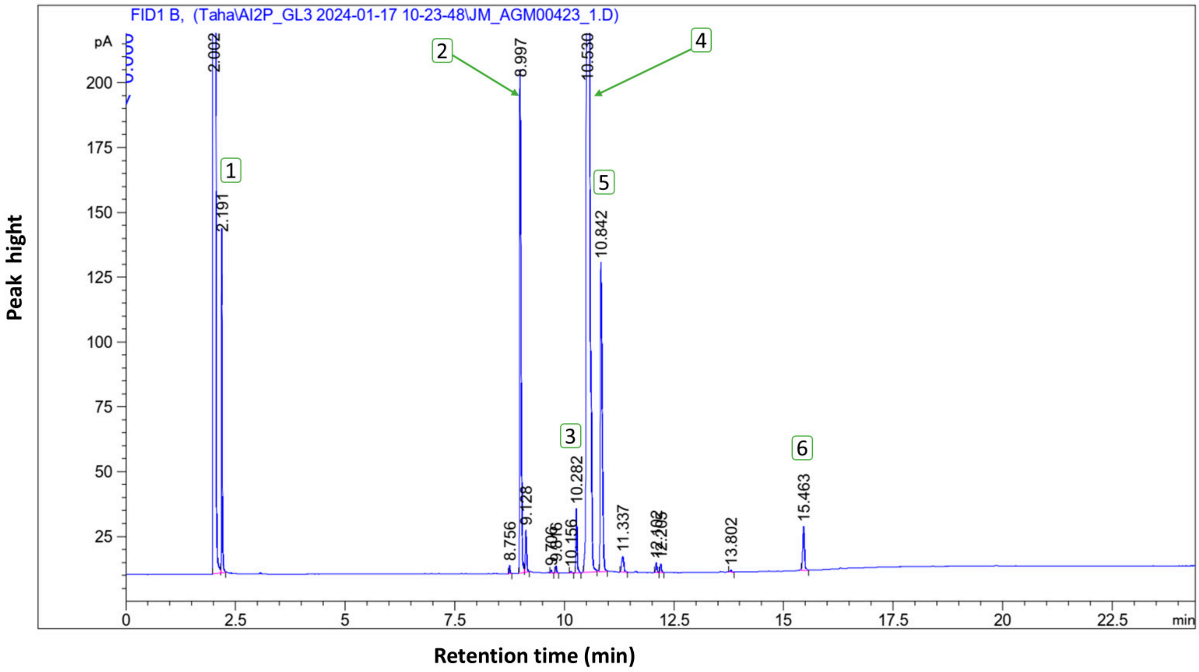

Figure S23. Total ion chromatograms (TICs) from the gas chromatography analysis of the fatty acids in EP_C1: Empeltre_Control 1. (1: Internal standard (4-Methyl-2-pentanol); 2: Palmitic acid; 3: Stearic acid; 4: Oleic acid; 5: Linoleic acid; 6: Lignoceric acid.

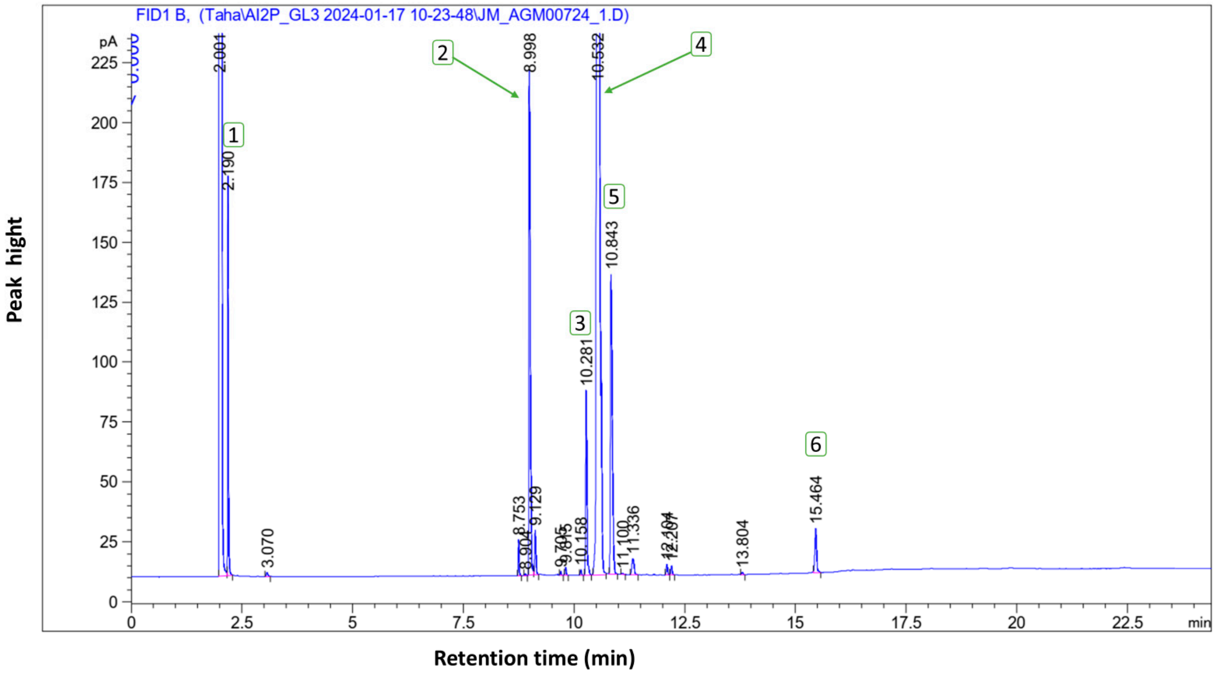

Figure S24. Total ion chromatograms (TICs) from the gas chromatography analysis of the fatty acids in EP_S: Empeltre_Supplemented. (1: Internal standard (4-Methyl-2-pentanol); 2: Palmitic acid; 3: Stearic acid; 4: Oleic acid; 5: Linoleic acid; 6: Lignoceric acid.

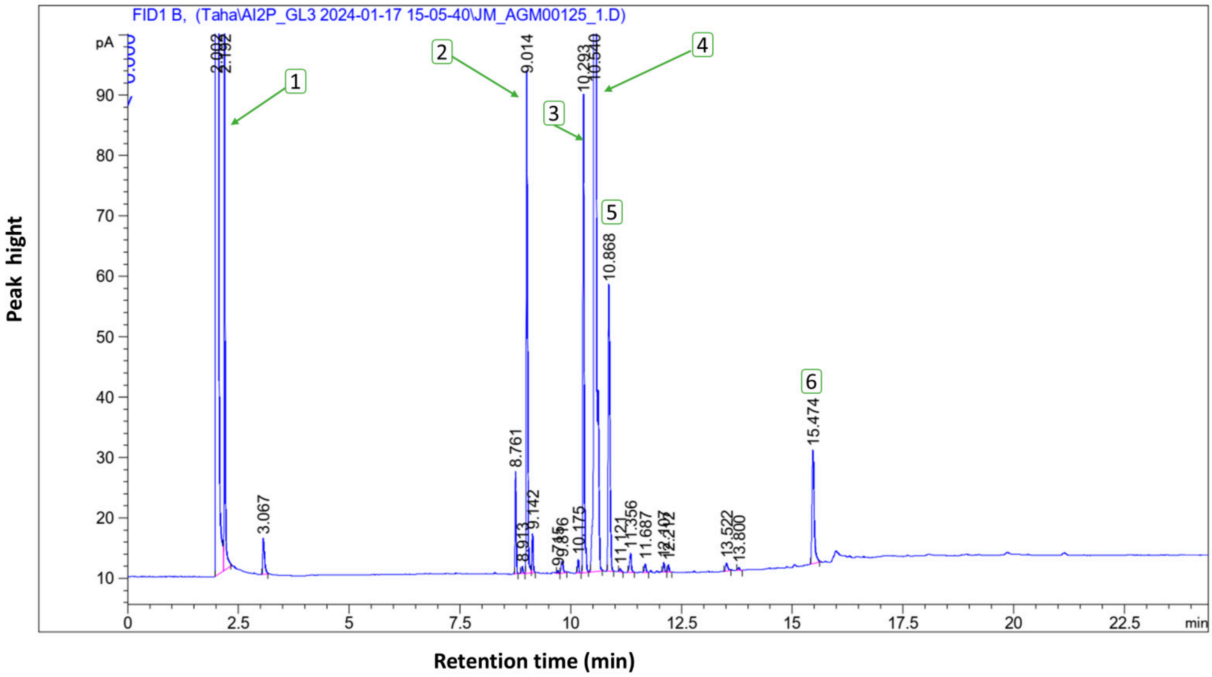

Figure S25. Total ion chromatograms (TICs) from the gas chromatography analysis of the fatty acids in EP_C2: Empeltre_ Control 2. (1: Internal standard (4-Methyl-2-pentanol); 2: Palmitic acid; 3: Stearic acid; 4: Oleic acid; 5: Linoleic acid; 6: Lignoceric acid.

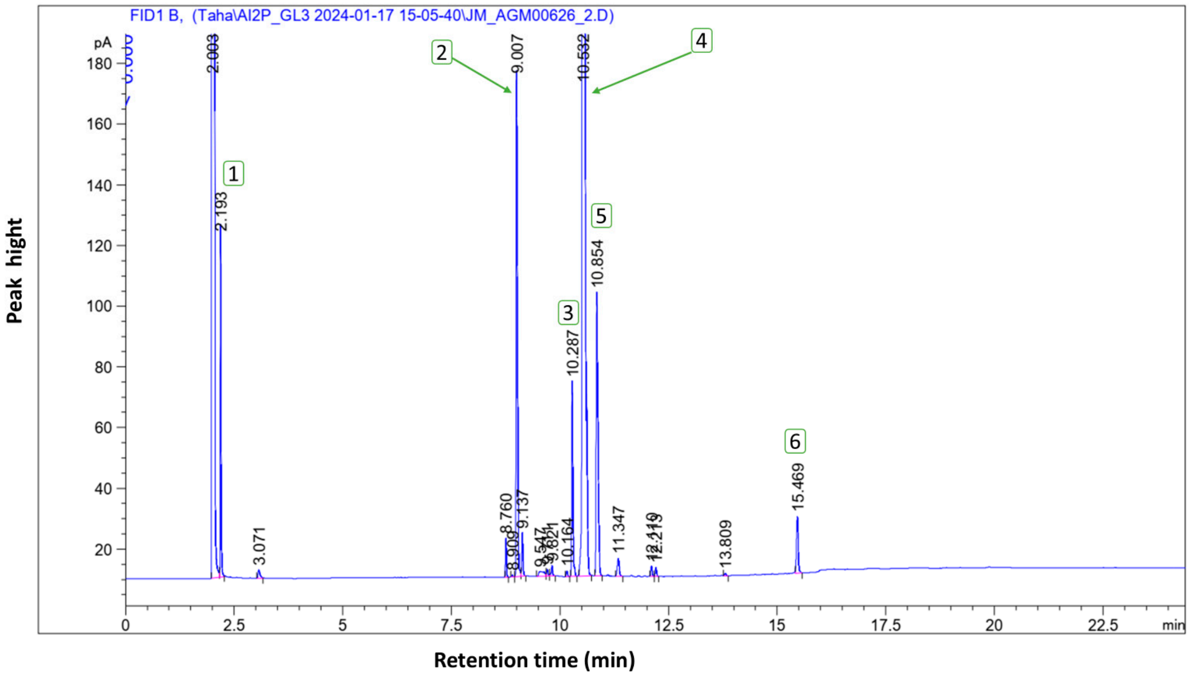

Figure S26. Total ion chromatograms (TICs) from the gas chromatography analysis of the fatty acids in EP_1: Empeltre_Exp 1. (1: Internal standard (4-Methyl-2-pentanol); 2: Palmitic acid; 3: Stearic acid; 4: Oleic acid; 5: Linoleic acid; 6: Lignoceric acid.

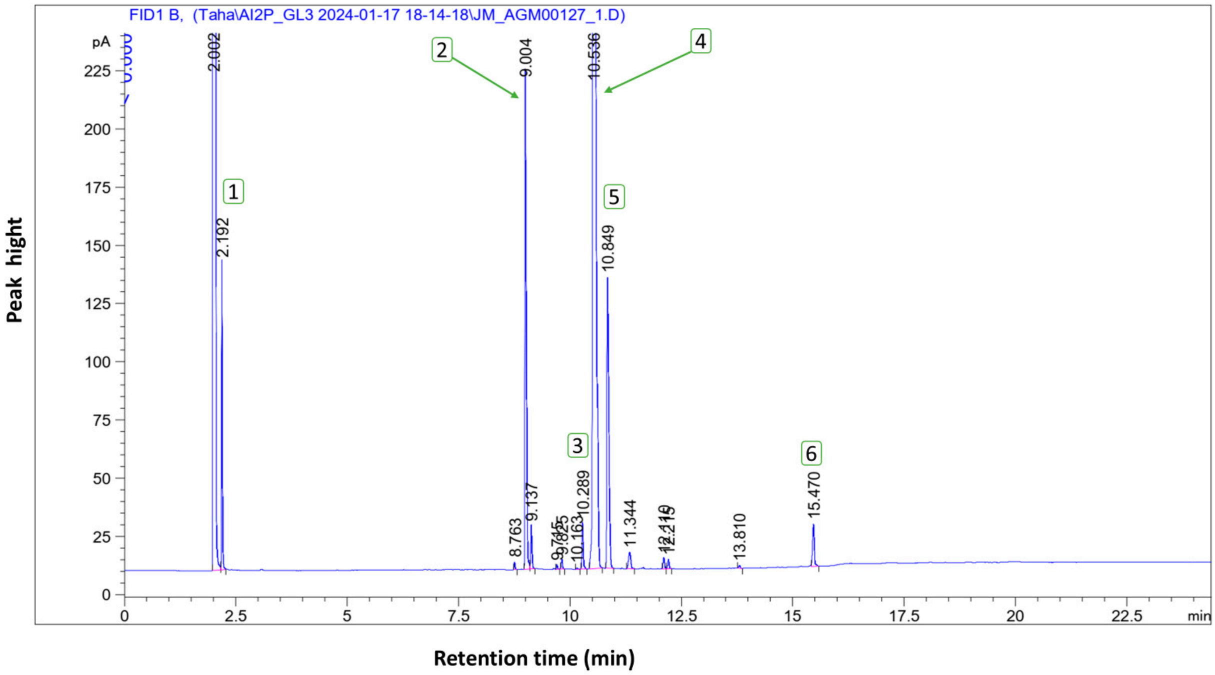

Figure S27. Total ion chromatograms (TICs) from the gas chromatography analysis of the fatty acids in EP_2: Empeltre_Exp 2. (1: Internal standard (4-Methyl-2-pentanol); 2: Palmitic acid; 3: Stearic acid; 4: Oleic acid; 5: Linoleic acid; 6: Lignoceric acid.

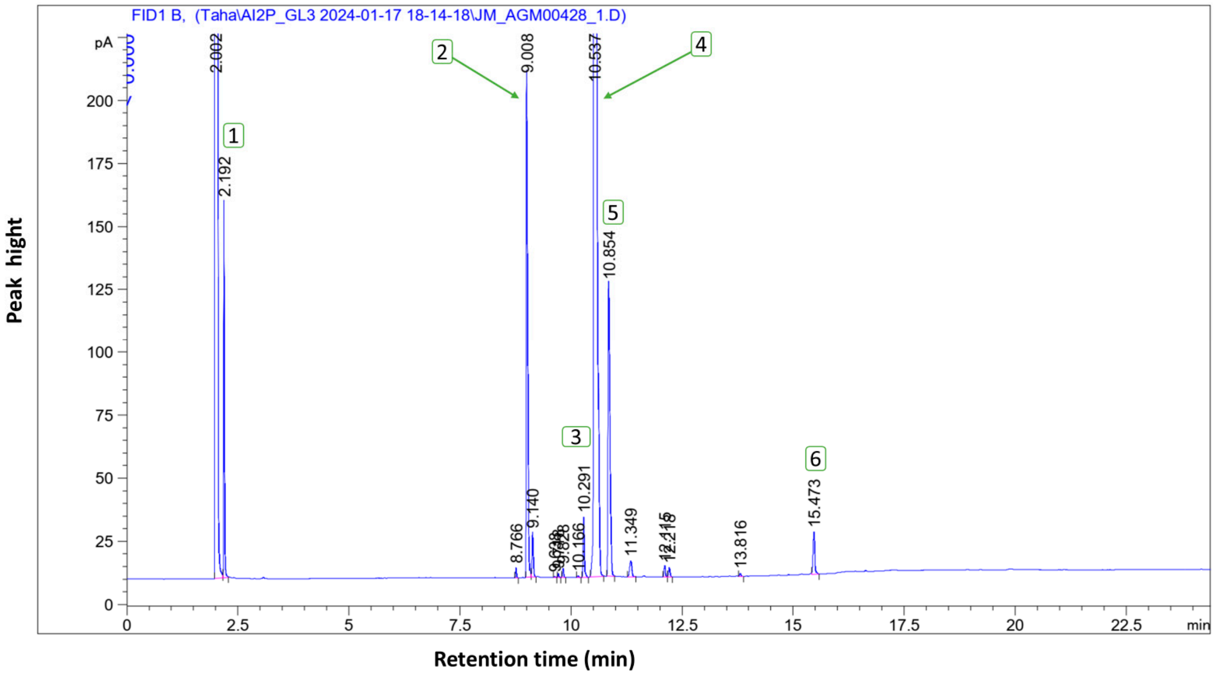

Figure S28. Total ion chromatograms (TICs) from the gas chromatography analysis of the fatty acids in EP_3: Empeltre_Exp 3. (1: Internal standard (4-Methyl-2-pentanol); 2: Palmitic acid; 3: Stearic acid; 4: Oleic acid; 5: Linoleic acid; 6: Lignoceric acid.

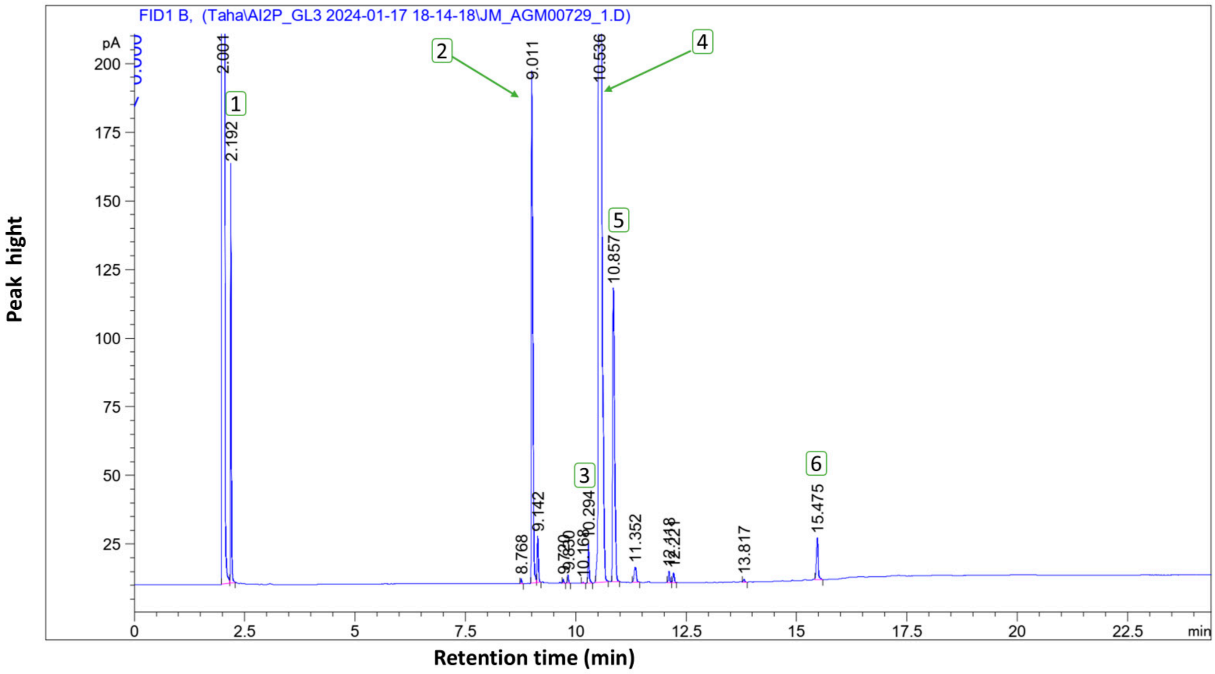

Figure S29. Total ion chromatograms (TICs) from the gas chromatography analysis of the fatty acids in EP_4: Empeltre_Exp 4. (1: Internal standard (4-Methyl-2-pentanol); 2: Palmitic acid; 3: Stearic acid; 4: Oleic acid; 5: Linoleic acid; 6: Lignoceric acid.

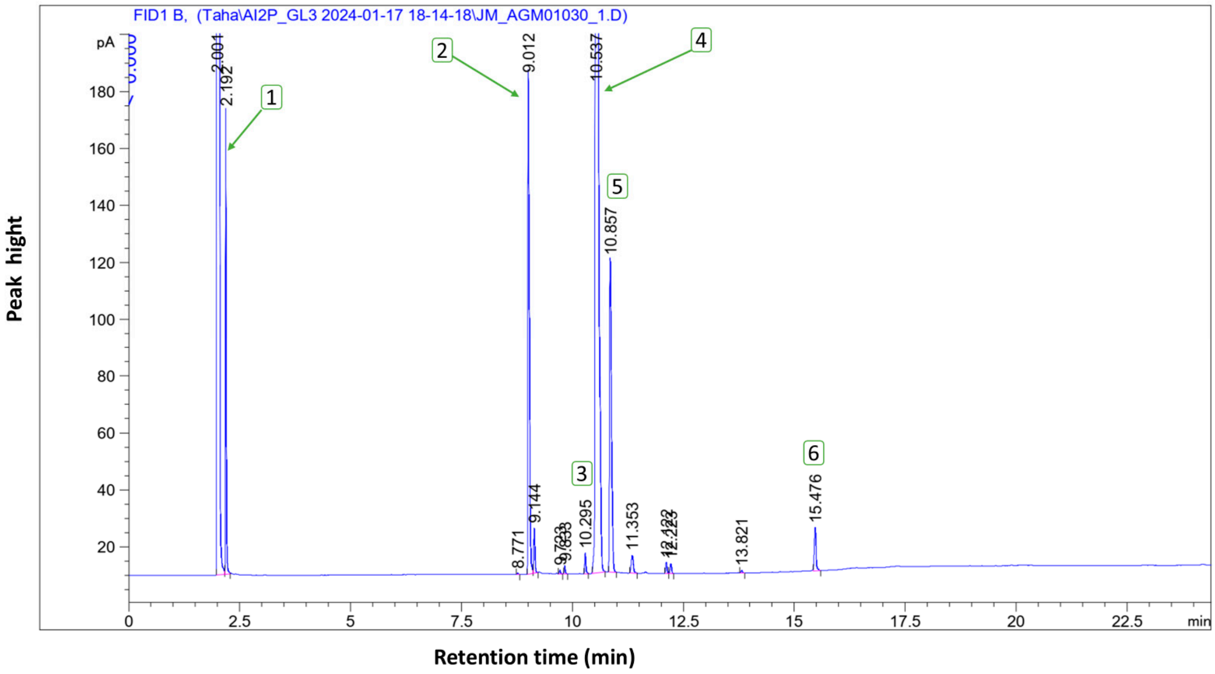

Figure S30. Total ion chromatograms (TICs) from the gas chromatography analysis of the fatty acids in EP_5: Empeltre_Exp 5. (1: Internal standard (4-Methyl-2-pentanol); 2: Palmitic acid; 3: Stearic acid; 4: Oleic acid; 5: Linoleic acid; 6: Lignoceric acid.

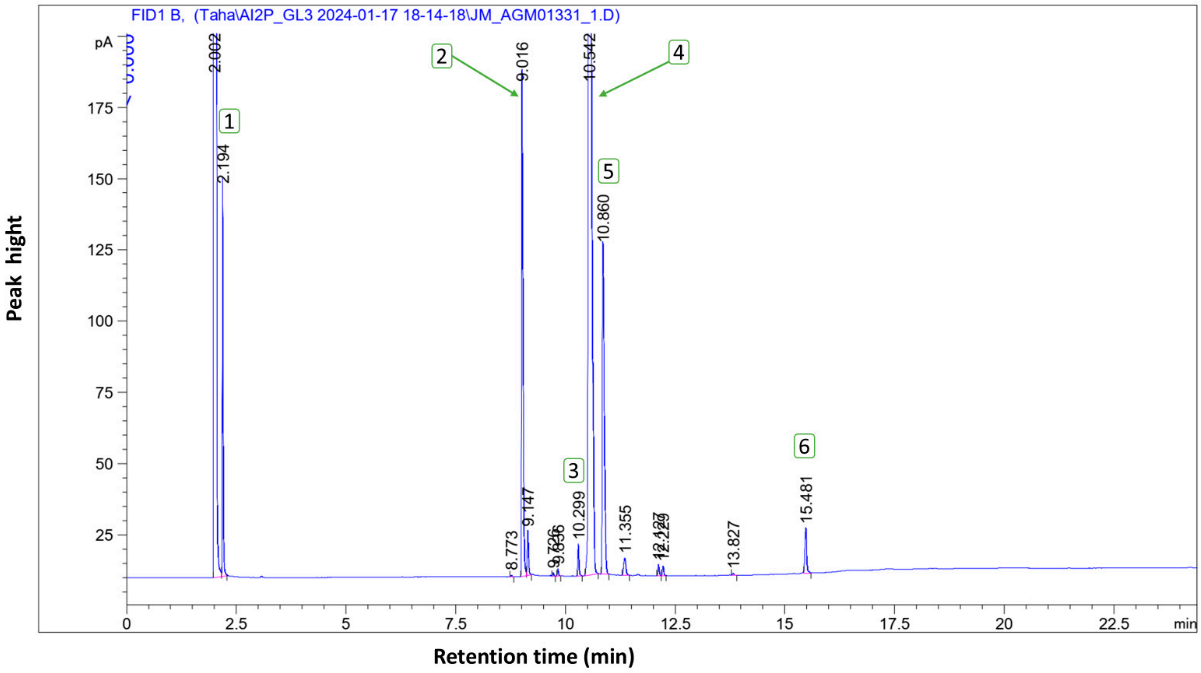

Figure S31. Total ion chromatograms (TICs) from the gas chromatography analysis of the fatty acids in EP_6: Empeltre_Exp 6. (1: Internal standard (4-Methyl-2-pentanol); 2: Palmitic acid; 3: Stearic acid; 4: Oleic acid; 5: Linoleic acid; 6: Lignoceric acid.

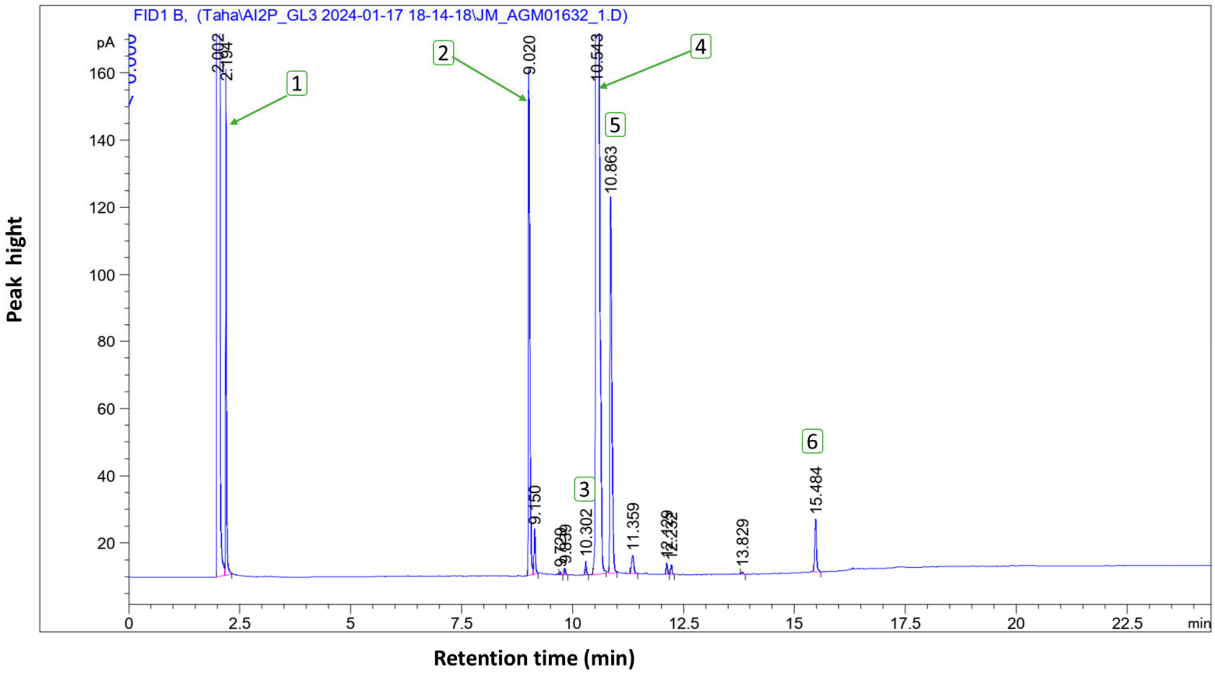

Figure S32. Total ion chromatograms (TICs) from the gas chromatography analysis of the fatty acids in EP_7: Empeltre_Exp 7. (1: Internal standard (4-Methyl-2-pentanol); 2: Palmitic acid; 3: Stearic acid; 4: Oleic acid; 5: Linoleic acid; 6: Lignoceric acid.

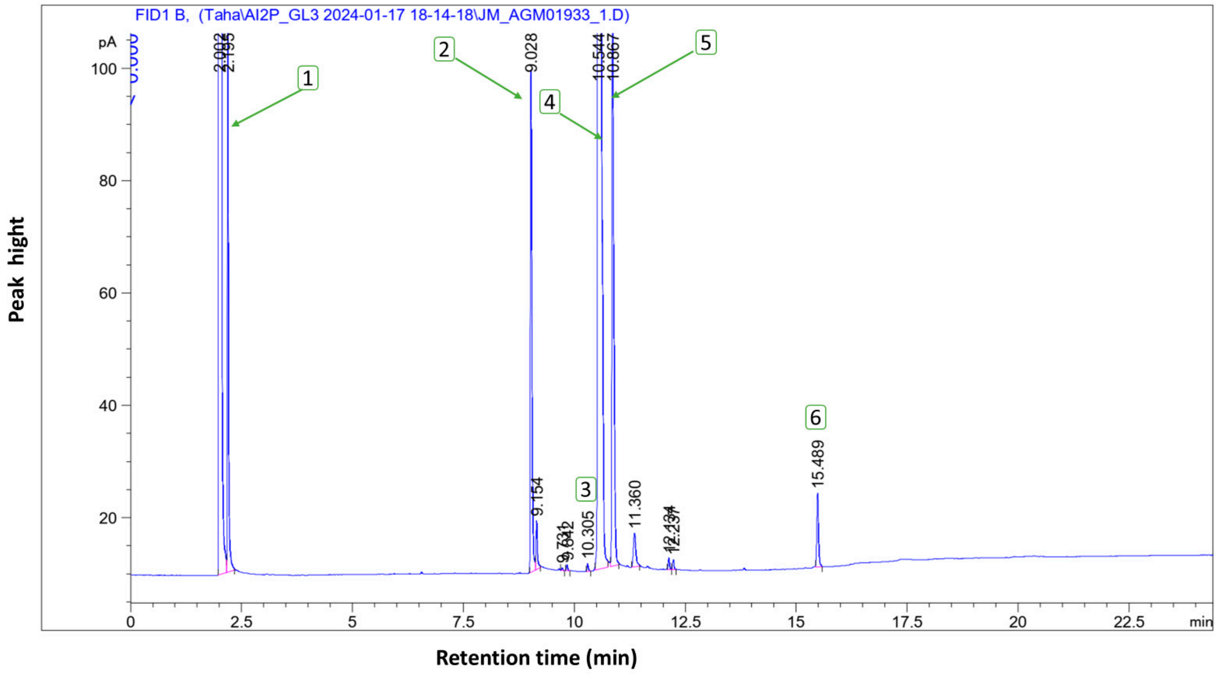

Figure S33. Total ion chromatograms (TICs) from the gas chromatography analysis of the fatty acids in EP_8: Empeltre_Exp 8. (1: Internal standard (4-Methyl-2-pentanol); 2: Palmitic acid; 3: Stearic acid; 4: Oleic acid; 5: Linoleic acid; 6: Lignoceric acid.

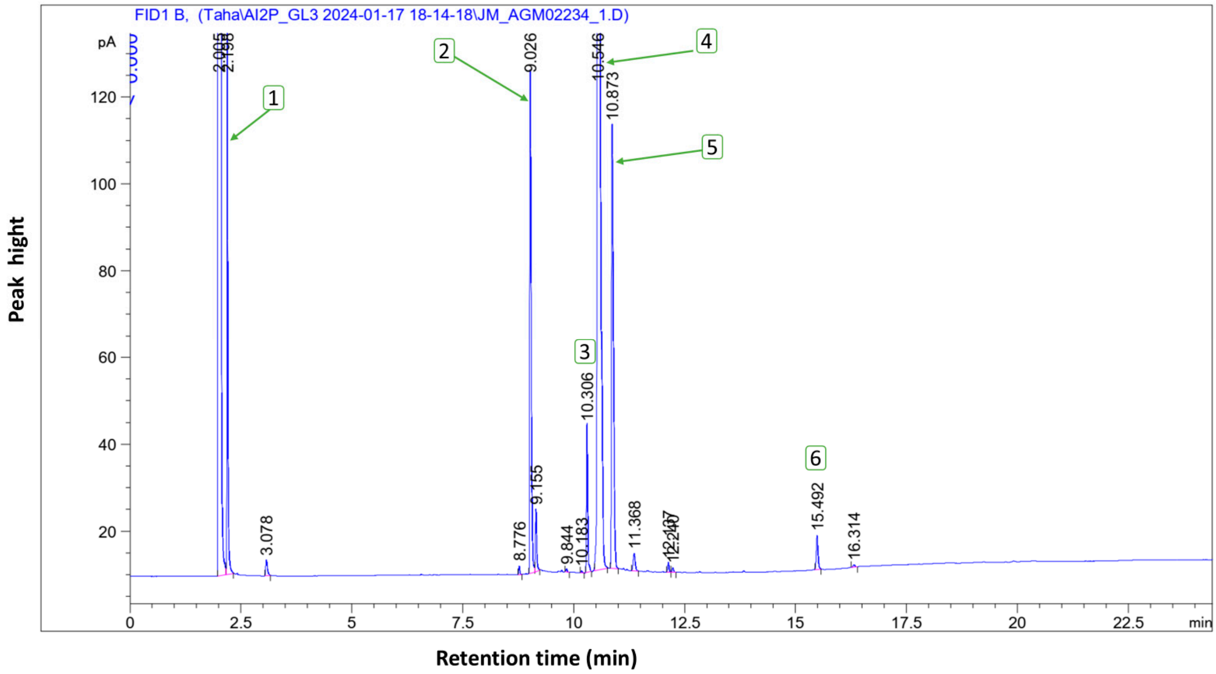

Figure S34. Total ion chromatograms (TICs) from the gas chromatography analysis of the fatty acids in AQ_C1: Arbequina_Control 1. (1: Internal standard (4-Methyl-2-pentanol); 2: Palmitic acid; 3: Stearic acid; 4: Oleic acid; 5: Linoleic acid; 6: Lignoceric acid.

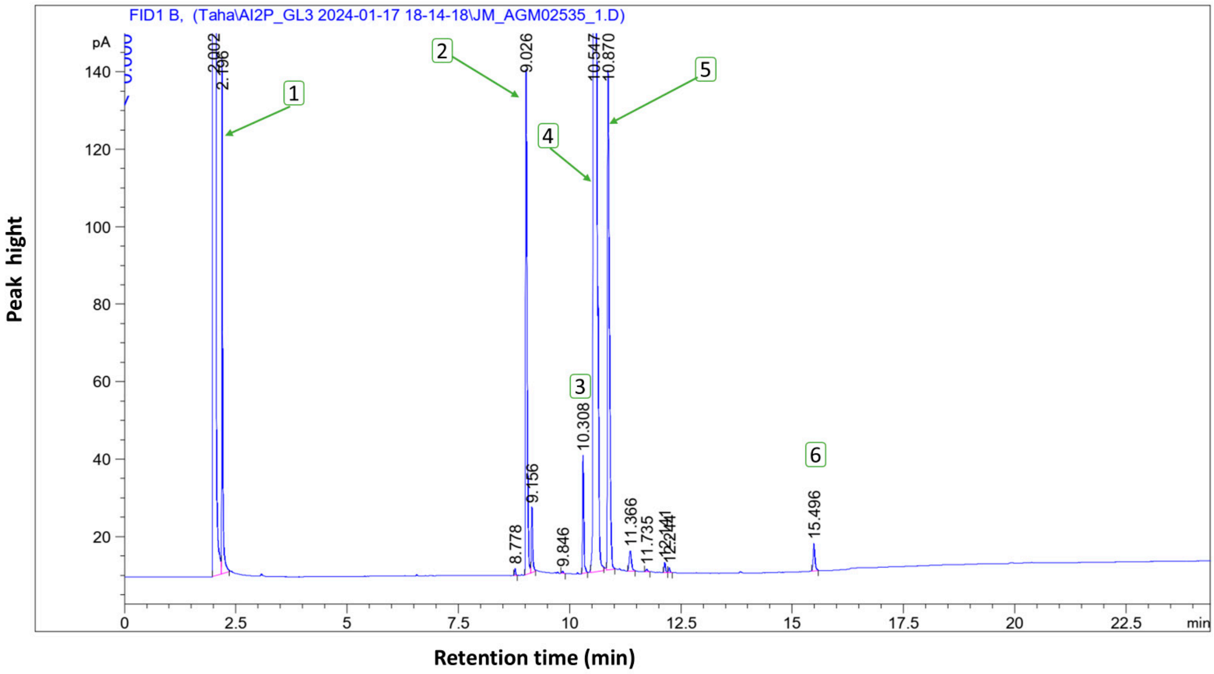

Figure S35. Total ion chromatograms (TICs) from the gas chromatography analysis of the fatty acids in AQ_S: Arbequina_Supplemented. (1: Internal standard (4-Methyl-2-pentanol); 2: Palmitic acid; 3: Stearic acid; 4: Oleic acid; 5: Linoleic acid; 6: Lignoceric acid.

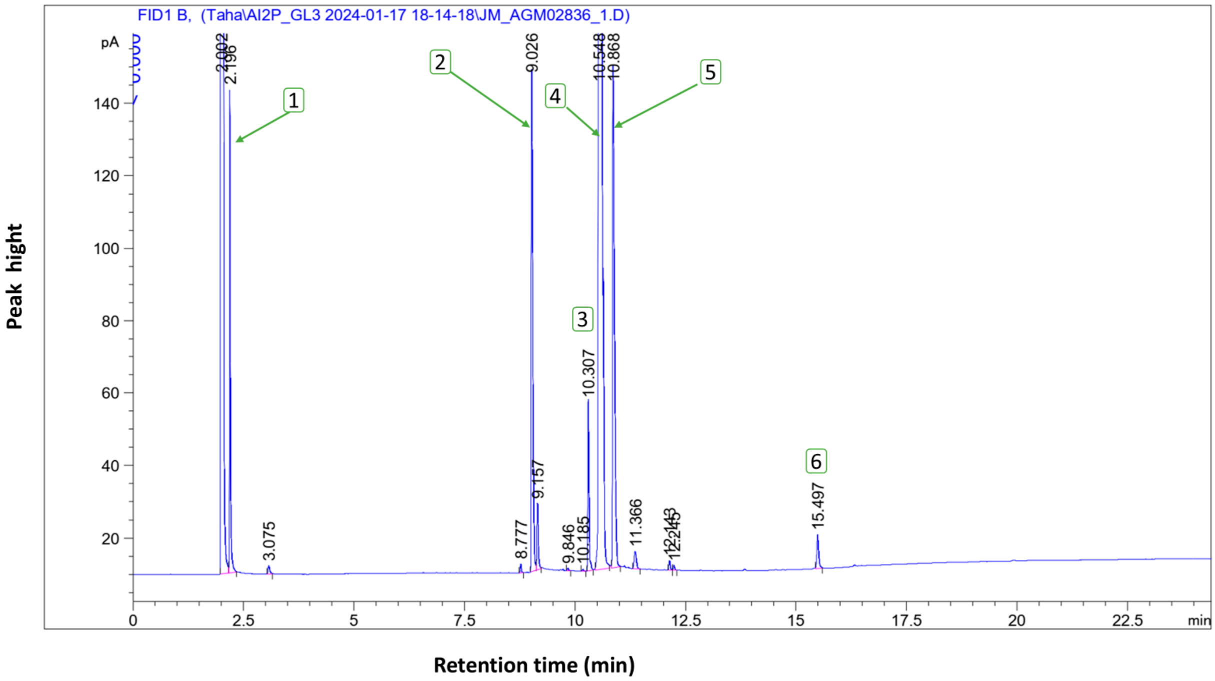

Figure S36. Total ion chromatograms (TICs) from the gas chromatography analysis of the fatty acids in AQ_C2: Arbequina_ Control 2. (1: Internal standard (4-Methyl-2-pentanol); 2: Palmitic acid; 3: Stearic acid; 4: Oleic acid; 5: Linoleic acid; 6: Lignoceric acid.

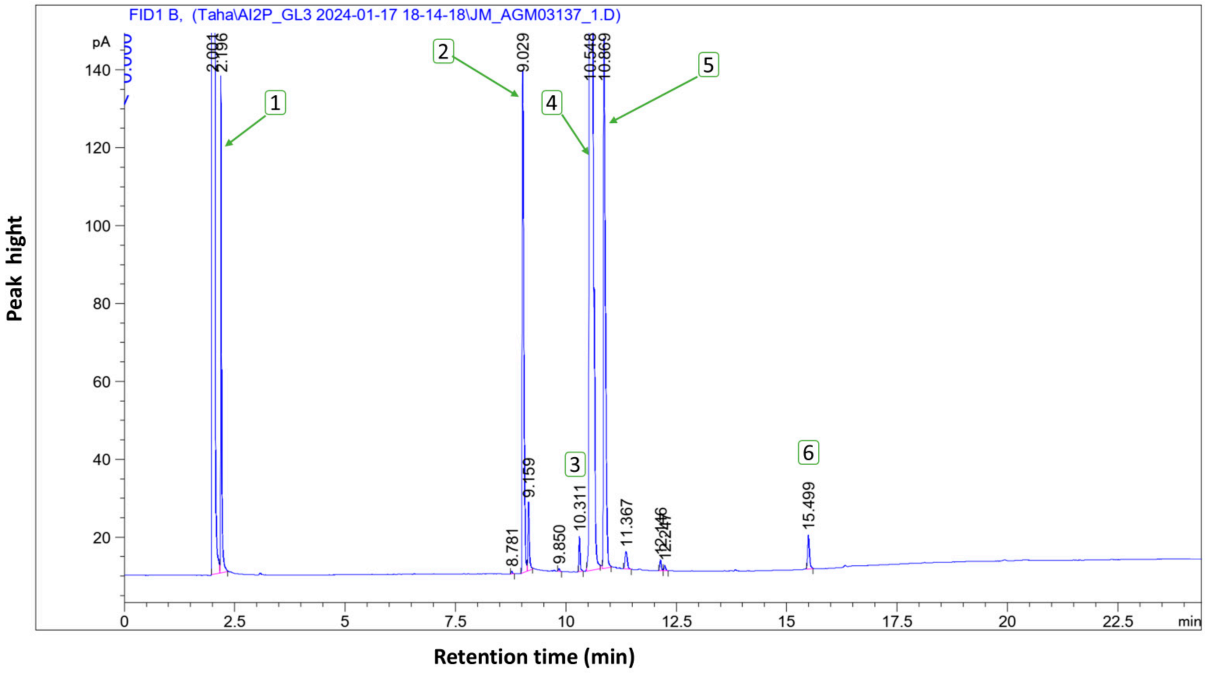

Figure S37. Total ion chromatograms (TICs) from the gas chromatography analysis of the fatty acids in AQ_1: Arbequina_Exp 1. (1: Internal standard (4-Methyl-2-pentanol); 2: Palmitic acid; 3: Stearic acid; 4: Oleic acid; 5: Linoleic acid; 6: Lignoceric acid.

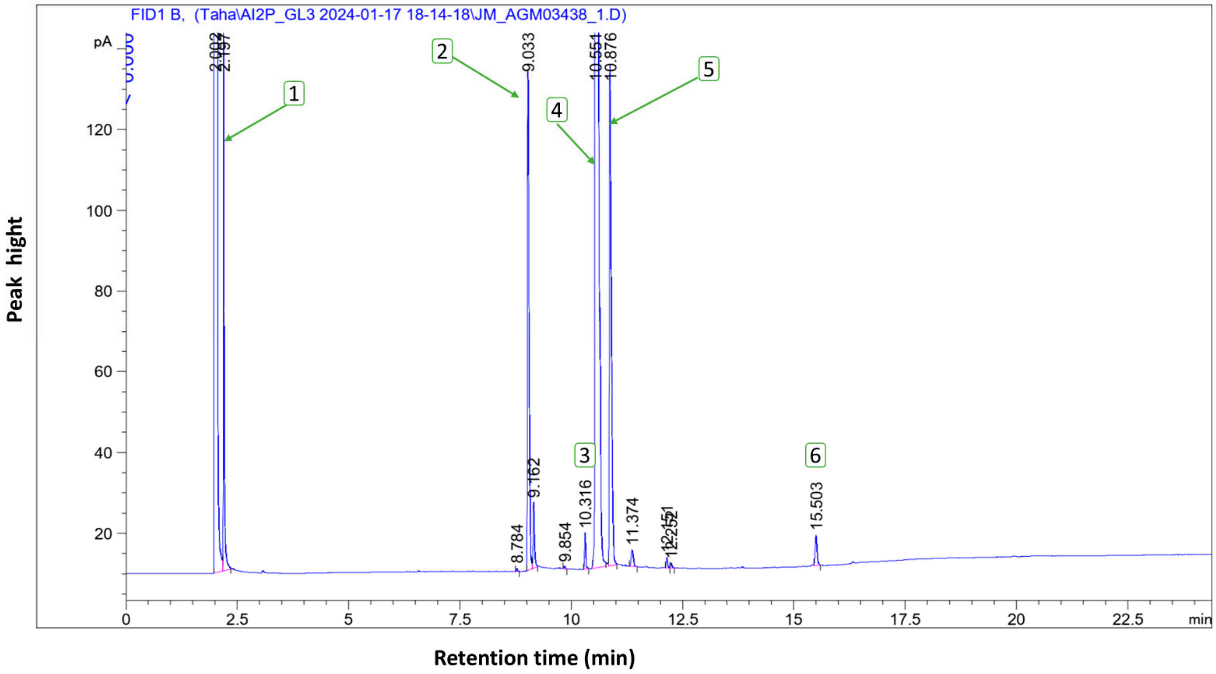

Figure S38. Total ion chromatograms (TICs) from the gas chromatography analysis of the fatty acids in AQ_2: Arbequina_Exp 2. (1: Internal standard (4-Methyl-2-pentanol); 2: Palmitic acid; 3: Stearic acid; 4: Oleic acid; 5: Linoleic acid; 6: Lignoceric acid.

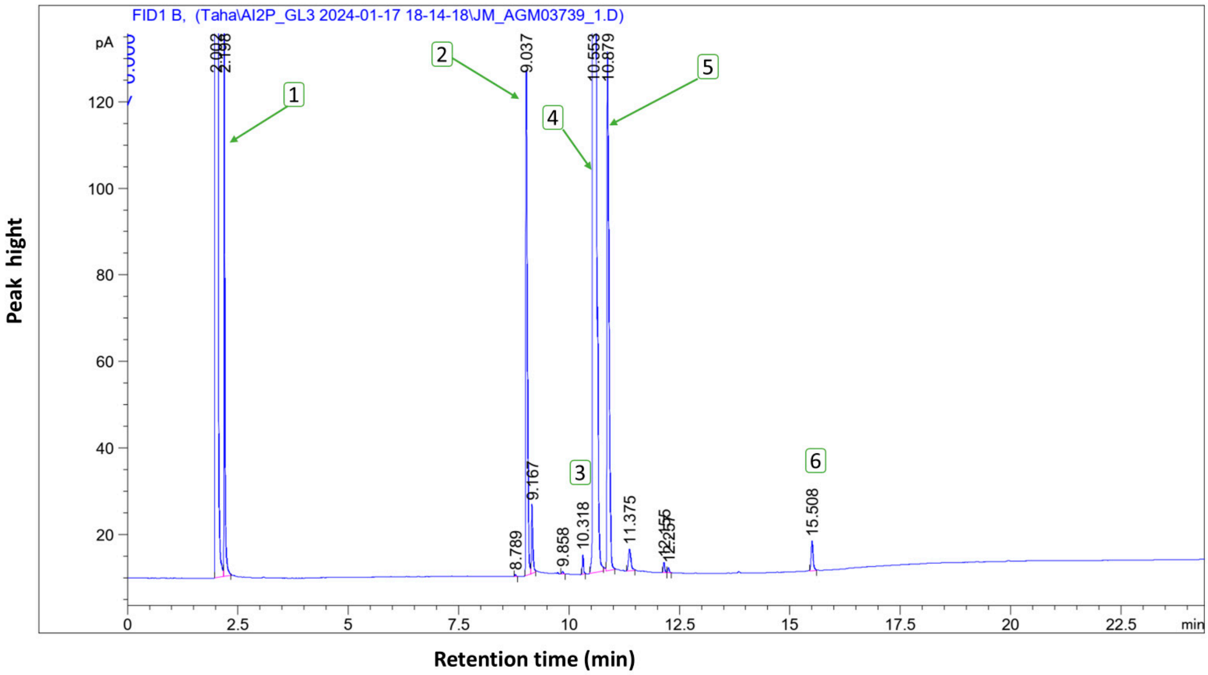

Figure S39. Total ion chromatograms (TICs) from the gas chromatography analysis of the fatty acids in AQ_3: Arbequina_Exp 3. (1: Internal standard (4-Methyl-2-pentanol); 2: Palmitic acid; 3: Stearic acid; 4: Oleic acid; 5: Linoleic acid; 6: Lignoceric acid.

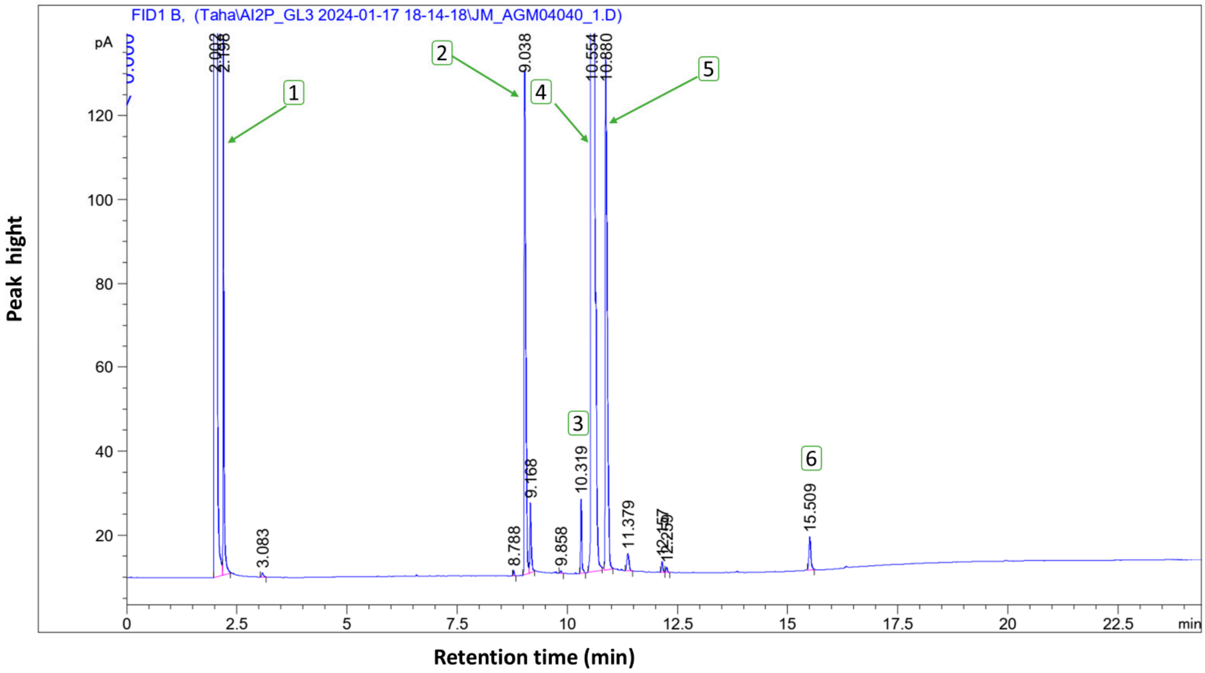

Figure S40. Total ion chromatograms (TICs) from the gas chromatography analysis of the fatty acids in AQ_4: Arbequina_Exp 4. (1: Internal standard (4-Methyl-2-pentanol); 2: Palmitic acid; 3: Stearic acid; 4: Oleic acid; 5: Linoleic acid; 6: Lignoceric acid.

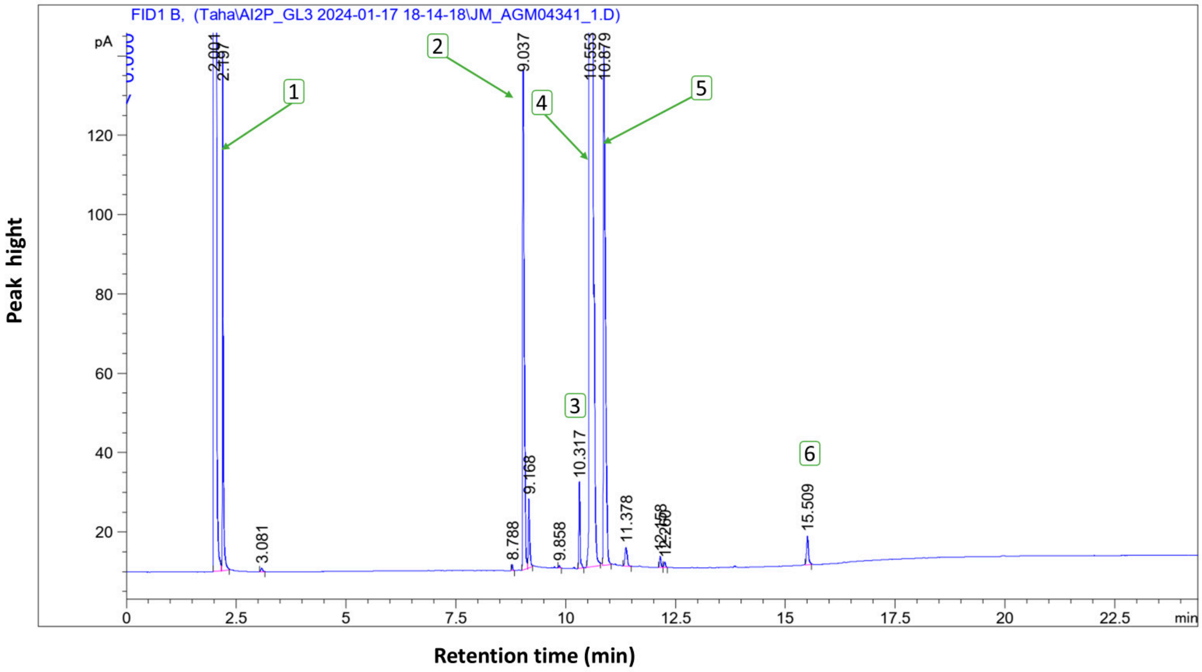

Figure S41. Total ion chromatograms (TICs) from the gas chromatography analysis of the fatty acids in AQ_5: Arbequina_Exp 5. (1: Internal standard (4-Methyl-2-pentanol); 2: Palmitic acid; 3: Stearic acid; 4: Oleic acid; 5: Linoleic acid; 6: Lignoceric acid.

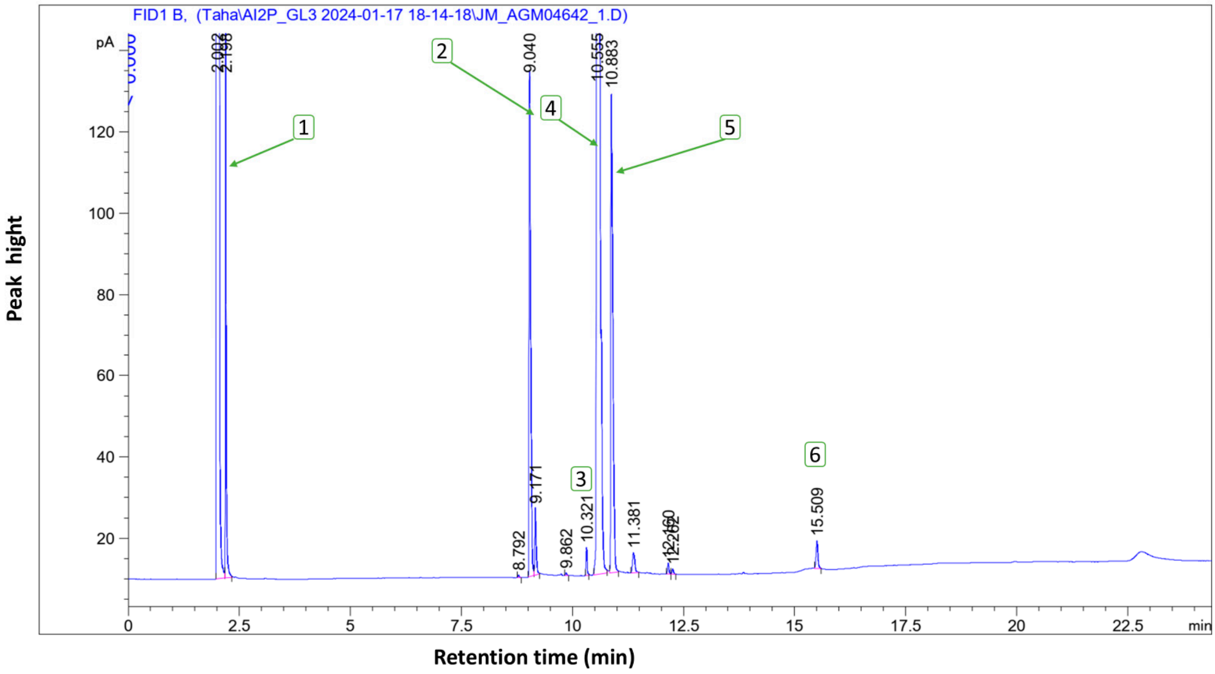

Figure S42. Total ion chromatograms (TICs) from the gas chromatography analysis of the fatty acids in AQ_6: Arbequina_Exp 6. (1: Internal standard (4-Methyl-2-pentanol); 2: Palmitic acid; 3: Stearic acid; 4: Oleic acid; 5: Linoleic acid; 6: Lignoceric acid.

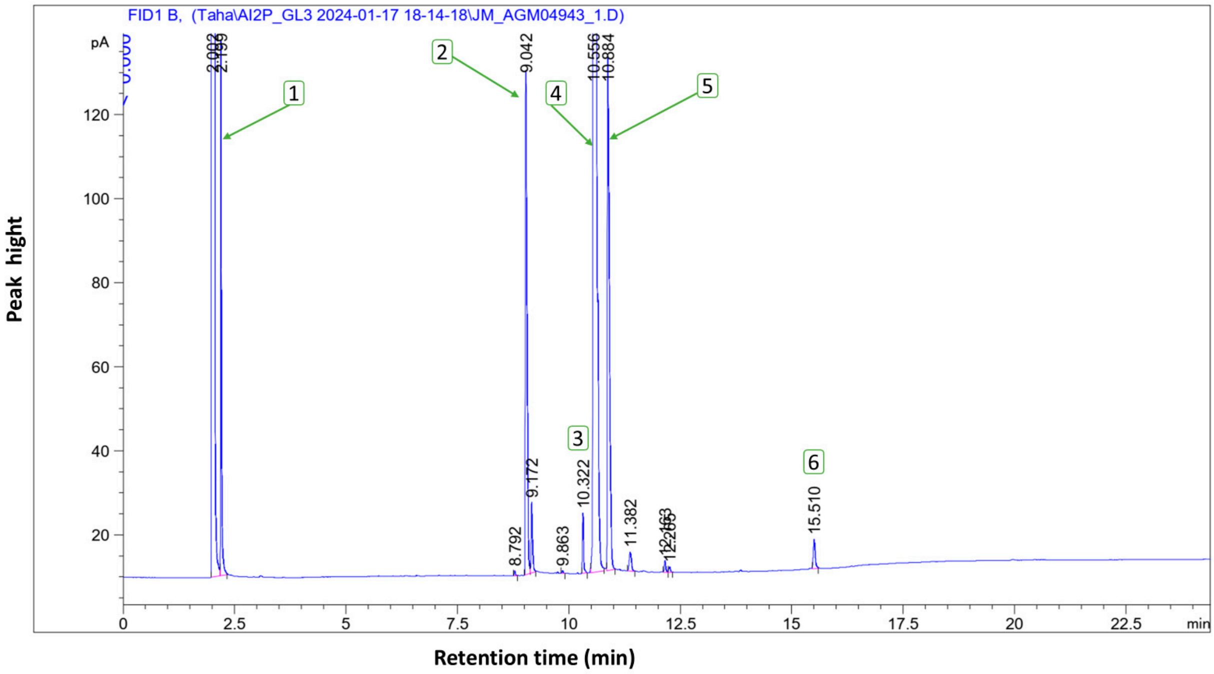

Figure S43. Total ion chromatograms (TICs) from the gas chromatography analysis of the fatty acids in AQ_7: Arbequina_Exp 7. (1: Internal standard (4-Methyl-2-pentanol); 2: Palmitic acid; 3: Stearic acid; 4: Oleic acid; 5: Linoleic acid; 6: Lignoceric acid.

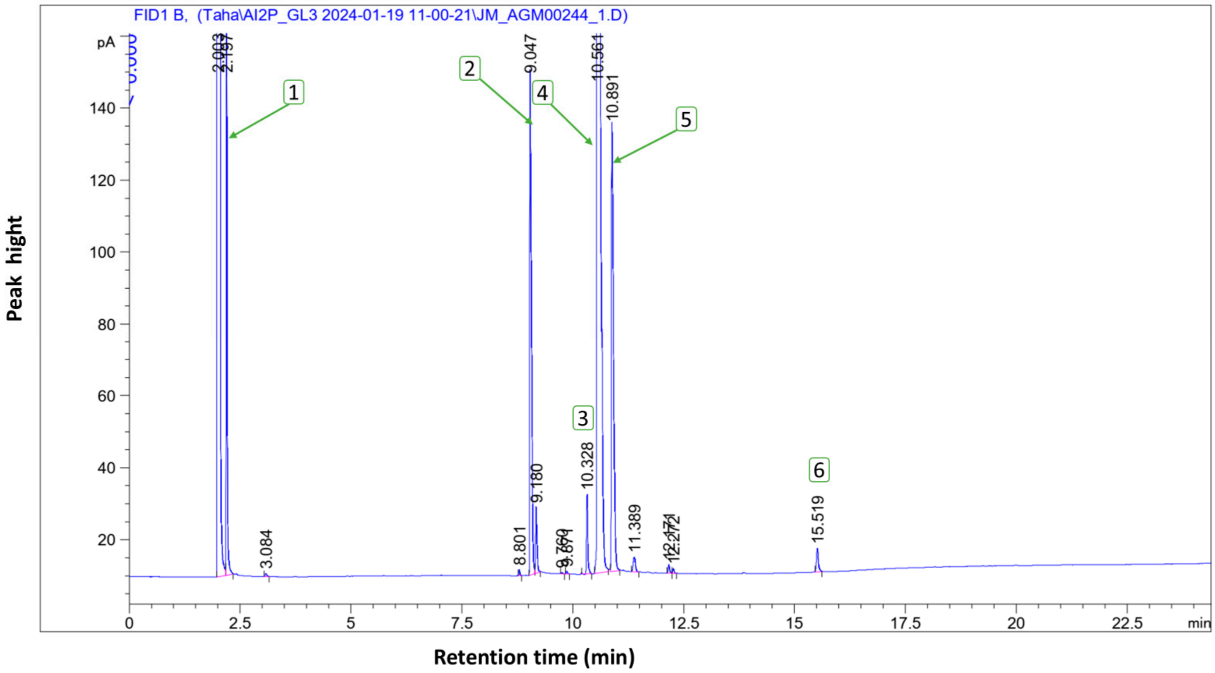

Figure S44. Total ion chromatograms (TICs) from the gas chromatography analysis of the fatty acids in AQ_8: Arbequina_Exp 8. (1: Internal standard (4-Methyl-2-pentanol); 2: Palmitic acid; 3: Stearic acid; 4: Oleic acid; 5: Linoleic acid; 6: Lignoceric acid.

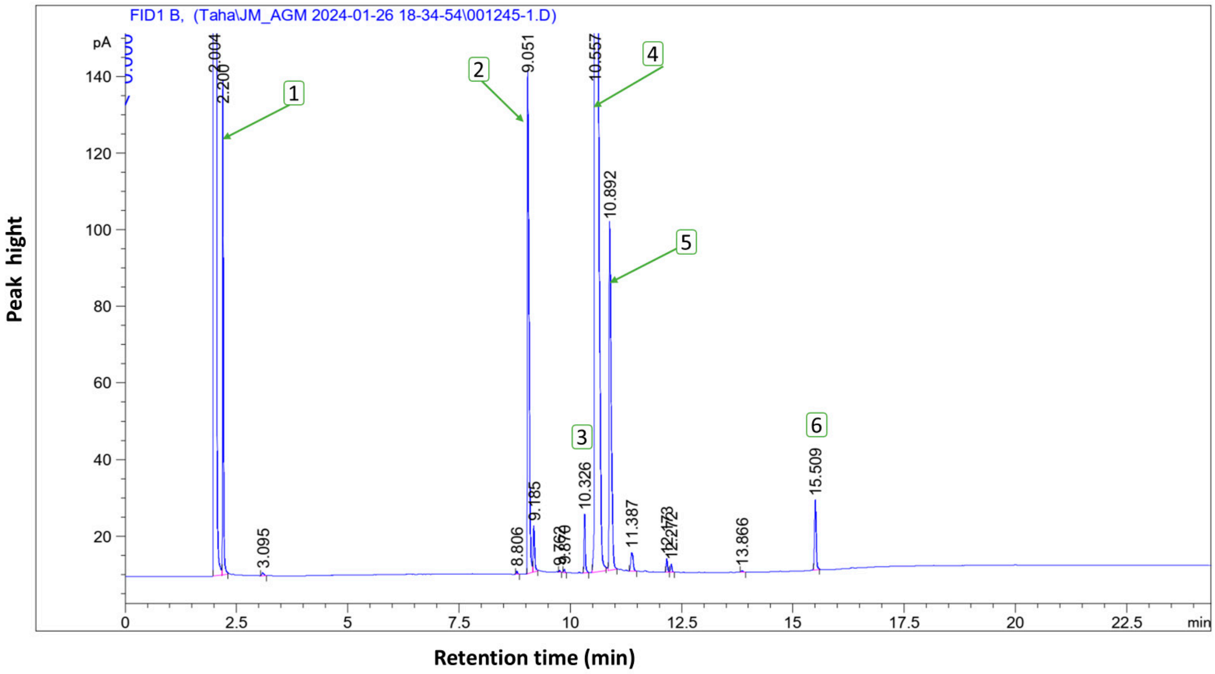

Figure S45. Total ion chromatograms (TICs) from the gas chromatography analysis of the fatty acids in HB_C1: Hojiblanca_Control 1. (1: Internal standard (4-Methyl-2-pentanol); 2: Palmitic acid; 3: Stearic acid; 4: Oleic acid; 5: Linoleic acid; 6: Lignoceric acid.

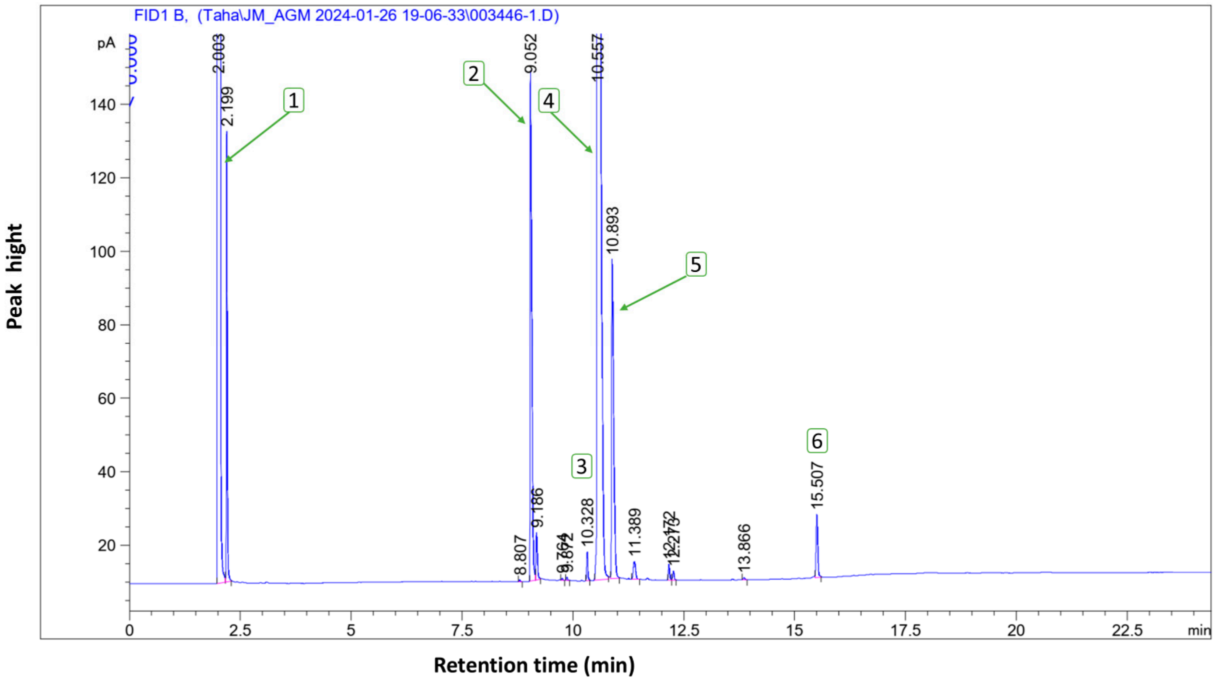

Figure S46. Total ion chromatograms (TICs) from the gas chromatography analysis of the fatty acids in Hojiblanca_Supplemented. (1: Internal standard (4-Methyl-2-pentanol); 2: Palmitic acid; 3: Stearic acid; 4: Oleic acid; 5: Linoleic acid; 6: Lignoceric acid.

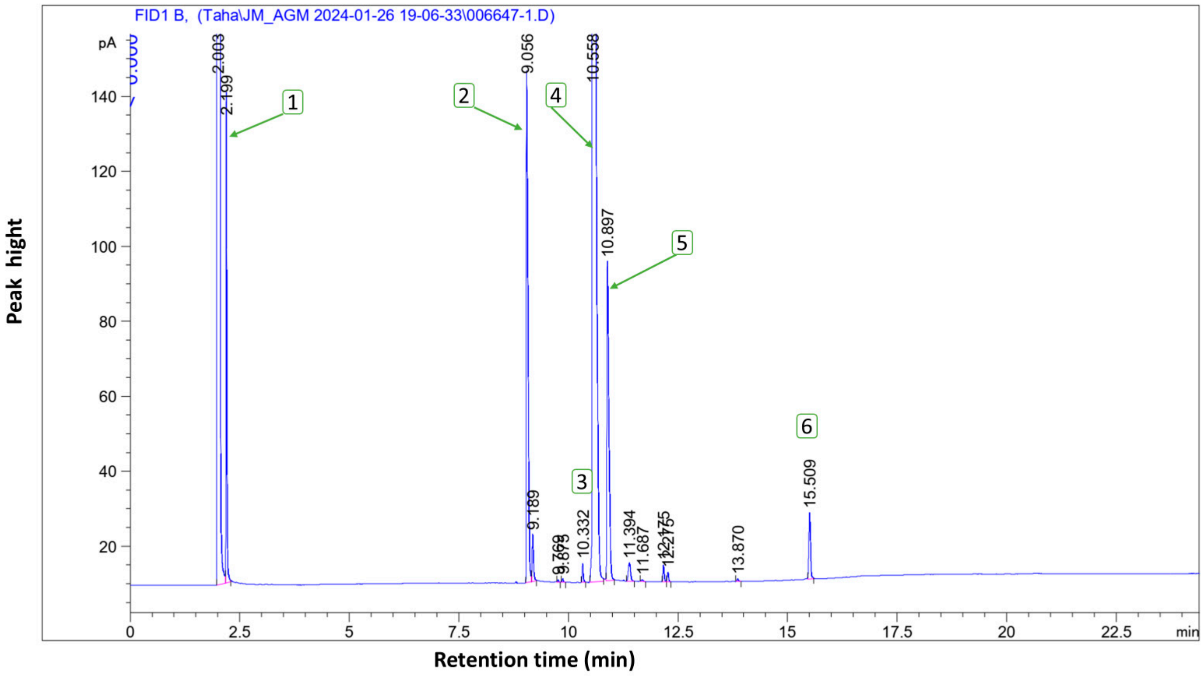

Figure S47. Total ion chromatograms (TICs) from the gas chromatography analysis of the fatty acids in Hojiblanca_Control 2. (1: Internal standard (4-Methyl-2-pentanol); 2: Palmitic acid; 3: Stearic acid; 4: Oleic acid; 5: Linoleic acid; 6: Lignoceric acid.

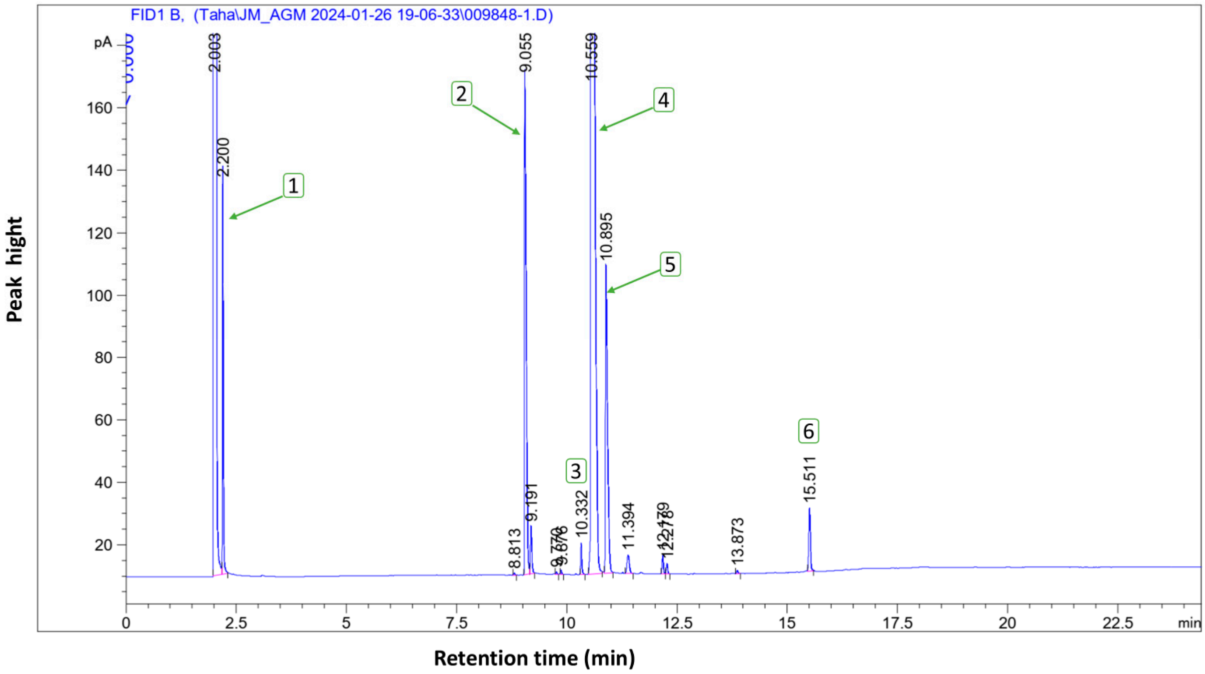

Figure S48. Total ion chromatograms (TICs) from the gas chromatography analysis of the fatty acids in HB_1: Hojiblanca_Exp 1. (1: Internal standard (4-Methyl-2-pentanol); 2: Palmitic acid; 3: Stearic acid; 4: Oleic acid; 5: Linoleic acid; 6: Lignoceric acid.

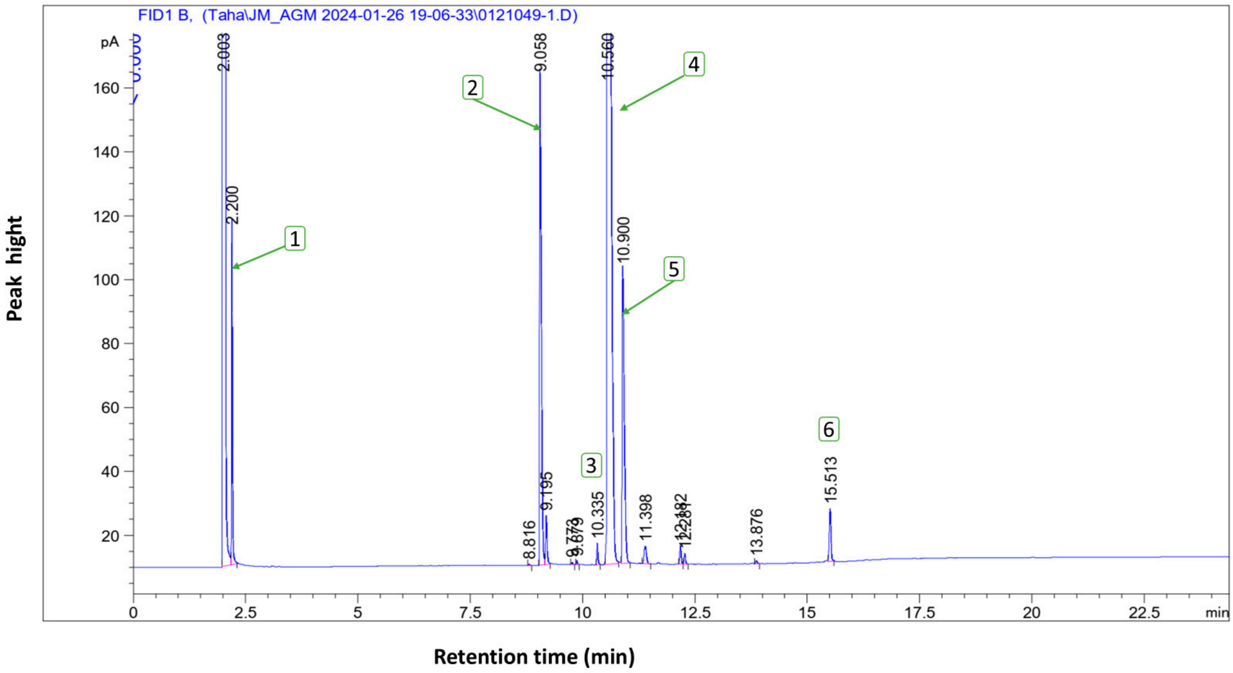

Figure S49. Total ion chromatograms (TICs) from the gas chromatography analysis of the fatty acids in HB_2: Hojiblanca_Exp 2. (1: Internal standard (4-Methyl-2-pentanol); 2: Palmitic acid; 3: Stearic acid; 4: Oleic acid; 5: Linoleic acid; 6: Lignoceric acid.

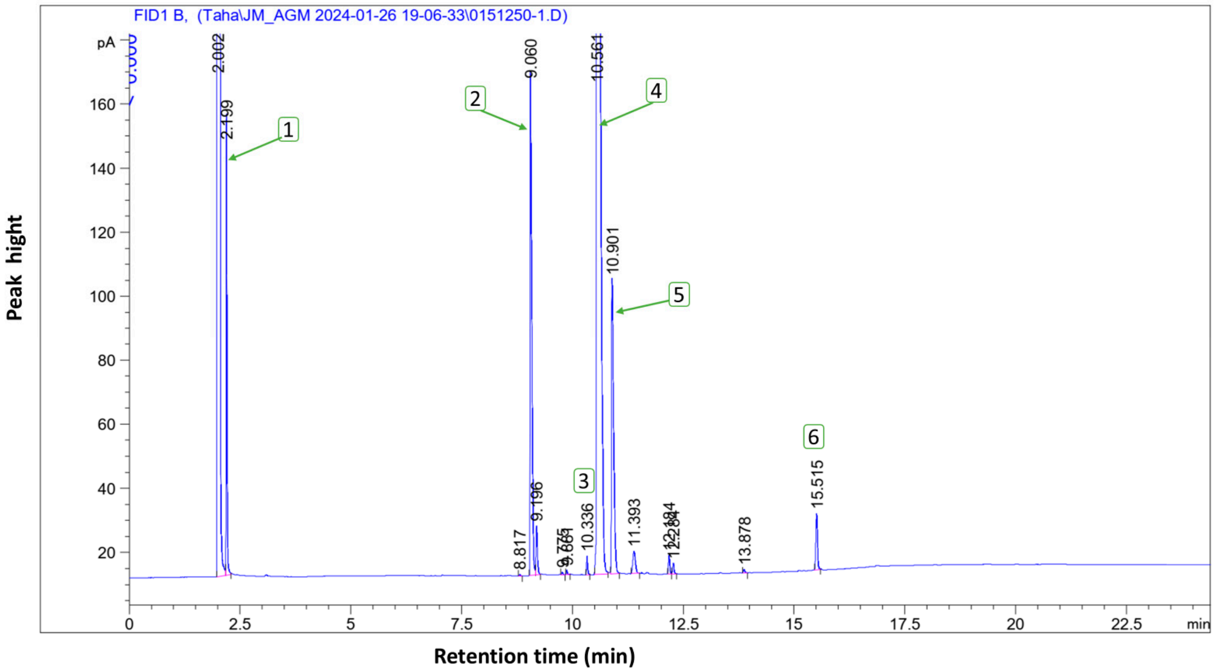

Figure S50. Total ion chromatograms (TICs) from the gas chromatography analysis of the fatty acids in HB_3: Hojiblanca_Exp 3. (1: Internal standard (4-Methyl-2-pentanol); 2: Palmitic acid; 3: Stearic acid; 4: Oleic acid; 5: Linoleic acid; 6: Lignoceric acid.

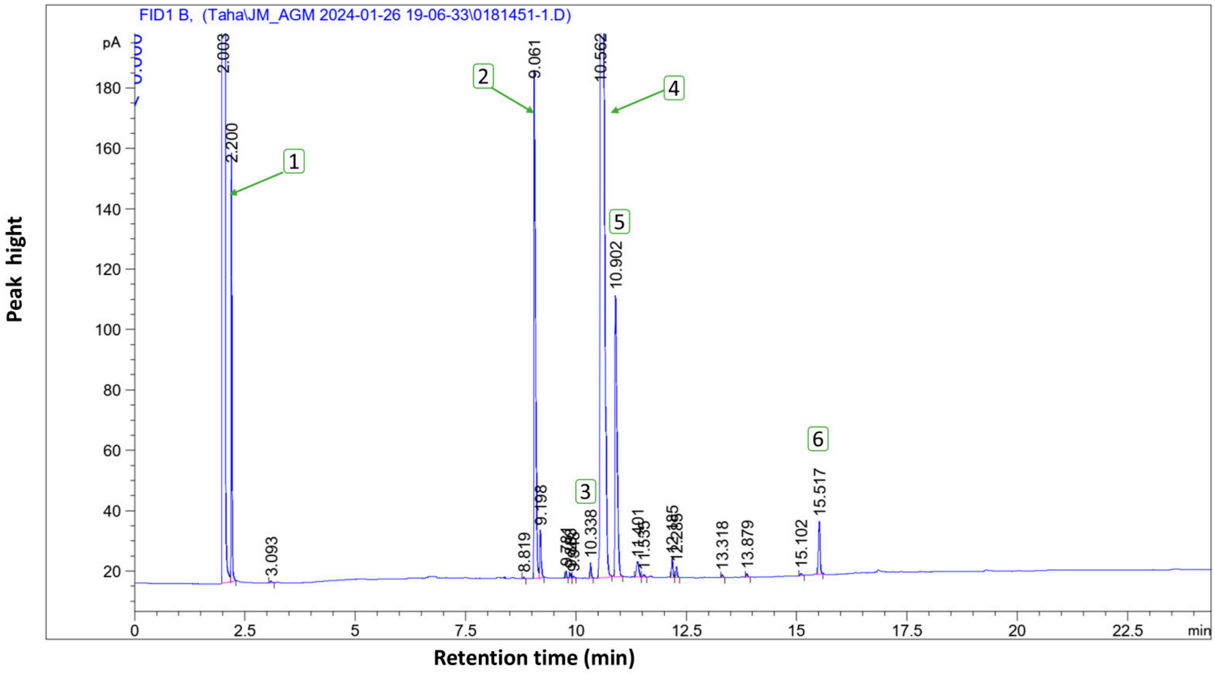

Figure S51. Total ion chromatograms (TICs) from the gas chromatography analysis of the fatty acids in HB_4: Hojiblanca_Exp 4. (1: Internal standard (4-Methyl-2-pentanol); 2: Palmitic acid; 3: Stearic acid; 4: Oleic acid; 5: Linoleic acid; 6: Lignoceric acid.

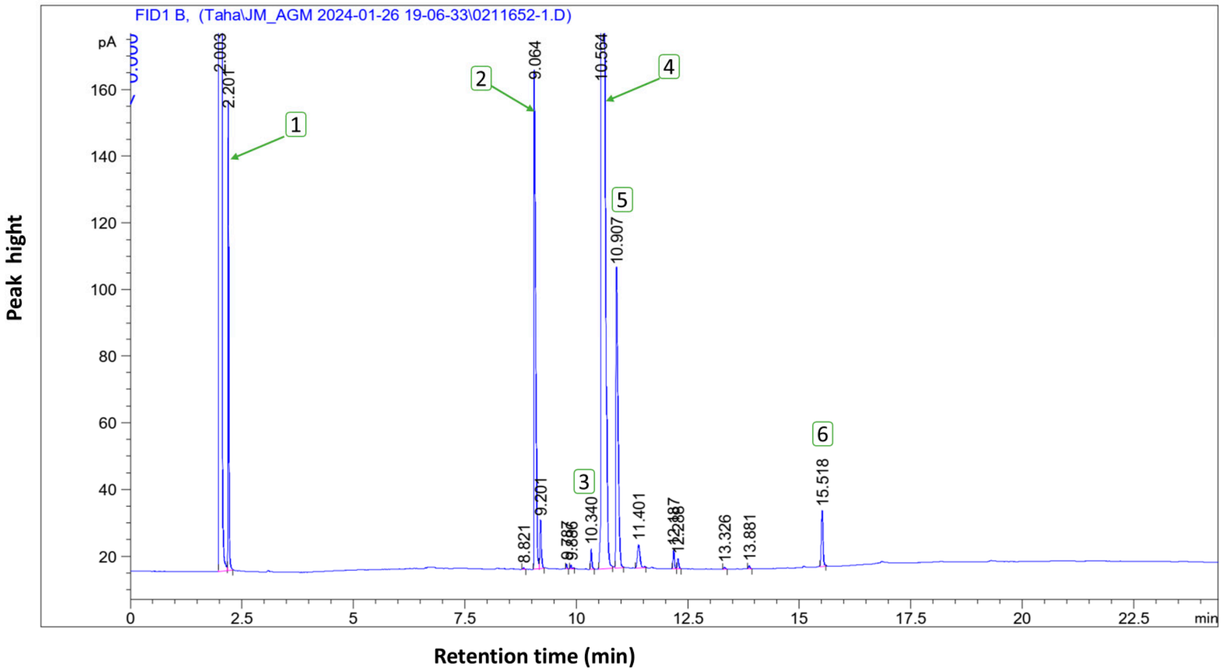

Figure S52. Total ion chromatograms (TICs) from the gas chromatography analysis of the fatty acids in HB_5: Hojiblanca_Exp 5. (1: Internal standard (4-Methyl-2-pentanol); 2: Palmitic acid; 3: Stearic acid; 4: Oleic acid; 5: Linoleic acid; 6: Lignoceric acid.

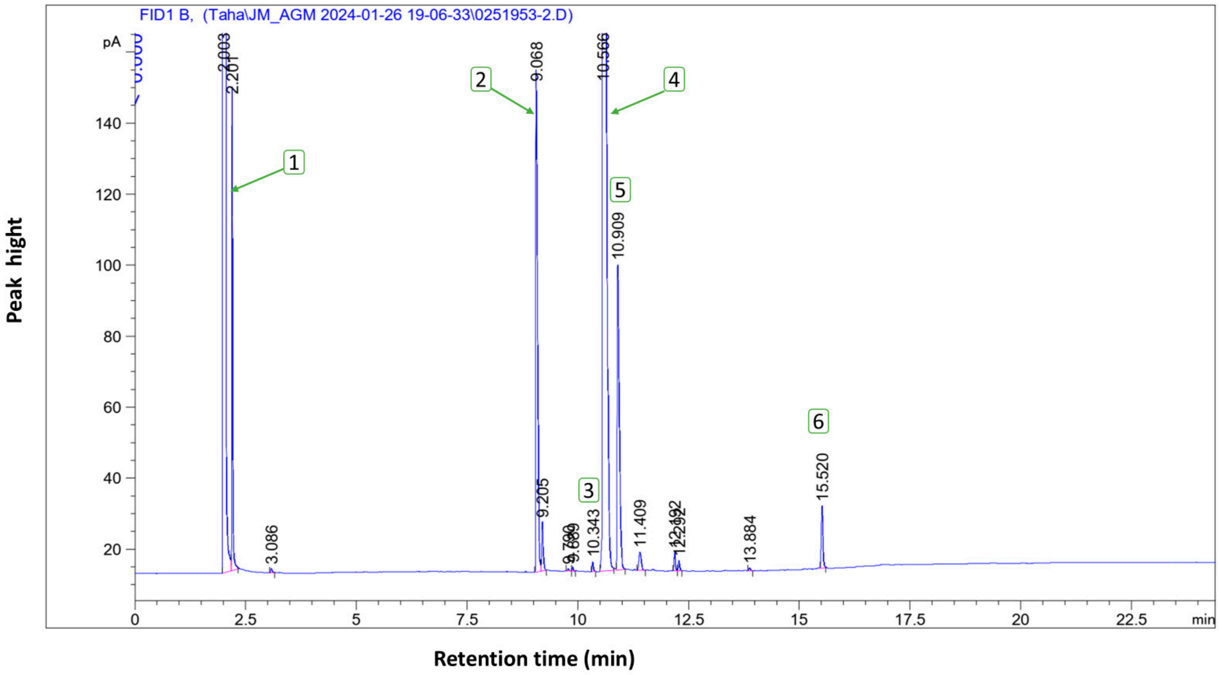

Figure S53. Total ion chromatograms (TICs) from the gas chromatography analysis of the fatty acids in HB_6: Hojiblanca_Exp 6. (1: Internal standard (4-Methyl-2-pentanol); 2: Palmitic acid; 3: Stearic acid; 4: Oleic acid; 5: Linoleic acid; 6: Lignoceric acid.

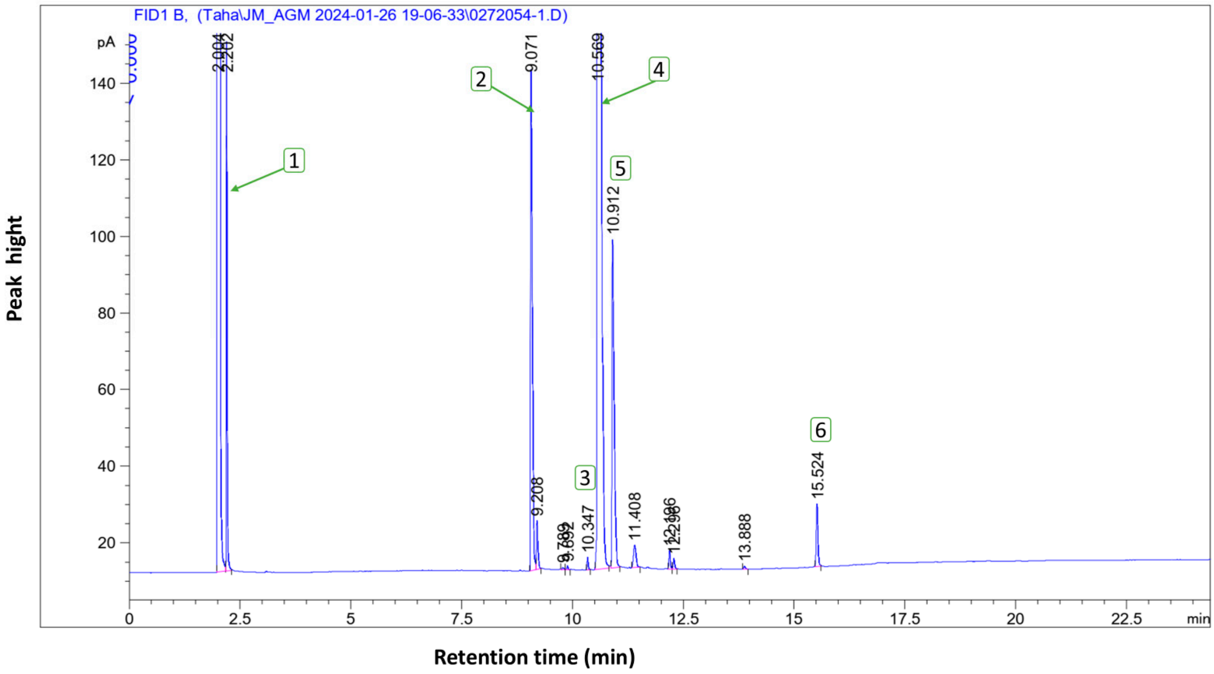

Figure S54. Total ion chromatograms (TICs) from the gas chromatography analysis of the fatty acids in HB_7: Hojiblanca_Exp 7. (1: Internal standard (4-Methyl-2-pentanol); 2: Palmitic acid; 3: Stearic acid; 4: Oleic acid; 5: Linoleic acid; 6: Lignoceric acid.

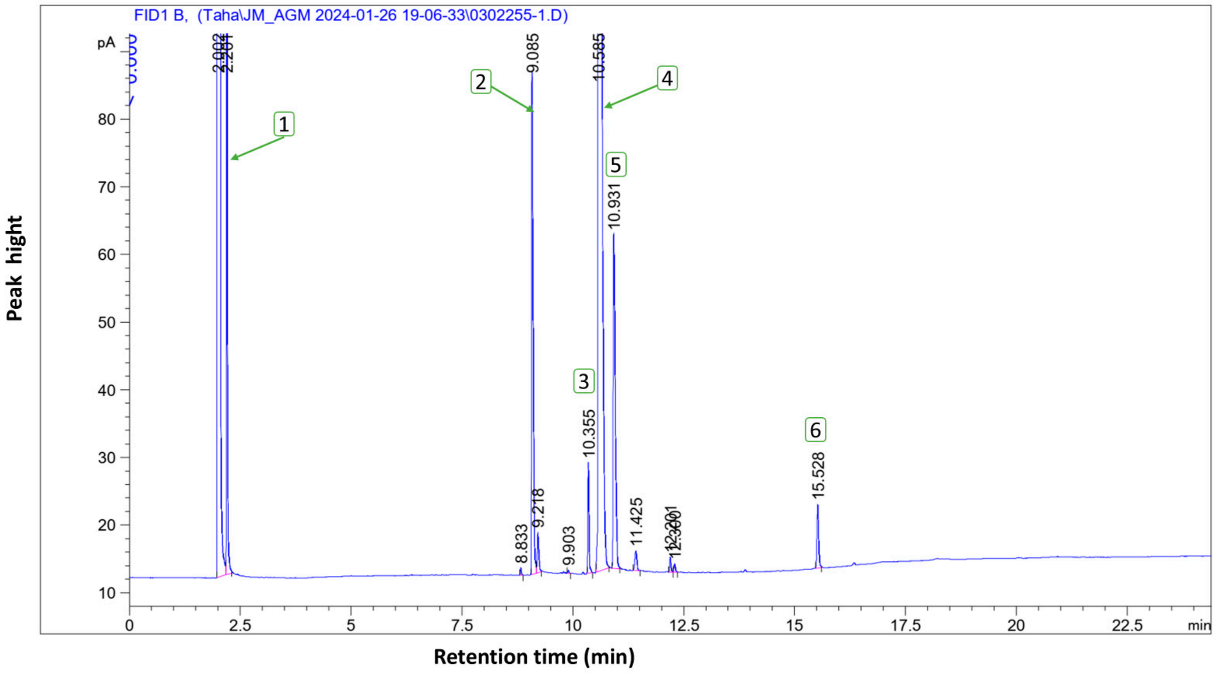

Figure S55. Total ion chromatograms (TICs) from the gas chromatography analysis of the fatty acids in HB_8: Hojiblanca_Exp 8. (1: Internal standard (4-Methyl-2-pentanol); 2: Palmitic acid; 3: Stearic acid; 4: Oleic acid; 5: Linoleic acid; 6: Lignoceric acid.

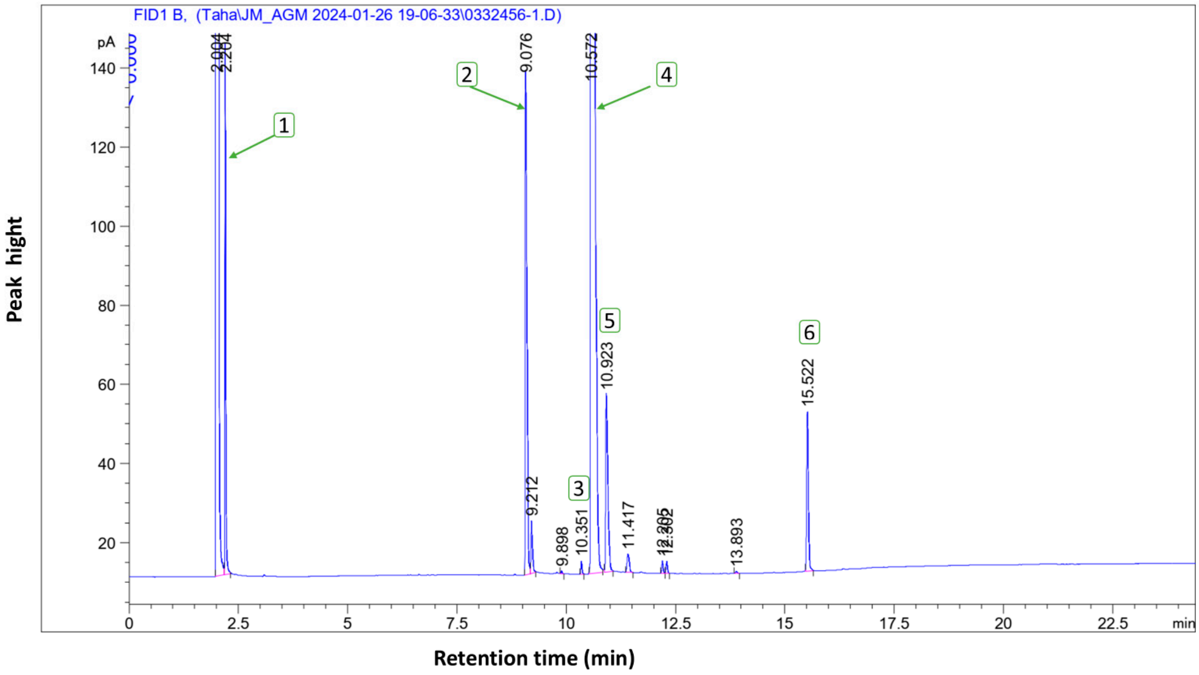

Figure S56. Total ion chromatograms (TICs) from the gas chromatography analysis of the fatty acids in MZ_C1: Manzanilla_ Control 1. (1: Internal standard (4-Methyl-2-pentanol); 2: Palmitic acid; 3: Stearic acid; 4: Oleic acid; 5: Linoleic acid; 6: Lignoceric acid.

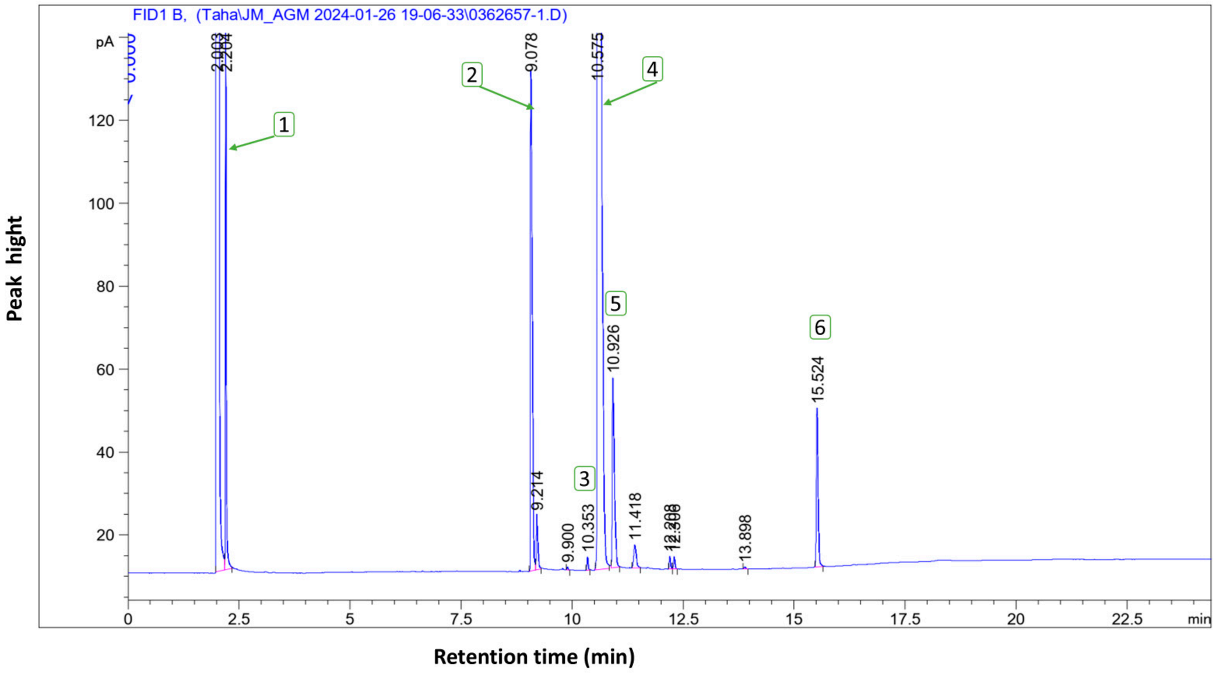

Figure S57. Total ion chromatograms (TICs) from the gas chromatography analysis of the fatty acids in MZ_S: Manzanilla_Supplemented. (1: Internal standard (4-Methyl-2-pentanol); 2: Palmitic acid; 3: Stearic acid; 4: Oleic acid; 5: Linoleic acid; 6: Lignoceric acid.

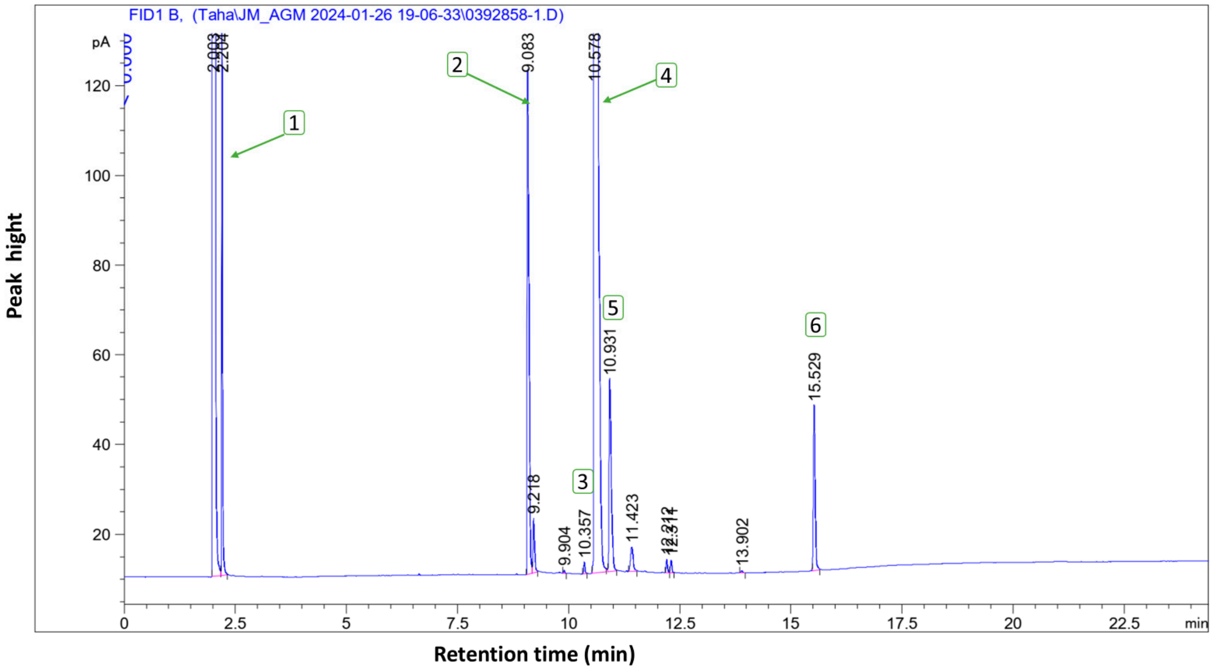

Figure S58. Total ion chromatograms (TICs) from the gas chromatography analysis of the fatty acids in MZ_C2: Manzanilla_Control 2. (1: Internal standard (4-Methyl-2-pentanol); 2: Palmitic acid; 3: Stearic acid; 4: Oleic acid; 5: Linoleic acid; 6: Lignoceric acid.

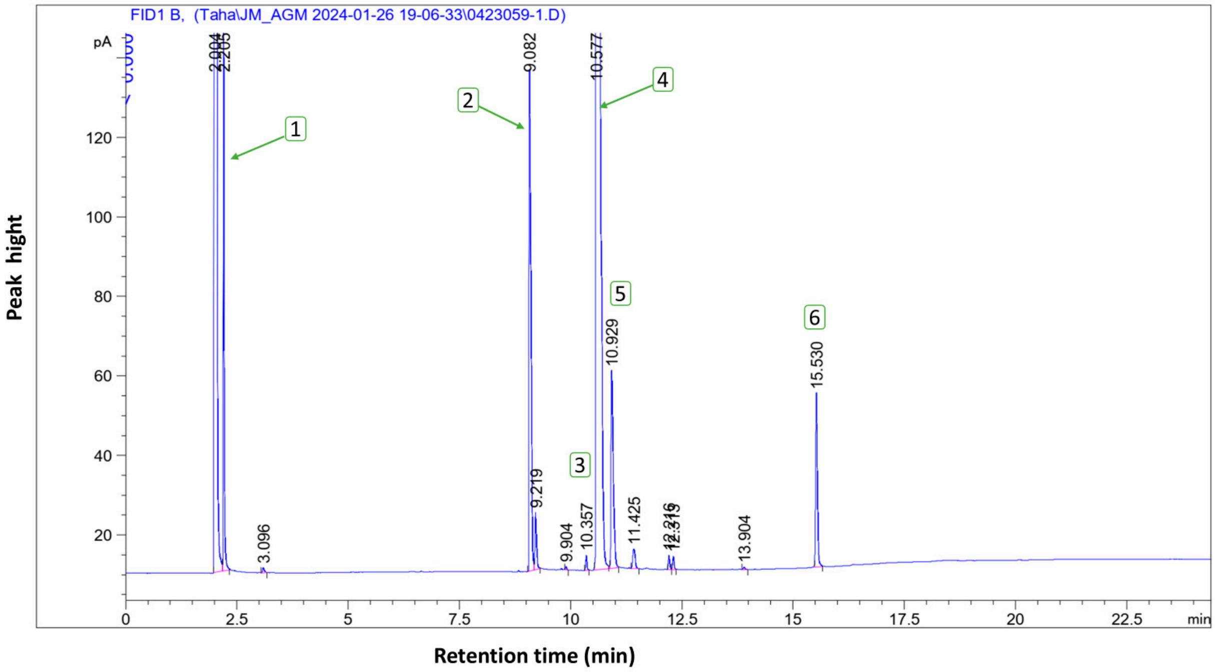

Figure S59. Total ion chromatograms (TICs) from the gas chromatography analysis of the fatty acids in MZ_1: Manzanilla_Exp 1. (1: Internal standard (4-Methyl-2-pentanol); 2: Palmitic acid; 3: Stearic acid; 4: Oleic acid; 5: Linoleic acid; 6: Lignoceric acid.

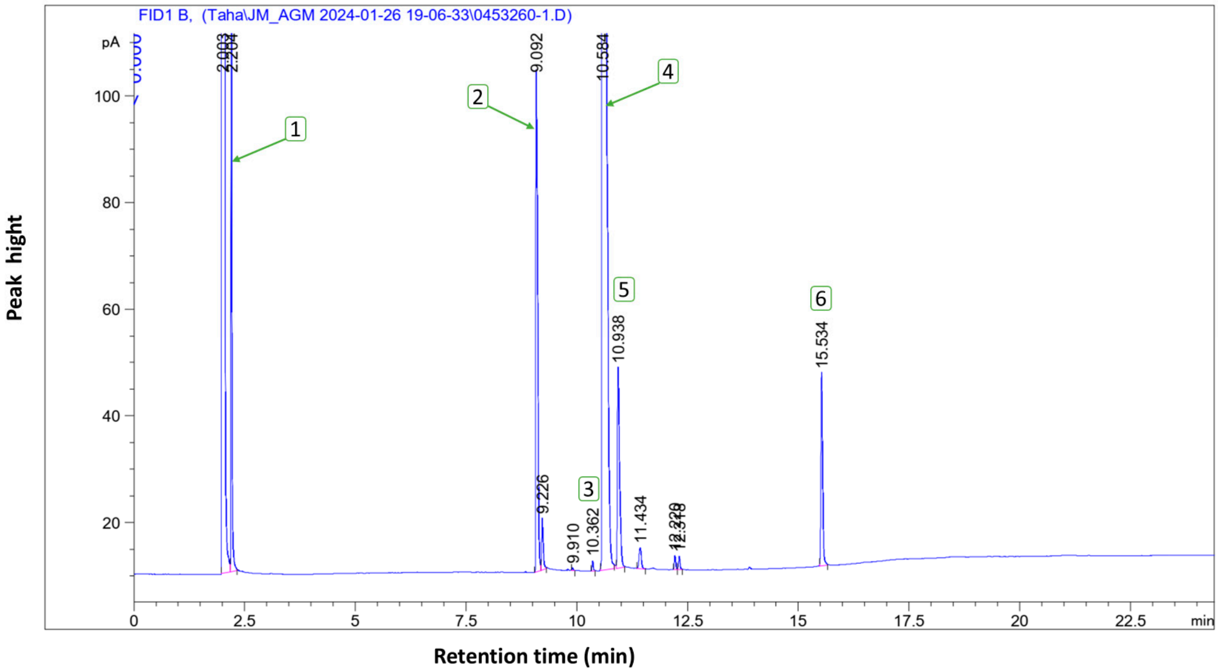

Figure S60. Total ion chromatograms (TICs) from the gas chromatography analysis of the fatty acids in MZ_2: Manzanilla_Exp 2. (1: Internal standard (4-Methyl-2-pentanol); 2: Palmitic acid; 3: Stearic acid; 4: Oleic acid; 5: Linoleic acid; 6: Lignoceric acid.

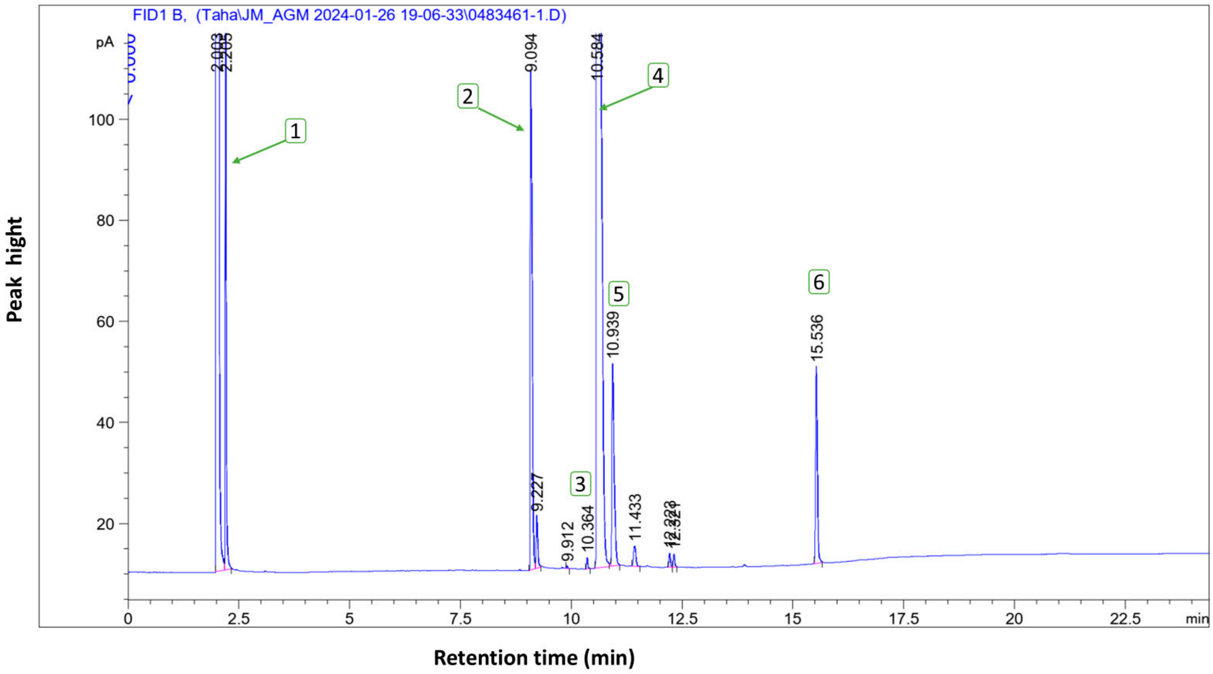

Figure S61. Total ion chromatograms (TICs) from the gas chromatography analysis of the fatty acids in MZ_3: Manzanilla_Exp 3. (1: Internal standard (4-Methyl-2-pentanol); 2: Palmitic acid; 3: Stearic acid; 4: Oleic acid; 5: Linoleic acid; 6: Lignoceric acid.

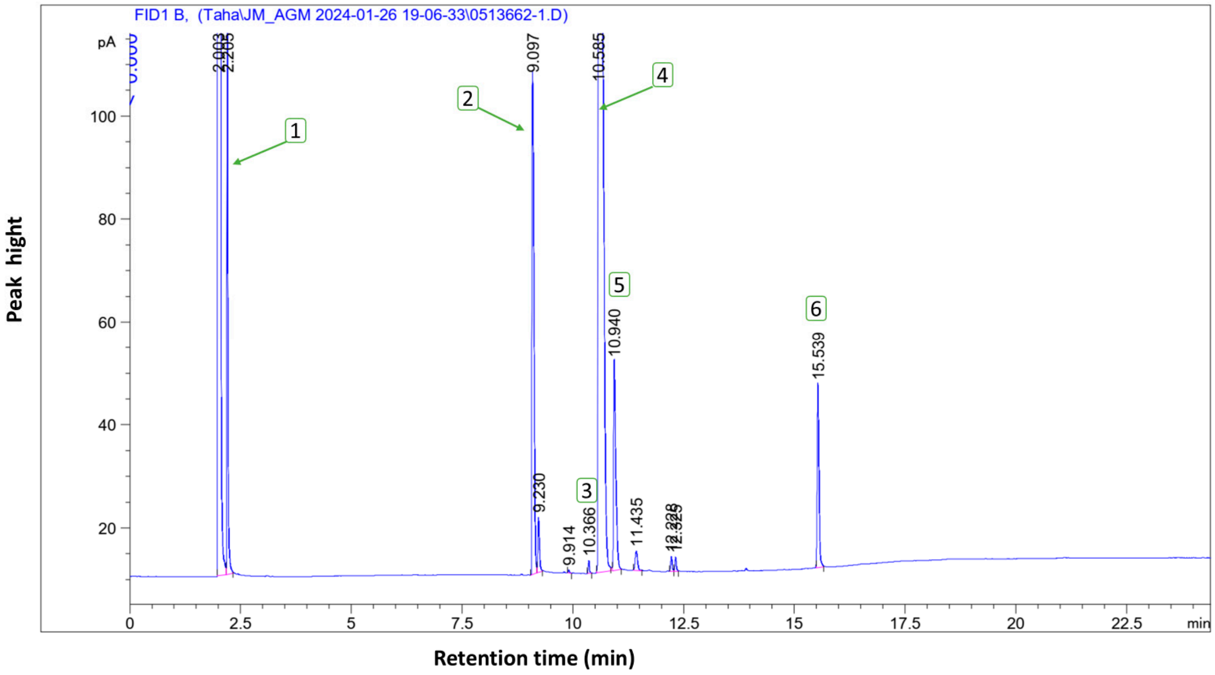

Figure S62. Total ion chromatograms (TICs) from the gas chromatography analysis of the fatty acids in MZ_4: Manzanilla_Exp 4. (1: Internal standard (4-Methyl-2-pentanol); 2: Palmitic acid; 3: Stearic acid; 4: Oleic acid; 5: Linoleic acid; 6: Lignoceric acid.

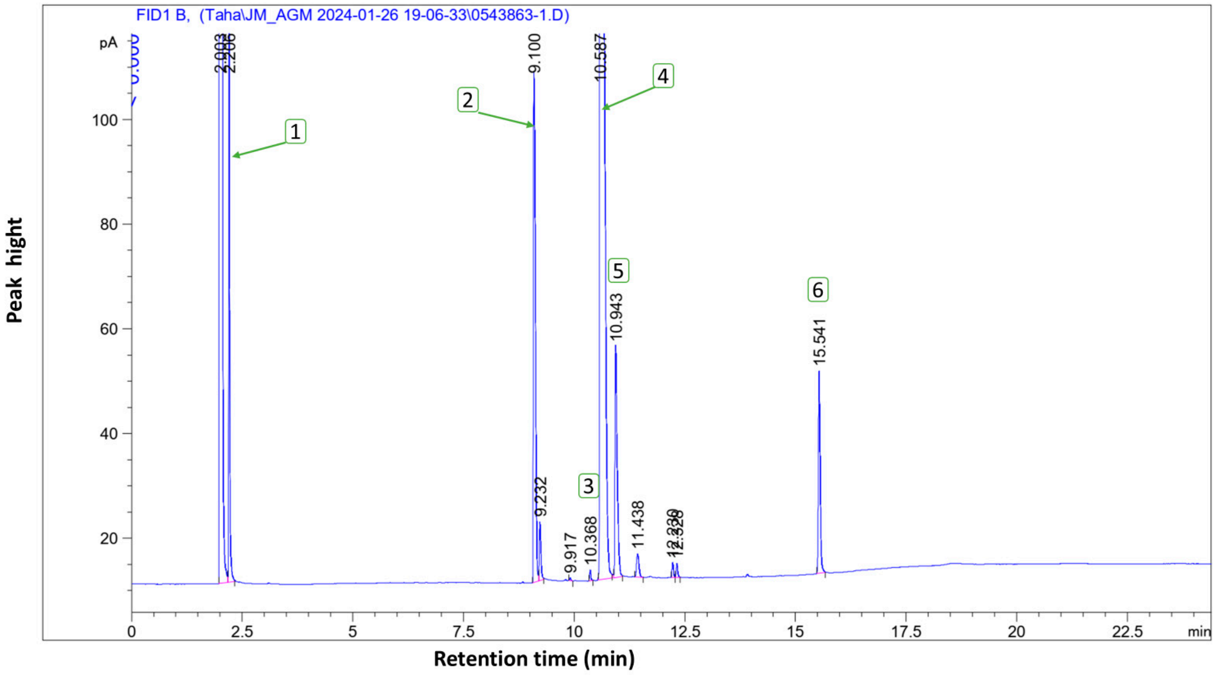

Figure S63. Total ion chromatograms (TICs) from the gas chromatography analysis of the fatty acids in MZ_5: Manzanilla_Exp 5. (1: Internal standard (4-Methyl-2-pentanol); 2: Palmitic acid; 3: Stearic acid; 4: Oleic acid; 5: Linoleic acid; 6: Lignoceric acid.

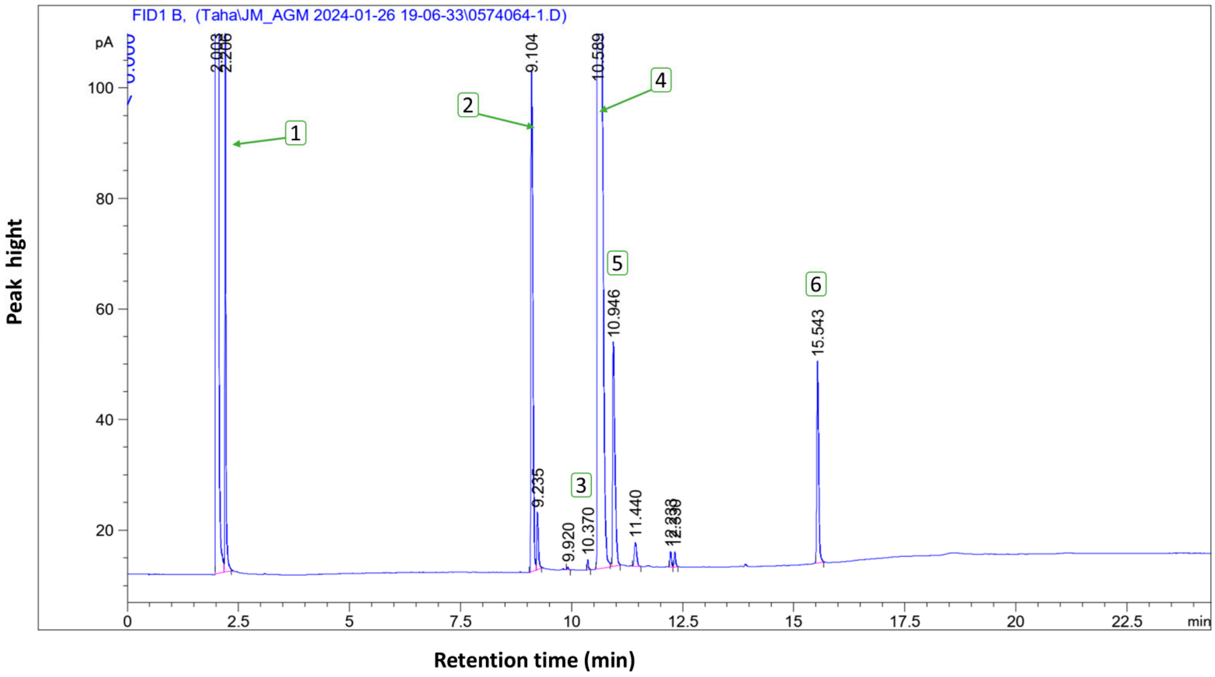

Figure S64. Total ion chromatograms (TICs) from the gas chromatography analysis of the fatty acids in MZ_6: Manzanilla_Exp 6. (1: Internal standard (4-Methyl-2-pentanol); 2: Palmitic acid; 3: Stearic acid; 4: Oleic acid; 5: Linoleic acid; 6: Lignoceric acid.

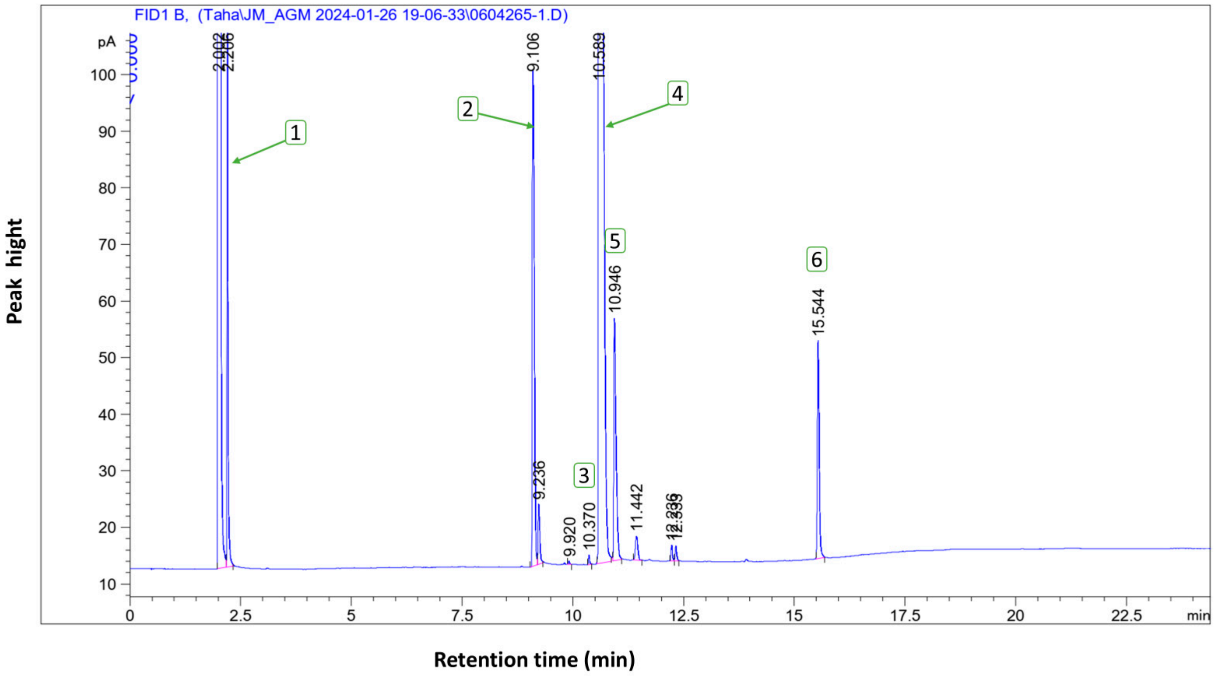

Figure S65. Total ion chromatograms (TICs) from the gas chromatography analysis of the fatty acids in MZ_7: Manzanilla_Exp 7. (1: Internal standard (4-Methyl-2-pentanol); 2: Palmitic acid; 3: Stearic acid; 4: Oleic acid; 5: Linoleic acid; 6: Lignoceric acid.

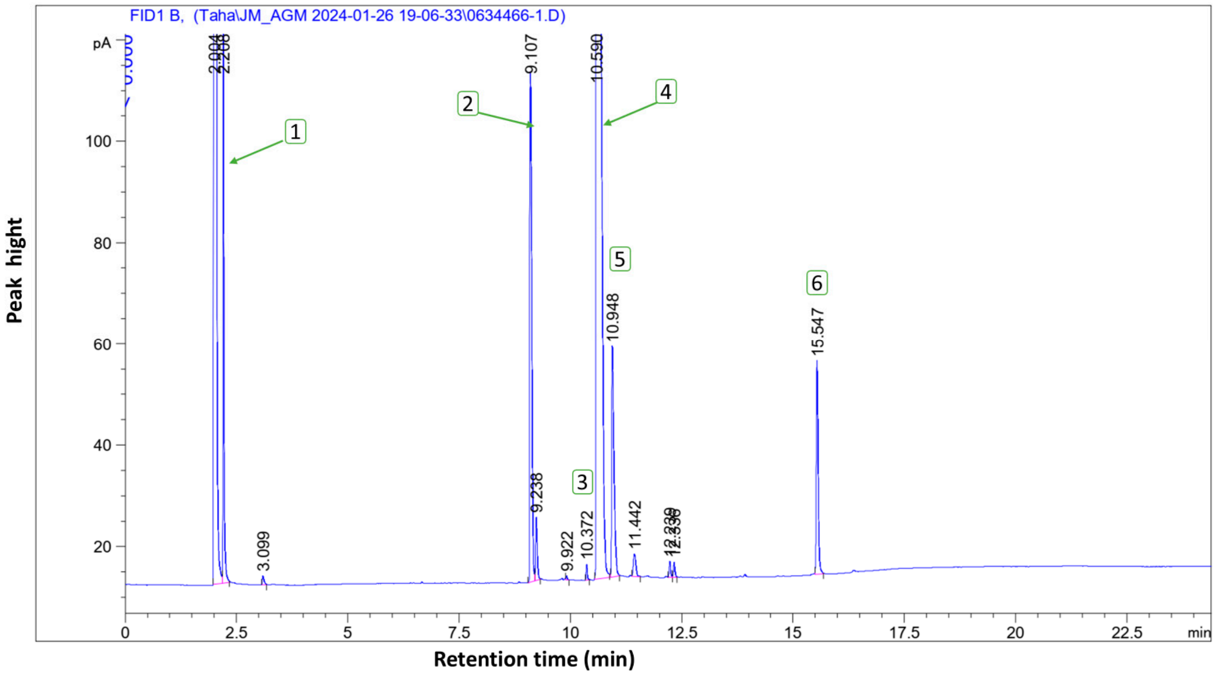

Figure S66. Total ion chromatograms (TICs) from the gas chromatography analysis of the fatty acids in MZ_8: Manzanilla_Exp 8. (1: Internal standard (4-Methyl-2-pentanol); 2: Palmitic acid; 3: Stearic acid; 4: Oleic acid; 5: Linoleic acid; 6: Lignoceric acid.

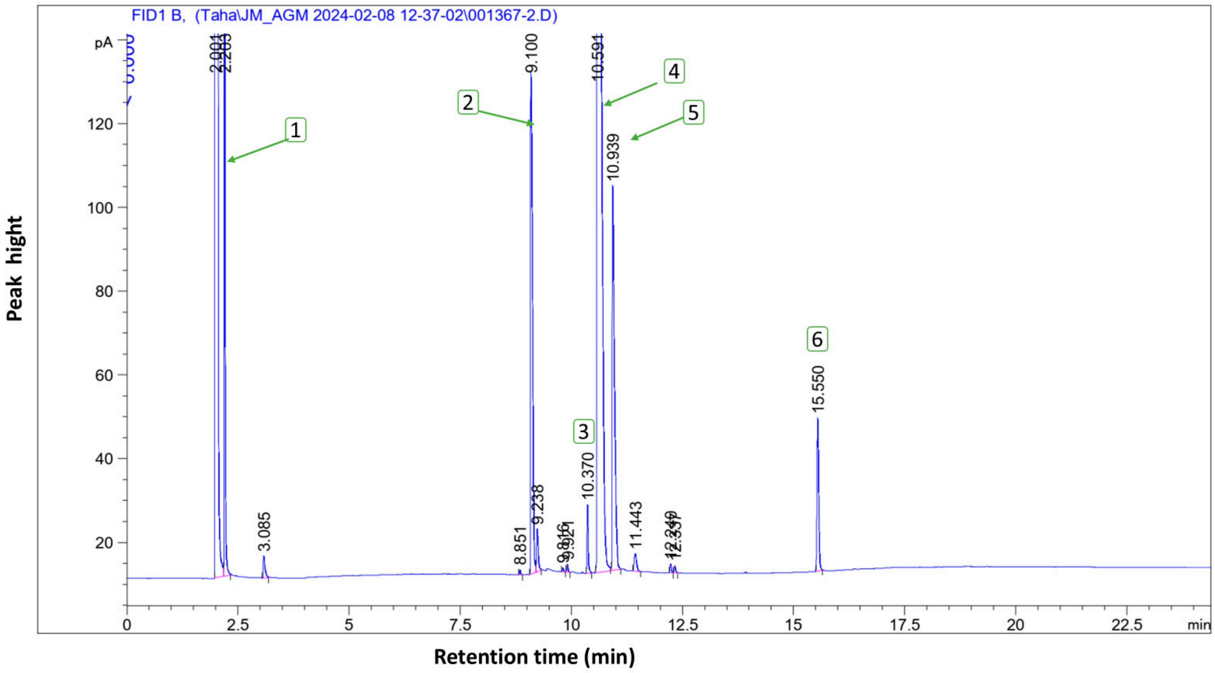

Figure S67. Total ion chromatograms (TICs) from the gas chromatography analysis of the fatty acids in RY_C1: Royuela_ Control 1. (1: Internal standard (4-Methyl-2-pentanol); 2: Palmitic acid; 3: Stearic acid; 4: Oleic acid; 5: Linoleic acid; 6: Lignoceric acid.

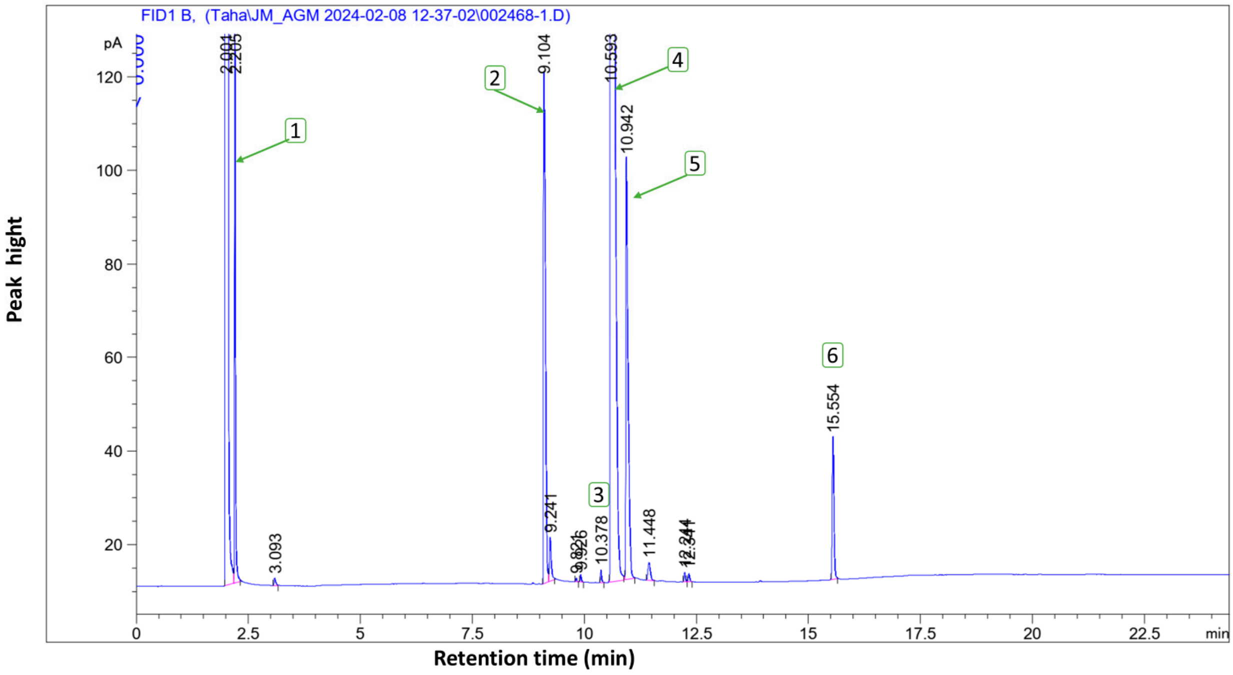

Figure S68. Total ion chromatograms (TICs) from the gas chromatography analysis of the fatty acids in RY_S: Royuela_Supplemented. (1: Internal standard (4-Methyl-2-pentanol); 2: Palmitic acid; 3: Stearic acid; 4: Oleic acid; 5: Linoleic acid; 6: Lignoceric acid.

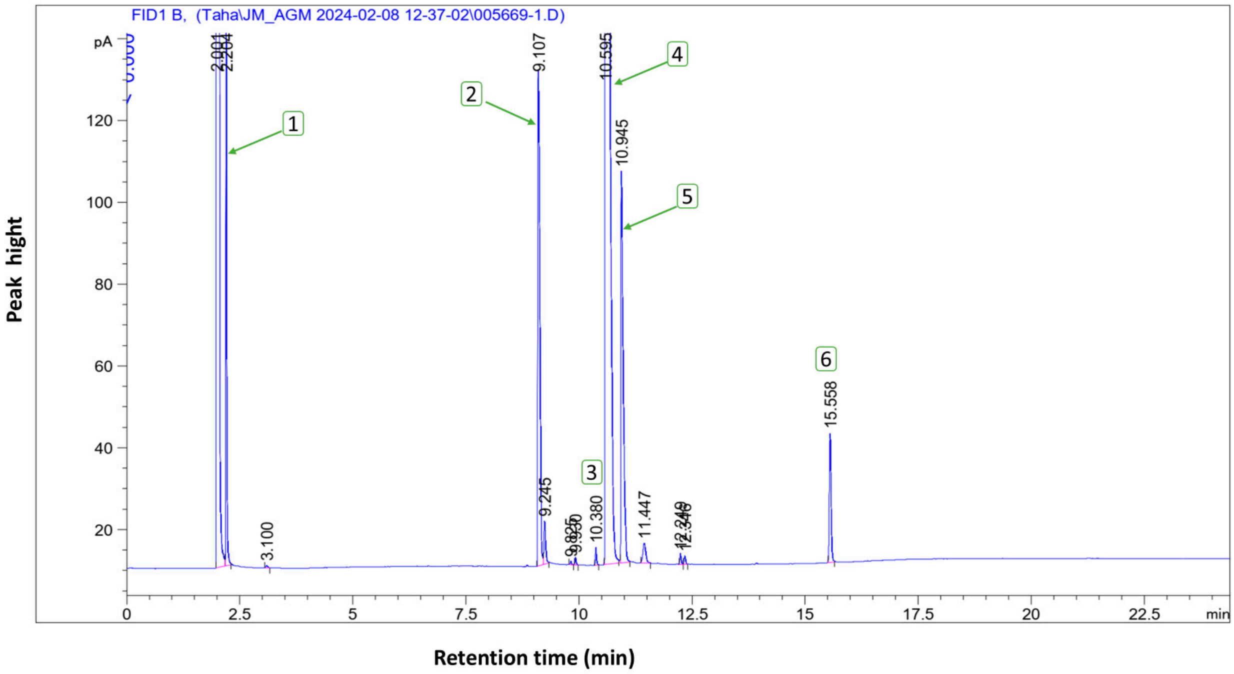

Figure 69. Total ion chromatograms (TICs) from the gas chromatography analysis of the fatty acids in RY_C2: Royuela_ Control 2. (1: Internal standard (4-Methyl-2-pentanol); 2: Palmitic acid; 3: Stearic acid; 4: Oleic acid; 5: Linoleic acid; 6: Lignoceric acid.

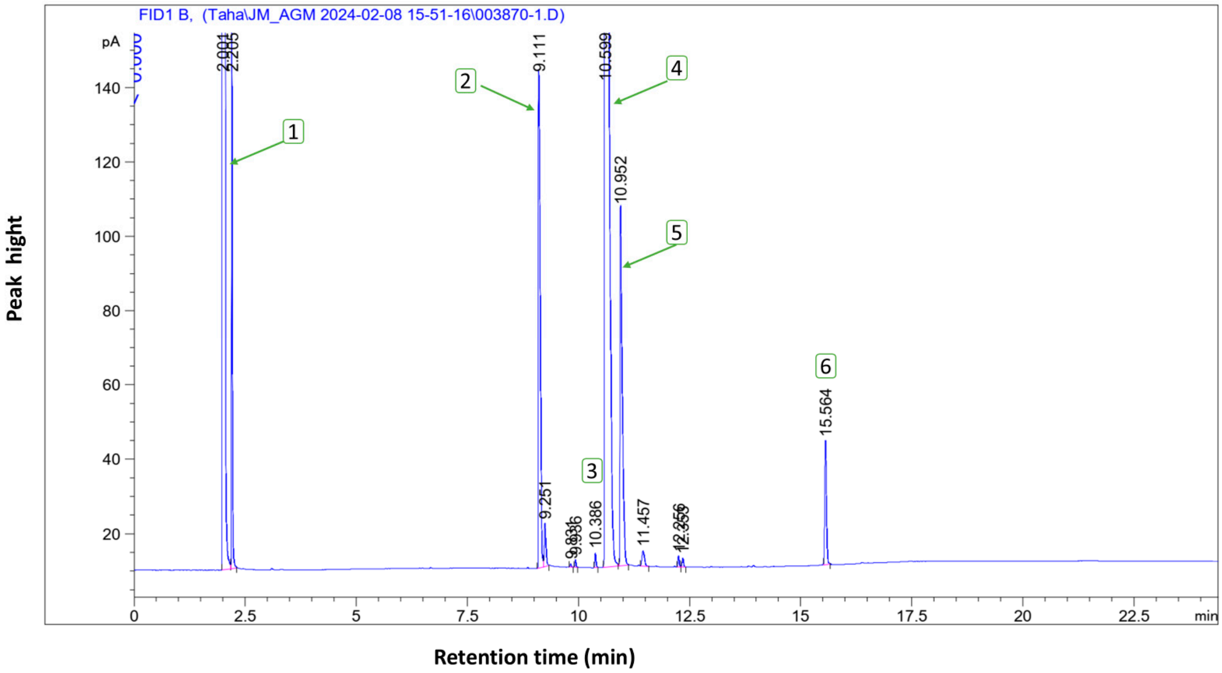

Figure S70. Total ion chromatograms (TICs) from the gas chromatography analysis of the fatty acids in RY_1: Royuela_Exp 1. (1: Internal standard (4-Methyl-2-pentanol); 2: Palmitic acid; 3: Stearic acid; 4: Oleic acid; 5: Linoleic acid; 6: Lignoceric acid.

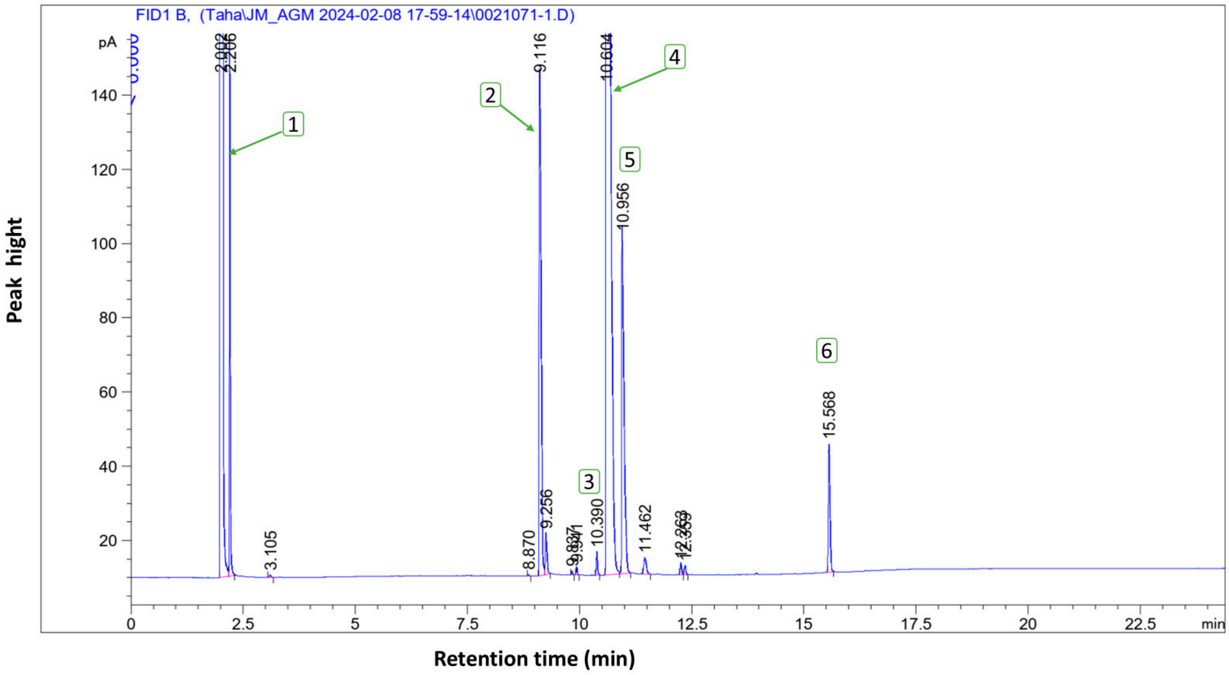

Figure S71. Total ion chromatograms (TICs) from the gas chromatography analysis of the fatty acids in RY_2: Royuela_Exp 2. (1: Internal standard (4-Methyl-2-pentanol); 2: Palmitic acid; 3: Stearic acid; 4: Oleic acid; 5: Linoleic acid; 6: Lignoceric acid.

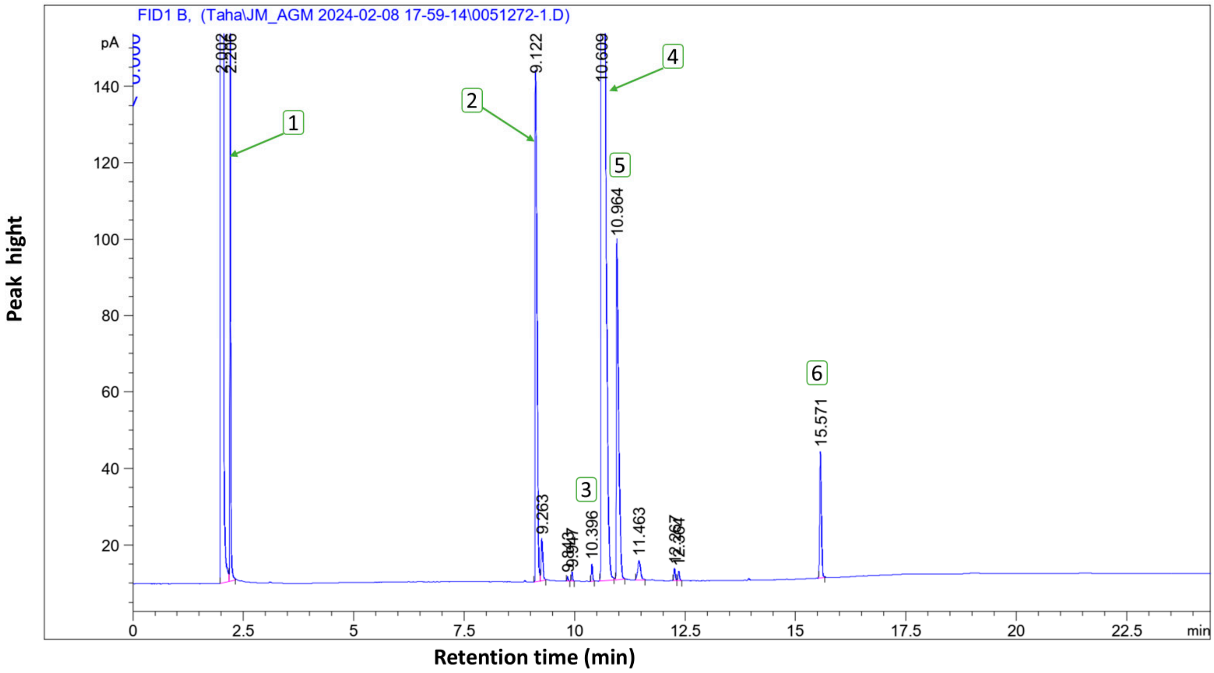

Figure S72. Total ion chromatograms (TICs) from the gas chromatography analysis of the fatty acids in RY_3: Royuela_Exp 3. (1: Internal standard (4-Methyl-2-pentanol); 2: Palmitic acid; 3: Stearic acid; 4: Oleic acid; 5: Linoleic acid; 6: Lignoceric acid.

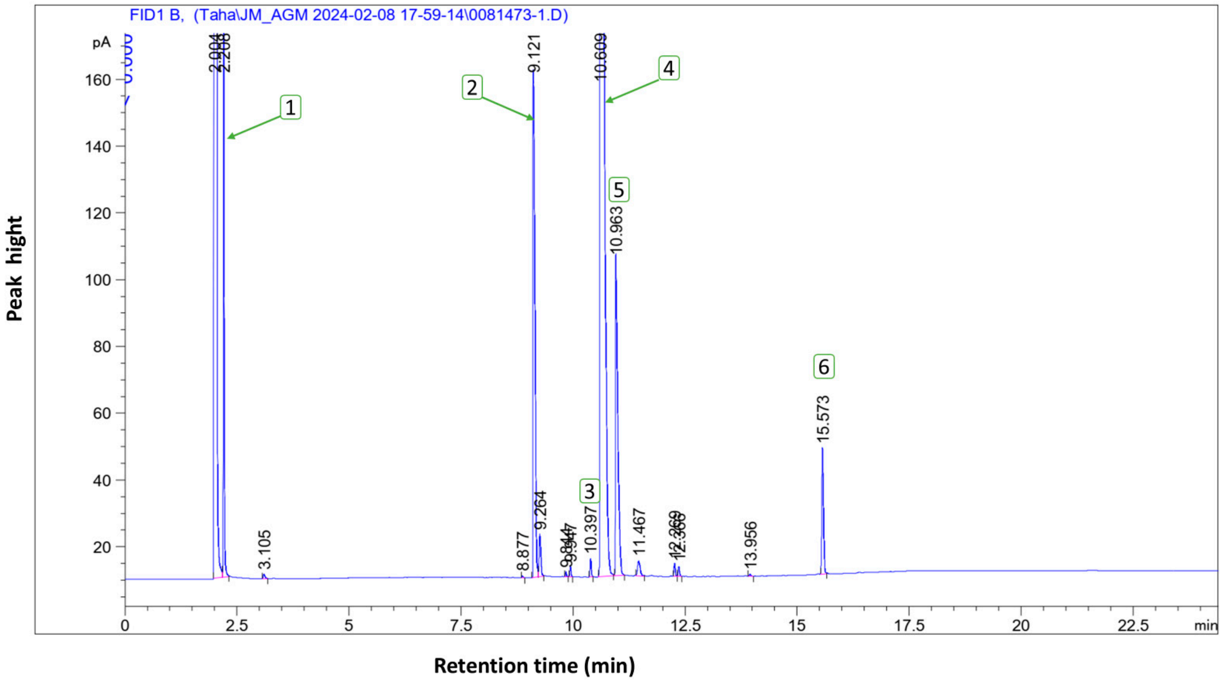

Figure S73. Total ion chromatograms (TICs) from the gas chromatography analysis of the fatty acids in RY_4: Royuela_Exp 4. (1: Internal standard (4-Methyl-2-pentanol); 2: Palmitic acid; 3: Stearic acid; 4: Oleic acid; 5: Linoleic acid; 6: Lignoceric acid.

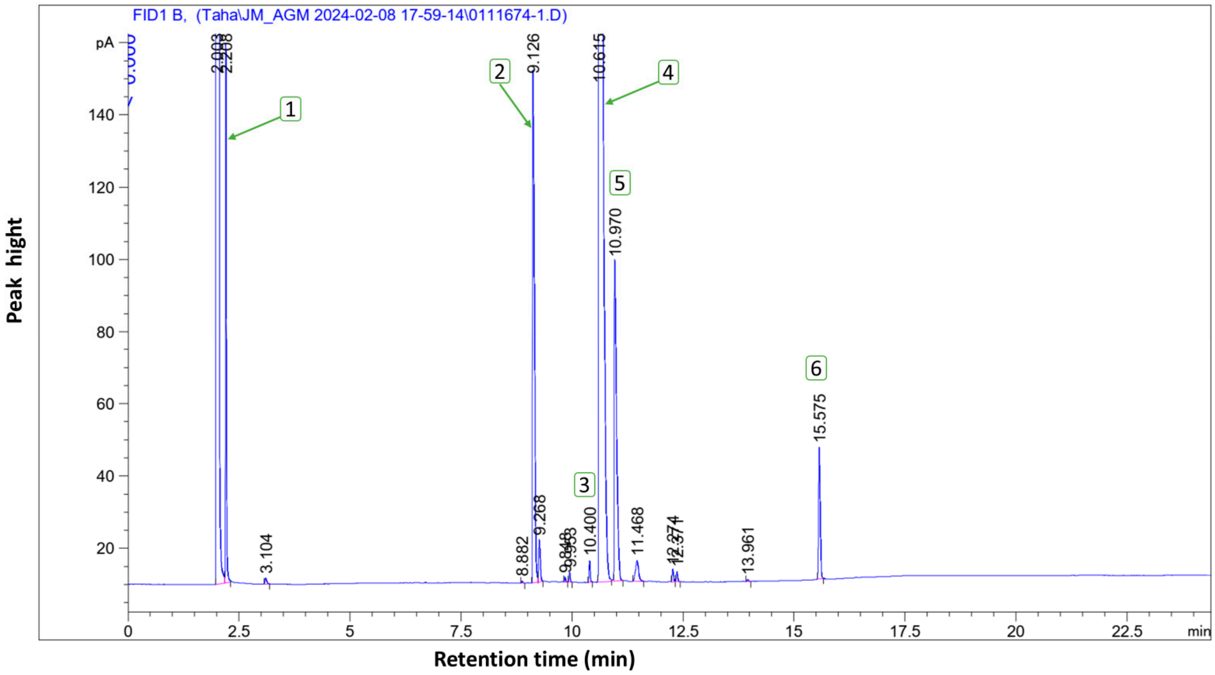

Figure S74. Total ion chromatograms (TICs) from the gas chromatography analysis of the fatty acids in RY_5: Royuela_Exp 5. (1: Internal standard (4-Methyl-2-pentanol); 2: Palmitic acid; 3: Stearic acid; 4: Oleic acid; 5: Linoleic acid; 6: Lignoceric acid.

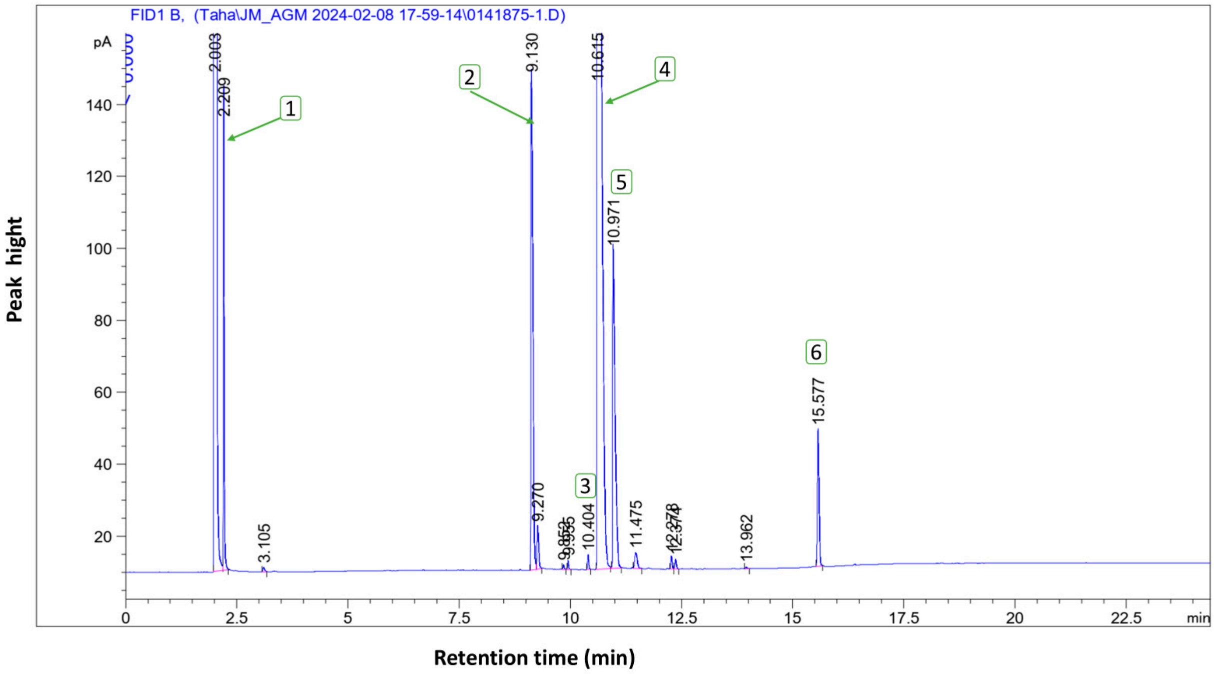

Figure S75. Total ion chromatograms (TICs) from the gas chromatography analysis of the fatty acids in RY_6: Royuela_Exp 6. (1: Internal standard (4-Methyl-2-pentanol); 2: Palmitic acid; 3: Stearic acid; 4: Oleic acid; 5: Linoleic acid; 6: Lignoceric acid.

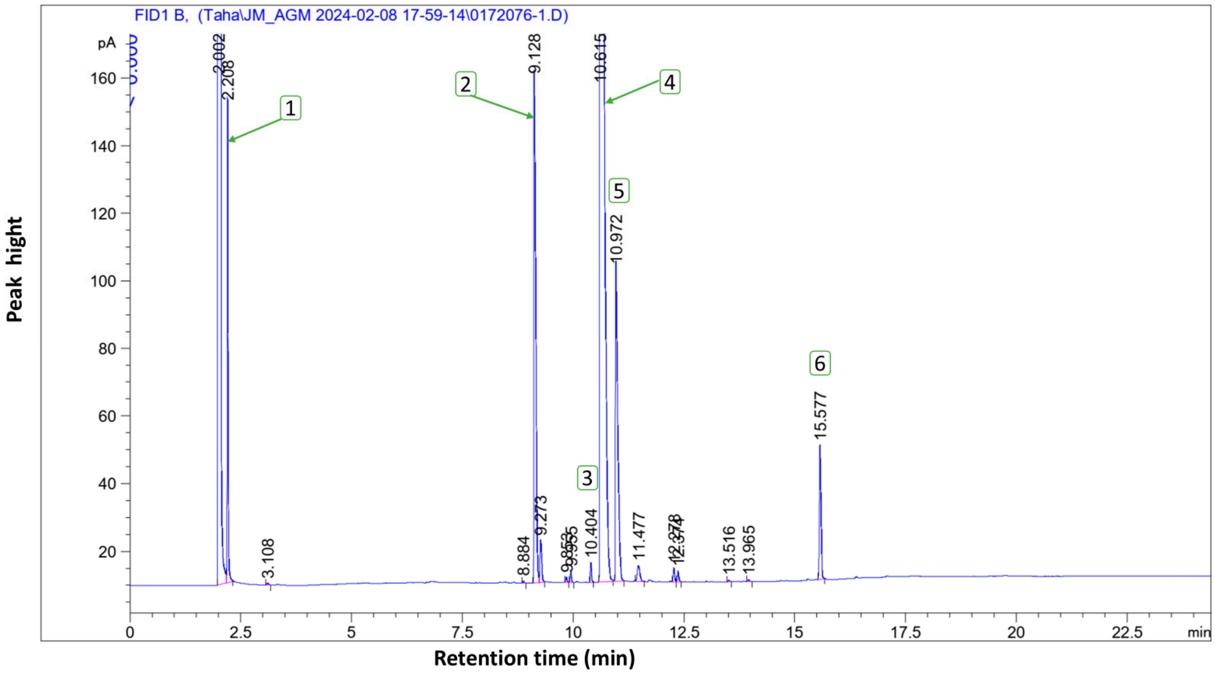

Figure S76. Total ion chromatograms (TICs) from the gas chromatography analysis of the fatty acids in RY_7: Royuela_Exp 7. (1: Internal standard (4-Methyl-2-pentanol); 2: Palmitic acid; 3: Stearic acid; 4: Oleic acid; 5: Linoleic acid; 6: Lignoceric acid.

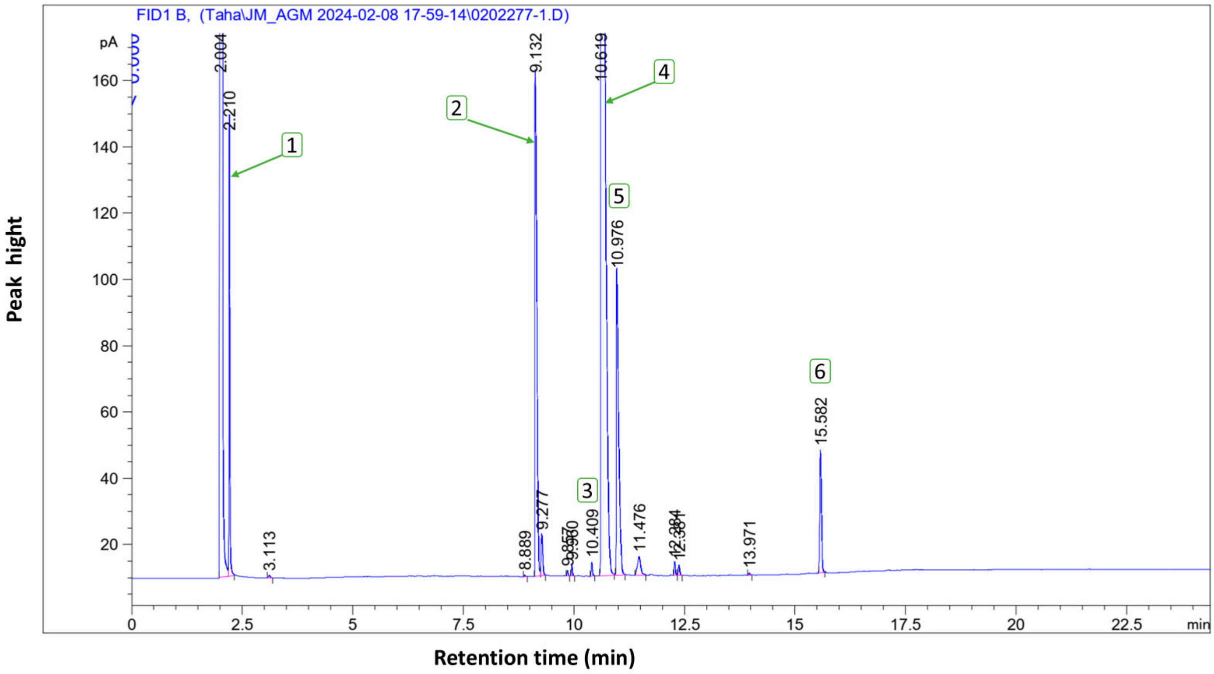

Figure S77. Total ion chromatograms (TICs) from the gas chromatography analysis of the fatty acids in RY_8: Royuela_Exp 8. (1: Internal standard (4-Methyl-2-pentanol); 2: Palmitic acid; 3: Stearic acid; 4: Oleic acid; 5: Linoleic acid; 6: Lignoceric acid.

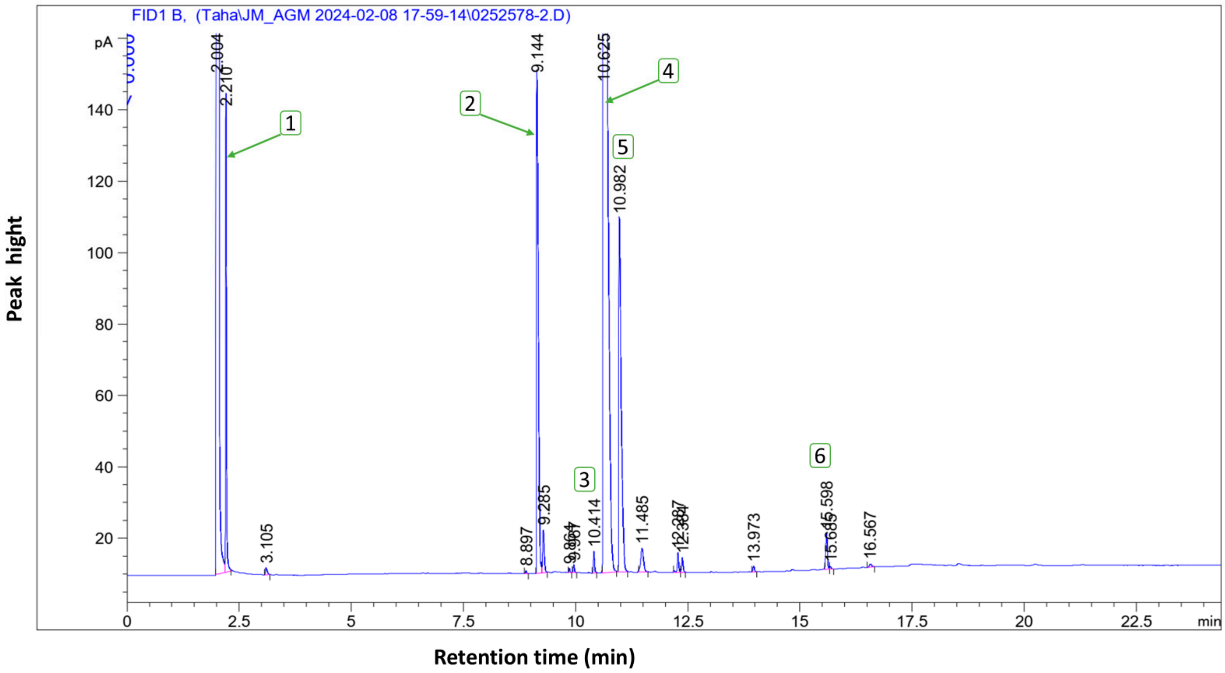

Figure S78. Total ion chromatograms (TICs) from the gas chromatography analysis of the fatty acids in OJ_C1: Orujo_Control 1. (1: Internal standard (4-Methyl-2-pentanol); 2: Palmitic acid; 3: Stearic acid; 4: Oleic acid; 5: Linoleic acid; 6: Lignoceric acid.

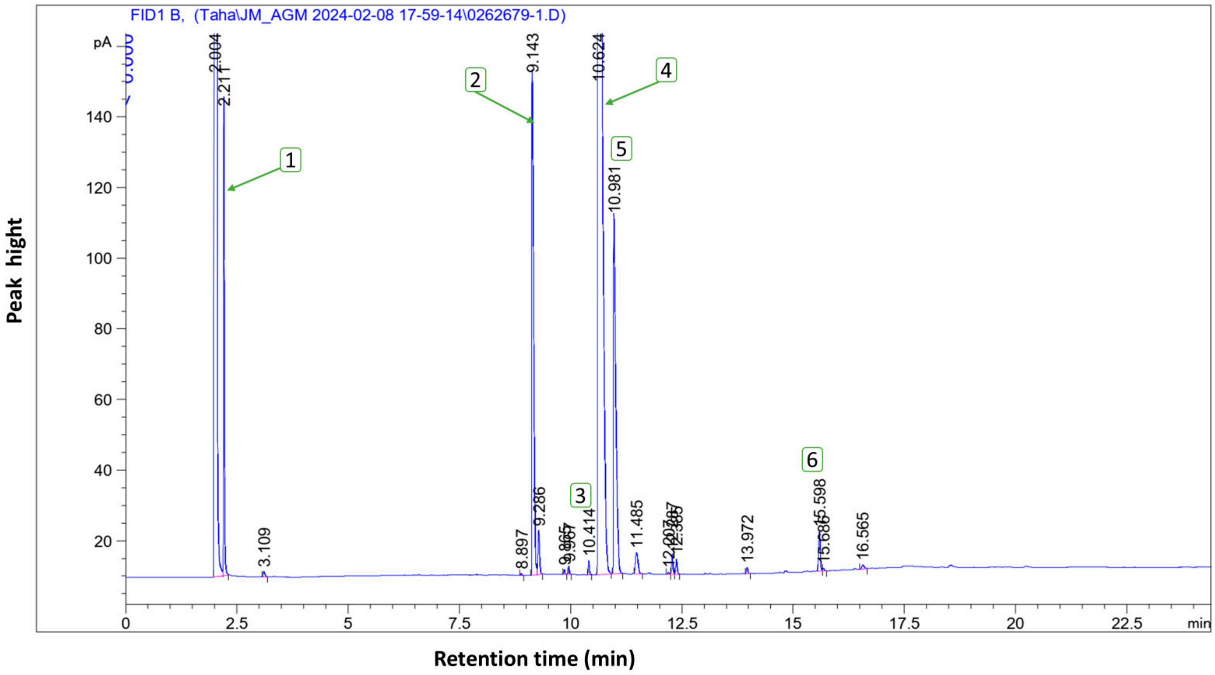

Figure S79. Total ion chromatograms (TICs) from the gas chromatography analysis of the fatty acids in OJ_S: Orujo_ Supplemented. (1: Internal standard (4-Methyl-2-pentanol); 2: Palmitic acid; 3: Stearic acid; 4: Oleic acid; 5: Linoleic acid; 6: Lignoceric acid.

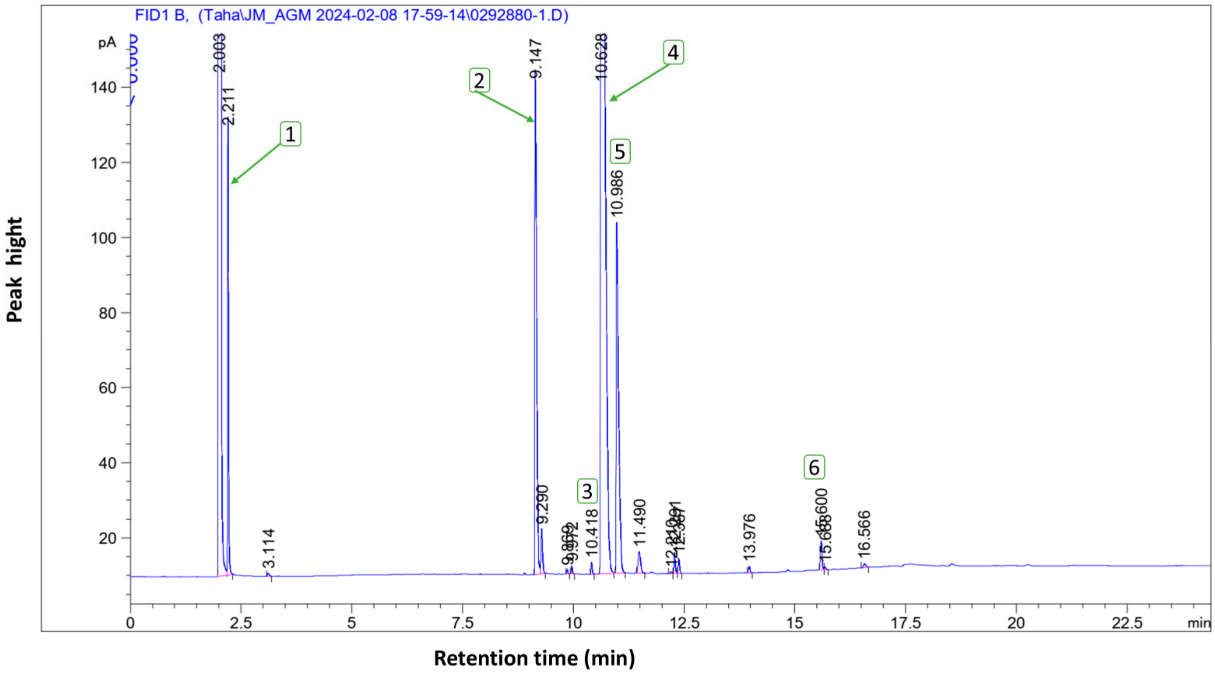

Figure S80. Total ion chromatograms (TICs) from the gas chromatography analysis of the fatty acids in OJ_C2: Orujo_ Control 2. (1: Internal standard (4-Methyl-2-pentanol); 2: Palmitic acid; 3: Stearic acid; 4: Oleic acid; 5: Linoleic acid; 6: Lignoceric acid.

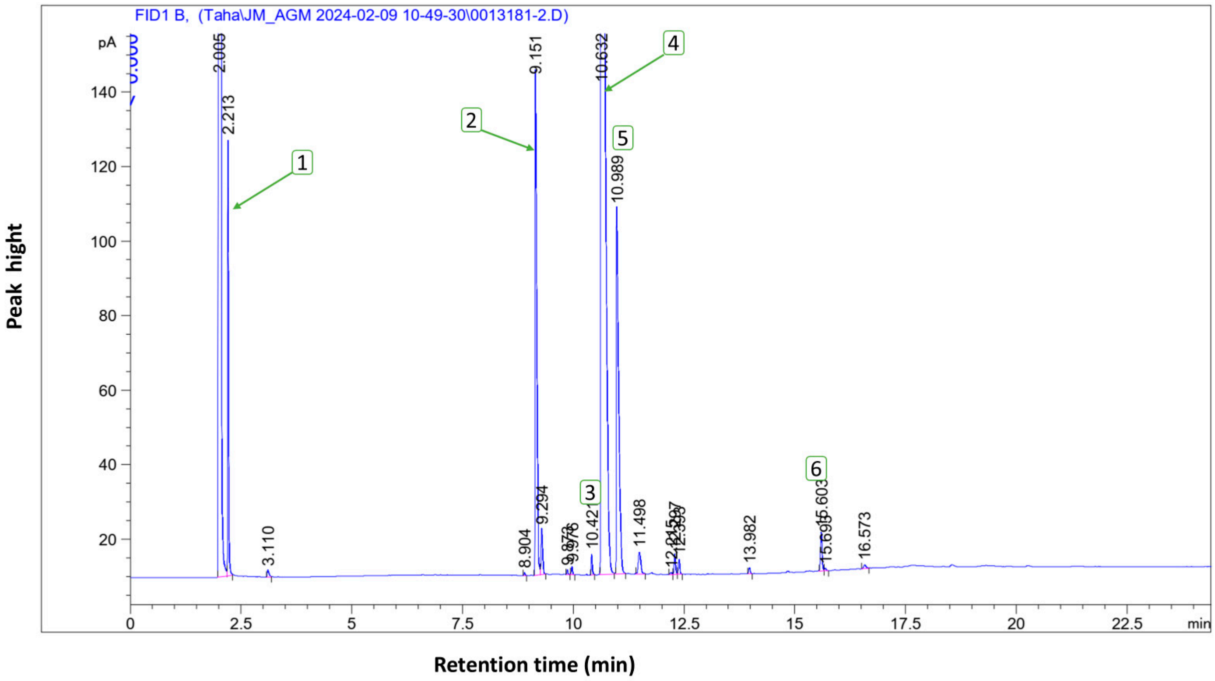

Figure S81. Total ion chromatograms (TICs) from the gas chromatography analysis of the fatty acids in OJ_1: Orujo_Exp 1. (1: Internal standard (4-Methyl-2-pentanol); 2: Palmitic acid; 3: Stearic acid; 4: Oleic acid; 5: Linoleic acid; 6: Lignoceric acid.

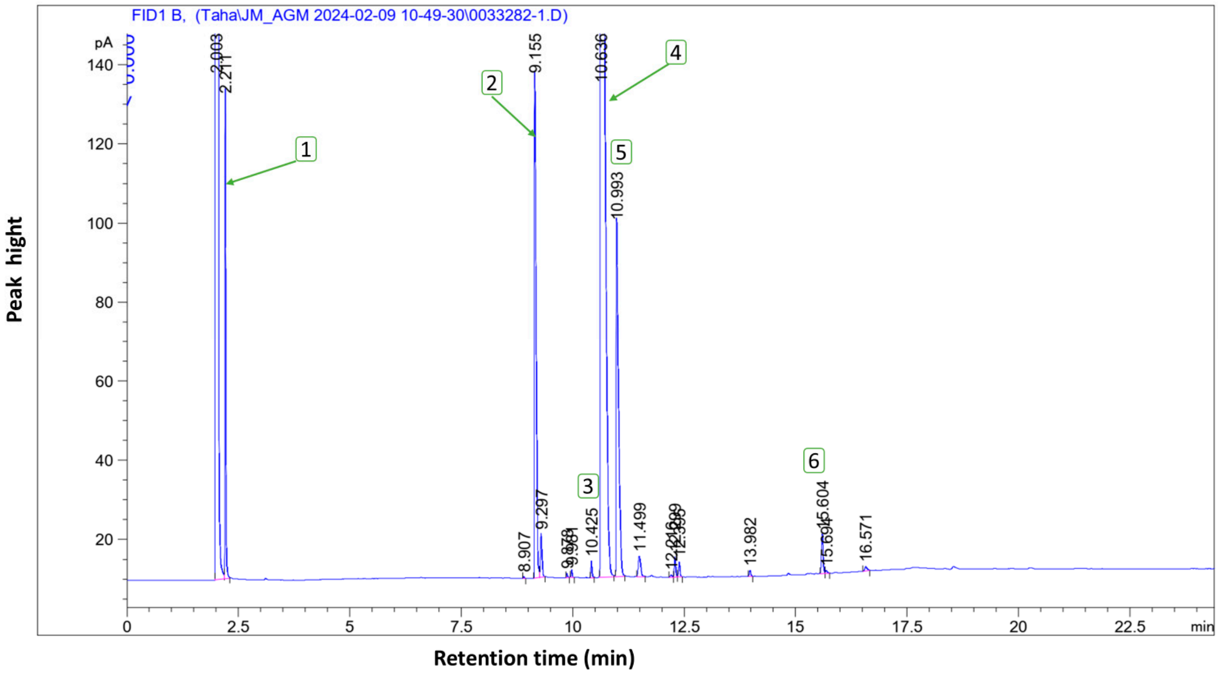

Figure S82. Total ion chromatograms (TICs) from the gas chromatography analysis of the fatty acids in OJ_2: Orujo_Exp 2. (1: Internal standard (4-Methyl-2-pentanol); 2: Palmitic acid; 3: Stearic acid; 4: Oleic acid; 5: Linoleic acid; 6: Lignoceric acid.

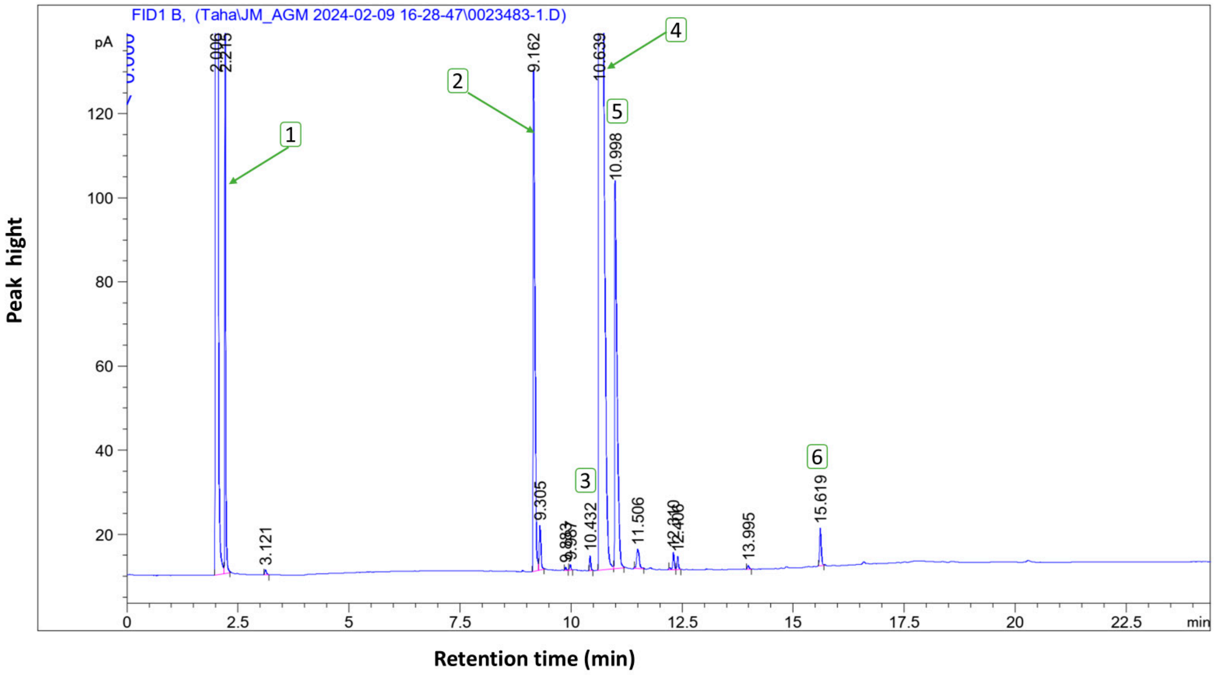

Figure S83. Total ion chromatograms (TICs) from the gas chromatography analysis of the fatty acids in OJ_3: Orujo_Exp 3. (1: Internal standard (4-Methyl-2-pentanol); 2: Palmitic acid; 3: Stearic acid; 4: Oleic acid; 5: Linoleic acid; 6: Lignoceric acid.

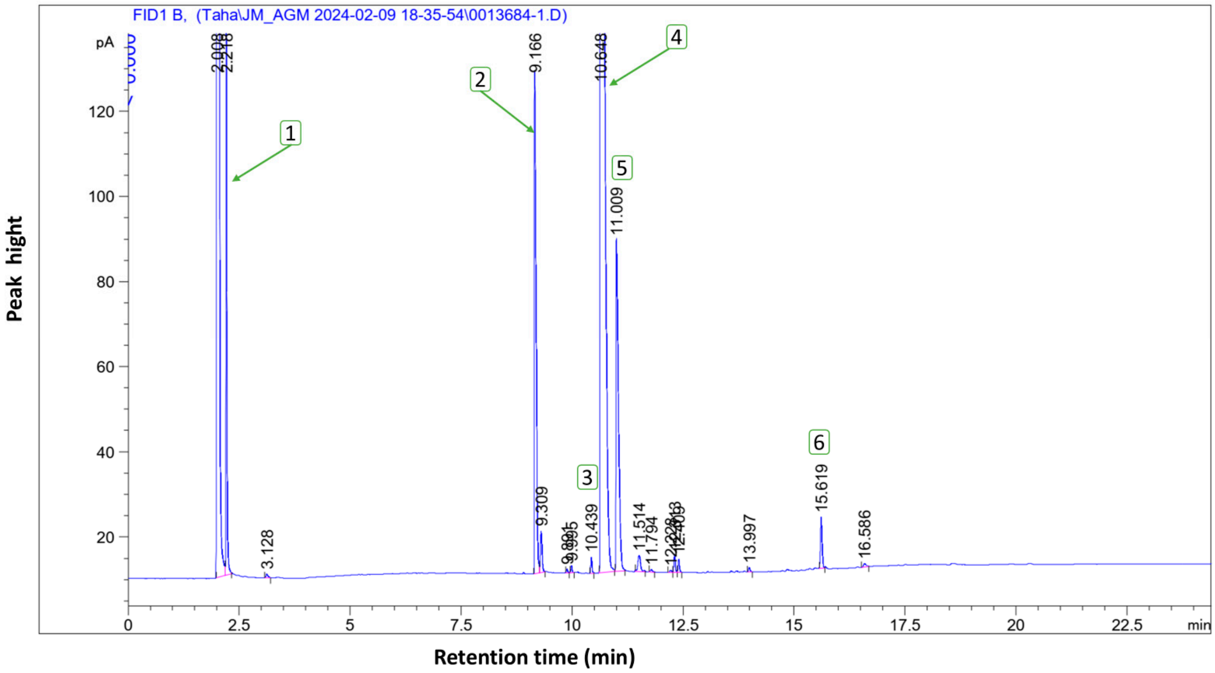

Figure S84. Total ion chromatograms (TICs) from the gas chromatography analysis of the fatty acids in OJ_4: Orujo_Exp 4. (1: Internal standard (4-Methyl-2-pentanol); 2: Palmitic acid; 3: Stearic acid; 4: Oleic acid; 5: Linoleic acid; 6: Lignoceric acid.

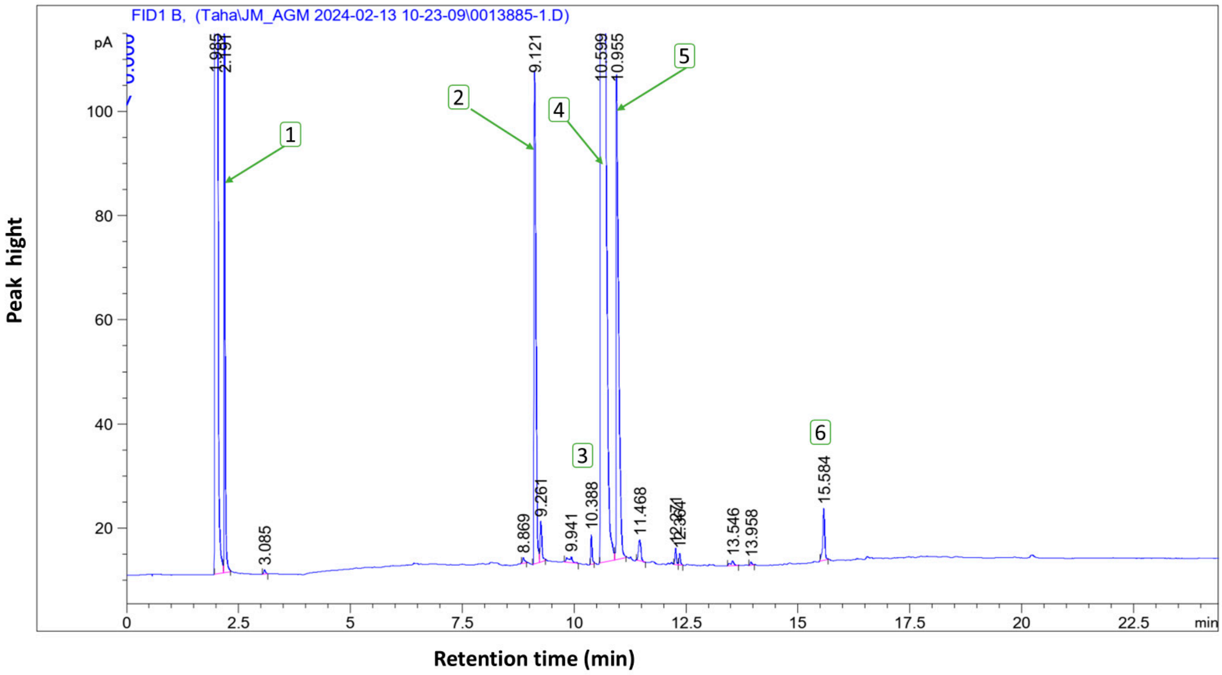

Figure S85. Total ion chromatograms (TICs) from the gas chromatography analysis of the fatty acids in OJ_5: Orujo_Exp 5. (1: Internal standard (4-Methyl-2-pentanol); 2: Palmitic acid; 3: Stearic acid; 4: Oleic acid; 5: Linoleic acid; 6: Lignoceric acid.

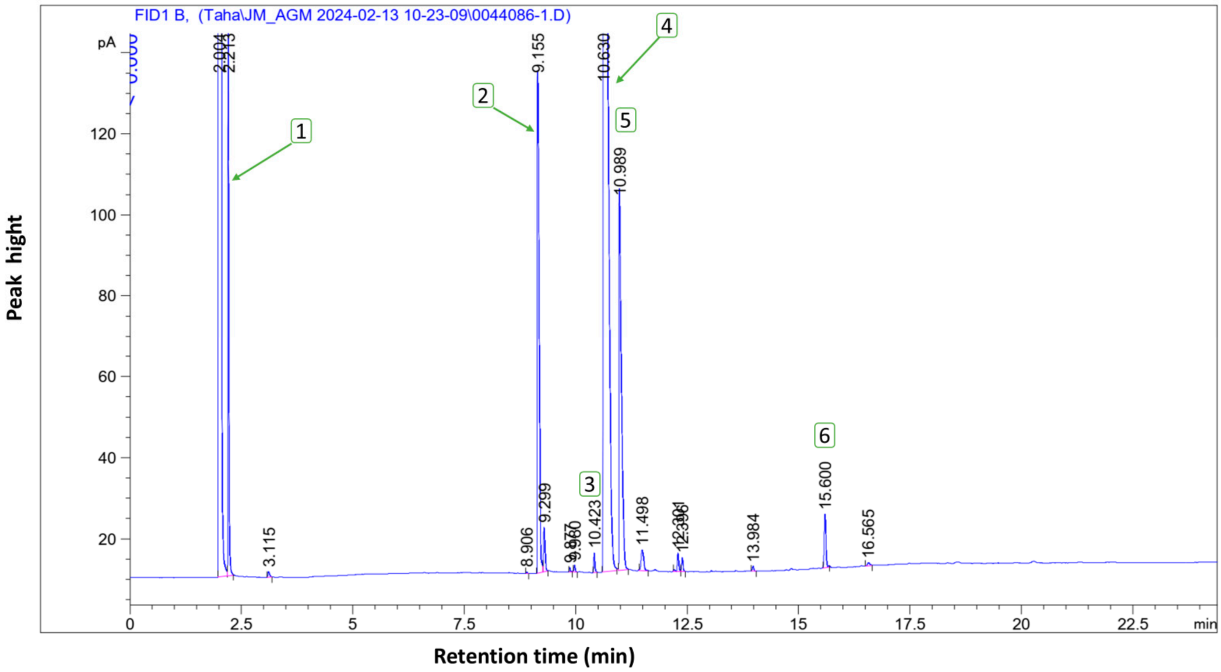

Figure S86. Total ion chromatograms (TICs) from the gas chromatography analysis of the fatty acids in OJ_6: Orujo_Exp 6. (1: Internal standard (4-Methyl-2-pentanol); 2: Palmitic acid; 3: Stearic acid; 4: Oleic acid; 5: Linoleic acid; 6: Lignoceric acid.

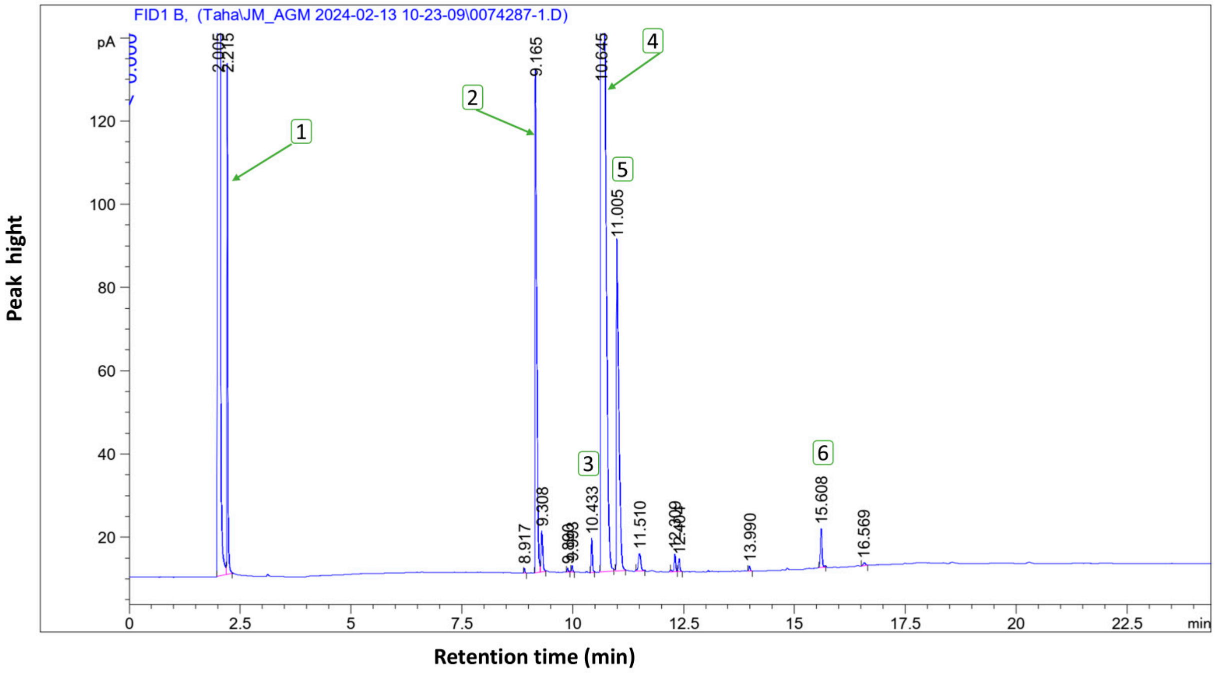

Figure S87. Total ion chromatograms (TICs) from the gas chromatography analysis of the fatty acids in OJ_7: Orujo_Exp 7. (1: Internal standard (4-Methyl-2-pentanol); 2: Palmitic acid; 3: Stearic acid; 4: Oleic acid; 5: Linoleic acid; 6: Lignoceric acid.

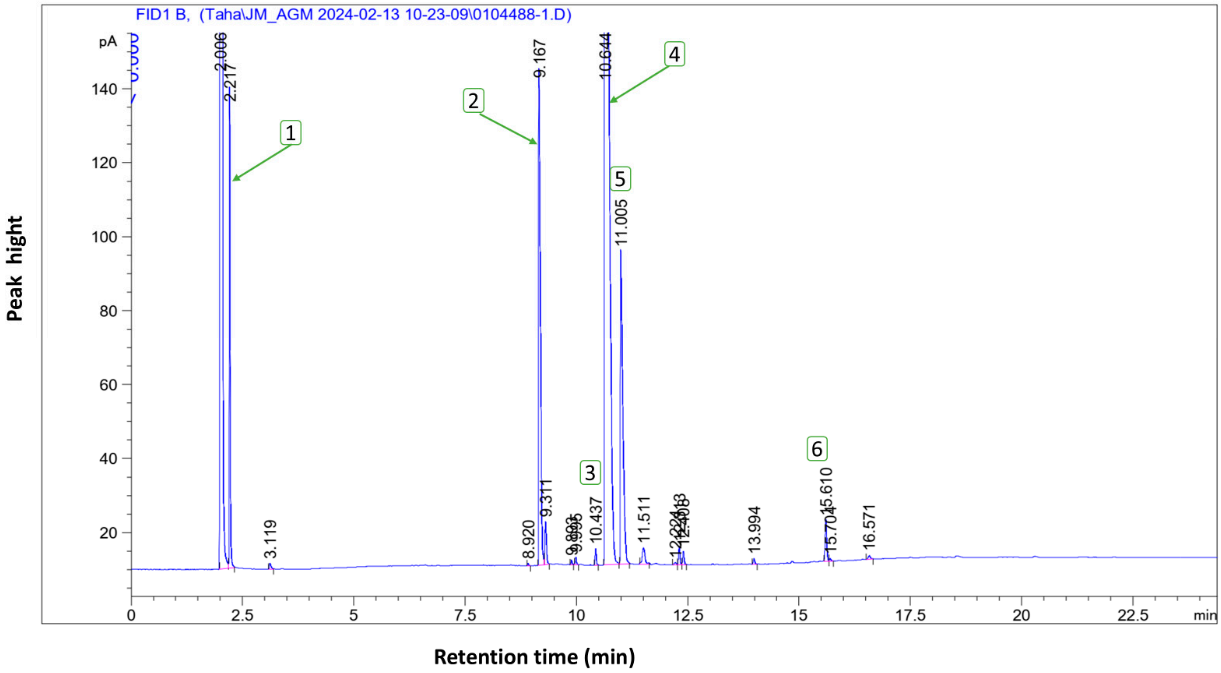

Figure S88. Total ion chromatograms (TICs) from the gas chromatography analysis of the fatty acids in OJ_8: Orujo_Exp 8. (1: Internal standard (4-Methyl-2-pentanol); 2: Palmitic acid; 3: Stearic acid; 4: Oleic acid; 5: Linoleic acid; 6: Lignoceric acid.

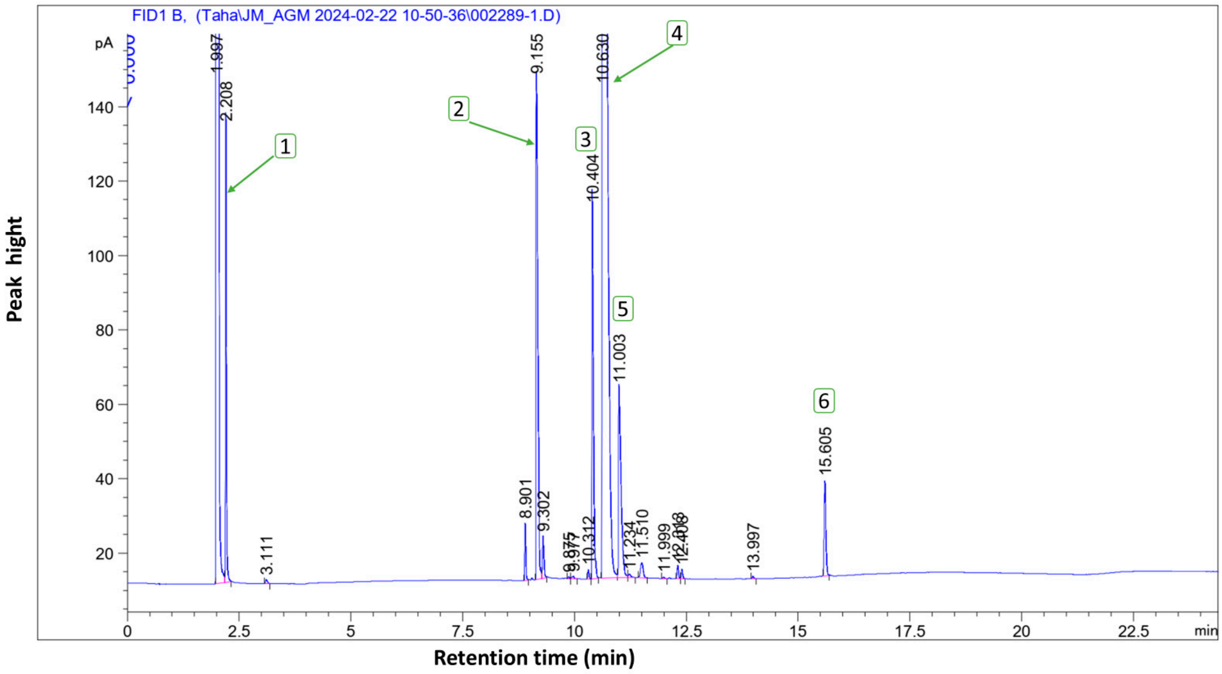

Figure S89. Total ion chromatograms (TICs) from the gas chromatography analysis of the fatty acids in KN_C1: Koroneiki_Control 1. (1: Internal standard (4-Methyl-2-pentanol); 2: Palmitic acid; 3: Stearic acid; 4: Oleic acid; 5: Linoleic acid; 6: Lignoceric acid.

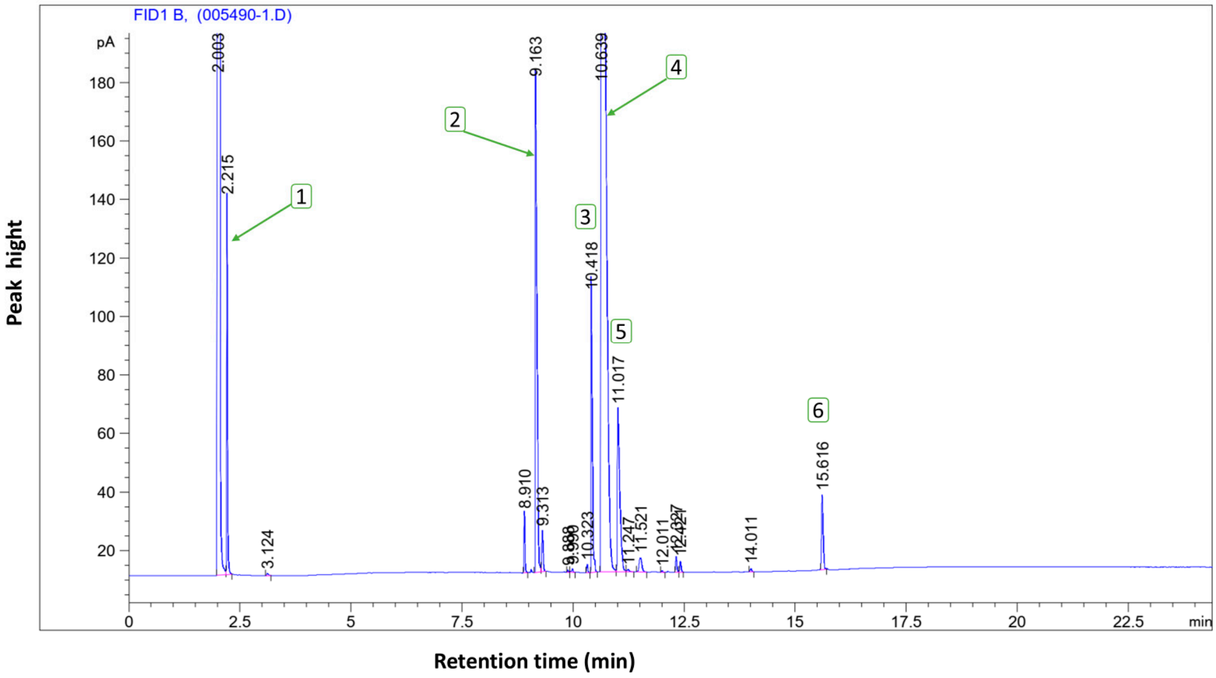

Figure S90. Total ion chromatograms (TICs) from the gas chromatography analysis of the fatty acids in KN_S: Koroneiki_Supplemented. (1: Internal standard (4-Methyl-2-pentanol); 2: Palmitic acid; 3: Stearic acid; 4: Oleic acid; 5: Linoleic acid; 6: Lignoceric acid.

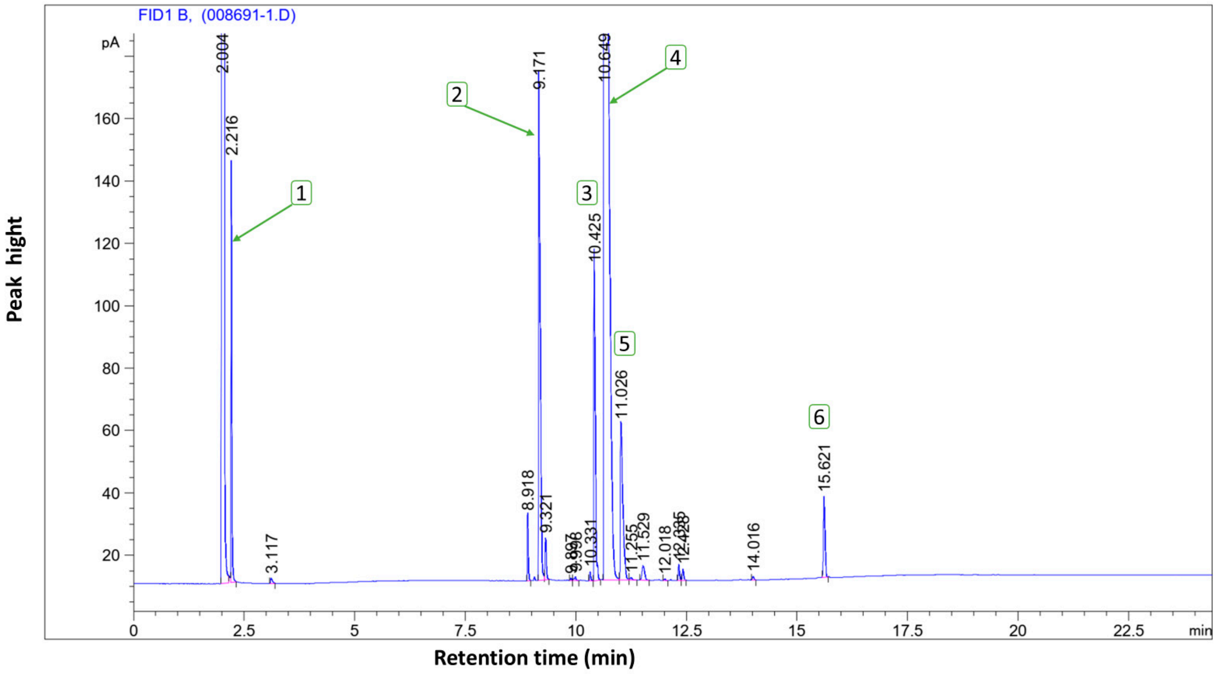

Figure S91. Total ion chromatograms (TICs) from the gas chromatography analysis of the fatty acids in KN_C2: Koroneiki_Control 2. (1: Internal standard (4-Methyl-2-pentanol); 2: Palmitic acid; 3: Stearic acid; 4: Oleic acid; 5: Linoleic acid; 6: Lignoceric acid.

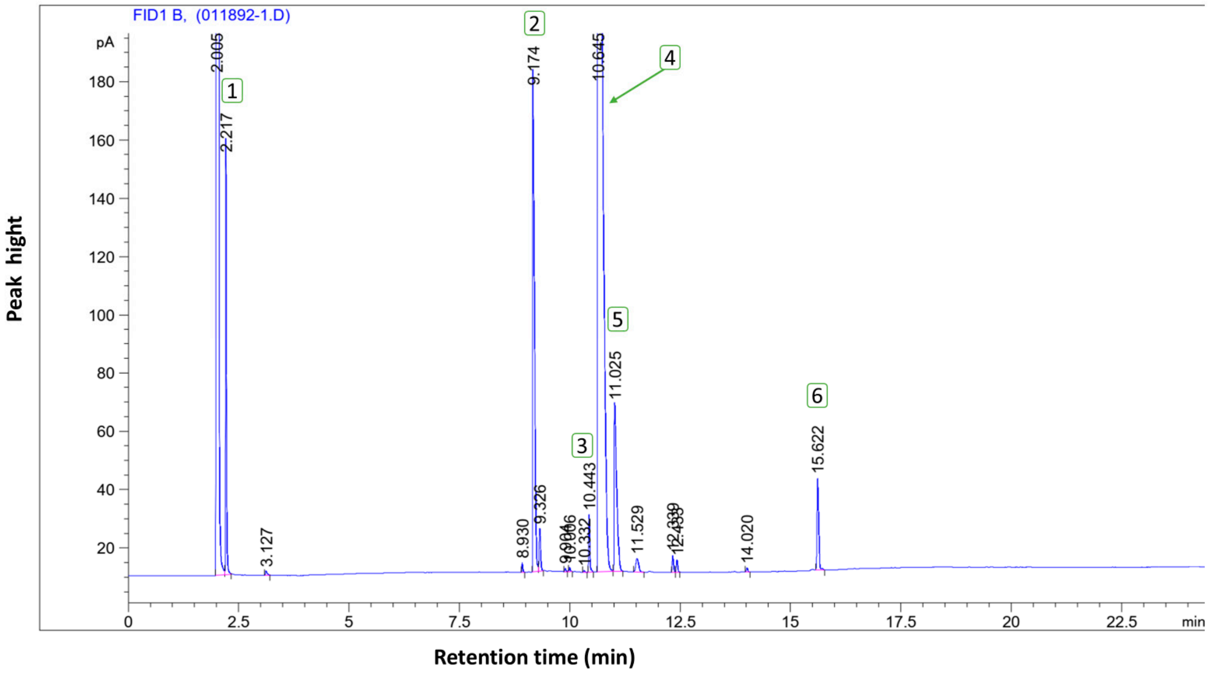

Figure S92. Total ion chromatograms (TICs) from the gas chromatography analysis of the fatty acids in KN_1: Koroneiki_Exp 1. (1: Internal standard (4-Methyl-2-pentanol); 2: Palmitic acid; 3: Stearic acid; 4: Oleic acid; 5: Linoleic acid; 6: Lignoceric acid.

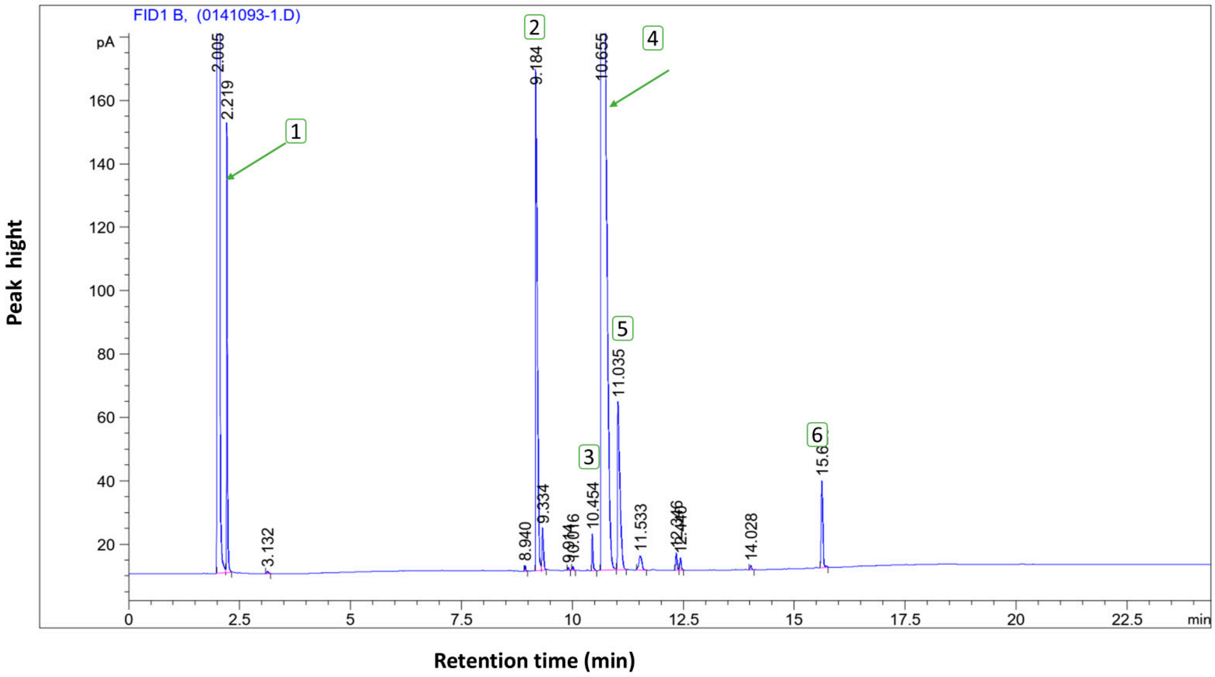

Figure S93. Total ion chromatograms (TICs) from the gas chromatography analysis of the fatty acids in KN_2: Koroneiki_Exp 2. (1: Internal standard (4-Methyl-2-pentanol); 2: Palmitic acid; 3: Stearic acid; 4: Oleic acid; 5: Linoleic acid; 6: Lignoceric acid.

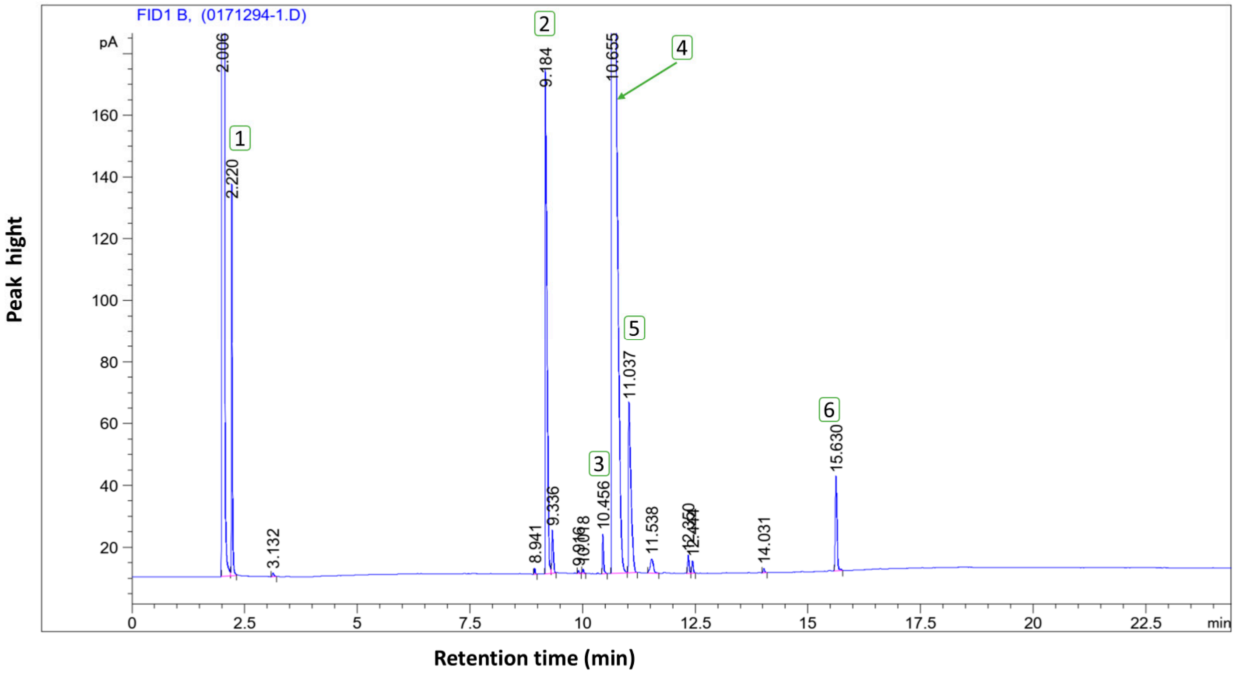

Figure S94. Total ion chromatograms (TICs) from the gas chromatography analysis of the fatty acids in KN_3: Koroneiki_Exp 3. (1: Internal standard (4-Methyl-2-pentanol); 2: Palmitic acid; 3: Stearic acid; 4: Oleic acid; 5: Linoleic acid; 6: Lignoceric acid.

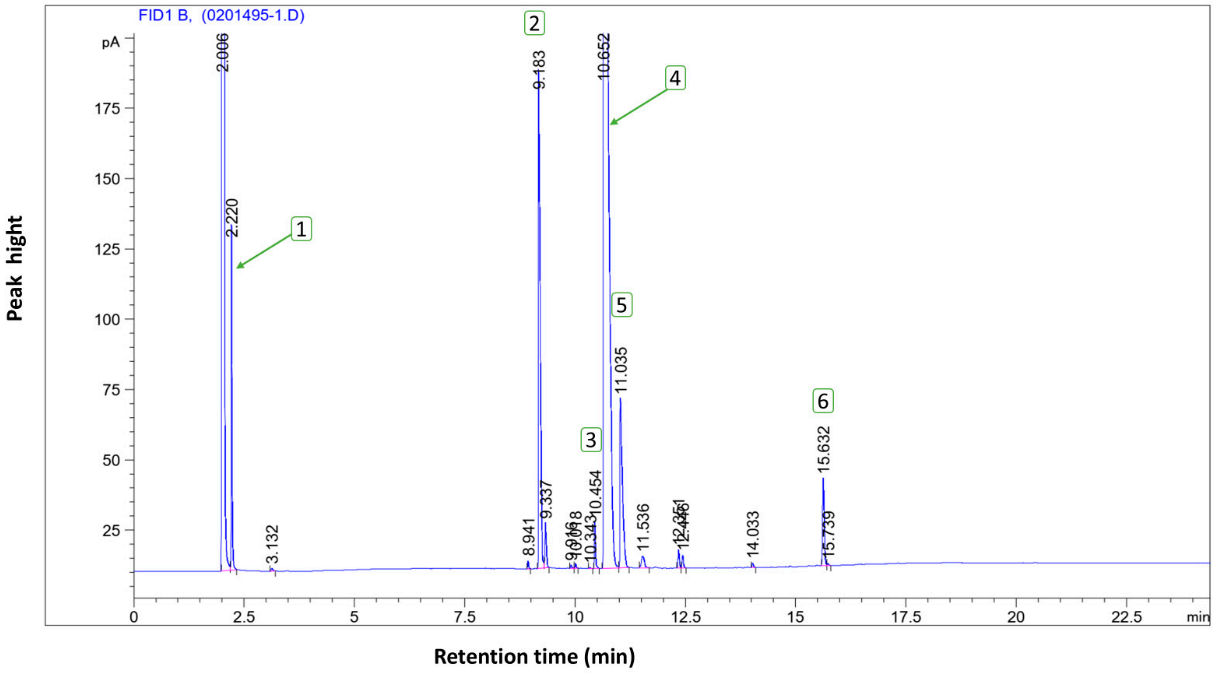

Figure S95. Total ion chromatograms (TICs) from the gas chromatography analysis of the fatty acids in KN_4: Koroneiki_Exp 4. (1: Internal standard (4-Methyl-2-pentanol); 2: Palmitic acid; 3: Stearic acid; 4: Oleic acid; 5: Linoleic acid; 6: Lignoceric acid.

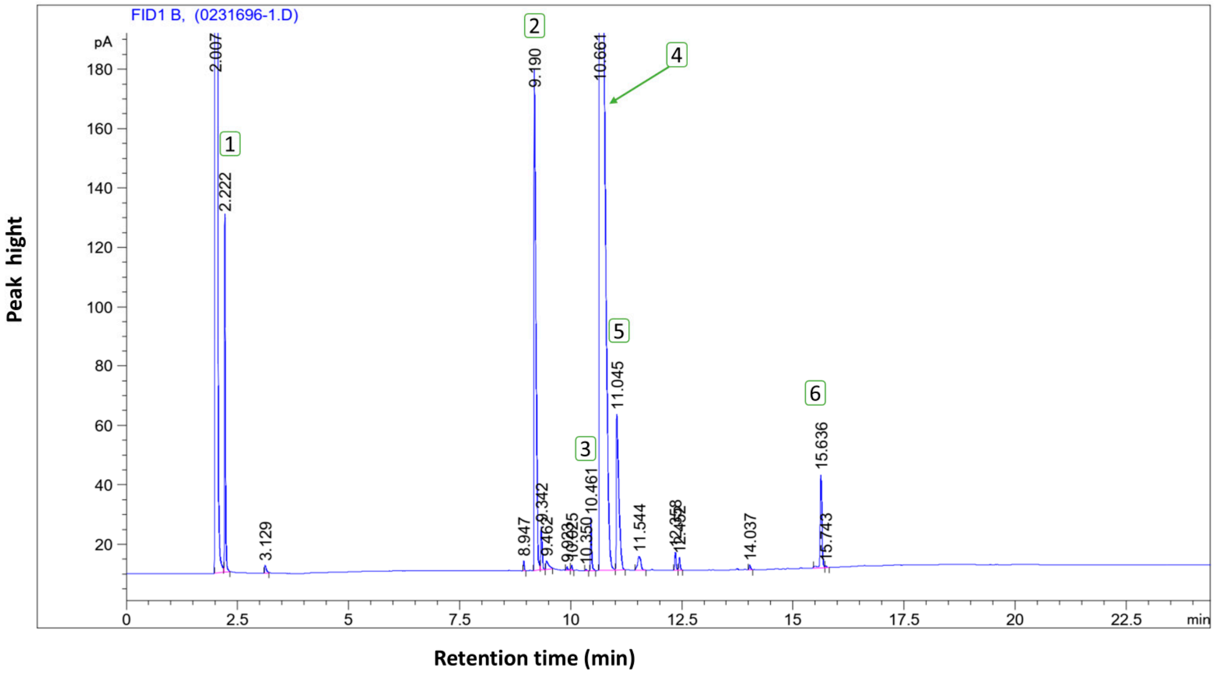

Figure S96. Total ion chromatograms (TICs) from the gas chromatography analysis of the fatty acids in KN_5: Koroneiki_Exp 5. (1: Internal standard (4-Methyl-2-pentanol); 2: Palmitic acid; 3: Stearic acid; 4: Oleic acid; 5: Linoleic acid; 6: Lignoceric acid.

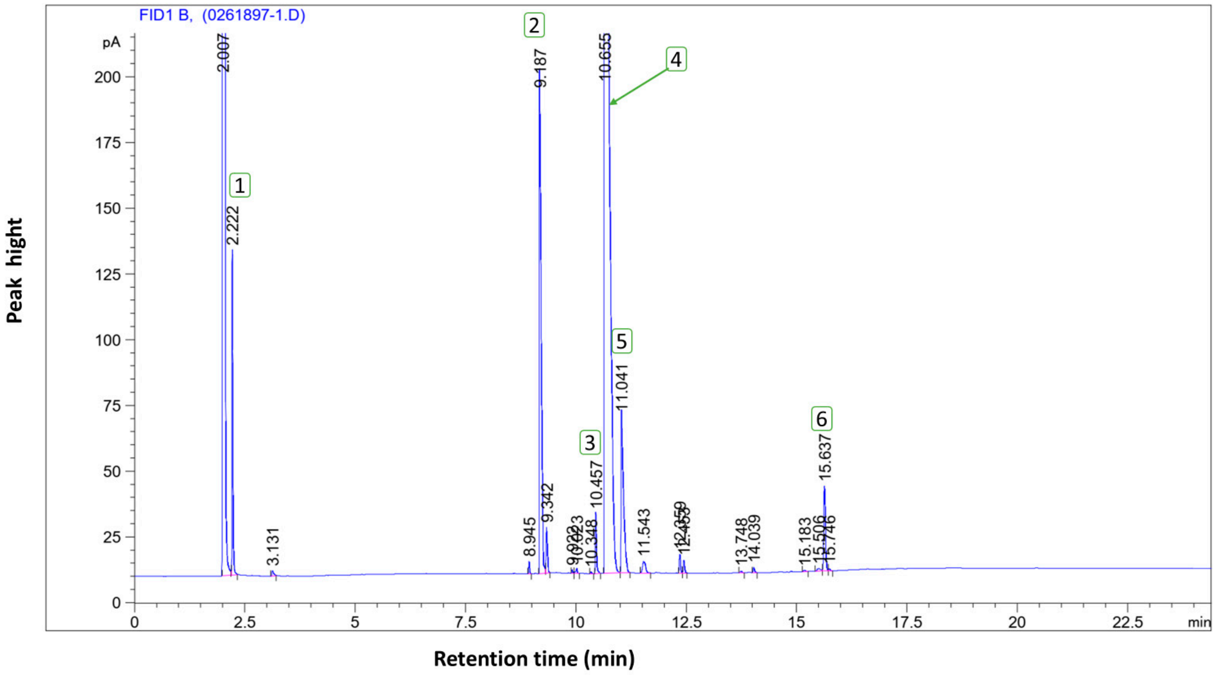

Figure S97. Total ion chromatograms (TICs) from the gas chromatography analysis of the fatty acids in KN_6: Koroneiki_Exp 6. (1: Internal standard (4-Methyl-2-pentanol); 2: Palmitic acid; 3: Stearic acid; 4: Oleic acid; 5: Linoleic acid; 6: Lignoceric acid.

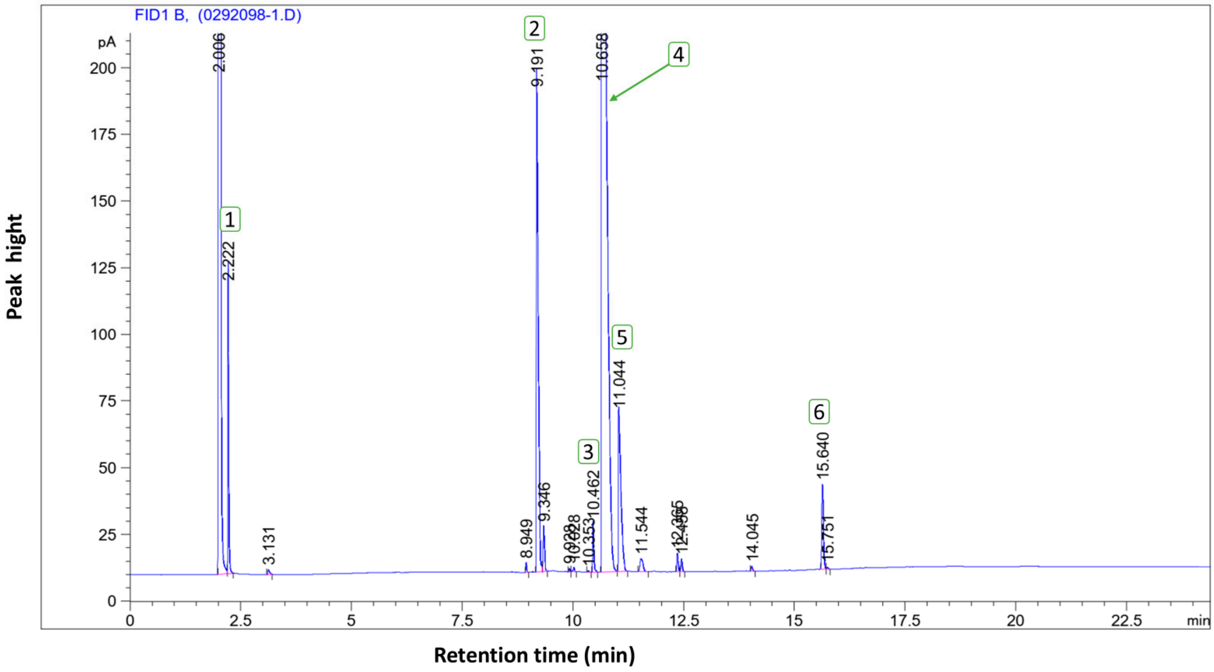

Figure S98. Total ion chromatograms (TICs) from the gas chromatography analysis of the fatty acids in KN_7: Koroneiki_Exp 7. (1: Internal standard (4-Methyl-2-pentanol); 2: Palmitic acid; 3: Stearic acid; 4: Oleic acid; 5: Linoleic acid; 6: Lignoceric acid.

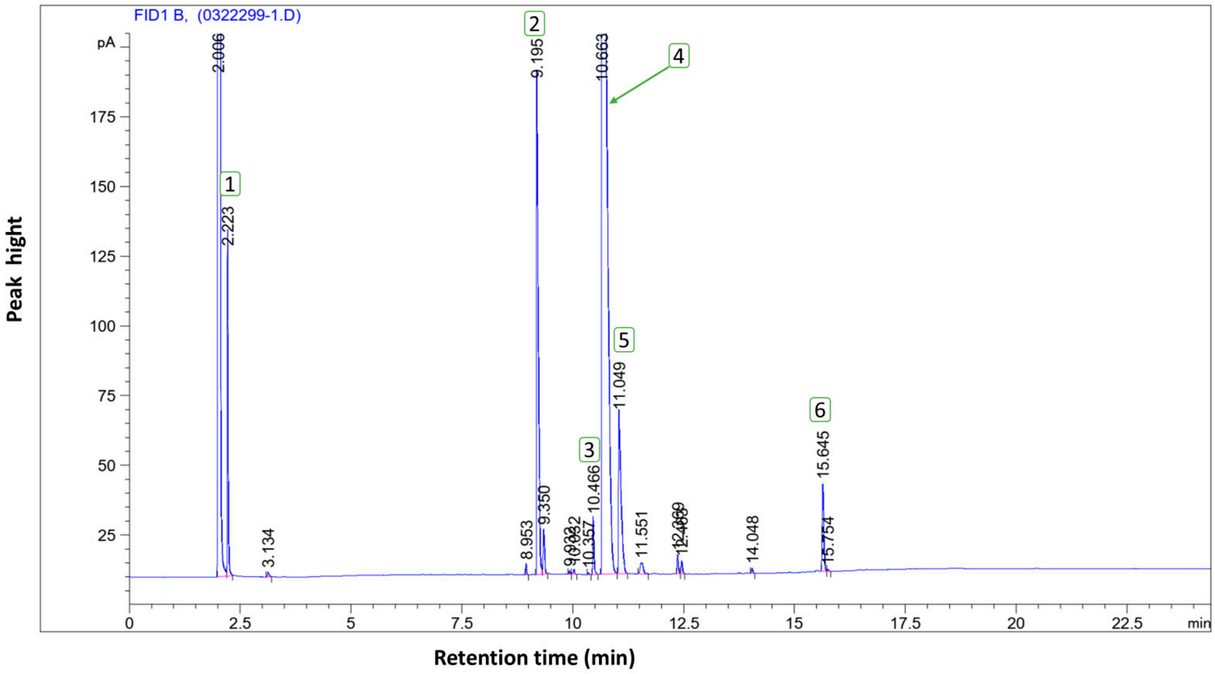

Figure S99. Total ion chromatograms (TICs) from the gas chromatography analysis of the fatty acids in KN_8: Koroneiki_Exp 8. (1: Internal standard (4-Methyl-2-pentanol); 2: Palmitic acid; 3: Stearic acid; 4: Oleic acid; 5: Linoleic acid; 6: Lignoceric acid.

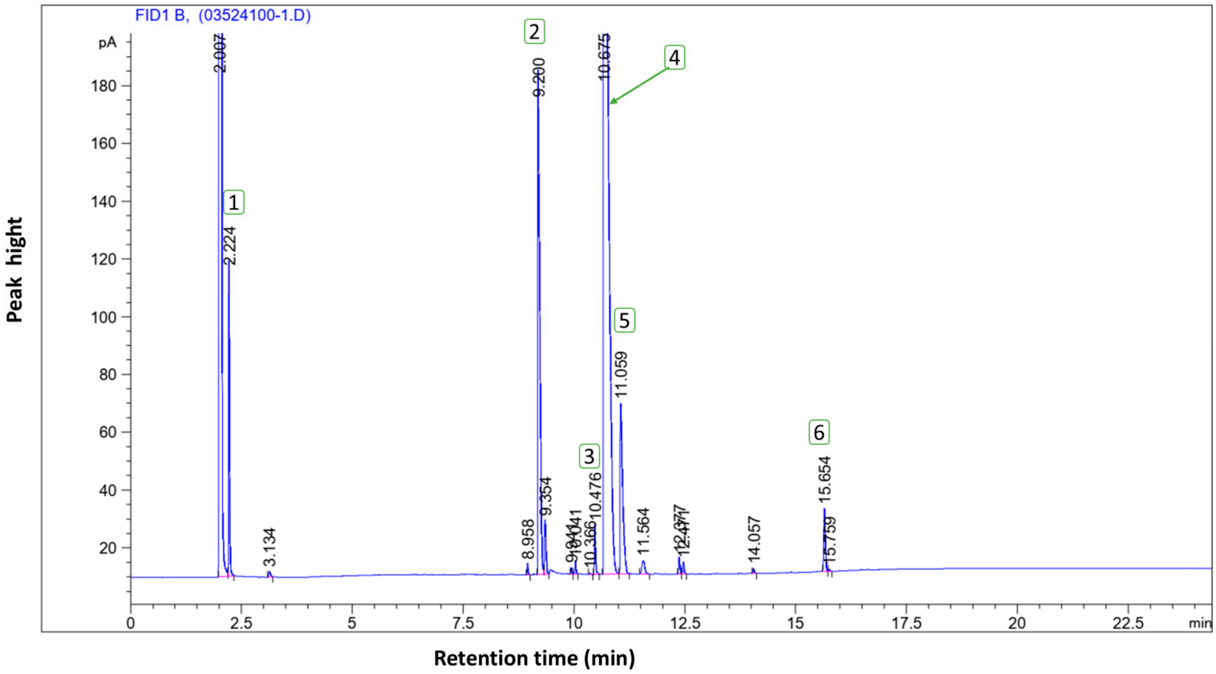

Figure S100. Total ion chromatograms (TICs) from the gas chromatography analysis of the fatty acids in AS_C1: Arbosana_Control 1. (1: Internal standard (4-Methyl-2-pentanol); 2: Palmitic acid; 3: Stearic acid; 4: Oleic acid; 5: Linoleic acid; 6: Lignoceric acid.

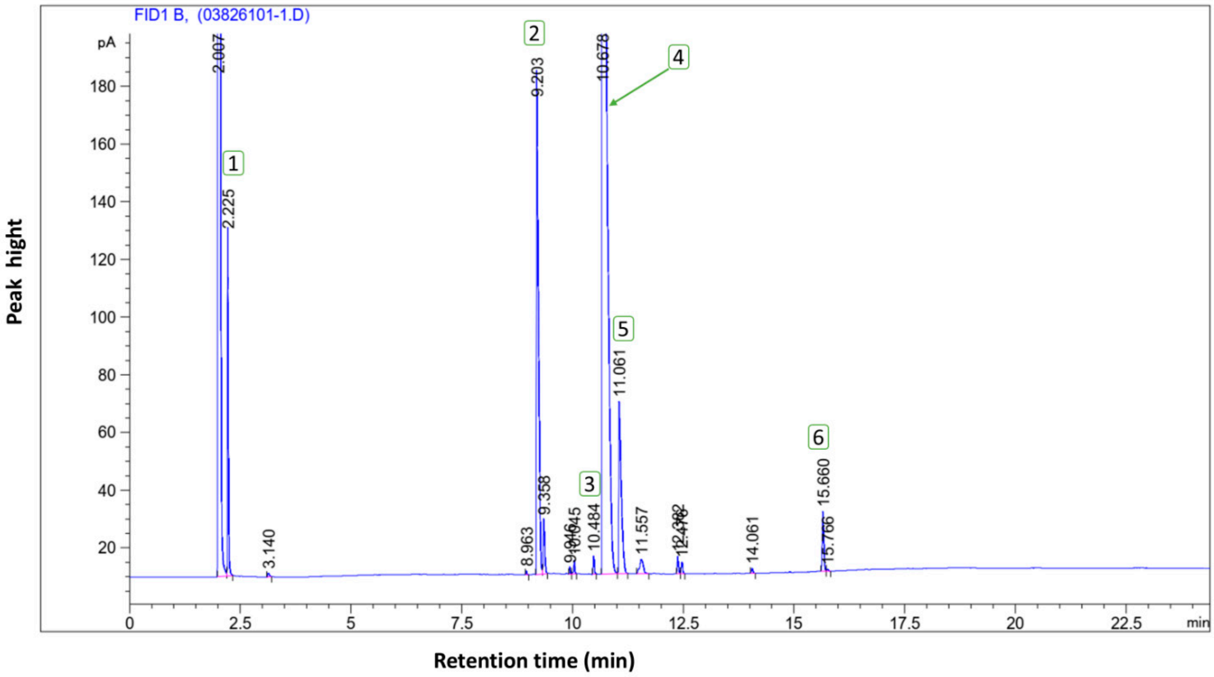

Figure S101. Total ion chromatograms (TICs) from the gas chromatography analysis of the fatty acids in AS_S: Arbosana_Supplemented. (1: Internal standard (4-Methyl-2-pentanol); 2: Palmitic acid; 3: Stearic acid; 4: Oleic acid; 5: Linoleic acid; 6: Lignoceric acid.

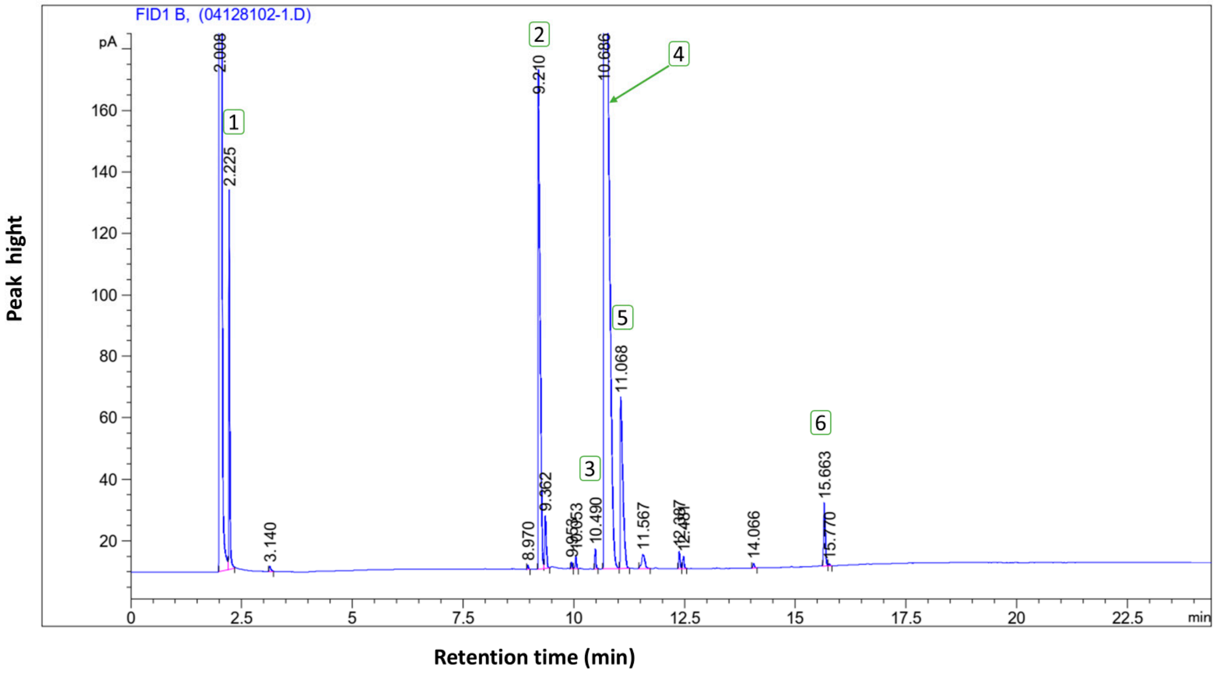

Figure S102. Total ion chromatograms (TICs) from the gas chromatography analysis of the fatty acids in AS_C2: Arbosana_Control 2. (1: Internal standard (4-Methyl-2-pentanol); 2: Palmitic acid; 3: Stearic acid; 4: Oleic acid; 5: Linoleic acid; 6: Lignoceric acid.

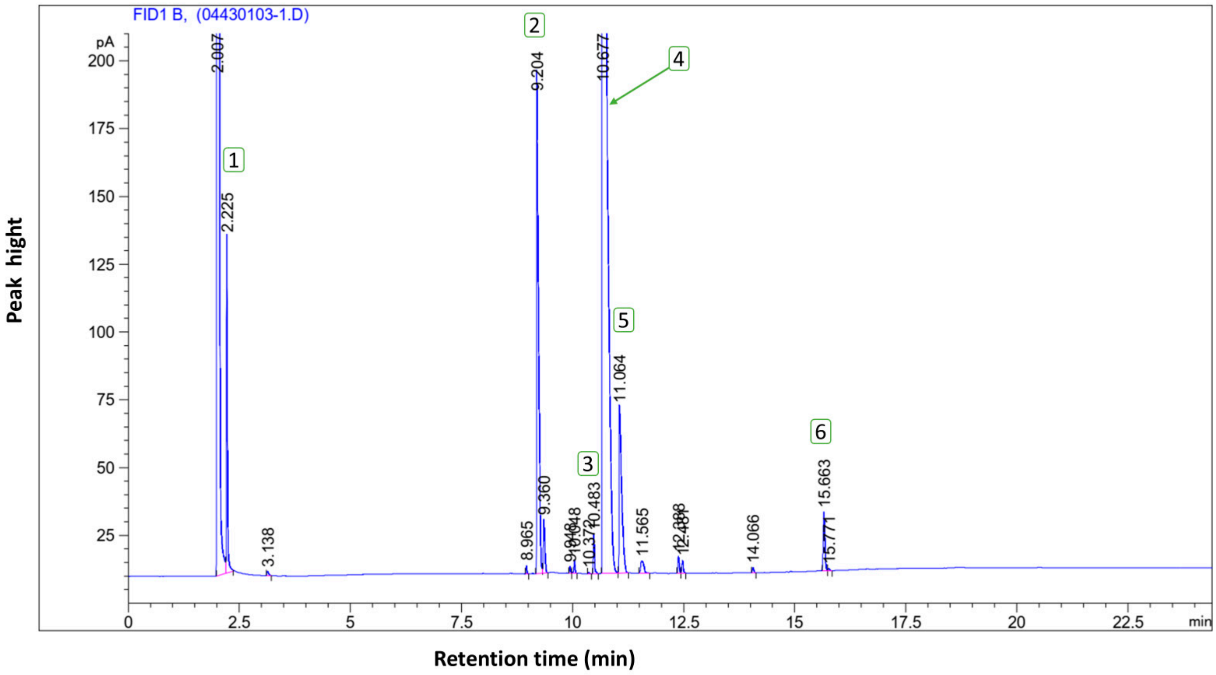

Figure S103. Total ion chromatograms (TICs) from the gas chromatography analysis of the fatty acids in AS_1: Arbosana_Exp 1. (1: Internal standard (4-Methyl-2-pentanol); 2: Palmitic acid; 3: Stearic acid; 4: Oleic acid; 5: Linoleic acid; 6: Lignoceric acid.

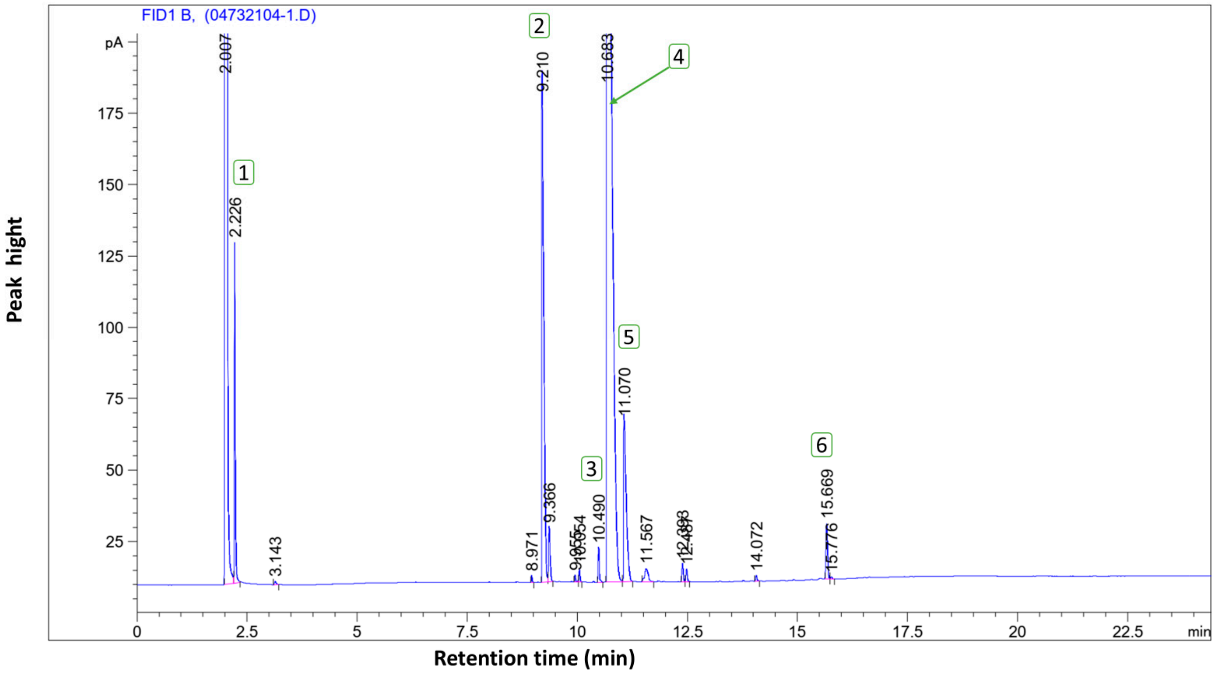

Figure S104. Total ion chromatograms (TICs) from the gas chromatography analysis of the fatty acids in AS_2: Arbosana_Exp 2. (1: Internal standard (4-Methyl-2-pentanol); 2: Palmitic acid; 3: Stearic acid; 4: Oleic acid; 5: Linoleic acid; 6: Lignoceric acid.

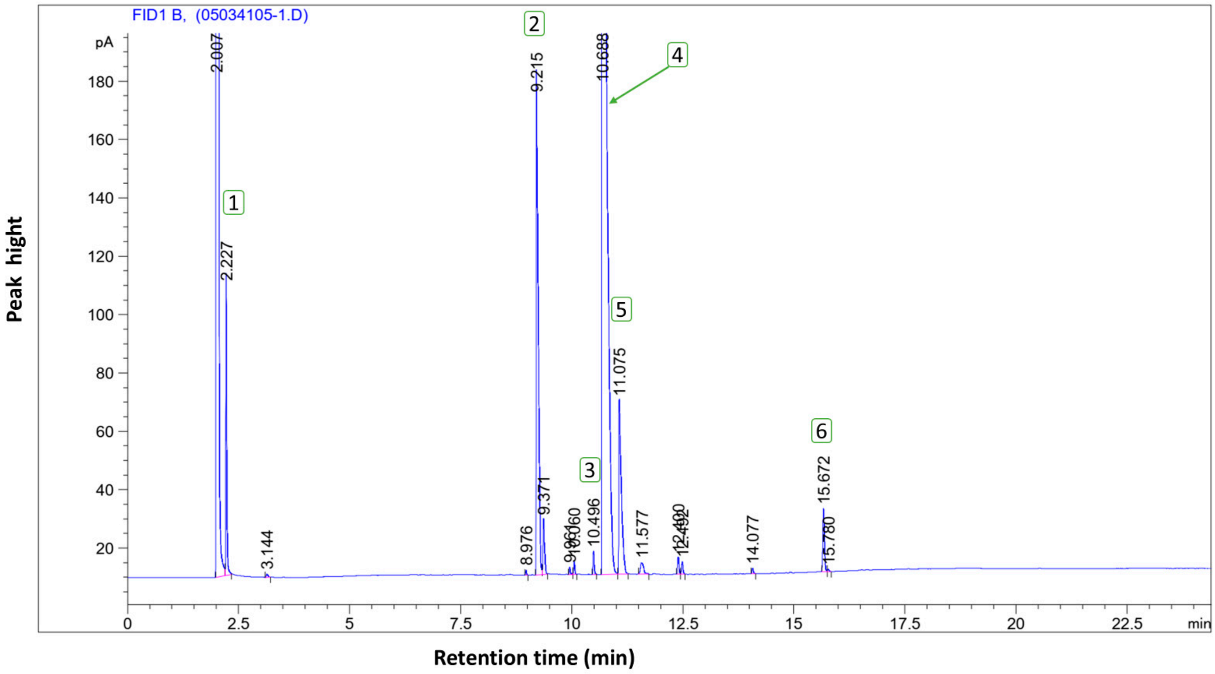

Figure S105. Total ion chromatograms (TICs) from the gas chromatography analysis of the fatty acids in AS_3: Arbosana_Exp 3. (1: Internal standard (4-Methyl-2-pentanol); 2: Palmitic acid; 3: Stearic acid; 4: Oleic acid; 5: Linoleic acid; 6: Lignoceric acid.

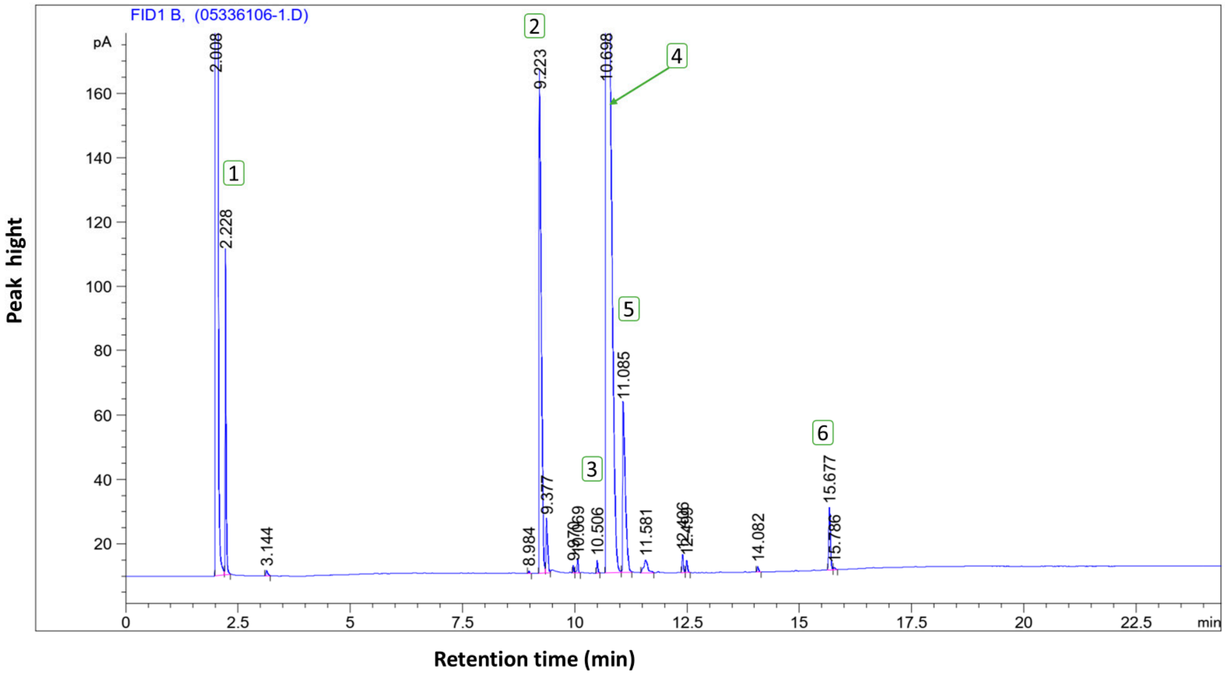

Figure S106. Total ion chromatograms (TICs) from the gas chromatography analysis of the fatty acids in AS_4: Arbosana_Exp 4. (1: Internal standard (4-Methyl-2-pentanol); 2: Palmitic acid; 3: Stearic acid; 4: Oleic acid; 5: Linoleic acid; 6: Lignoceric acid.

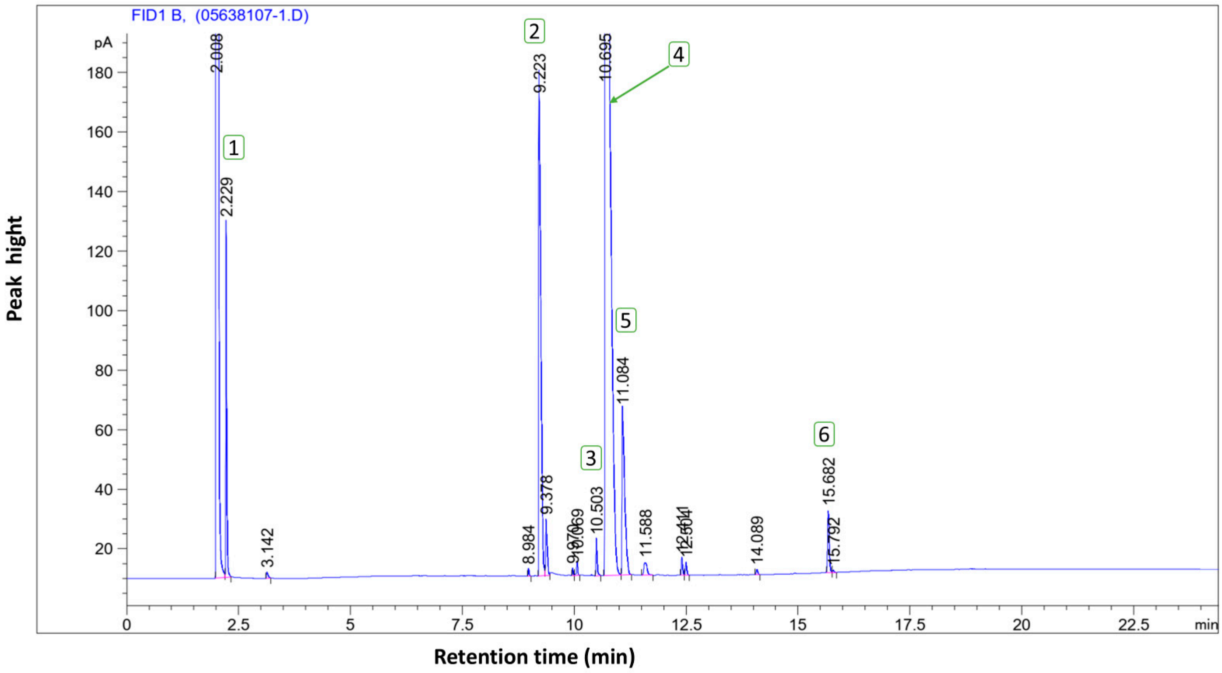

Figure S107. Total ion chromatograms (TICs) from the gas chromatography analysis of the fatty acids in AS_5: Arbosana_Exp 5. (1: Internal standard (4-Methyl-2-pentanol); 2: Palmitic acid; 3: Stearic acid; 4: Oleic acid; 5: Linoleic acid; 6: Lignoceric acid.

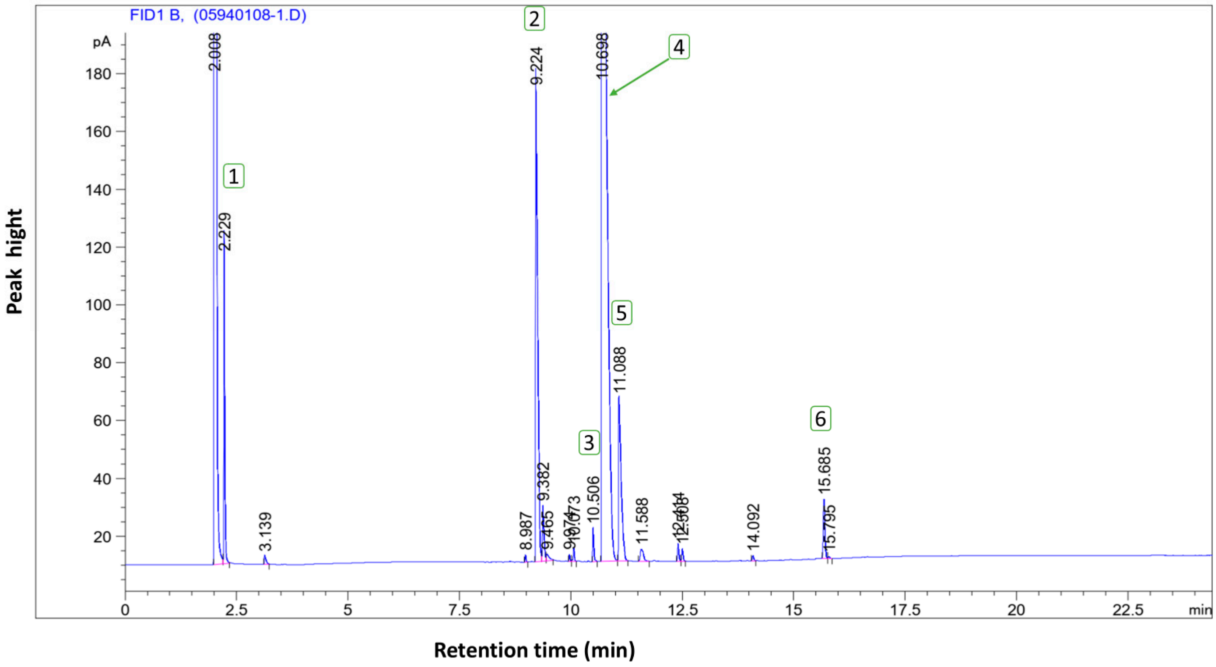

Figure S108. Total ion chromatograms (TICs) from the gas chromatography analysis of the fatty acids in AS_6: Arbosana_Exp 6. (1: Internal standard (4-Methyl-2-pentanol); 2: Palmitic acid; 3: Stearic acid; 4: Oleic acid; 5: Linoleic acid; 6: Lignoceric acid.

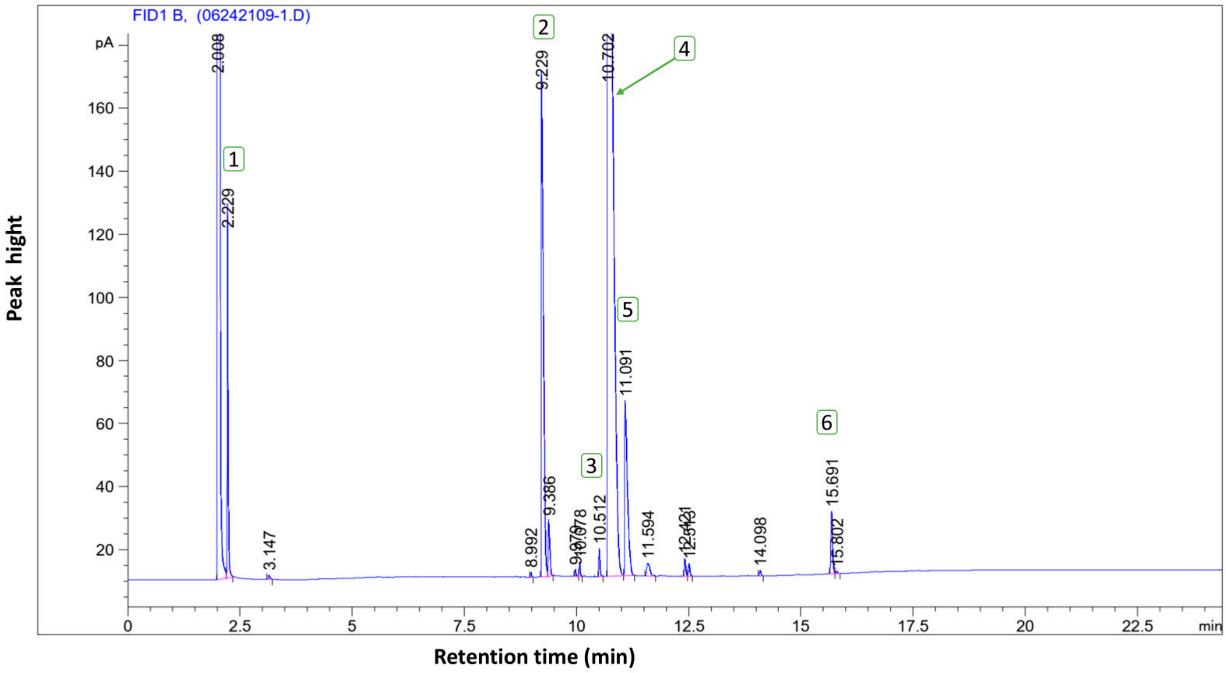

Figure S109. Total ion chromatograms (TICs) from the gas chromatography analysis of the fatty acids in AS_7: Arbosana_Exp 7. (1: Internal standard (4-Methyl-2-pentanol); 2: Palmitic acid; 3: Stearic acid; 4: Oleic acid; 5: Linoleic acid; 6: Lignoceric acid.

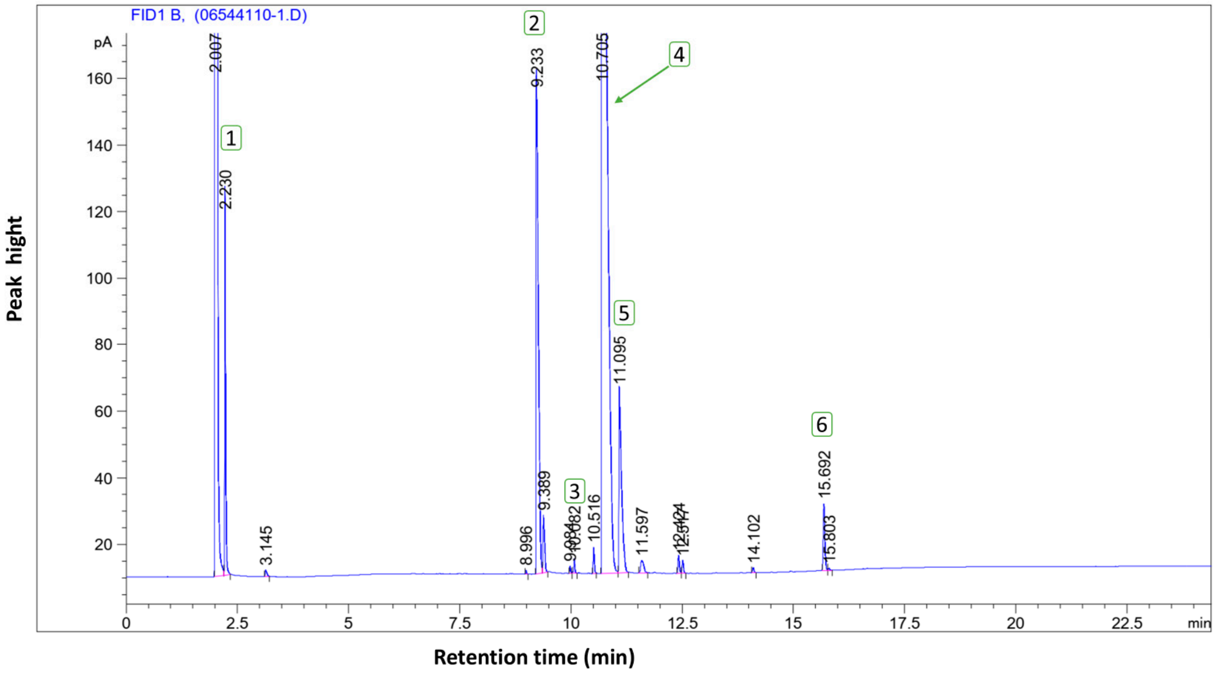

Figure S110. Total ion chromatograms (TICs) from the gas chromatography analysis of the fatty acids in AS_8: Arbosana_Exp 8. (1: Internal standard (4-Methyl-2-pentanol); 2: Palmitic acid; 3: Stearic acid; 4: Oleic acid; 5: Linoleic acid; 6: Lignoceric acid.

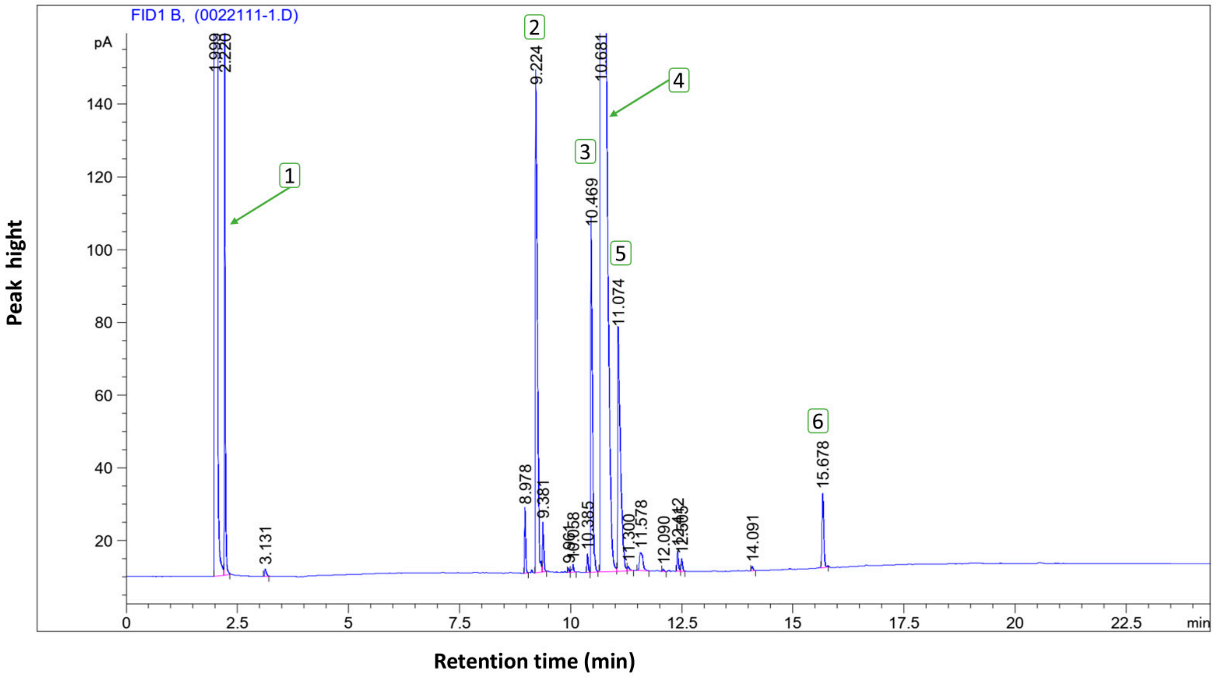

Figure S111. Total ion chromatograms (TICs) from the gas chromatography analysis of the fatty acids in 1ºO_C1: Olive 1º_ Control 1. (1: Internal standard (4-Methyl-2-pentanol); 2: Palmitic acid; 3: Stearic acid; 4: Oleic acid; 5: Linoleic acid; 6: Lignoceric acid.

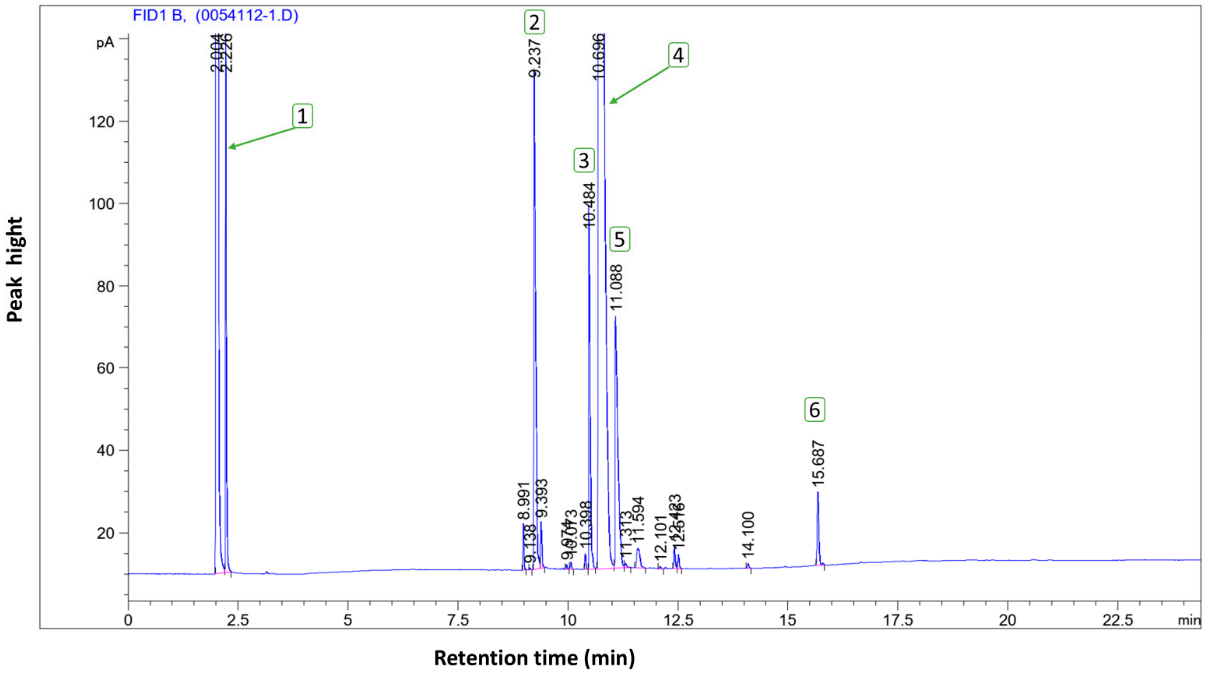

Figure S112. Total ion chromatograms (TICs) from the gas chromatography analysis of the fatty acids in 1ºO_S: Olive 1º_Supplemented. (1: Internal standard (4-Methyl-2-pentanol); 2: Palmitic acid; 3: Stearic acid; 4: Oleic acid; 5: Linoleic acid; 6: Lignoceric acid.

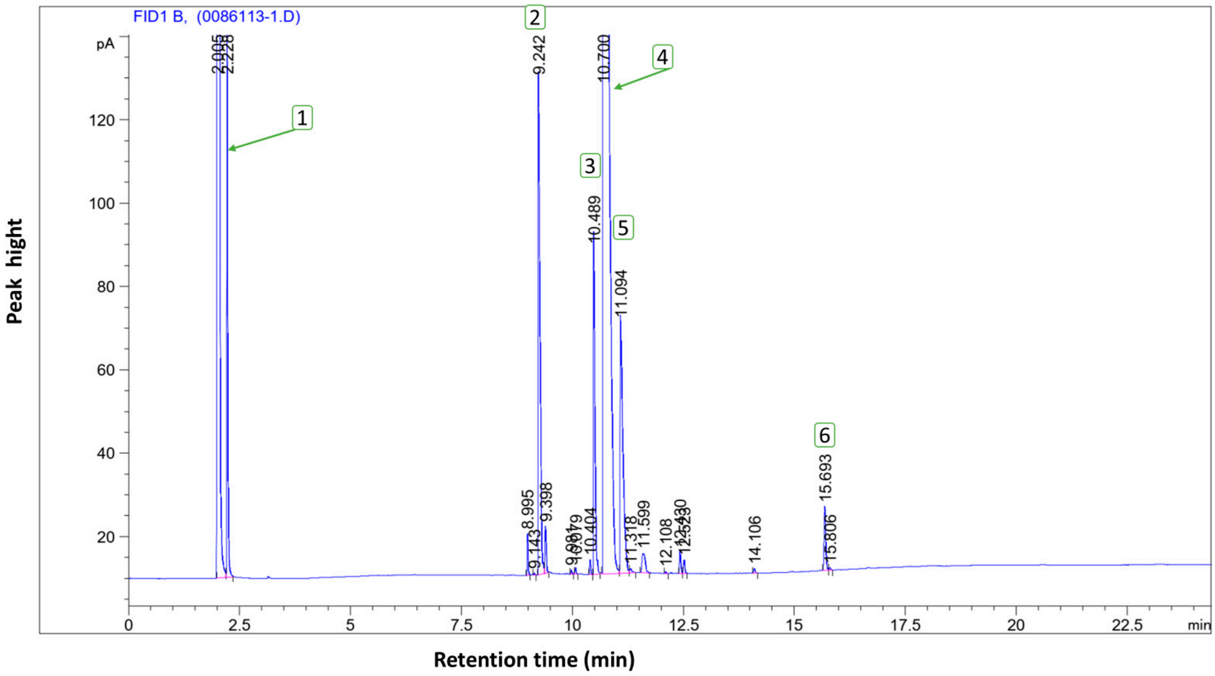

Figure S113. Total ion chromatograms (TICs) from the gas chromatography analysis of the fatty acids in 1ºO_C2: Olive 1º_ Control 2. (1: Internal standard (4-Methyl-2-pentanol); 2: Palmitic acid; 3: Stearic acid; 4: Oleic acid; 5: Linoleic acid; 6: Lignoceric acid.

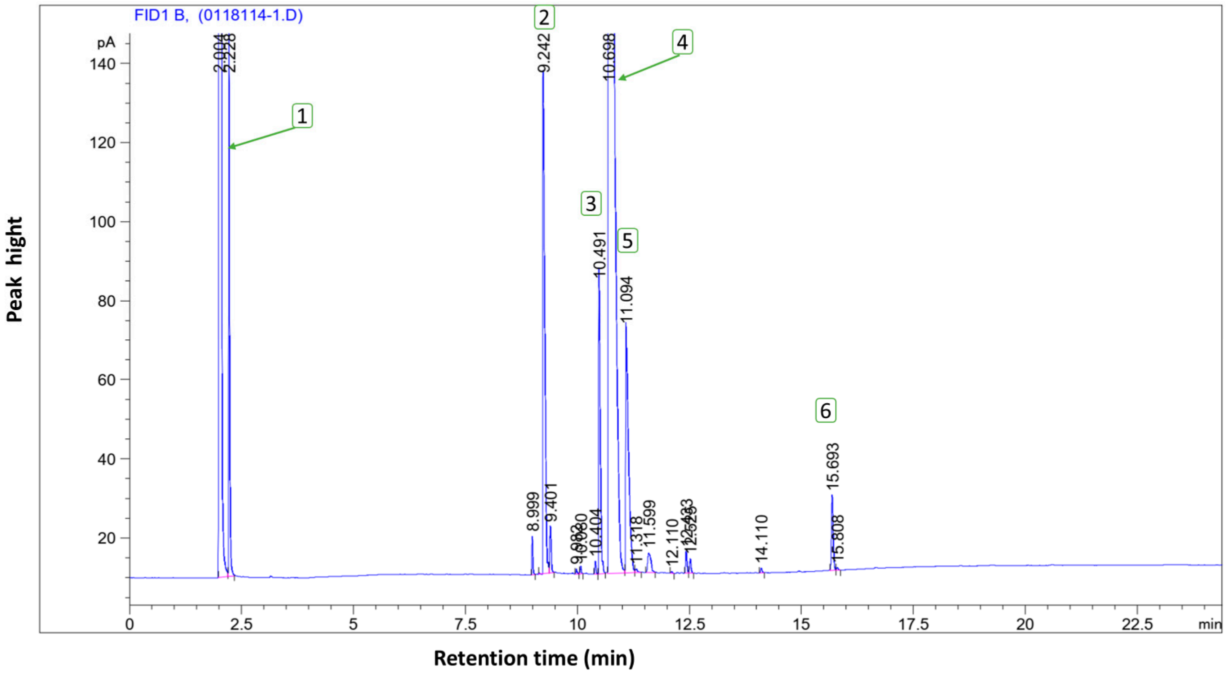

Figure S114. Total ion chromatograms (TICs) from the gas chromatography analysis of the fatty acids in 1ºO_1: Olive 1º_Exp 1. (1: Internal standard (4-Methyl-2-pentanol); 2: Palmitic acid; 3: Stearic acid; 4: Oleic acid; 5: Linoleic acid; 6: Lignoceric acid.

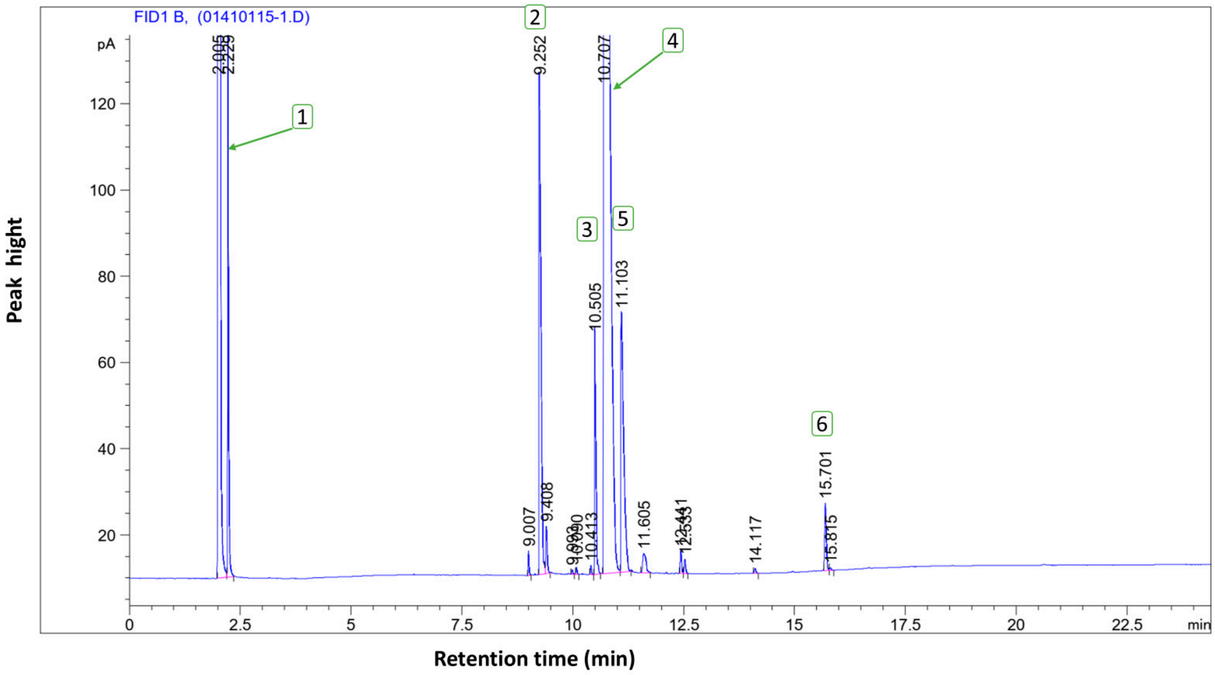

Figure S115. Total ion chromatograms (TICs) from the gas chromatography analysis of the fatty acids in 1ºO_2: Olive 1º_Exp 2. (1: Internal standard (4-Methyl-2-pentanol); 2: Palmitic acid; 3: Stearic acid; 4: Oleic acid; 5: Linoleic acid; 6: Lignoceric acid.

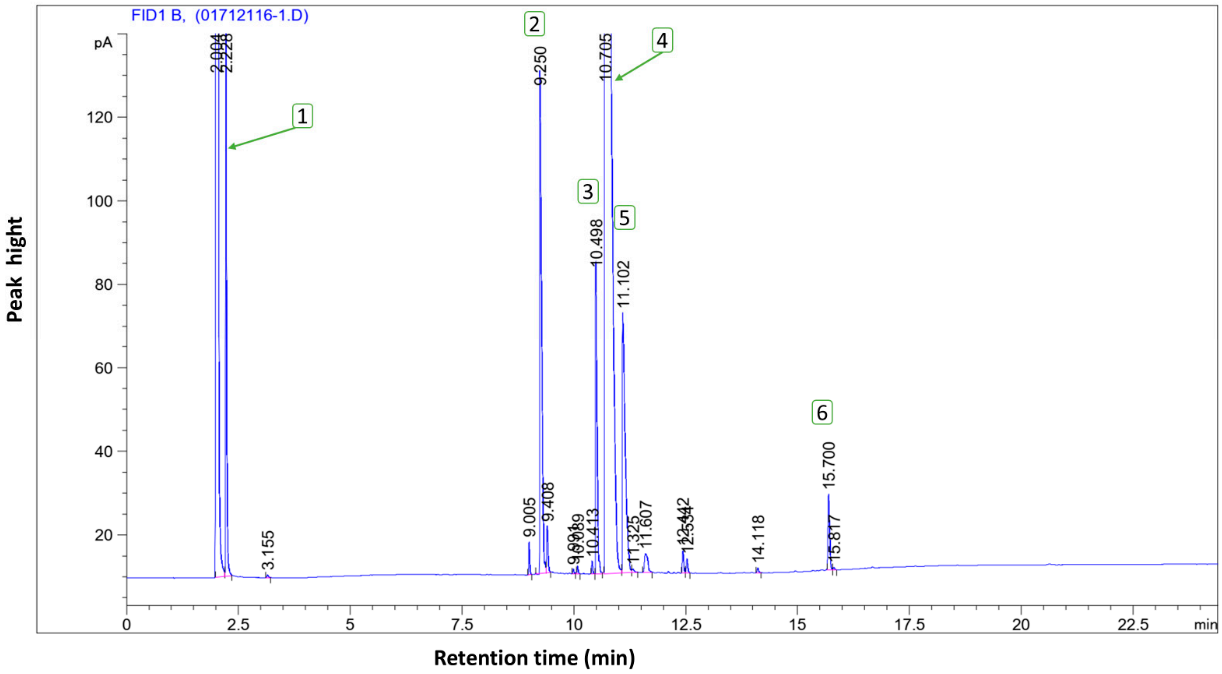

Figure S116. Total ion chromatograms (TICs) from the gas chromatography analysis of the fatty acids in 1ºO_3: Olive 1º_Exp 3. (1: Internal standard (4-Methyl-2-pentanol); 2: Palmitic acid; 3: Stearic acid; 4: Oleic acid; 5: Linoleic acid; 6: Lignoceric acid.

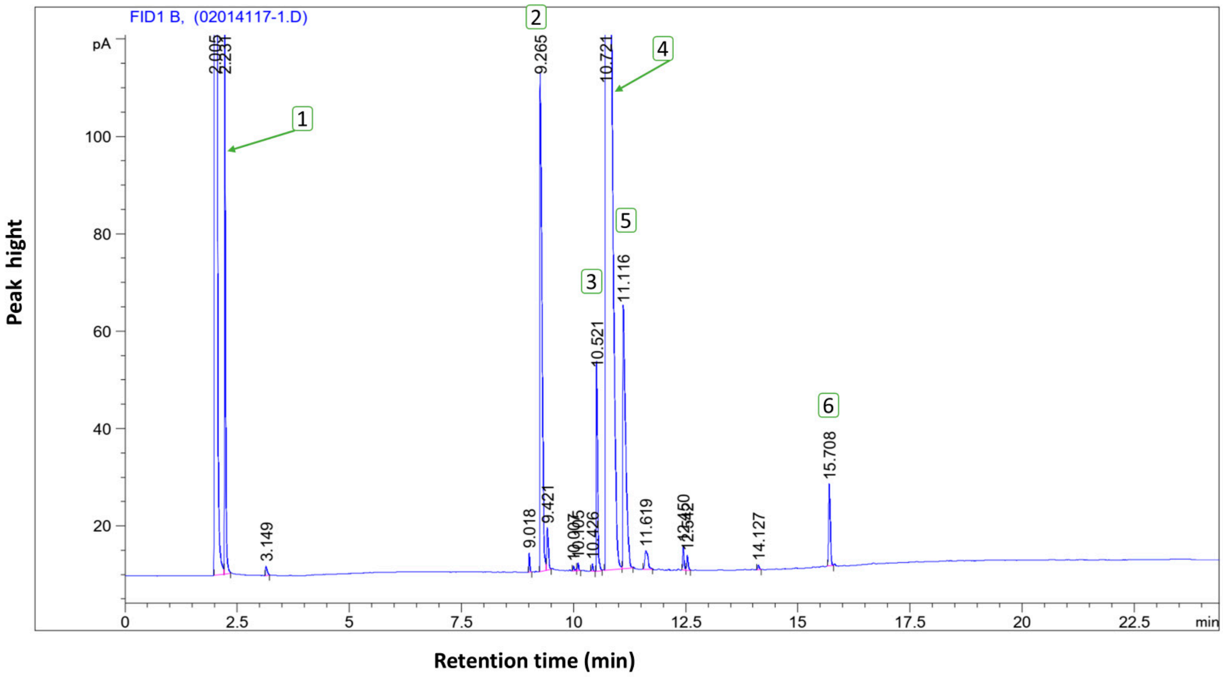

Figure S117. Total ion chromatograms (TICs) from the gas chromatography analysis of the fatty acids in 1ºO_4: Olive 1º_Exp 4. (1: Internal standard (4-Methyl-2-pentanol); 2: Palmitic acid; 3: Stearic acid; 4: Oleic acid; 5: Linoleic acid; 6: Lignoceric acid.

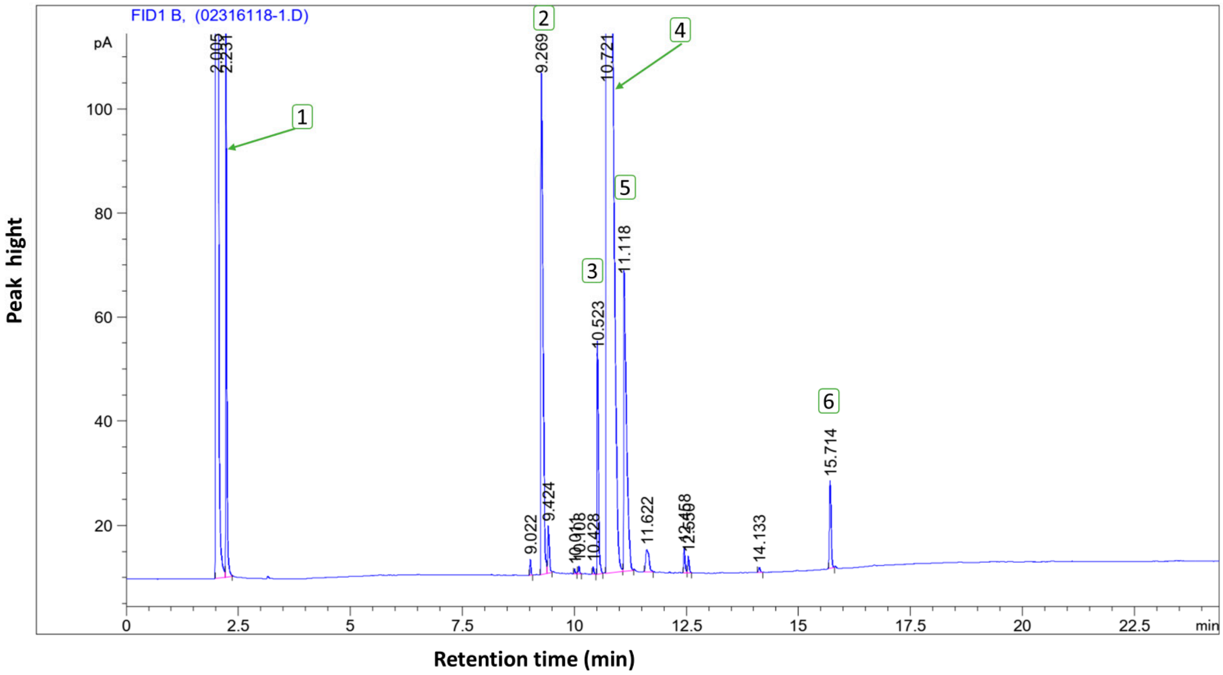

Figure S118. Total ion chromatograms (TICs) from the gas chromatography analysis of the fatty acids in 1ºO_5: Olive 1º_Exp 5. (1: Internal standard (4-Methyl-2-pentanol); 2: Palmitic acid; 3: Stearic acid; 4: Oleic acid; 5: Linoleic acid; 6: Lignoceric acid.

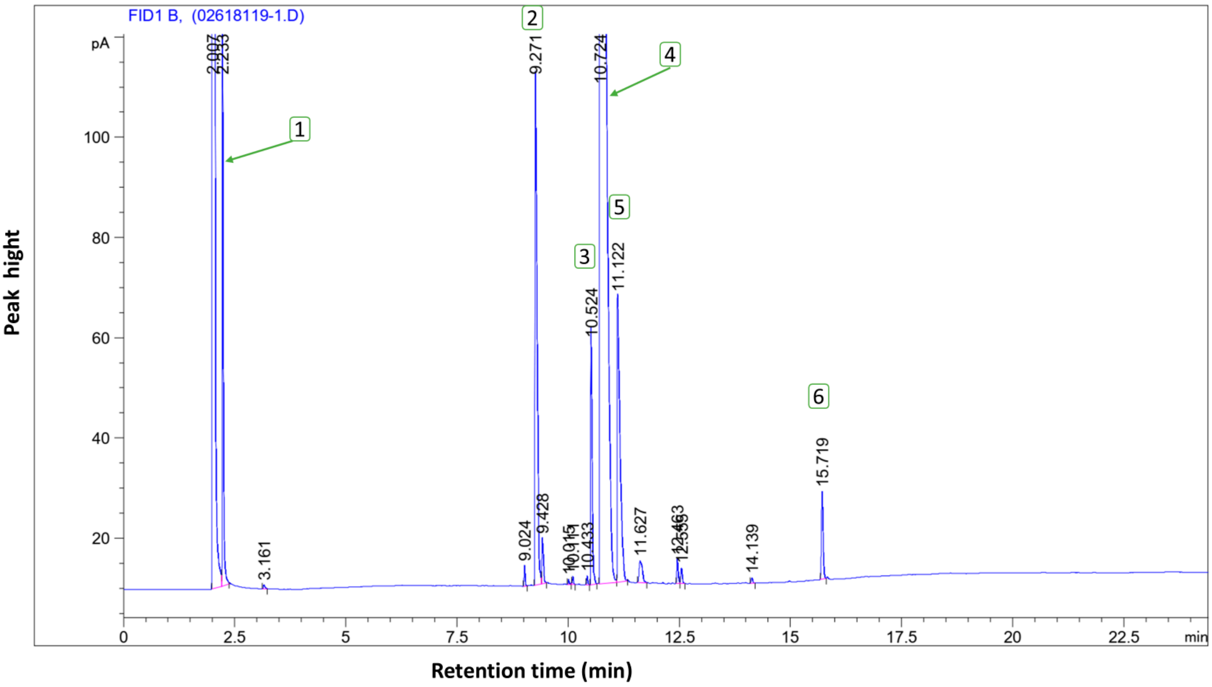

Figure S119. Total ion chromatograms (TICs) from the gas chromatography analysis of the fatty acids in 1ºO_6: Olive 1º_Exp 6. (1: Internal standard (4-Methyl-2-pentanol); 2: Palmitic acid; 3: Stearic acid; 4: Oleic acid; 5: Linoleic acid; 6: Lignoceric acid.

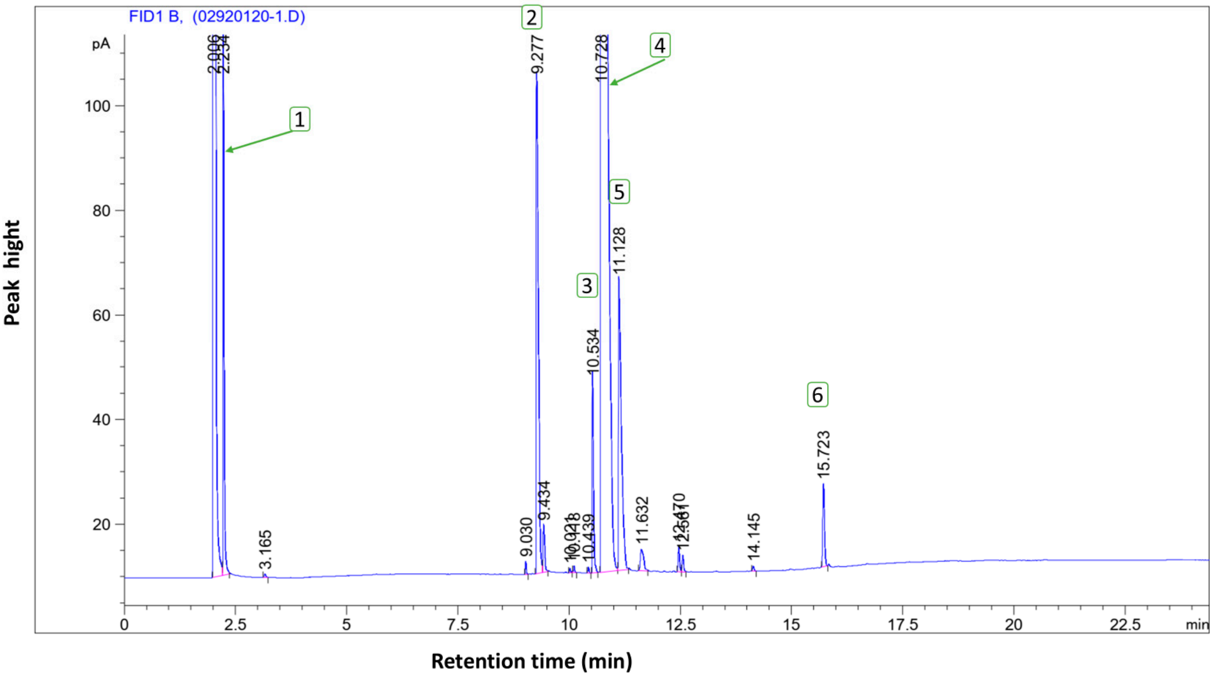

Figure S120. Total ion chromatograms (TICs) from the gas chromatography analysis of the fatty acids in 1ºO_7: Olive 1º_Exp 7. (1: Internal standard (4-Methyl-2-pentanol); 2: Palmitic acid; 3: Stearic acid; 4: Oleic acid; 5: Linoleic acid; 6: Lignoceric acid.

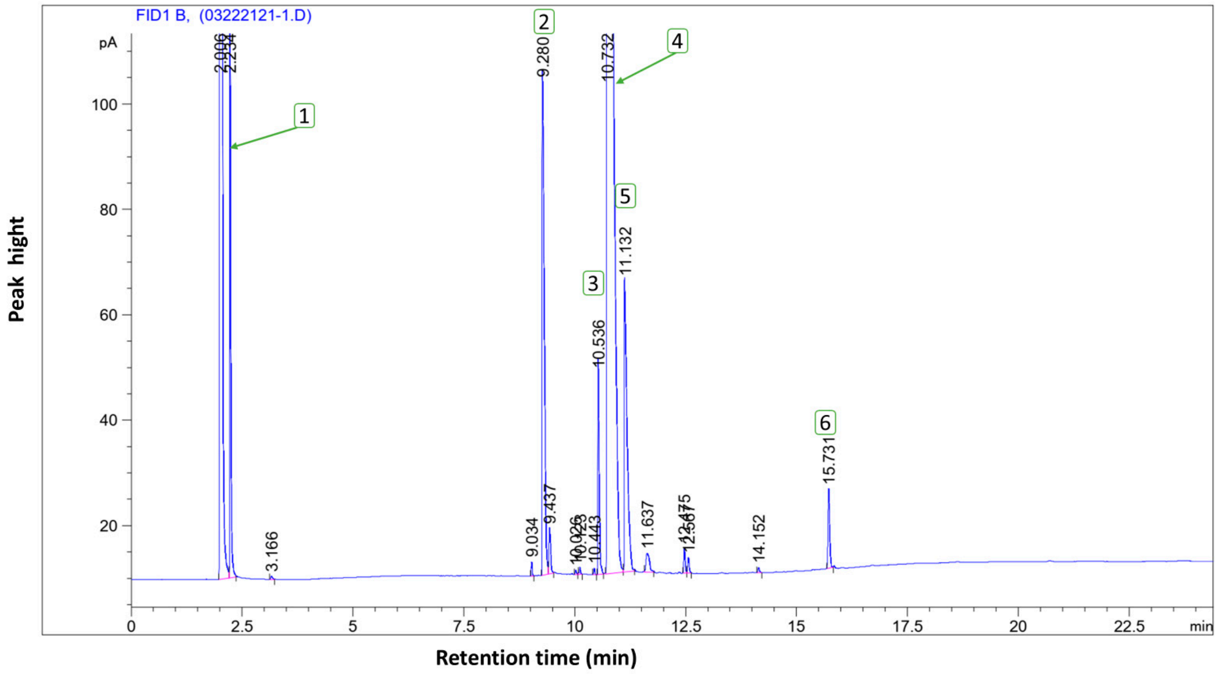

Figure S121. Total ion chromatograms (TICs) from the gas chromatography analysis of the fatty acids in 1ºO_8: Olive 1º_Exp 8. (1: Internal standard (4-Methyl-2-pentanol); 2: Palmitic acid; 3: Stearic acid; 4: Oleic acid; 5: Linoleic acid; 6: Lignoceric acid.

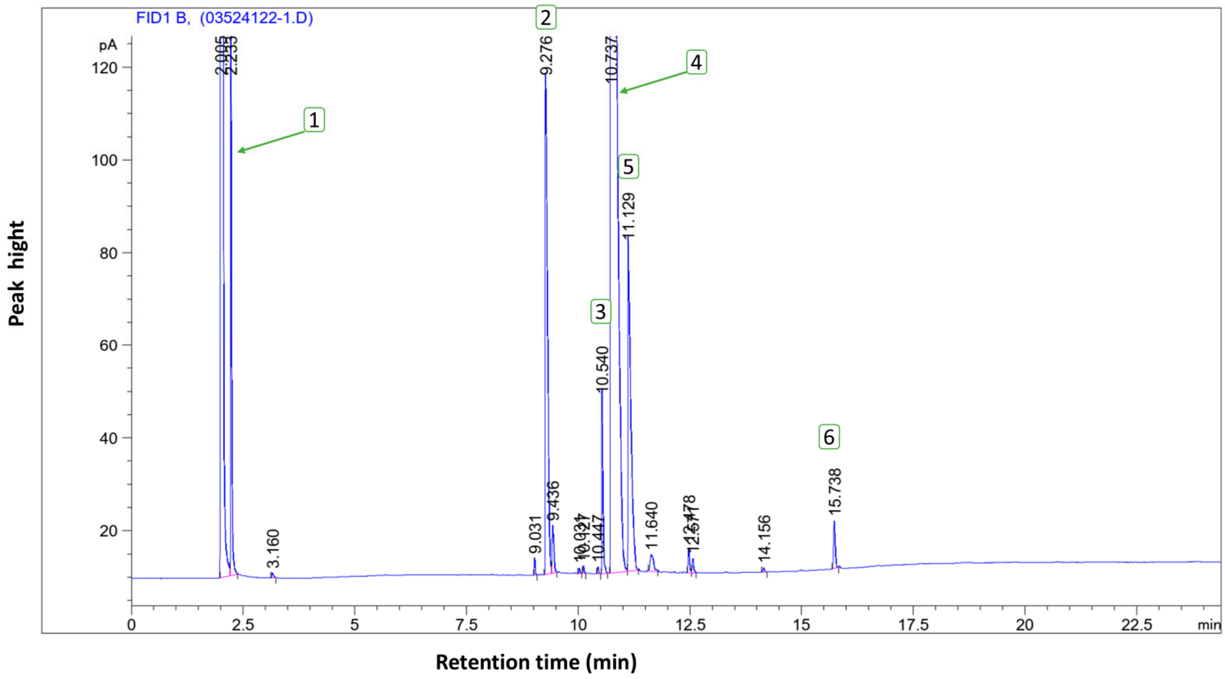

Figure S122. Total ion chromatograms (TICs) from the gas chromatography analysis of the fatty acids in 0.4ºO_C1: Olive 0.4º_ Control 1. (1: Internal standard (4-Methyl-2-pentanol); 2: Palmitic acid; 3: Stearic acid; 4: Oleic acid; 5: Linoleic acid; 6: Lignoceric acid.

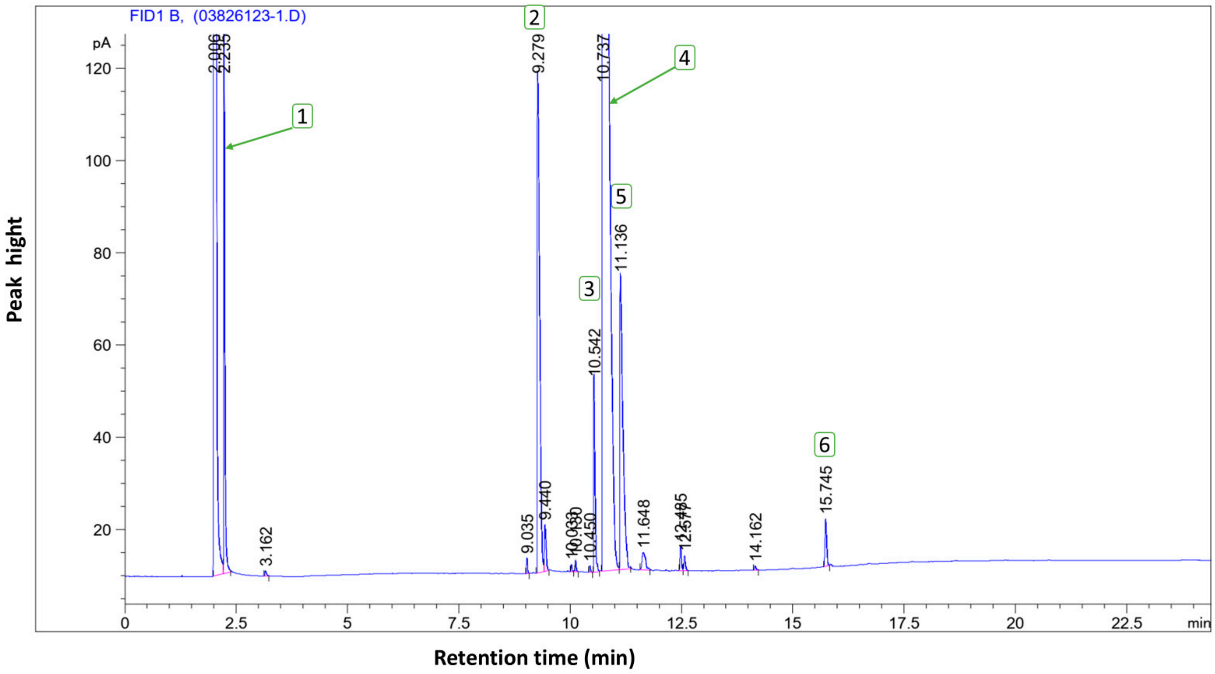

Figure S123. Total ion chromatograms (TICs) from the gas chromatography analysis of the fatty acids in 0.4ºO_S: Olive 0.4º_Supplemented. (1: Internal standard (4-Methyl-2-pentanol); 2: Palmitic acid; 3: Stearic acid; 4: Oleic acid; 5: Linoleic acid; 6: Lignoceric acid.

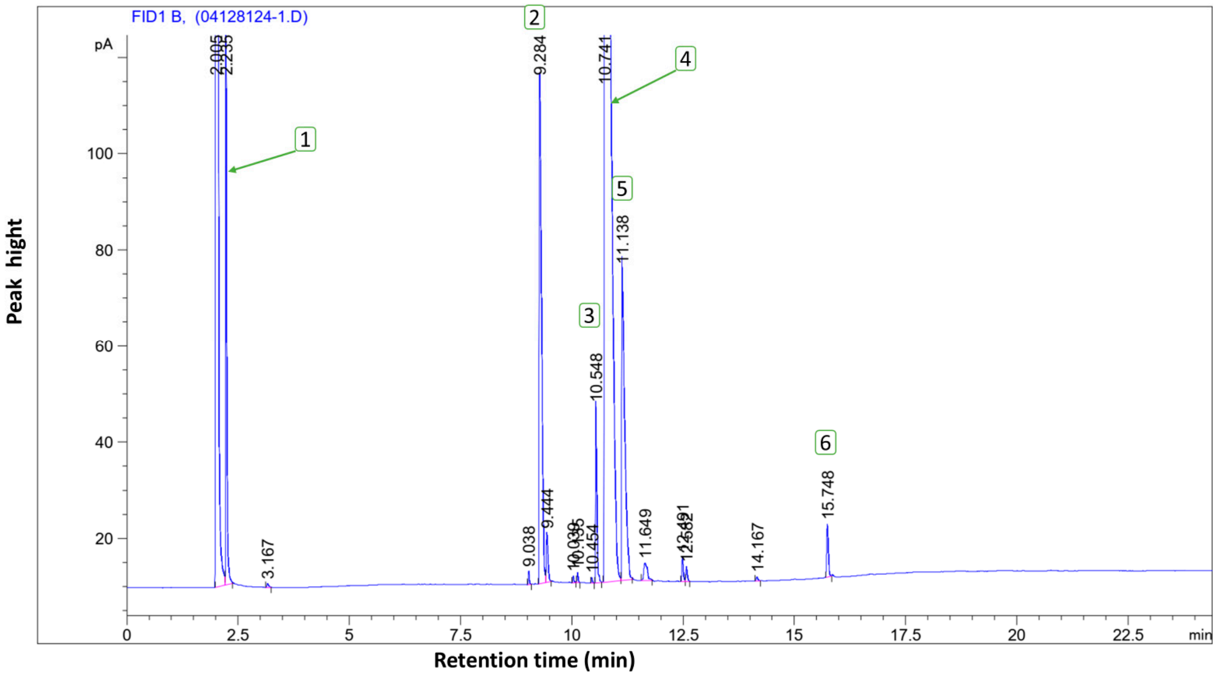

Figure S124. Total ion chromatograms (TICs) from the gas chromatography analysis of the fatty acids in 0.4ºO_C2: Olive 0.4º_ Control 2. (1: Internal standard (4-Methyl-2-pentanol); 2: Palmitic acid; 3: Stearic acid; 4: Oleic acid; 5: Linoleic acid; 6: Lignoceric acid.

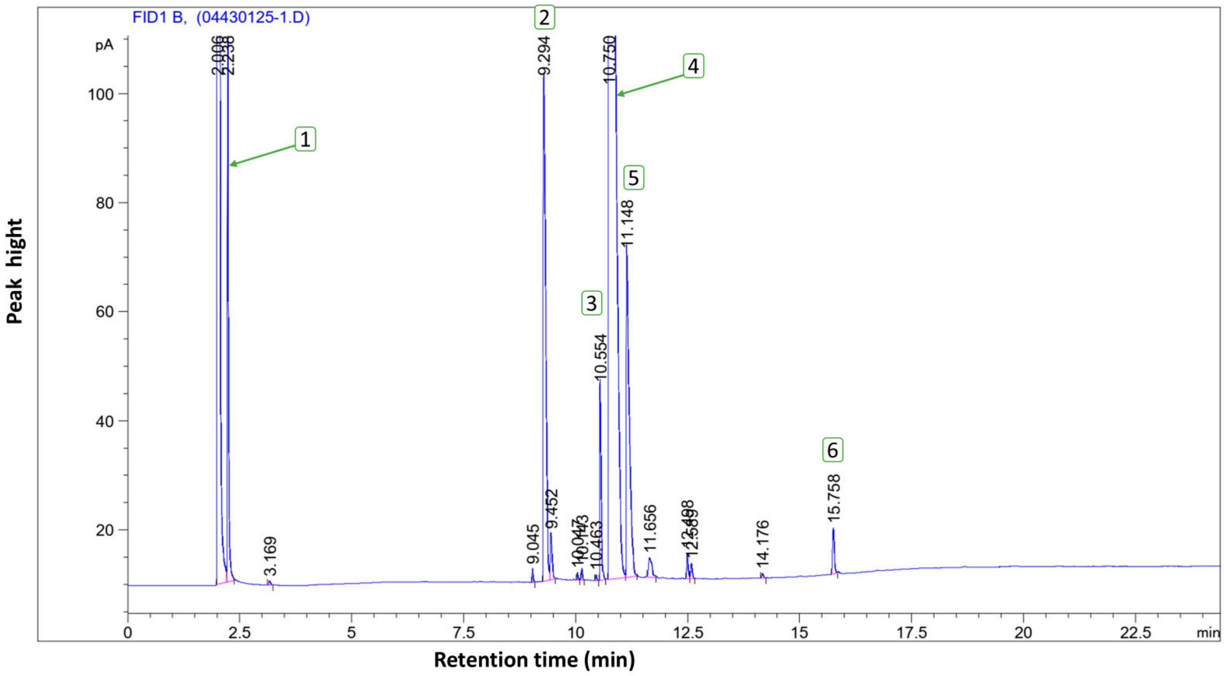

Figure S125. Total ion chromatograms (TICs) from the gas chromatography analysis of the fatty acids in 0.4ºO_1: Olive 0.4º_Exp 1. (1: Internal standard (4-Methyl-2-pentanol); 2: Palmitic acid; 3: Stearic acid; 4: Oleic acid; 5: Linoleic acid; 6: Lignoceric acid.

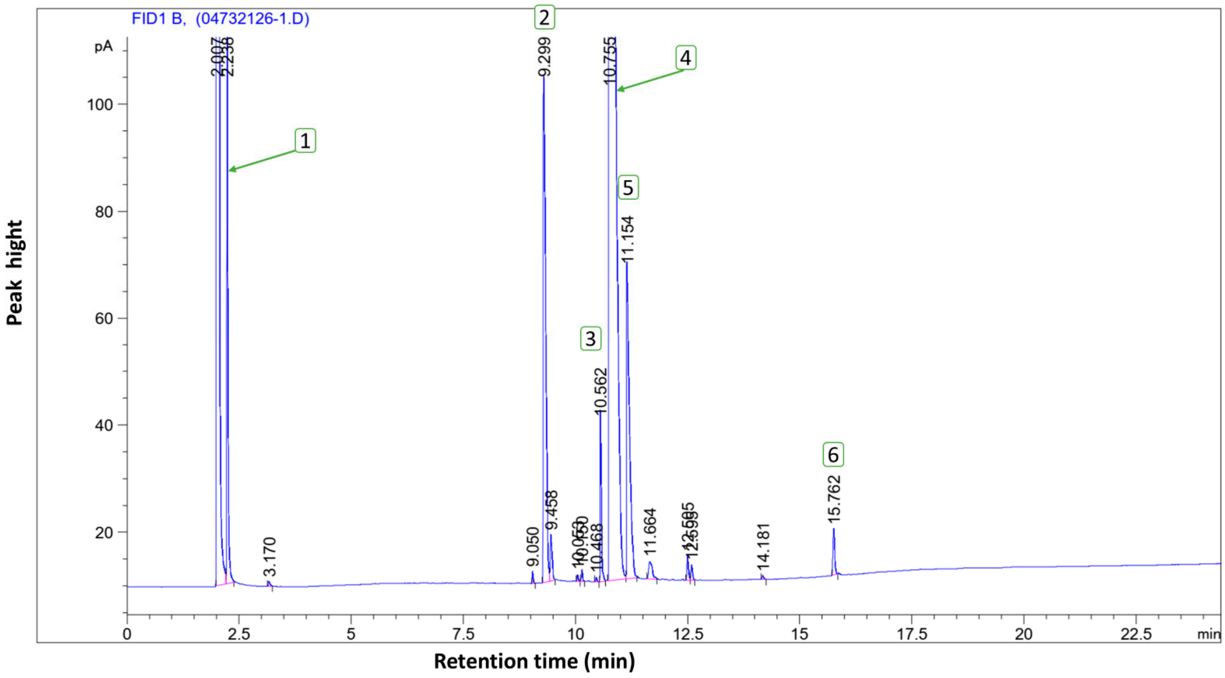

Figure S126. Total ion chromatograms (TICs) from the gas chromatography analysis of the fatty acids in 0.4ºO_2: Olive 0.4º_Exp 2. (1: Internal standard (4-Methyl-2-pentanol); 2: Palmitic acid; 3: Stearic acid; 4: Oleic acid; 5: Linoleic acid; 6: Lignoceric acid.

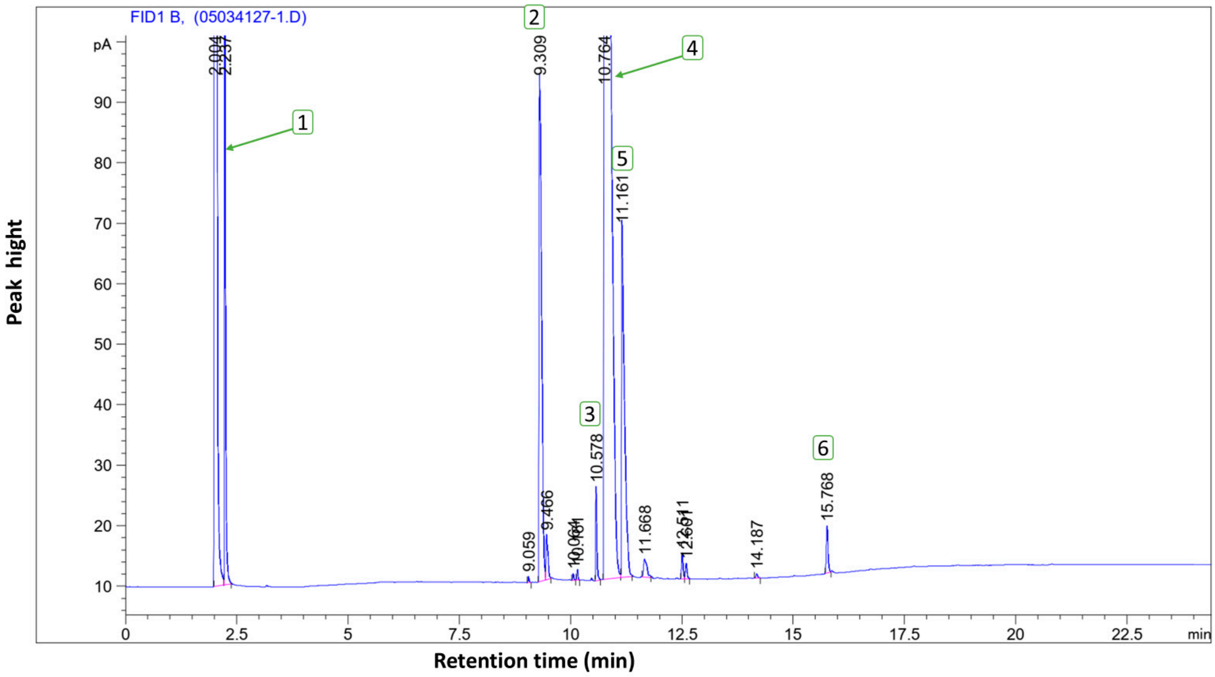

Figure S127. Total ion chromatograms (TICs) from the gas chromatography analysis of the fatty acids in 0.4ºO_3: Olive 0.4º_Exp 3. (1: Internal standard (4-Methyl-2-pentanol); 2: Palmitic acid; 3: Stearic acid; 4: Oleic acid; 5: Linoleic acid; 6: Lignoceric acid.

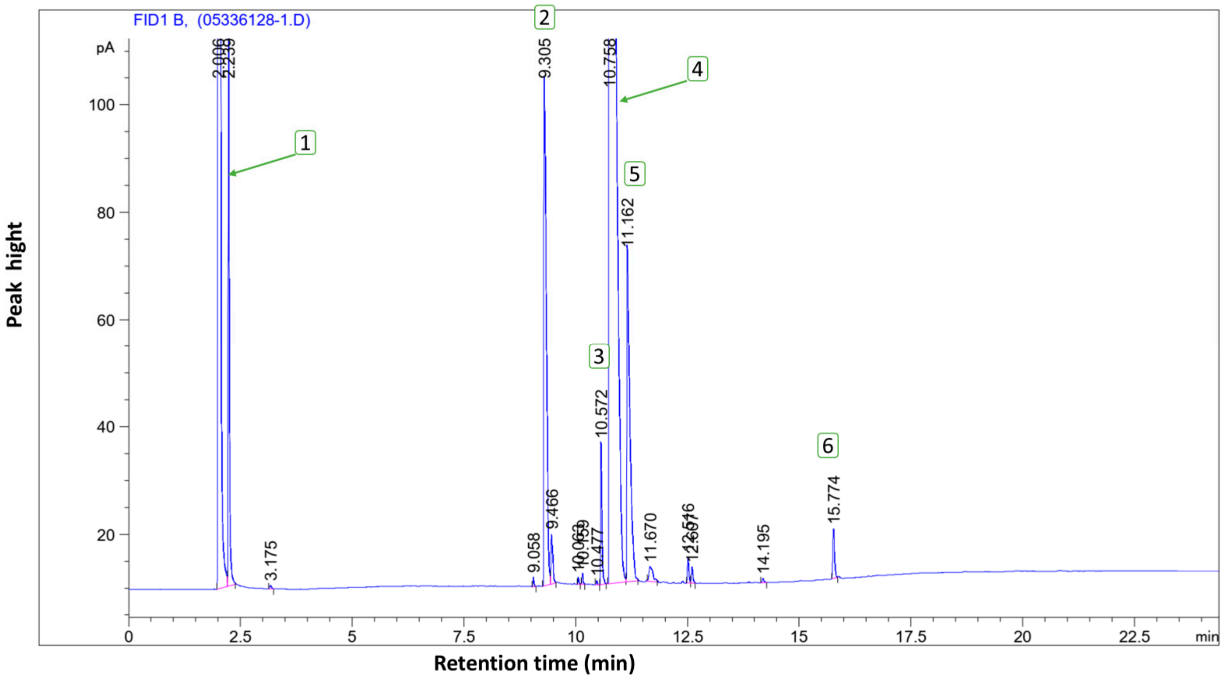

Figure S128. Total ion chromatograms (TICs) from the gas chromatography analysis of the fatty acids in 0.4ºO_4: Olive 0.4º_Exp 4. (1: Internal standard (4-Methyl-2-pentanol); 2: Palmitic acid; 3: Stearic acid; 4: Oleic acid; 5: Linoleic acid; 6: Lignoceric acid.

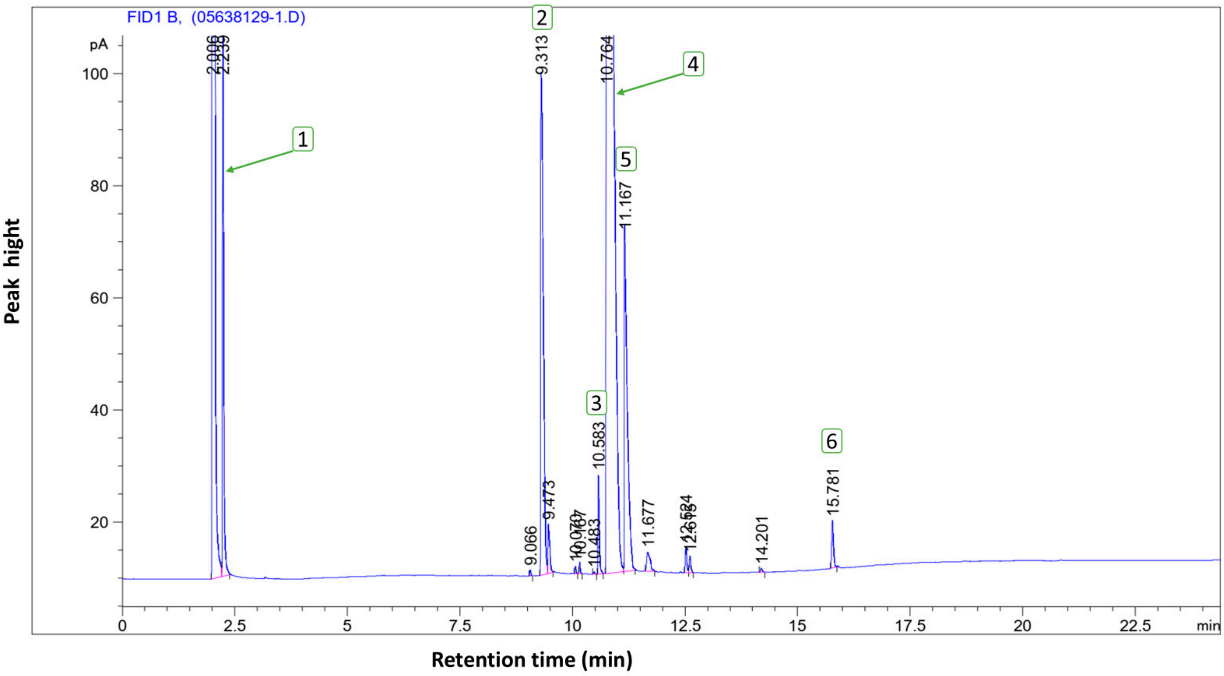

Figure S129. Total ion chromatograms (TICs) from the gas chromatography analysis of the fatty acids in 0.4ºO_5: Olive 0.4º_Exp 5. (1: Internal standard (4-Methyl-2-pentanol); 2: Palmitic acid; 3: Stearic acid; 4: Oleic acid; 5: Linoleic acid; 6: Lignoceric acid.

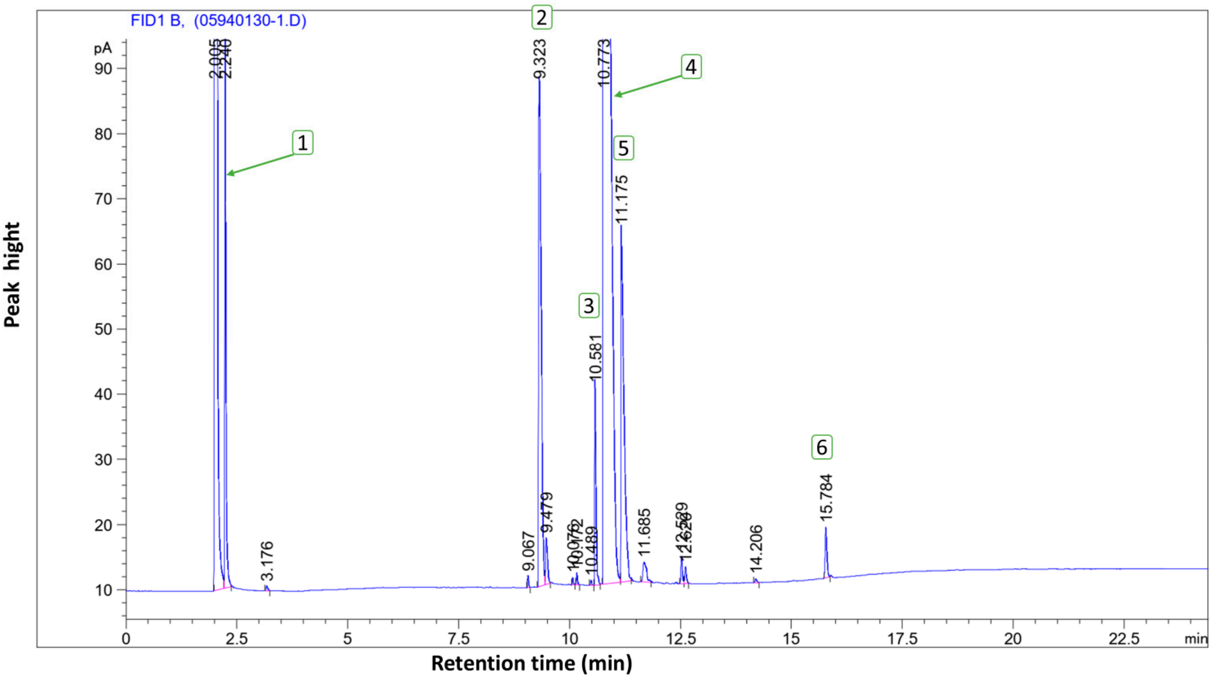

Figure S130. Total ion chromatograms (TICs) from the gas chromatography analysis of the fatty acids in 0.4ºO_6: Olive 0.4º_Exp 6. (1: Internal standard (4-Methyl-2-pentanol); 2: Palmitic acid; 3: Stearic acid; 4: Oleic acid; 5: Linoleic acid; 6: Lignoceric acid.

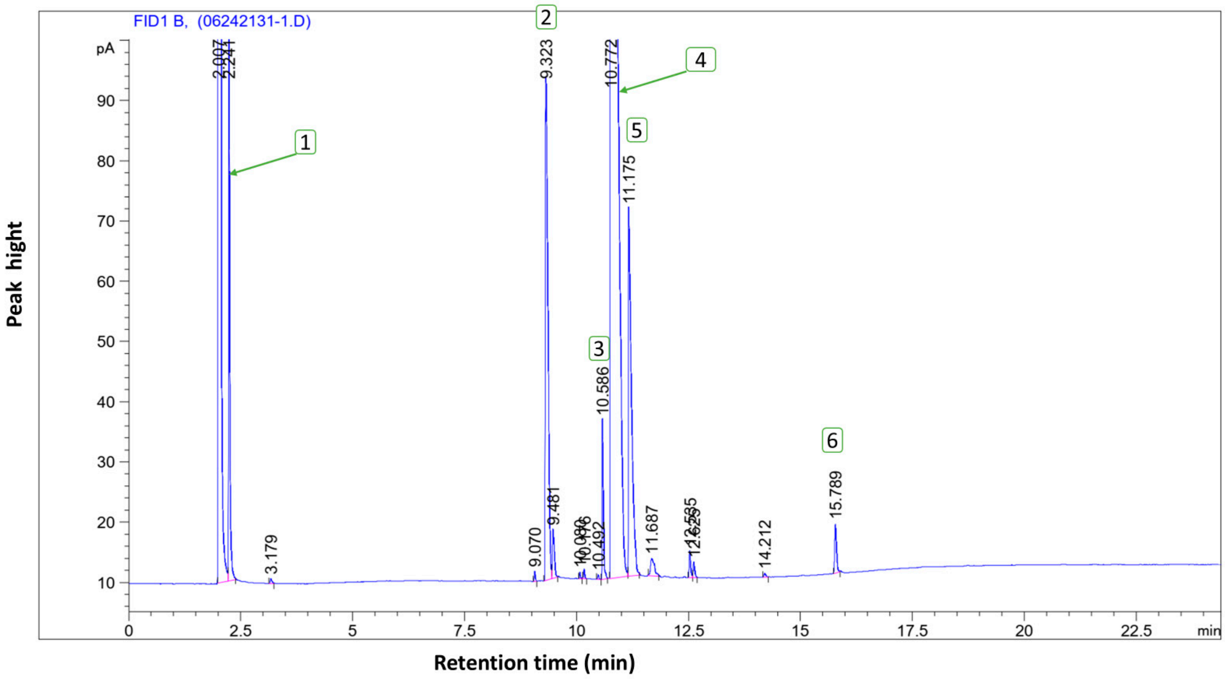

Figure S131. Total ion chromatograms (TICs) from the gas chromatography analysis of the fatty acids in 0.4ºO_7: Olive 0.4º_Exp 7. (1: Internal standard (4-Methyl-2-pentanol); 2: Palmitic acid; 3: Stearic acid; 4: Oleic acid; 5: Linoleic acid; 6: Lignoceric acid.

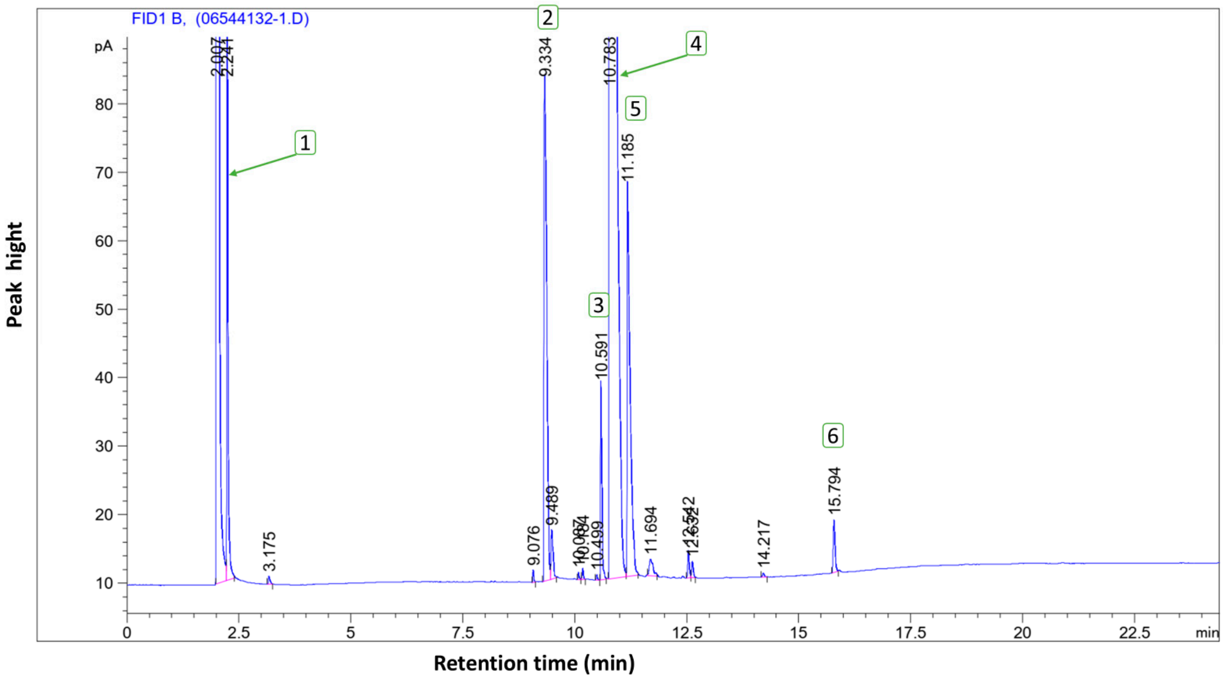

Figure S132. Total ion chromatograms (TICs) from the gas chromatography analysis of the fatty acids in 0.4.ºO_8: Olive 0.4º_Exp 8. (1: Internal standard (4-Methyl-2-pentanol); 2: Palmitic acid; 3: Stearic acid; 4: Oleic acid; 5: Linoleic acid; 6: Lignoceric acid.

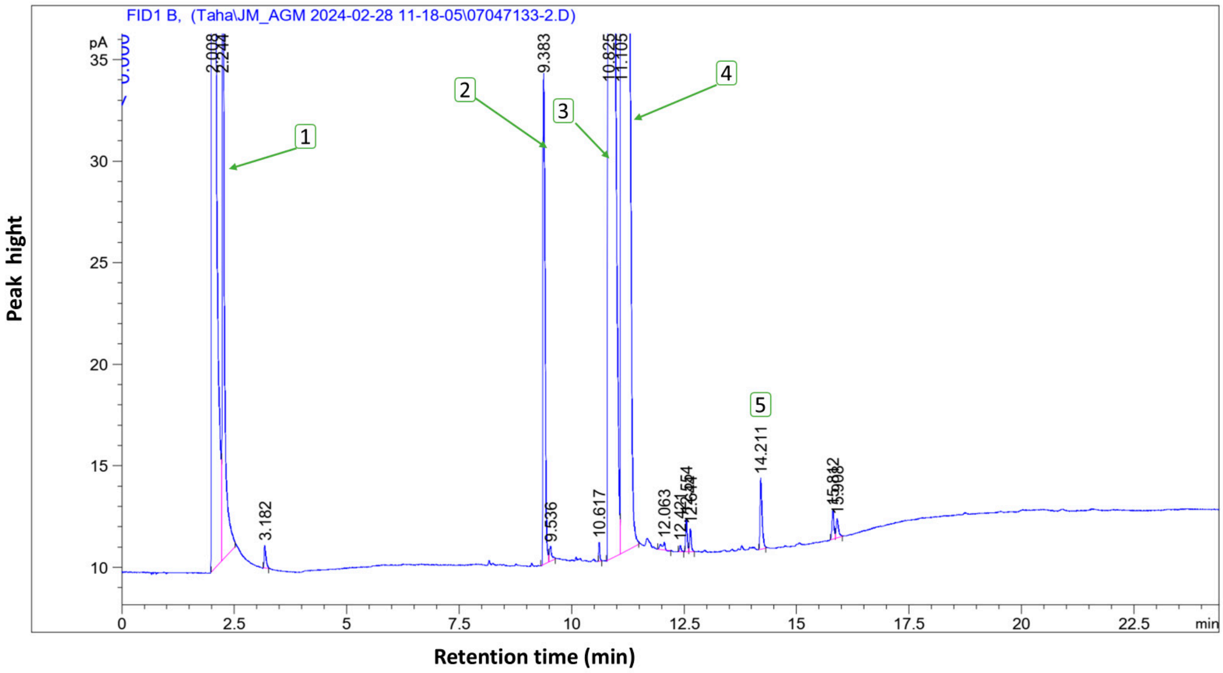

Figure S133. Total ion chromatograms (TICs) from the gas chromatography analysis of the fatty acids in SO_C: Sunflower oil_Contol. (1: Internal standard (4-Methyl-2-pentanol); 2: Palmitic acid; 3: Oleic acid; 4: Linoleic acid; 5: Gadoleic acid.

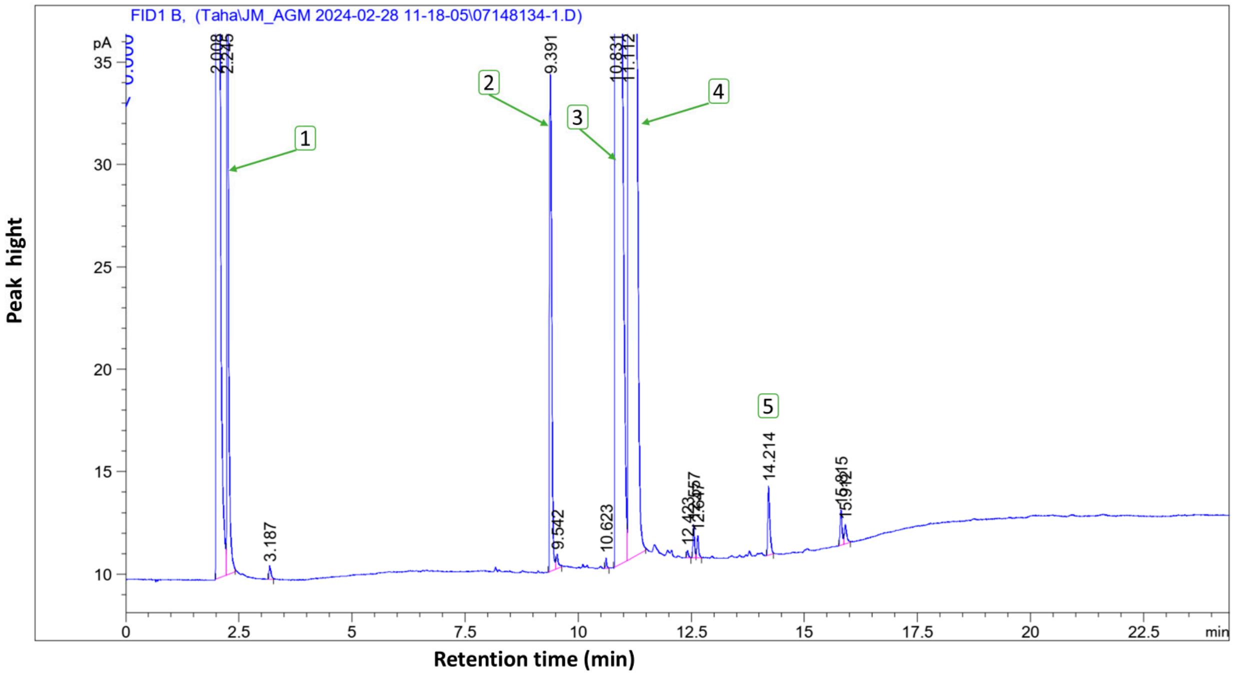

Figure S134. Total ion chromatograms (TICs) from the gas chromatography analysis of the fatty acids in SO_1: Sunflower oil_Exp 1. (1: Internal standard (4-Methyl-2-pentanol); 2: Palmitic acid; 3: Oleic acid; 4: Linoleic acid; 5: Gadoleic acid.

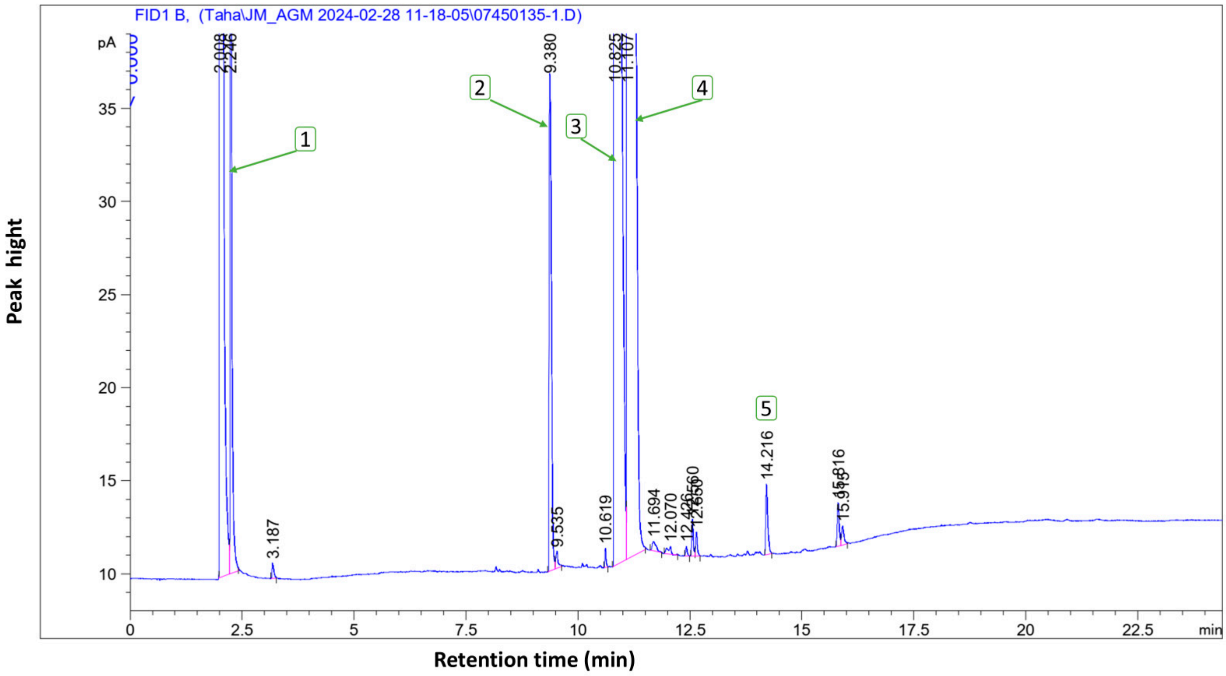

Figure S135. Total ion chromatograms (TICs) from the gas chromatography analysis of the fatty acids in SO_2: Sunflower oil_Exp 2. (1: Internal standard (4-Methyl-2-pentanol); 2: Palmitic acid; 3: Oleic acid; 4: Linoleic acid; 5: Gadoleic acid.

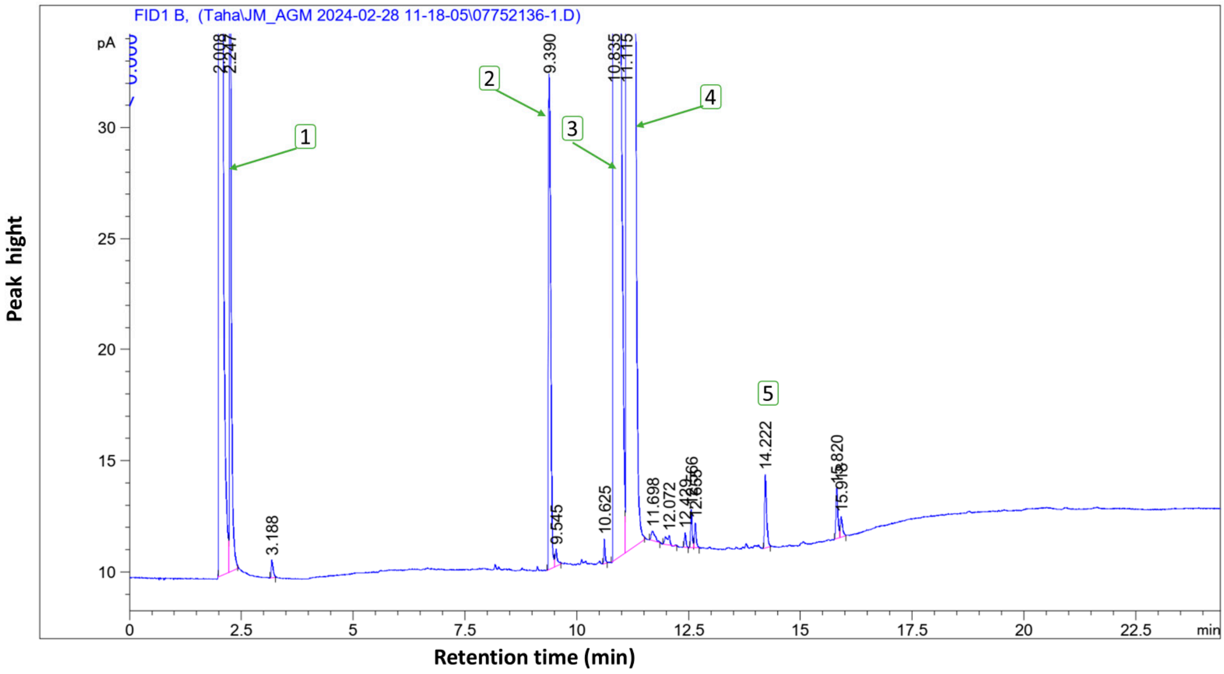

Figure S136. Total ion chromatograms (TICs) from the gas chromatography analysis of the fatty acids in SO_3: Sunflower oil_Exp 3. (1: Internal standard (4-Methyl-2-pentanol); 2: Palmitic acid; 3: Oleic acid; 4: Linoleic acid; 5: Gadoleic acid.

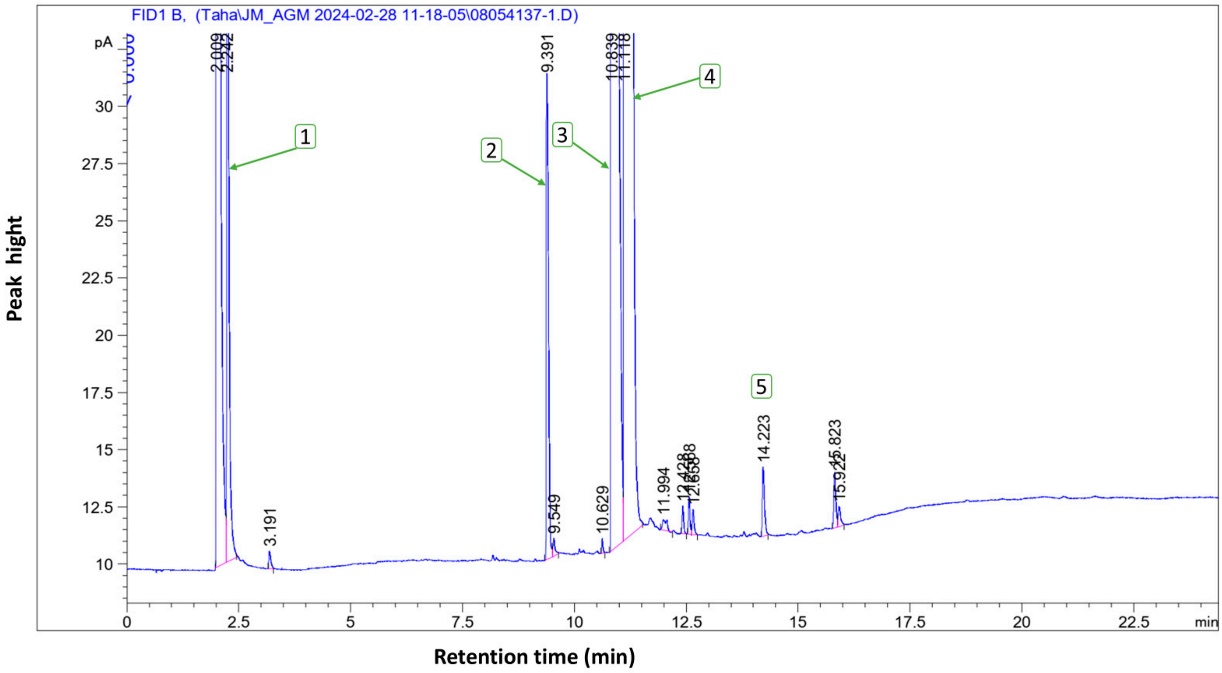

Figure S137. Total ion chromatograms (TICs) from the gas chromatography analysis of the fatty acids in SO_4: Sunflower oil_Exp 4. (1: Internal standard (4-Methyl-2-pentanol); 2: Palmitic acid; 3: Oleic acid; 4: Linoleic acid; 5: Gadoleic acid.

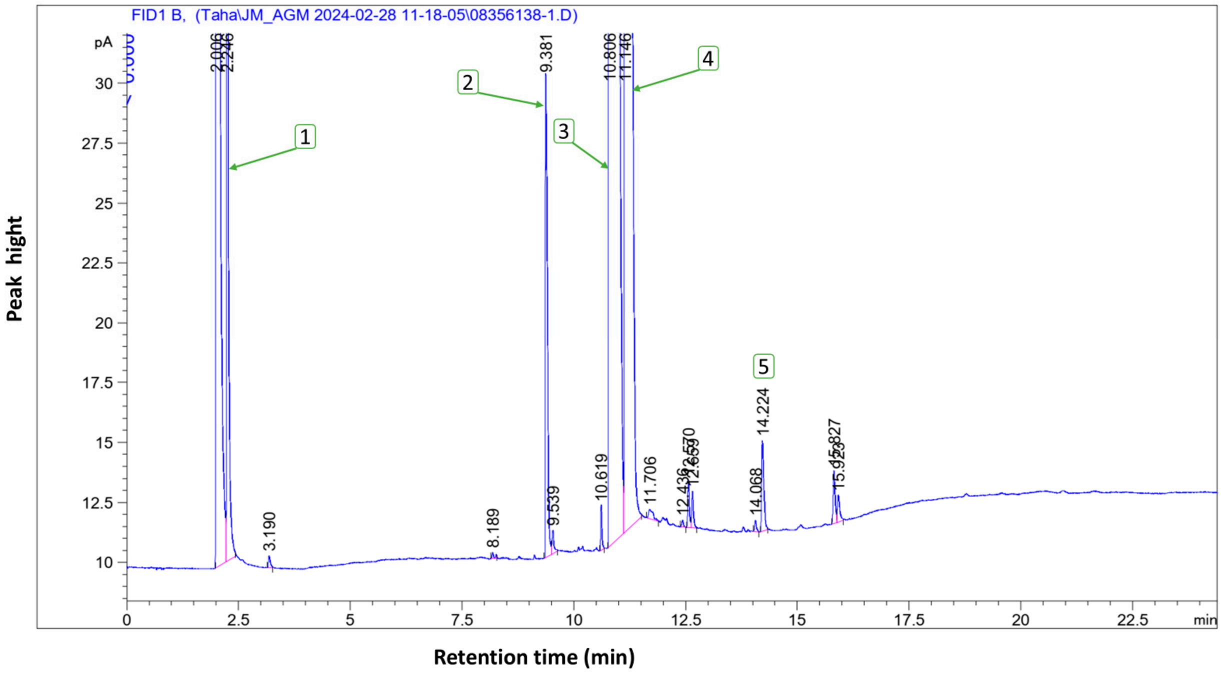

Figure S138. Total ion chromatograms (TICs) from the gas chromatography analysis of the fatty acids in SOHO_C: Sunflower oil_high oleic acid_Contol. (1: Internal standard (4-Methyl-2-pentanol); 2: Palmitic acid; 3: Oleic acid; 4: Linoleic acid; 5: Gadoleic acid.

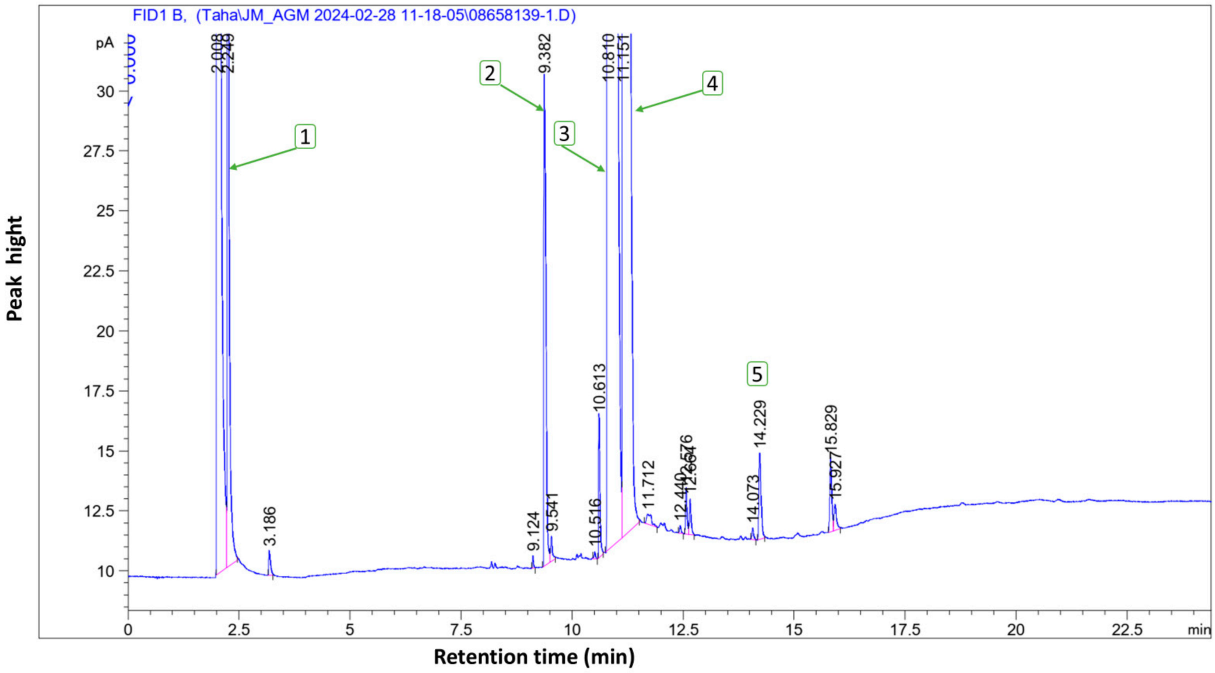

Figure S139. Total ion chromatograms (TICs) from the gas chromatography analysis of the fatty acids in SOHO_1: Sunflower oil_ high oleic acid _Exp 1. (1: Internal standard (4-Methyl-2-pentanol); 2: Palmitic acid; 3: Oleic acid; 4: Linoleic acid; 5: Gadoleic acid.

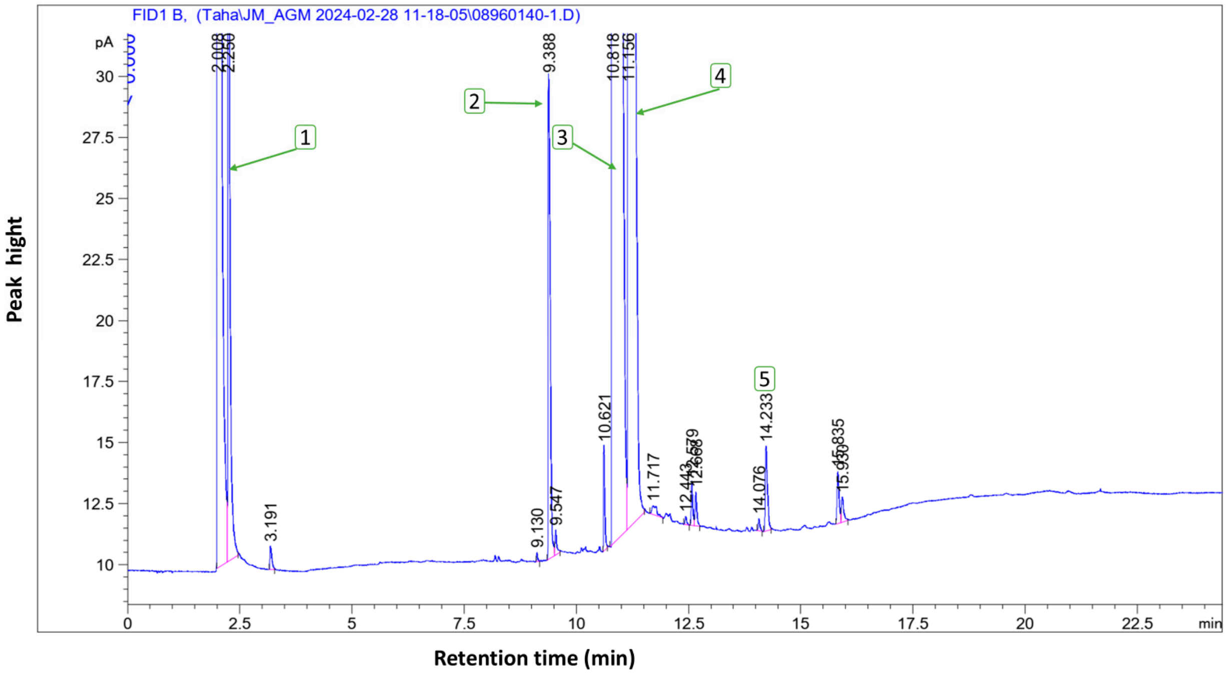

Figure S140. Total ion chromatograms (TICs) from the gas chromatography analysis of the fatty acids in SOHO_2: Sunflower oil_ high oleic acid_Exp 2. (1: Internal standard (4-Methyl-2-pentanol); 2: Palmitic acid; 3: Oleic acid; 4: Linoleic acid; 5: Gadoleic acid.

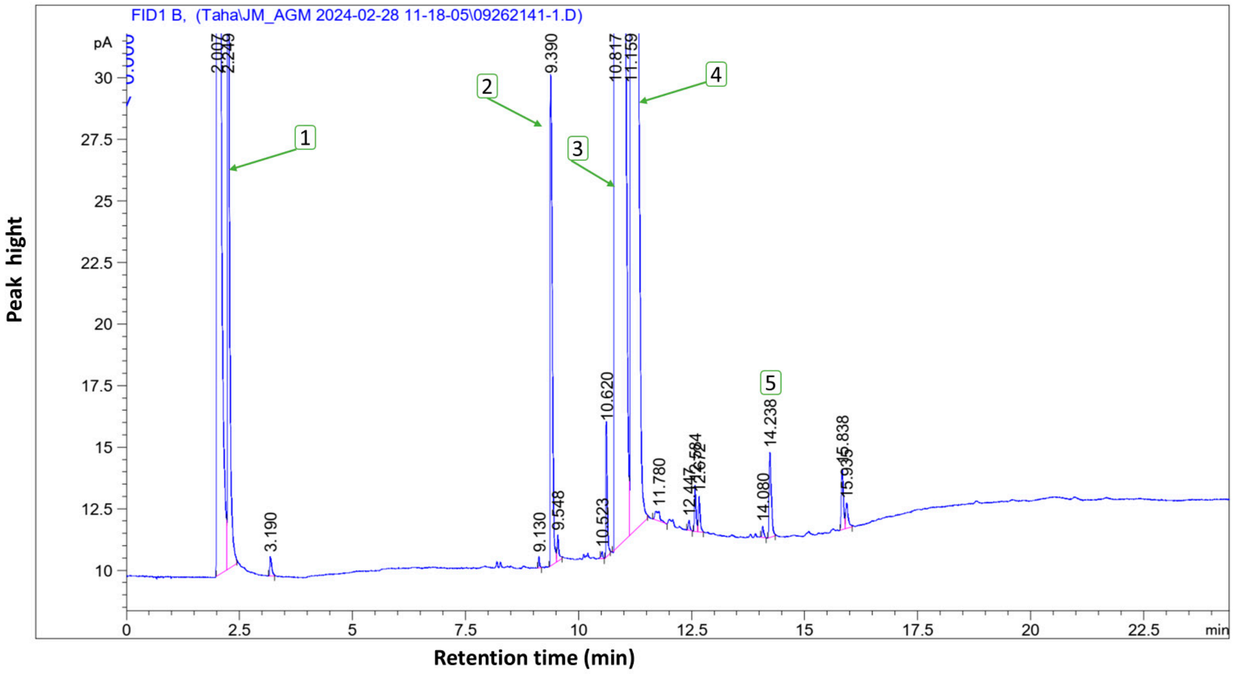

Figure S141. Total ion chromatograms (TICs) from the gas chromatography analysis of the fatty acids in SOHO_3: Sunflower oil_ high oleic acid_Exp 3. (1: Internal standard (4-Methyl-2-pentanol); 2: Palmitic acid; 3: Oleic acid; 4: Linoleic acid; 5: Gadoleic acid.

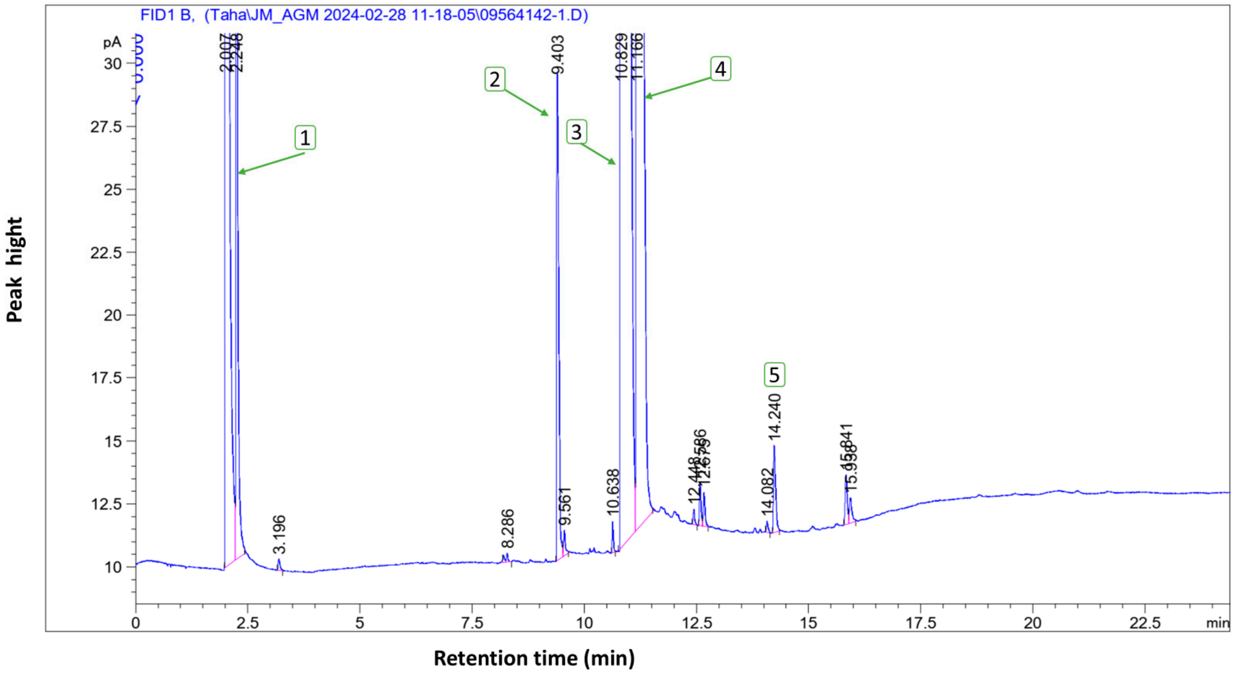

Figure S142. Total ion chromatograms (TICs) from the gas chromatography analysis of the fatty acids in SOHO_4: Sunflower oil_ high oleic acid_Exp 4. (1: Internal standard (4-Methyl-2-pentanol); 2: Palmitic acid; 3: Oleic acid; 4: Linoleic acid; 5: Gadoleic acid.
